# Controllable Synthesis of Trifluoromethyl- or *gem*-Difluorovinyl-containing Analogues of Neonicotinoids by the Reaction of α-(Trifluoromethyl)styrenes with 2-Nitroimino-imidazolidine

**DOI:** 10.3390/molecules28083530

**Published:** 2023-04-17

**Authors:** Jingjing He, Zhudi Sun, Yupian Deng, Ying Liu, Pai Zheng, Song Cao

**Affiliations:** 1 Shanghai Key Laboratory of Chemical Biology, School of Pharmacy, East China University of Science and Technology (ECUST), Shanghai 200237, China; hejingjing97@163.com (J.H.); szd13836710138@163.com (Z.S.); dengyupian981121@163.com (Y.D.); liuying199803@163.com (Y.L.); zhengpai_zp@163.com (P.Z.); 2 Key Laboratory of Organofluorine Chemistry, Shanghai Institute of Organic Chemistry, Chinese Academy of Sciences, Shanghai 200032, China

**Keywords:** α-(trifluoromethyl)styrenes, 2-nitroimino-imidazolidine, trifluoromethyl- or *gem*-difluorovinyl-containing analogues of neonicotinoids

## Abstract

A simple and straightforward addition or defluorination of α-(trifluoromethyl)styrenes with 2-nitroimino-imidazolidine (**2a**), 2-(nitromethylene)imidazolidine (**2b**), 2-cyanoimino-thiazolidine (**2c**), and (*E*)-1-methyl-2-nitroguanidine (**2d**), in a controlled manner, was developed. The hydroamination of α-(trifluoromethyl)styrenes with **2a**, **2b**, **2c**, and **2d** was completed in the presence of DBN at room temperature within 0.5–6 h, affording structurally diverse β-trifluoromethyl-β-arylethyl analogues of neonicotinoids in moderate to good yields. The γ,γ-difluoro-β-arylallyl analogues of neonicotinoids were also successfully synthesized via defluorination of α-(trifluoromethyl)styrenes, with **2a** and **2c** using NaH as base at an elevated temperature together with a prolonged reaction time of 12 h. The method features simple reaction setup, mild reaction conditions, broad substrate scope, high functional group compatibility, and easy scalability.

## 1. Introduction

**Figure 1:**
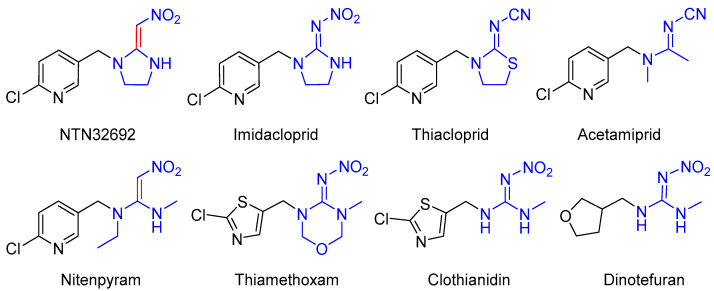
The lead compound NTN32692 and commercialized neonicotinoid insecticides.

Neonicotinoid insecticides, which act as insect nicotinic acetylcholine receptor (nAChR) agonists, are one of the most important classes of insecticides, and are used for crop protection and veterinary pest control due to their supreme insecticidal ability, broad insecticidal spectrum, mammalian safety, and unique mode of action [1,2,3,4]. Since the first commercialized neonicotinoid insecticide, Imidacloprid (IMI), was launched in 1991 by Bayer CropScience, considerable efforts have been made on the development of novel neonicotinoid insecticides with high insecticidal activity and low toxicity to mammalians, and thus other first, second, and third generation neonicotinoids have subsequently been taken into the market (Figure 1) [5,6,7,8].

**Figure 2:**
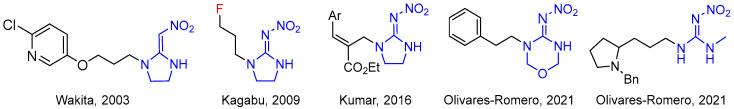
The modifications of linkages of neonicotinoid insecticides [13,14,15,16,17].

Generally, the structure of neonicotinoid insecticides is composed of four segments: an aromatic heterocycle or heteroalicycle, a nitrogen-containing heteroalicycle or guanidine/amidine, an electron-withdrawing functional group, and flexible linkage such as the methylene group (–CH_2_–) [9,10,11,12]. Until now, most research has focused overwhelmingly on the modification of the former three moieties, whereas optimization studies on the methylene group (–CH_2_–) are very scarce and only sporadic examples have been reported (Figure 2) [13,14,15,16,17]. In addition, a survey of the literature reveals that only rare examples of fluorine-containing neonicotinoid insecticides have been described [18].

**Scheme 1:**
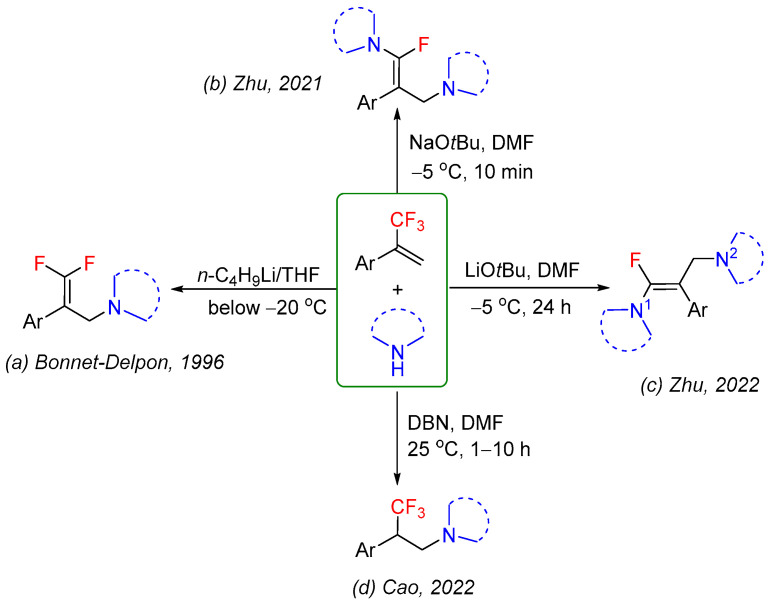
The reaction of α-(trifluoromethyl)styrenes with nitrogen nucleophiles under the different reaction conditions [37,38,39,40].

α-(Trifluoromethyl)styrene and its derivatives are useful and versatile trifluoromethyl-containing building blocks for the synthesis of various fluorine-containing organic molecules [19,20,21,22]. With the development of defluorinative functionalization of α-(trifluoromethyl)styrene, a great number of methods for the synthesis of *gem*-difluoroalkenes have been reported [23,24,25,26]. They became one of the most straightforward approaches to *gem*-difluoroalkenes, which might be ascribed to the fact that the carbon–fluorine of the α-(trifluoromethyl)styrenes easily undergoes β-fluoride elimination [27,28,29,30]. However, addition reaction of α-(trifluoromethyl)styrenes without fluoride elimination remains largely elusive [31,32,33,34,35,36]. The reaction of α-(trifluoromethyl)styrenes with different nitrogen nucleophiles might proceed via three pathways: the S_N_2′ type of defluorinative addition/elimination (Scheme 1a) [37], sequential *ipso*/*γ*-selective defluorinative amination (Scheme 1b,c) [38,39], or hydroamination (Scheme 1d) [40]. Although conceptually simple, the reaction outcomes of α-(trifluoromethyl)styrenes with nitrogen nucleophiles are still mainly based on empirical analysis and experiences, and no general rule for predicting the products has been established. The reaction pathway, product distribution, and regioselectivity of the reaction are remarkably dependent on the electronic nature and position of the substituents on the phenyl ring of α-(trifluoromethyl)styrenes, the type of nitrogen nucleophiles, and the reaction conditions employed.

**Scheme 2:**
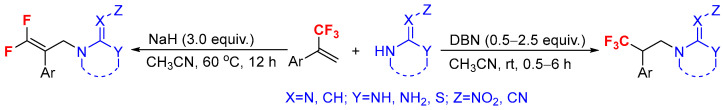
Amination of α-(trifluoromethyl)styrenes with nitrogen nucleophiles (this work).

Based on the above-mentioned considerations, we envisaged that the modification of flexible linkage of neonicotinoid insecticide by the incorporation of fluorine-containing groups into neonicotinoid might be realized through the nucleophilic addition or defluorination reaction between α-(trifluoromethyl)styrenes and nitrogen-containing heteroalicycles or guanidine under controlled reaction conditions. In this paper, we developed a facile and practical method for the synthesis of trifluoromethyl or *gem*-difluorovinyl-containing neonicotinoid analogs via hydroamination or mono-defluorinative amination of α-(trifluoromethyl)styrenes with different nitrogen-containing heteroalicycles or guanidine, respectively, in a controlled manner under basic conditions (Scheme 2).

## 2. Results and Discussion

We began our investigation using the reaction of 4-(3,3,3-trifluoroprop-1-en-2-yl)-1,1′-biphenyl **1a** with 2-nitroimino-imidazolidine **2a** as the model reaction to optimize the reaction conditions (Table 1). Generally, the product distribution of the amination was highly dependent on the base employed. Thus, our first effort focused on the influence of the base on the outcome of this reaction. A mixture of unidentified byproducts was observed when LiHMDS was used as base (entry 1). Other inorganic bases such as KOH, Cs_2_CO_3_, and KO*t*Bu gave a mixture of addition product **3aa** and defluorination **4aa** (entries 2–4). Among various organic bases examined, only DBN was found to be the most acceptable base for this hydroamination, providing **3aa** in 97% yield (entry 13). The competing defluorination reaction was suppressed completely and no defluorinative product **4aa** was detected. When the base was changed from DBN to TMG, TBD, and DBU, the product yields of **3aa** decreased significantly (entries 10–12). Other organic bases such as Et_3_N, TMEDA, DIPEA, DMAP, and DABCO, all resulted in no reaction (entries 5–9).

Further screening of the solvents indicated that CH_3_CN, THF, and NMP could afford excellent yields of **3aa** (entries 13, 18, and 19), whereas the use of other solvents, such as DMSO, CH_2_Cl_2_, and toluene, resulted in lower yields (entries 15–17). When the polar protic solvent CH_3_OH was used as solvent, only a small amount of the desired product **3aa** was formed (entry 14). To our delight, decreasing the amount of DBN from 3.0 to 2.5 equivalents would also provide **3aa** in excellent yield (97%, entry 20). Further decreasing the amount of DBN led to significant decrease in the yield (82%, entry 21). In addition, when the reaction was performed at 25 °C using NaH as a base for 12 h, defluorinative amination product **4aa** was produced in 30% yield, whereas hydroamination product **3aa** was not detected (entry 22). It was pleasing to find that elevating the reaction temperature to 40 °C and 60 °C significantly improved the yield of **4aa**, to 43% and 71%, respectively (entries 23 and 24).

**Scheme 3:**
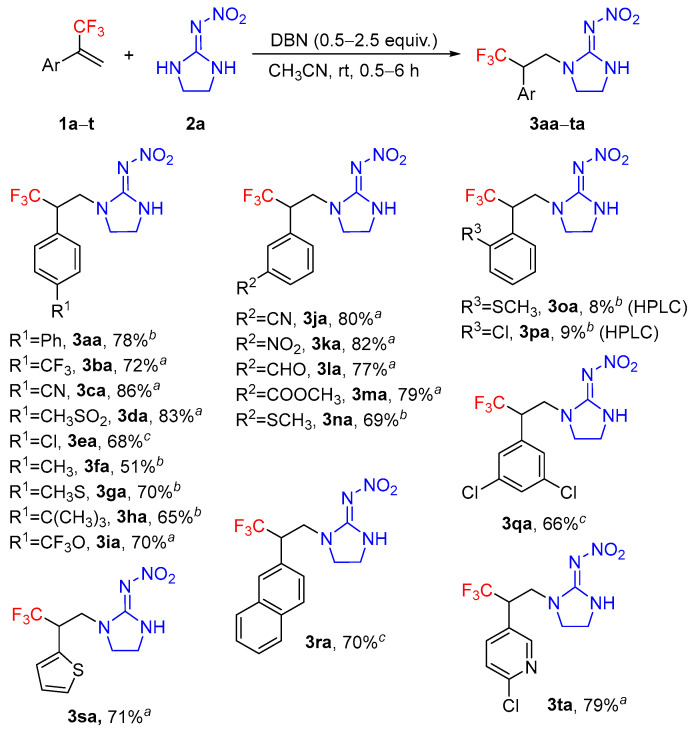
Hydroamination of various α-(trifluoromethyl)styrenes with 2-nitroimino-imidazolidine **2a** *^a^*. *^a^* Reaction conditions: **1a**–**t** (1.0 mmol), **2a** (1.0 mmol), DBN (0.5 equiv., 0.5 mmol), CH_3_CN (3 mL), 25 °C, 0.5 h. *^b^* 2.5 equiv. of DBN was used, 6 h. *^c^* 1.0 equiv. of DBN was used, 3 h. Isolated yields.

With the optimized reaction conditions in hand (Table 1, entry 20), the substrate scope of this novel hydroamination was then investigated. As shown in Scheme 3, when a series of α-(trifluoromethyl)styrenes were treated with 2-nitroimino-imidazolidine **2a**, the reactions proceeded smoothly to deliver the corresponding addition products in moderate to good yields. Generally, α-(trifluoromethyl)styrenes bearing electron-withdrawing groups on the phenyl ring are more favorable for this conversion than electron-donating groups (**3ca** versus **3fa**). However, the α-(trifluoromethyl)styrene having a strong electron-donating group such as CH_3_O was found to be an unsuitable substrate, and only a trace amount of addition product was observed. The reactions exhibited excellent functional group compatibility, and a wide range of functional groups, such as trifluoromethyl, cyano, methylsulfonyl, chloro, methylthio, trifluoromethoxy, nitro, formyl, and ester were well tolerated under the reaction conditions, which may serve as useful reaction handles for further derivatization. The steric effect of an *ortho*-substituent had an obvious influence on the reaction efficiency. Compared to *para*- and *meta*-substituted styrenes, *ortho*-substituted substrates were unreactive and only small amounts of addition products were observed (**3oa** and **3pa**). In addition, 4-(3,3,3-trifluoroprop-1-en-2-yl)-1,1′-biphenyl **1a** and 2-(3,3,3-trifluoroprop-1-en-2-yl)naphthalene **1r** were found to be good substrates for the reaction. Importantly, heterocyclic substrates such as 2-(3,3,3-trifluoroprop-1-en-2-yl)thiophene **1s** and 2-chloro-5-(3,3,3-trifluoroprop-1-en-2-yl)pyridine **1t** were also suitable for this reaction to give the desired products **3sa** and **3ta** in 71% and 79% yields, respectively. It was worth noting that in most cases, 0.5 or 1.0 equivalents of DBN were enough to make the hydroamination reaction proceed efficiently, and good results were achieved.

**Scheme 4:**
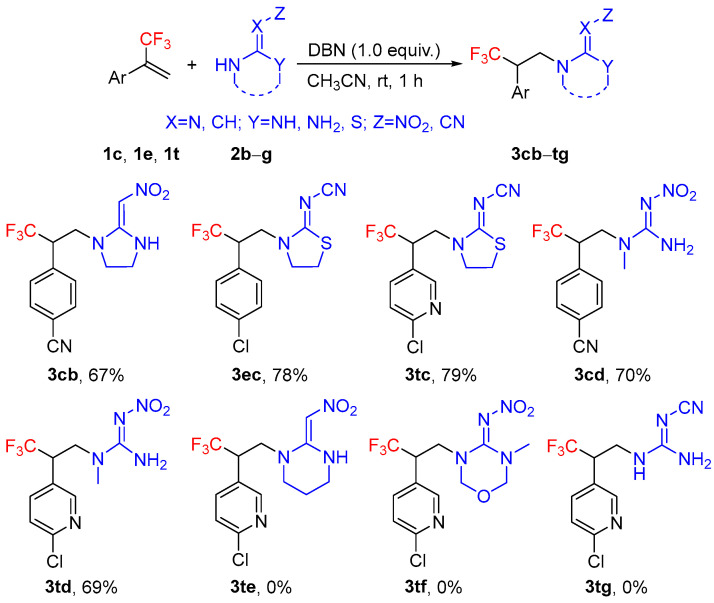
Hydroamination of α-(trifluoromethyl)styrenes with different nitrogen-containing heteroalicycles or guanidine. Reaction conditions: **1c**, **1e**, **1t** (1.0 mmol), **2b**–**g** (1.0 mmol), DBN (1.0 equiv., 1.0 mmol), CH_3_CN (3 mL), 25 °C, 1 h. Isolated yields.

To further verify the scope of this novel hydroamination reaction, four nitrogen-containing heteroalicycles (**2b**, **2c**, **2e**, and **2f**) and two guanidines (**2d** and **2g**) were subjected to the addition reaction with α-(trifluoromethyl)styrenes (**1c**, **1e**, and **1t**) under the optimized reaction conditions (Scheme 4). Gratifyingly, without further optimization of the reaction conditions, the reactions of 2-(nitromethylene)imidazolidine **2b**, 2-cyanoimino-thiazolidine **2c**, and (*E*)-1-methyl-2-nitroguanidine **2d** with α-(trifluoromethyl)styrenes proceeded smoothly and provided corresponding products in moderate to good yields, despite the fact that those substrates are structurally different. However, 2-(nitromethylene)hexahydropyrimidine **2e**, 3-methyl-4-nitroimino-tetrahydro-1,3,5-oxadiazine **2f**, and 2-cyanoguanidine **2g** were poor substrates and failed to furnish the desired products. Therefore, the reaction conditions must be further investigated.

**Scheme 5:**
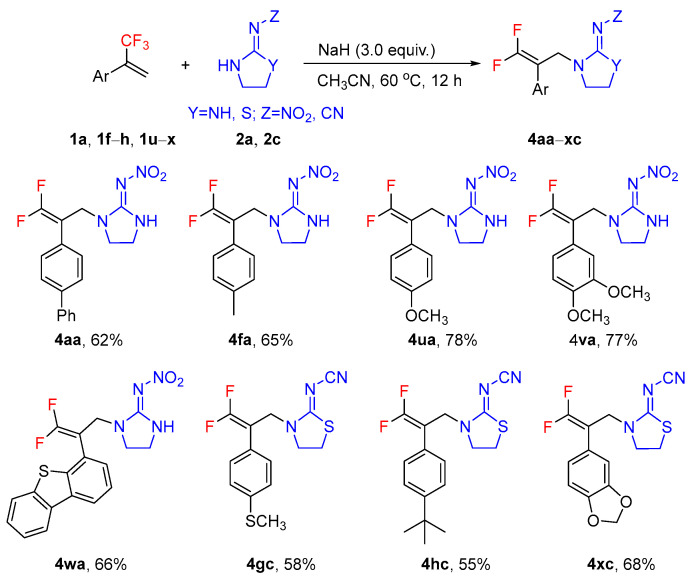
Defluorinative reaction of α-(trifluoromethyl)styrenes with 2-nitroimino-imidazolidine **2a** and 2-cyanoimino-thiazolidine **2c**. Reaction conditions: **1a**, **1f**–**h**, **1u**–**x** (1.0 mmol), **2a**, **2c** (1.0 mmol), NaH (3.0 equiv., 3.0 mmol), CH_3_CN (3 mL), 60 °C, 12 h. The reaction vial was sealed with a septum. Isolated yields.

Subsequently, the defluorinative amination of α-(trifluoromethyl)styrenes with different nitrogen-containing heteroalicycles and guanidines under optimal conditions (using NaH in CH_3_CN at 60 °C for 12 h, Table 1, entry 24) was investigated (Scheme 5). Unfortunately, the scope of the α-(trifluoromethyl)styrenes was rather narrow, where only electron-rich α-(trifluoromethyl)styrenes could furnish *gem*-difluoroalkenes in acceptable yields. Unexpectedly, 4-(3,3,3-trifluoroprop-1-en-2-yl)dibenzo[*b*,*d*]thiophene **1w** could be successfully converted to *gem*-difluoroalkene **4wa** in 66% yield. Furthermore, only 2-nitroimino-imidazolidine **2a** and 2-cyanoimino-thiazolidine **2c** could undergo defluorination reaction efficiently, whereas no reaction occurred when 2-(nitromethylene)imidazolidine **2b**, (*E*)-1-methyl-2-nitroguanidine **2d**, 2-(nitromethylene)hexahydropyrimidine **2e**, 3-methyl-4-nitroimino-tetrahydro-1,3,5-oxadiazine **2f,** and 2-cyanoguanidine **2g** were used as substrates. These results further demonstrated that although the reaction conditions seemed the best, this was not the general case for other substrates. The outcomes of the reactions between α-(trifluoromethyl)styrenes and nitrogen nucleophiles are highly dependent on the structures and electronic properties of the substrates and reaction conditions (see Supplementary Materials).

**Scheme 6:**
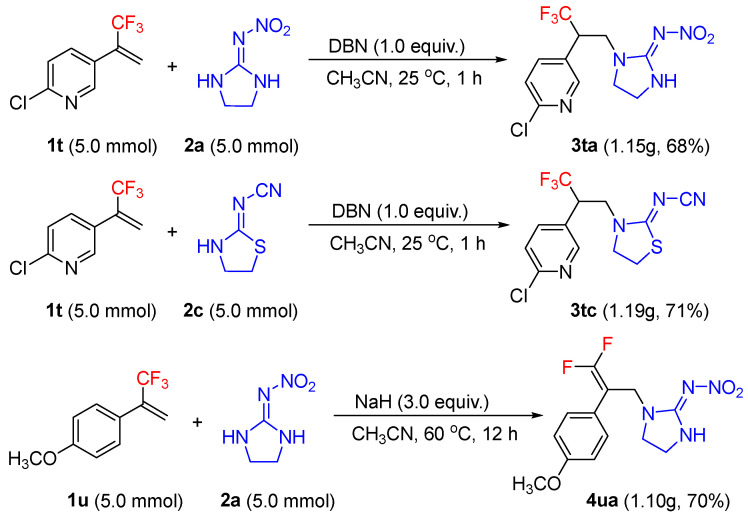
Gram-scale synthesis of **3ta**, **3tc**, and **4ua**.

To prove the preparative usefulness of the developed methods, three scale-up reactions were performed. All reactions were conducted in 5.0 mmol scale. Without further optimization of reaction conditions, the hydroamination of α-(trifluoromethyl)styrenes **1t** with **2a** and **2c**, and the defluorinative reaction of α-(trifluoromethyl)styrenes **1u** with **2a,** were easy to scale-up, however, the desired products were obtained in slightly lower yields (**3ta**, **3tc**, and **4ua**, Scheme 6).

**Scheme 7:**
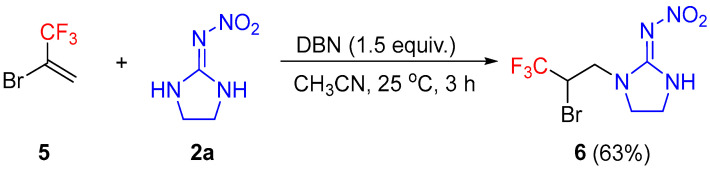
Reaction of 2-bromo-3,3,3-trifluoroprop-1-ene with **2a**.

Surprisingly, the reaction of 2-bromo-3,3,3-trifluoroprop-1-ene **5** with 2-nitroimino-imidazolidine **2a** also proceeded smoothly, affording the addition product **6** in moderate yield (Scheme 7). Notably, the remaining bromo group in the product offers the opportunity for further downstream diversification.

## 3. Materials and Methods

### 3.1. General Information

All solvents were of analytical grade, unless otherwise mentioned, and solvents and reagents were received from commercial suppliers and used without further purification. α-(Trifluoromethyl)styrenes **1** [41,42], 2-(nitromethylene)imidazolidine **2b**, and 2-(nitromethylene)hexahydropyrimidine **2e** were synthesized by previously reported methods [43]. 2-Nitroimino-imidazolidine **2a**, 2-cyanoimino-thiazolidine **2c**, (*E*)-1-methyl-2-nitroguanidine **2d**, 3-methyl-4-nitroimino-tetrahydro-1,3,5-oxadiazine **2f**, and 2-cyanoguanidine **2g** were purchased from commercial sources. All the products obtained in this work are new compounds.

Reactions were stirred using Teflon-coated magnetic stir bars. Elevated temperatures were maintained using thermostat-controlled silicone oil baths. ^1^H NMR and ^13^C NMR spectra were recorded on a 400 spectrometer (400 MHz for ^1^H and 100 MHz for ^13^C, respectively) using TMS as an internal standard. The ^19^F NMR spectra were obtained on a 600 spectrometer (564 MHz) with CF_3_COOH as an internal standard. CDCl_3_, DMSO-*d*_6_, or (CD_3_)_2_CO were used as the NMR solvents. Data for ^1^H, ^13^C, and ^19^F NMR were recorded as follows: chemical shift (δ, ppm), multiplicity (s = singlet, d = doublet, t = triplet, m = multiplet, q = quartet, dd = double of doublet). Coupling constants are reported in hertz (Hz). High resolution mass spectra (HRMS) were recorded on the EI or ESI mode using a TOF mass analyzer. The melting points were measured on an open capillary using EZ-Melt automated melting point apparatus and were not corrected. HPLC were recorded on a Shimadzu LC-20AT. Silica gel (300–400 mesh size) was used for column chromatography. TLC analysis of reaction mixtures was performed using silica gel plates.

### 3.2. General Procedure for the Synthesis of the Target Compounds ***3aa**–**td***

To a glass tube charged with a stirring bar were added DBN (0.5–2.5 equiv.), α-(trifluoromethyl)styrenes (**1a**–**t**, 1.0 mmol), nitrogen nucleophiles **2a**–**d** (1.0 mmol, 1.0 equiv.), and CH_3_CN (3 mL). The reaction was stirred for 0.5–6 h under room temperature (monitored by TLC). After the completion of reaction, the reaction mixture was quenched with a saturated aqueous solution of NH_4_Cl (15 mL) and extracted with ethyl acetate (3 × 15 mL). The organic layer was separated and dried over Na_2_SO_4_, filtered and concentrated in vacuo. The residue was purified by column chromatography on silica gel using *n*-hexane/ethyl acetate (5/1–1/1) as eluent to afford the target compounds **3aa**–**td**.

(*E*)-*N*-(1-(2-([1,1′-Biphenyl]-4-yl)-3,3,3-trifluoropropyl)imidazolidin-2-ylidene)nitramide (**3aa**). White solid, m.p. 135.3–136.8 °C, yield 78% (295.1 mg); ^1^H NMR (400 MHz, CDCl_3_) δ 8.03 (s, 1H), 7.63–7.58 (m, 4H), 7.47–7.42 (m, 4H), 7.39–7.35 (m, 1H), 3.98–3.81 (m, 3H), 3.67–3.56 (m, 2H), 3.55–3.48 (m, 1H), 3.24 (q, *J* = 9.2 Hz, 1H); ^13^C NMR (100 MHz, CDCl_3_) δ 160.2, 140.8, 138.9, 129.9, 129.8, 128.4, 127.9, 126.7, 126.6, 126.0, 124.8 (q, ^1^*J*_CF_ = 279.0 Hz), 46.9 (q, ^2^*J*_CF_ = 26.2 Hz), 45.6, 43.5 (d, ^3^*J*_CF_ = 2.6 Hz), 40.5; ^19^F NMR (564 MHz, CDCl_3_) δ −67.9 (d, *J* = 7.9 Hz, 3F); HRMS (EI): calcd for C_18_H_17_F_3_N_4_O_2_ [M]^+^: 378.1304, found: 378.1301.

(*E*)-*N*-(1-(3,3,3-Trifluoro-2-(4-(trifluoromethyl)phenyl)propyl)imidazolidin-2-ylidene)nitramide (**3ba**). White solid, m.p. 104.4–106.0 °C, yield 72% (266.8 mg); ^1^H NMR (400 MHz, CDCl_3_) δ 8.04 (s, 1H), 7.65 (d, *J* = 8.0 Hz, 2H), 7.51 (d, *J* = 8.0 Hz, 2H), 4.00–3.94 (m, 1H), 3.92–3.82 (m, 2H), 3.68–3.63 (m, 2H), 3.57–3.51 (m, 1H), 3.27 (q, *J* = 9.2 Hz, 1H); ^13^C NMR (100 MHz, CDCl_3_) δ 161.3, 136.1, 131.3 (q, ^2^*J*_CF_ = 32.6 Hz), 129.6, 126.0 (q, ^3^*J*_CF_ = 3.6 Hz), 125.4 (q, ^1^*J*_CF_ = 278.8 Hz), 123.8 (q, ^1^*J*_CF_ = 270.6 Hz), 48.1 (q, ^2^*J*_CF_ = 26.4 Hz), 46.7, 44.5 (d, ^3^*J*_CF_ = 2.1 Hz), 41.6; ^19^F NMR (564 MHz, CDCl_3_) δ −62.8 (s, 3F), −67.9 (d, *J* = 7.9 Hz, 3F); HRMS (EI): calcd for C_13_H_12_F_6_N_4_O_2_ [M]^+^: 370.0864, found: 370.0868.

(*E*)-*N*-(1-(2-(4-Cyanophenyl)-3,3,3-trifluoropropyl)imidazolidin-2-ylidene)nitramide (**3ca**). White solid, m.p. 126.0–127.4 °C, yield 86% (281.9 mg); ^1^H NMR (400 MHz, CDCl_3_) δ 8.05 (s, 1H), 7.67 (d, *J* = 8.4 Hz, 2H), 7.50 (d, *J* = 8.4 Hz, 2H), 4.00–3.94 (m, 1H), 3.91–3.80 (m, 2H), 3.66 (t, *J* = 9.0 Hz, 2H), 3.59–3.52 (m, 1H), 3.30 (q, *J* = 9.2 Hz, 1H); ^13^C NMR (100 MHz, CDCl_3_) δ 161.3, 137.3, 132.7, 130.0, 125.4 (q, ^1^*J*_CF_ = 279.2 Hz), 118.1, 113.1, 48.2 (q, ^2^*J*_CF_ = 26.5 Hz), 46.7, 44.4 (d, ^3^*J*_CF_ = 2.4 Hz), 41.7; ^19^F NMR (564 MHz, CDCl_3_) δ −67.7 (d, *J* = 7.9 Hz, 3F); HRMS (EI): calcd for C_13_H_12_F_3_N_5_O_2_ [M]^+^: 327.0943, found: 327.0947.

(*E*)-*N*-(1-(3,3,3-Trifluoro-2-(4-(methylsulfonyl)phenyl)propyl)imidazolidin-2-ylidene)nitramide (**3da**). White solid, m.p. 123.9–145.1 °C, yield 83% (315.6 mg); ^1^H NMR (400 MHz, CDCl_3_) δ 8.04 (s, 1H), 7.94 (d, *J* = 8.0 Hz, 2H), 7.59 (d, *J* = 8.0 Hz, 2H), 4.03–3.97 (m, 1H), 3.94–3.81 (m, 2H), 3.65 (t, *J* = 8.8 Hz, 2H), 3.59–3.53 (m, 1H), 3.31 (q, *J* = 8.8 Hz, 1H), 3.06 (s, 3H); ^13^C NMR (100 MHz, CDCl_3_) δ 161.3, 141.3, 138.3, 130.2, 128.1, 125.4 (q, ^1^*J*_CF_ = 279.2 Hz), 48.2 (q, ^2^*J*_CF_ = 26.6 Hz), 46.7, 44.6 (d, ^3^*J*_CF_ = 2.1 Hz), 44.4, 41.7; ^19^F NMR (564 MHz, CDCl_3_) δ −67.5 (d, *J* = 9.0 Hz, 3F); HRMS (EI): calcd for C_13_H_15_F_3_N_4_O_4_S [M]^+^: 380.0766, found: 380.0764.

(*E*)-*N*-(1-(2-(4-Chlorophenyl)-3,3,3-trifluoropropyl)imidazolidin-2-ylidene)nitramide (**3ea**). White solid, m.p. 117.0–118.9 °C, yield 68% (228.7 mg); ^1^H NMR (400 MHz, CDCl_3_) δ 8.02 (s, 1H), 7.35 (d, *J* = 8.4 Hz, 2H), 7.29 (d, *J* = 8.4 Hz, 2H), 3.89–3.78 (m, 3H), 3.65–3.60 (m, 2H), 3.53–3.46 (m, 1H), 3.24 (q, *J* = 8.8 Hz, 1H); ^13^C NMR (100 MHz, CDCl_3_) δ 161.2, 135.1, 130.5, 130.4, 129.3, 125.6 (q, ^1^*J*_CF_ = 279.0 Hz), 47.6 (q, ^2^*J*_CF_ = 26.4 Hz), 46.6, 44.4 (d, ^3^*J*_CF_ = 2.4 Hz), 41.6; ^19^F NMR (564 MHz, CDCl_3_) δ −68.2 (d, *J* = 7.3 Hz, 3F); HRMS (EI): calcd for C_12_H_12_ClF_3_N_4_O_2_ [M]^+^: 336.0601, found: 336.0600.

(*E*)-*N*-(1-(3,3,3-Trifluoro-2-(p-tolyl)propyl)imidazolidin-2-ylidene)nitramide (**3fa**). White solid, m.p. 121.5–122.9 °C, yield 51% (161.2 mg); ^1^H NMR (400 MHz, CDCl_3_) δ 8.01 (s, 1H), 7.23 (d, *J* = 8.0 Hz, 2H), 7.18 (d, *J* = 8.0 Hz, 2H), 3.95–3.89 (m, 1H), 3.82–3.73 (m, 2H), 3.63–3.57 (m, 2H), 3.50–3.43 (m, 1H), 3.17 (q, *J* = 9.2 Hz, 1H), 2.34 (s, 3H); ^13^C NMR (100 MHz, CDCl_3_) δ 161.3, 138.9, 129.7, 128.9, 128.8, 129.5, 129.4, 125.8 (q, ^1^*J*_CF_ = 278.8 Hz), 47.9 (q, ^2^*J*_CF_ = 26.0 Hz), 46.6, 44.5 (d, ^3^*J*_CF_ = 2.4 Hz), 41.5, 21.1; ^19^F NMR (564 MHz, CDCl_3_) δ −68.2 (d, *J* = 7.9 Hz, 3F); HRMS (EI): calcd for C_13_H_15_F_3_N_4_O_2_ [M]^+^: 316.1147, found: 316.1149.

(*E*)-*N*-(1-(3,3,3-Trifluoro-2-(4-(methylthio)phenyl)propyl)imidazolidin-2-ylidene)nitramide (**3ga**). White solid, m.p. 132.4–134.0 °C, yield 70% (243.7 mg); ^1^H NMR (400 MHz, CDCl_3_) δ 8.06 (s, 1H), 7.32–7.28 (m, 4H), 3.98–3.91 (m, 1H), 3.87–3.79 (m, 2H), 3.68–3.61 (m, 2H), 3.55–3.48 (m, 1H), 3.26 (q, *J* = 9.2 Hz, 1H), 2.52 (s, 3H); ^13^C NMR (100 MHz, CDCl_3_) δ 161.3, 140.0, 129.4, 128.4, 126.6, 125.7 (q, ^1^*J*_CF_ = 278.8 Hz), 47.8 (q, ^2^*J*_CF_ = 26.2 Hz), 46.6, 44.4 (d, ^3^*J*_CF_ = 2.5 Hz), 41.6, 15.3; ^19^F NMR (564 MHz, CDCl_3_) δ −68.2 (d, *J* = 8.5 Hz, 3F); HRMS (EI): calcd for C_13_H_15_F_3_N_4_O_2_S [M]^+^: 348.0868, found: 348.0864.

(*E*)-*N*-(1-(2-(4-(*tert*-Butyl)phenyl)-3,3,3-trifluoropropyl)imidazolidin-2-ylidene)nitramide (**3ha**). White solid, m.p. 157.8–159.1 °C, yield 65% (233.0 mg); ^1^H NMR (400 MHz, CDCl_3_) δ 8.02 (s, 1H), 7.38 (d, *J* = 8.4 Hz, 2H), 7.25 (d, *J* = 8.0 Hz, 2H), 3.95–3.90 (m, 1H), 3.83–3.73 (m, 2H), 3.67–3.55 (m, 2H), 3.52–3.45 (m, 1H), 3.19 (q, *J* = 9.6 Hz, 1H), 1.30 (s, 9H); ^13^C NMR (100 MHz, CDCl_3_) δ 160.2, 151.0, 127.8, 127.6, 124.9, 124.8 (q, ^1^*J*_CF_ = 278.8 Hz), 46.7 (q, ^2^*J*_CF_ = 26.1 Hz), 45.6, 43.5 (d, ^3^*J*_CF_ = 2.7 Hz), 40.5, 33.6, 30.2; ^19^F NMR (564 MHz, CDCl_3_) δ −68.1 (d, *J* = 7.9 Hz, 3F); HRMS (EI): calcd for C_16_H_21_F_3_N_4_O_2_ [M]^+^: 358.1617, found: 358.1615.

(*E*)-*N*-(1-(3,3,3-Trifluoro-2-(4-(trifluoromethoxy)phenyl)propyl)imidazolidin-2-ylidene)nitramide (**3ia**). White solid, m.p. 110.5–112.8 °C, yield 70% (270.1 mg); ^1^H NMR (400 MHz, CDCl_3_) δ 8.03 (s, 1H), 7.40 (d, *J* = 8.8 Hz, 2H), 7.23 (d, *J* = 8.4 Hz, 2H), 3.94–3.78 (m, 3H), 3.70–3.60 (m, 2H), 3.57–3.50 (m, 1H), 3.26 (q, *J* = 9.2 Hz, 1H); ^13^C NMR (100 MHz, CDCl_3_) δ 161.3, 149.6, 130.6, 125.6 (q, ^1^*J*_CF_ = 278.5 Hz), 121.3, 120.4 (q, ^1^*J*_CF_ = 256.4 Hz), 47.6 (q, ^2^*J*_CF_ = 26.4 Hz), 46.7, 44.6 (d, ^3^*J*_CF_ = 2.4 Hz), 41.6; ^19^F NMR (564 MHz, CDCl_3_) δ −57.9 (s, 3F), −68.2 (d, *J* = 9.6 Hz, 3F); HRMS (EI): calcd for C_13_H_12_F_6_N_4_O_3_ [M]^+^: 386.0814, found: 386.0816.

(*E*)-*N*-(1-(2-(3-Cyanophenyl)-3,3,3-trifluoropropyl)imidazolidin-2-ylidene)nitramide (**3ja**). White solid, m.p. 111.5–112.9 °C, yield 80% (262.1 mg); ^1^H NMR (400 MHz, CDCl_3_) δ 8.05 (s, 1H), 7.68–7.65 (m, 3H), 7.53 (*J* = 8.0 Hz, 1H), 3.99–3.80 (m, 3H), 3.71–3.67 (m, 2H), 3.63–3.57 (m, 1H), 3.34 (q, *J* = 9.2 Hz, 1H); ^13^C NMR (100 MHz, CDCl_3_) δ 161.3, 133.7, 133.6, 132.7, 132.6, 130.0, 125.4 (q, ^1^*J*_CF_ = 278.9 Hz), 118.1, 113.3, 47.9 (q, ^2^*J*_CF_ = 26.5 Hz), 46.7, 44.5 (d, ^3^*J*_CF_ = 2.1 Hz), 41.7; ^19^F NMR (564 MHz, CDCl_3_) δ −67.9 (d, *J* = 7.9 Hz, 3F); HRMS (EI): calcd for C_13_H_12_F_3_N_5_O_2_ [M]^+^: 327.0943, found: 327.0940.

(*E*)-*N*-(1-(3,3,3-Trifluoro-2-(3-nitrophenyl)propyl)imidazolidin-2-ylidene)nitramide (**3ka**). White solid, m.p. 121.6–123.2 °C, yield 82% (285.0 mg); ^1^H NMR (400 MHz, CDCl_3_) δ 8.23 (t, *J* = 2.4 Hz, 2H), 8.04 (s, 1H), 7.77 (d, *J* = 7.6 Hz, 1H), 7.61 (t, *J* = 8.4 Hz, 1H), 4.08–3.94 (m, 2H), 3.89–3.83 (m, 1H), 3.72–3.60 (m, 3H), 3.43–3.36 (m, 1H); ^13^C NMR (100 MHz, CDCl_3_) δ 161.3, 148.4, 135.5, 134.0, 130.2, 125.4 (q, ^1^*J*_CF_ = 279.0 Hz), 124.1, 124.0, 47.9 (q, ^2^*J*_CF_ = 26.5 Hz), 46.7, 44.3 (d, ^3^*J*_CF_ = 2.2 Hz), 41.7; ^19^F NMR (564 MHz, CDCl_3_) δ −67.9 (d, *J* = 9.6 Hz, 3F); HRMS (EI): calcd for C_12_H_12_F_3_N_5_O_4_ [M]^+^: 347.0841, found: 347.0843.

(*E*)-*N*-(1-(3,3,3-Trifluoro-2-(3-formylphenyl)propyl)imidazolidin-2-ylidene)nitramide (**3la**). White solid, m.p. 123.7–124.8 °C, yield 77% (254.4 mg); ^1^H NMR (400 MHz, CDCl_3_) δ 10.01 (s, 1H), 8.03 (s, 1H), 7.88 (d, *J* = 6.4 Hz, 2H), 7.67–7.56 (m, 2H), 4.01–3.95 (m, 1H), 3.92–3.85 (m, 2H), 3.68–3.54 (m, 3H), 3.33 (q, *J* = 8.8 Hz, 1H); ^13^C NMR (100 MHz, CDCl_3_) δ 191.7, 161.3, 136.9, 135.1, 133.3, 130.5, 129.9, 129.8, 125.6 (q, ^1^*J*_CF_ = 278.9 Hz), 48.0 (q, ^2^*J*_CF_ = 26.4 Hz), 46.6, 44.4 (d, ^3^*J*_CF_ = 2.2 Hz), 41.6; ^19^F NMR (564 MHz, CDCl_3_) δ −67.9 (d, *J* = 7.9 Hz, 3F); HRMS (EI): calcd for C_13_H_13_F_3_N_4_O_3_ [M]^+^: 330.0940, found: 330.0945.

Methyl (*E*)-3-(1,1,1-trifluoro-3-(2-(nitroimino)imidazolidin-1-yl)propan-2-yl)benzoate (**3ma**). White solid, m.p. 125.5–126.8 °C, yield 79% (284.7 mg). ^1^H NMR (400 MHz, CDCl_3_) δ 8.04 (s, 1H), 8.02 (s, 2H), 7.57 (d, *J* = 7.6 Hz, 1H), 7.47 (t, *J* = 7.6 Hz, 1H), 3.96–3.86 (m, 3H), 3.91 (s, 3H), 3.64–3.57 (m, 2H), 3.55–3.48 (m, 1H), 3.25 (q, *J* = 8.8 Hz, 1H); ^13^C NMR (100 MHz, CDCl_3_) δ 166.4, 161.3, 133.7, 132.5, 131.0, 130.2, 130.1, 129.3, 125.6 (q, ^1^*J*_CF_ = 278.8 Hz), 52.4, 48.0 (q, ^2^*J*_CF_ = 26.2 Hz), 46.6, 44.3 (d, ^3^*J*_CF_ = 2.3 Hz), 41.6; ^19^F NMR (564 MHz, CDCl_3_) δ −68.1 (d, *J* = 7.9 Hz, 3F); HRMS (EI): calcd for C_14_H_15_F_3_N_4_O_4_ [M]^+^: 360.1045, found: 360.1042.

(*E*)-*N*-(1-(3,3,3-Trifluoro-2-(3-(methylthio)phenyl)propyl)imidazolidin-2-ylidene)nitramide (**3na**). White solid, m.p. 142.6–144.0 °C, yield 69% (240.3 mg); ^1^H NMR (400 MHz, CDCl_3_) δ 7.97 (s, 1H), 7.23 (d, *J* = 8.4 Hz, 1H), 7.19–7.15 (m, 2H), 7.05 (d, *J* = 7.2 Hz, 1H), 3.85–3.73 (m, 3H), 3.60–3.52 (m, 2H), 3.48–3.41 (m, 1H), 3.17 (q, *J* = 9.2 Hz, 1H), 2.43 (s, 3H); ^13^C NMR (100 MHz, CDCl_3_) δ 161.2, 139.9, 132.8, 129.4, 126.8, 126.7, 125.7 (q, ^1^*J*_CF_ = 279.0 Hz), 125.5, 48.1 (q, ^2^*J*_CF_ = 26.2 Hz), 46.6, 44.4 (d, ^3^*J*_CF_ = 2.4 Hz), 41.6, 15.5; ^19^F NMR (564 MHz, CDCl_3_) δ −67.9 (d, *J* = 7.3 Hz, 3F); HRMS (EI): calcd for C_13_H_15_F_3_N_4_O_2_S [M]^+^: 348.0868, found: 348.0864.

(*E*)-*N*-(1-(3,3,3-Trifluoro-2-(thiophen-2-yl)propyl)imidazolidin-2-ylidene)nitramide (**3qa**). White solid, m.p. 114.3–116.2 °C, yield 71% (218.8 mg); ^1^H NMR (400 MHz, CDCl_3_) δ 8.04 (s, 1H), 7.33 (d, *J* = 5.2 Hz, 1H), 7.11 (d, *J* = 3.2 Hz, 1H), 7.03 (dd, *J*_1_ = 5.2 Hz, *J*_2_ = 4.0 Hz, 1H), 4.26–4.16 (m, 1H), 4.02–3.97 (m, 1H), 3.71–3.48 (m, 4H), 3.15 (q, *J* = 9.2 Hz, 1H); ^13^C NMR (100 MHz, CDCl_3_) δ 161.2, 133.4, 128.4, 127.4, 126.5, 125.1 (q, ^1^*J*_CF_ = 279.0 Hz), 46.9, 45.7 (d, ^3^*J*_CF_ = 2.4 Hz), 43.8 (q, ^2^*J*_CF_ = 27.6 Hz), 41.7; ^19^F NMR (564 MHz, CDCl_3_) δ −74.1 (d, *J* = 9.6 Hz, 3F); HRMS (EI): calcd for C_10_H_11_F_3_N_4_O_2_S [M]^+^: 308.0555, found: 308.0551.

(*E*)-*N*-(1-(3,3,3-Trifluoro-2-(naphthalen-2-yl)propyl)imidazolidin-2-ylidene)nitramide (**3ra**). White solid, m.p. 128.6–130.1 °C, yield 70% (246.6 mg); ^1^H NMR (400 MHz, CDCl_3_) δ 7.97 (s, 1H), 7.88–7.84 (m, 4H), 7.54–7.51 (m, 2H), 7.47 (d, *J* = 8.4 Hz, 1H), 4.04–4.00 (m, 2H), 3.95–3.88 (m, 1H), 3.56–3.46 (m, 3H), 3.19–3.12 (m, 1H); ^13^C NMR (100 MHz, CDCl_3_) δ 161.3, 133.3, 133.2, 129.4, 129.0, 128.9, 128.1, 127.7, 126.9, 126.8, 125.9 (q, ^1^*J*_CF_ = 279.2 Hz), 125.8, 48.4 (q, ^2^*J*_CF_ = 26.1 Hz), 46.6, 44.5 (d, ^3^*J*_CF_ = 2.3 Hz), 41.5; ^19^F NMR (564 MHz, CDCl_3_) δ −67.8 (d, *J* = 7.3 Hz, 3F); HRMS (EI): calcd for C_16_H_15_F_3_N_4_O_2_ [M]^+^: 352.1147, found: 352.1150.

(*E*)-*N*-(1-(2-(3,5-Dichlorophenyl)-3,3,3-trifluoropropyl)imidazolidin-2-ylidene)nitramide (**3sa**). White solid, m.p. 166.4–168.1 °C, yield 66% (244.8 mg); ^1^H NMR (400 MHz, acetone-*d*_6_) δ 8.47 (s, 1H), 7.60 (s, 2H), 7.54 (t, *J* = 1.6 Hz, 1H), 4.30–4.24 (m, 1H), 4.07–3.93 (m, 2H), 3.74–3.67 (m, 3H), 3.54–3.50 (m, 1H); ^13^C NMR (100 MHz, acetone-*d*_6_) δ 161.4, 136.5, 135.0, 128.8, 128.3, 125.9 (q, ^1^*J*_CF_ = 278.3 Hz), 47.1 (q, ^2^*J*_CF_ = 26.0 Hz), 46.2, 43.4 (d, ^3^*J*_CF_ = 2.6 Hz), 41.8; ^19^F NMR (564 MHz, acetone-*d*_6_) δ −68.9 (d, *J* = 7.9 Hz, 3F); HRMS (EI): calcd for C_12_H_11_Cl_2_F_3_N_4_O_2_ [M]^+^: 370.0211, found: 370.0212.

(*E*)-*N*-(1-(2-(6-Chloropyridin-3-yl)-3,3,3-trifluoropropyl)imidazolidin-2-ylidene)nitramide (**3ta**). White solid, m.p. 112.4–113.9 °C, yield 79% (266.5 mg); ^1^H NMR (400 MHz, CDCl_3_) δ 8.34 (d, *J* = 2.0 Hz, 1H), 8.06 (s, 1H), 7.76 (dd, *J*_1_ = 8.4 Hz, *J*_2_ = 2.0 Hz, 1H), 7.38 (d, *J* = 8.4 Hz, 1H), 3.99–3.89 (m, 2H), 3.82–3.77 (m, 1H), 3.73–3.66 (m, 2H), 3.63–3.57 (m, 1H), 3.43–3.36 (m, 1H); ^13^C NMR (100 MHz, CDCl_3_) δ 161.3, 152.4, 150.3, 139.1, 126.9, 125.3 (q, ^1^*J*_CF_ = 279.0 Hz), 124.7, 46.5, 45.4 (q, ^2^*J*_CF_ = 26.8 Hz), 44.1 (d, ^3^*J*_CF_ = 1.7 Hz), 41.6; ^19^F NMR (564 MHz, CDCl_3_) δ −72.9 (d, *J* = 7.3 Hz, 3F); HRMS (EI): calcd for C_11_H_11_ClF_3_N_5_O_2_ [M]^+^: 337.0553, found: 337.0554.

(*E*)-4-(1,1,1-Trifluoro-3-(2-(nitromethylene)imidazolidin-1-yl)propan-2-yl)benzonitrile (**3cb**). White solid, m.p. 132.4–133.9 °C, yield 67% (218.4 mg); ^1^H NMR (400 MHz, CDCl_3_) δ 8.55 (s, 1H), 7.66 (d, *J* = 8.0 Hz, 2H), 7.46 (d, *J* = 8.0 Hz, 2H), 6.40 (s, 1H), 3.79–3.71 (m, 2H), 3.63–3.47 (m, 4H), 3.22 (q, *J* = 8.8 Hz, 1H); ^13^C NMR (100 MHz, CDCl_3_) δ 159.1, 137.2, 133.0, 129.8, 125.2 (q, ^1^*J*_CF_ = 279.1 Hz), 118.0, 113.4, 96.3, 49.5, 48.6 (q, ^2^*J*_CF_ = 26.6 Hz), 45.8 (d, ^3^*J*_CF_ = 1.7 Hz), 42.5; ^19^F NMR (564 MHz, CDCl_3_) δ −67.7 (d, *J* = 7.9 Hz, 3F); HRMS (EI): calcd for C_14_H_13_F_3_N_4_O_2_ [M]^+^: 326.0991, found: 326.0993.

(*Z*)-*N*-(3-(2-(4-Chlorophenyl)-3,3,3-trifluoropropyl)thiazolidin-2-ylidene)cyanamide (**3ec**). White solid, m.p. 124.8–126.2 °C, yield 78% (259.9 mg). ^1^H NMR (400 MHz, CDCl_3_) δ 7.40 (d, *J* = 8.4 Hz, 2H), 7.28 (d, *J* = 8.0 Hz, 2H), 4.04–3.91 (m, 2H), 3.83–3.75 (m, 2H), 3.47–3.41 (m, 1H), 3.32–3.25 (m, 1H), 3.21–3.15 (m, 1H); ^13^C NMR (100 MHz, CDCl_3_) δ 174.8, 135.4, 130.4, 130.2, 129.5, 125.5 (q, ^1^*J*_CF_ = 278.8 Hz), 116.7, 54.1, 46.9 (q, ^2^*J*_CF_ = 26.8 Hz), 46.6 (d, ^3^*J*_CF_ = 2.3 Hz), 27.8; ^19^F NMR (564 MHz, CDCl_3_) δ −68.1 (d, *J* = 9.0 Hz, 3F); HRMS (EI): calcd for C_13_H_11_ClF_3_N_3_S [M]^+^: 333.0314, found: 333.0311.

(*Z*)-*N*-(3-(2-(6-Chloropyridin-3-yl)-3,3,3-trifluoropropyl)thiazolidin-2-ylidene)cyanamide (**3tc**). White solid, m.p. 122.1–123.7 °C, yield 79% (264.1 mg). ^1^H NMR (400 MHz, CDCl_3_) δ 8.34 (s, 1H), 7.69 (d, *J* = 8.4 Hz, 1H), 7.40 (d, *J* = 8.4 Hz, 1H), 4.02–3.85 (m, 4H), 3.67–3.60 (m, 1H), 3.36–3.25 (m, 2H); ^13^C NMR (100 MHz, CDCl_3_) δ 175.3, 152.6, 150.1, 138.9, 126.8, 125.2 (q, ^1^*J*_CF_ = 279.0 Hz), 124.9, 116.4, 53.9, 46.1 (d, ^3^*J*_CF_ = 2.0 Hz), 44.8 (q, ^2^*J*_CF_ = 27.3 Hz), 27.8; ^19^F NMR (564 MHz, CDCl_3_) δ −68.0 (d, *J* = 7.9 Hz, 3F); HRMS (EI): calcd for C_12_H_10_ClF_3_N_4_S [M]^+^: 334.0267, found: 334.0269.

(*E*)-1-(2-(4-Cyanophenyl)-3,3,3-trifluoropropyl)-1-methyl-2-nitroguanidine (**3cd**). White solid, m.p. 118.9–120.1 °C, yield 70% (220.9 mg); ^1^H NMR (400 MHz, DMSO-*d*_6_) δ 8.36 (s, 2H), 7.88 (d, *J* = 8.0 Hz, 2H), 7.65 (d, *J* = 8.0 Hz, 2H), 4.41–4.30 (m, 1H), 4.10–4.00 (m, 2H), 2.79 (s, 3H); ^13^C NMR (100 MHz, DMSO-*d*_6_) δ 159.1, 138.3, 133.0, 130.9, 126.4 (q, ^1^*J*_CF_ = 279.3 Hz), 118.8, 112.1, 48.5, 47.3 (q, ^2^*J*_CF_ = 25.9 Hz), 36.7; ^19^F NMR (564 MHz, DMSO-*d*_6_) δ −66.7 (d, *J* = 9.6 Hz, 3F); HRMS (EI): calcd for C_12_H_12_F_3_N_5_O_2_ [M]^+^: 315.0943, found: 315.0946.

(*E*)-1-(2-(6-Chloropyridin-3-yl)-3,3,3-trifluoropropyl)-1-methyl-2-nitroguanidine (**3td**). White solid, m.p. 113.5–114.9 °C, yield 69% (224.3 mg); ^1^H NMR (400 MHz, DMSO-*d*_6_) δ 8.49 (d, *J* = 2.0 Hz, 1H), 8.37 (s, 2H), 7.97 (dd, *J*_1_ = 8.4 Hz, *J*_2_ = 2.4 Hz, 1H), 7.59 (d, *J* = 8.4 Hz, 1H), 4.39–4.33 (m, 1H), 4.12–4.00 (m, 2H), 2.83 (s, 3H); ^13^C NMR (150 MHz, DMSO-*d*_6_) δ 158.6, 150.8, 150.7, 140.2, 127.8, 125.8 (q, ^1^*J*_CF_ = 279.0 Hz), 124.3, 47.6, 43.9 (q, ^2^*J*_CF_ = 26.7 Hz), 36.2; ^19^F NMR (564 MHz, DMSO-*d*_6_) δ −62.4 (d, *J* = 9.6 Hz, 3F); HRMS (EI): calcd for C_10_H_11_ClF_3_N_5_O_2_ [M]^+^: 325.0553, found: 325.0550.

### 3.3. General Procedure for the Synthesis of the Target Compounds ***4aa**–**xc***

To a glass tube charged with a stirring bar were added NaH (3.0 mmol, 3.0 equiv.), α-(trifluoromethyl)styrenes (**1a**, **1f**–**h**, **1u**–**x**, 1.0 mmol), 2-nitroimino-imidazolidine **2a** (1.0 mmol, 1.0 equiv.) or 2-cyanoimino-thiazolidine **2c** (1.0 mmol, 1.0 equiv.), and CH_3_CN (3 mL). The reaction vial was sealed with a rubber septum and then the reaction mixture was stirred at 60 °C in an oil bath for 12 h (monitored by TLC). After the completion of reaction, the reaction mixture was quenched with a saturated aqueous solution of NH_4_Cl (15 mL) and extracted with ethyl acetate (3 × 15 mL). The organic layer was separated and dried over Na_2_SO_4_, filtered and concentrated in vacuo. The residue was purified by column chromatography on silica gel using *n*-hexane/ethyl acetate (5/1–1/1) as eluent to afford the target compounds **4aa**–**xc**.

(*E*)-*N*-(1-(2-([1,1′-Biphenyl]-4-yl)-3,3-difluoroallyl)imidazolidin-2-ylidene)nitramide (**4aa**). White solid, m.p. 168.6–169.0 °C, yield 62% (222.1 mg); ^1^H NMR (400 MHz, CDCl_3_) δ 8.04 (s, 1H), 7.60 (t, *J* = 8.0 Hz, 4H), 7.49–7.42 (m, 4H), 7.36 (t, *J* = 7.2 Hz, 1H), 4.49 (t, *J* = 2.0 Hz, 2H), 3.69–3.65 (m, 2H), 3.52–3.48 (m, 2H); ^13^C NMR (100 MHz, CDCl_3_) δ 161.3, 155.2 (dd, ^1^*J*_CF_ = 294.3, 290.5 Hz), 140.9, 140.1, 129.5, 129.1 (t, ^3^*J*_CF_ = 3.2 Hz), 128.9, 128.5 (t, ^4^*J*_CF_ = 3.2 Hz), 127.7, 127.4, 127.0, 88.3 (dd, ^2^*J*_CF_ = 17.7, 14.0 Hz), 44.8, 41.5 (d, ^3^*J*_CF_ = 3.0 Hz), 41.3; ^19^F NMR (564 MHz, CDCl_3_) δ −84.6 (d, *J* = 46.8 Hz, 1F), −86.6 (d, *J* = 47.4 Hz, 1F); HRMS (ESI): calcd for C_18_H_16_F_2_N_4_O_2_Na [M + Na]^+^: 381.1139, found: 381.1135.

(*E*)-*N*-(1-(3,3-Difluoro-2-(p-tolyl)allyl)imidazolidin-2-ylidene)nitramide (**4fa**). White solid, m.p. 109.9–110.6 °C, yield 65% (193.0 mg); ^1^H NMR (400 MHz, CDCl_3_) δ 8.02 (s, 1H), 7.27 (d, *J* = 7.2 Hz, 2H), 7.17 (d, *J* = 8.0 Hz, 2H), 4.42 (t, *J* = 2.0 Hz, 2H), 3.66–3.62 (m, 2H), 3.48–3.44 (m, 2H), 2.33 (s, 3H); ^13^C NMR (100 MHz, CDCl_3_) δ 161.3, 155.0 (dd, ^1^*J*_CF_ = 293.5, 289.8 Hz), 138.2, 129.5, 127.9 (t, ^3^*J*_CF_ = 3.2 Hz), 127.2 (t, ^4^*J*_CF_ = 3.4 Hz), 88.4 (dd, ^2^*J*_CF_ = 17.2, 14.7 Hz), 44.7, 41.5 (d, ^3^*J*_CF_ = 2.3 Hz), 41.3, 21.2; ^19^F NMR (564 MHz, CDCl_3_) δ −85.8 (d, *J* = 33.3 Hz, 1F), −87.8 (d, *J* = 33.8 Hz, 1F); HRMS (ESI): calcd for C_13_H_14_F_2_N_4_O_2_Na [M + Na]^+^: 319.0983, found: 319.0981.

(*E*)-*N*-(1-(3,3-Difluoro-2-(4-methoxyphenyl)allyl)imidazolidin-2-ylidene)nitramide (**4ua**). White solid, m.p. 128.8–130.2 °C, yield 78% (243.9 mg); ^1^H NMR (400 MHz, acetone-*d*_6_) δ 8.47 (s, 1H), 7.43 (d, *J* = 8.4 Hz, 2H), 6.68 (d, *J* = 8.4 Hz, 2H), 4.41 (t, *J* = 2.0 Hz, 2H), 3.83 (s, 3H), 3.74–3.70 (m, 2H), 3.62–3.57 (m, 2H); ^13^C NMR (100 MHz, acetone-*d*_6_) δ 161.3, 159.5, 154.7 (dd, ^1^*J*_CF_ = 288.8, 288.2 Hz), 129.7 (t, ^3^*J*_CF_ = 3.0 Hz), 122.9 (t, ^4^*J*_CF_ = 3.2 Hz), 113.9, 88.9 (dd, ^2^*J*_CF_ = 17.9, 14.7 Hz), 54.7, 44.8, 41.6 (d, ^3^*J*_CF_ = 3.0 Hz), 41.4; ^19^F NMR (564 MHz, acetone-*d*_6_) δ −89.9 (d, *J* = 40.0 Hz, 1F), −91.4 (d, *J* = 41.2 Hz, 1F); HRMS (ESI): calcd for C_13_H_14_F_2_N_4_O_3_Na [M + Na]^+^: 335.0932, found: 335.0935.

(*E*)-*N*-(1-(2-(3,4-Dimethoxyphenyl)-3,3-difluoroallyl)imidazolidin-2-ylidene)nitramide (**4va**). White solid, m.p. 123.9–124.6 °C, yield 77% (263.5 mg); ^1^H NMR (400 MHz, CDCl_3_) δ 7.98 (s, 1H), 6.96–6.94 (m, 2H), 6.83 (d, *J* = 8.4 Hz, 1H), 4.42 (t, *J* = 2.2 Hz, 2H), 3.85 (s, 3H), 3.84 (s, 3H), 3.66–3.61 (m, 2H), 3.48–3.43 (m, 2H); ^13^C NMR (100 MHz, CDCl_3_) δ 161.1, 154.9 (dd, ^1^*J*_CF_ = 293.4, 289.1 Hz), 149.0, 132.0, 128.6, 122.4 (t, ^3^*J*_CF_ = 3.3 Hz), 120.8 (dd, ^4^*J*_CF_ = 5.0, 2.8 Hz), 111.2, 111.0 (t, ^4^*J*_CF_ = 3.1 Hz), 88.3 (dd, ^2^*J*_CF_ = 17.6, 13.7 Hz), 56.0, 55.9, 44.7, 41.3 (d, ^3^*J*_CF_ = 4.5 Hz); ^19^F NMR (564 MHz, CDCl_3_) δ −85.7 (d, *J* = 35.5 Hz, 1F), −88.1 (d, *J* = 35.5 Hz, 1F); HRMS (ESI): calcd for C_14_H_16_F_2_N_4_O_4_Na [M + Na]^+^: 365.1038, found: 365.1035.

(*E*)-*N*-(1-(2-(Dibenzo[*b*,*d*]thiophen-4-yl)-3,3-difluoroallyl)imidazolidin-2-ylidene)nitramide (**4wa**). White solid, m.p. 181.9–183.0 °C, yield 66% (256.7 mg); ^1^H NMR (400 MHz, CDCl_3_) δ 8.14 (s, 1H), 8.13 (d, *J* = 7.6 Hz, 1H), 7.92–7.84 (m, 2H), 7.52–7.41 (m, 4H), 4.50 (s, 2H), 3.54 (s, 4H); ^13^C NMR (100 MHz, CDCl_3_) δ 156.5, 149.6 (t, ^1^*J*_CF_ = 293.4 Hz), 135.0, 133.9, 131.4, 131.0, 123.0, 122.5, 120.4, 120.1, 118.0, 117.2, 83.0 (dd, ^2^*J*_CF_ = 19.5, 17.9 Hz), 40.7, 37.8 (d, ^3^*J*_CF_ = 4.5 Hz), 36.6; ^19^F NMR (564 MHz, CDCl_3_) δ −80.3 (d, *J* = 25.9 Hz, 1F), −86.8 (d, *J* = 25.9 Hz, 1F); HRMS (ESI): calcd for C_18_H_14_F_2_N_4_O_2_SNa [M + Na]^+^: 411.0704, found: 411.0705.

(*Z*)-*N*-(3-(3,3-Difluoro-2-(4-(methylthio)phenyl)allyl)thiazolidin-2-ylidene)cyanamide (**4gc**). White solid, m.p. 133.1–134.7 °C, yield 58% (189.1 mg); ^1^H NMR (400 MHz, CDCl_3_) δ 7.25 (s, 4H), 4.48 (t, *J* = 2.0 Hz, 2H), 3.69 (t, *J* = 7.6 Hz, 2H), 3.21 (t, *J* = 7.6 Hz, 2H), 2.50 (s, 3H); ^13^C NMR (100 MHz, CDCl_3_) δ 174.7, 155.0 (dd, ^1^*J*_CF_ = 294.2, 290.9 Hz), 139.5, 128.3, 126.4, 126.1, 117.0, 88.2 (dd, ^2^*J*_CF_ = 17.8, 15.2 Hz), 51.7, 43.1 (d, ^3^*J*_CF_ = 2.6 Hz), 27.2, 15.3; ^19^F NMR (564 MHz, CDCl_3_) δ −84.9 (d, *J* = 30.4 Hz, 1F), −86.4 (d, *J* = 31.0 Hz, 1F); HRMS (EI): calcd for C_14_H_13_F_2_N_3_S_2_ [M]^+^: 325.0519, found: 325.0522.

(*Z*)-*N*-(3-(2-(4-(*tert*-Butyl)phenyl)-3,3-difluoroallyl)thiazolidin-2-ylidene)cyanamide (**4hc**). White solid, m.p. 133.5–135.0 °C, yield 55% (184.7 mg); ^1^H NMR (400 MHz, CDCl_3_) δ 7.34 (d, *J* = 7.6 Hz, 2H), 7.21 (d, *J* = 7.2 Hz, 2H), 4.43 (t, *J* = 2.0 Hz, 2H), 3.67–3.63 (m, 2H), 3.17–3.13 (m, 2H), 1.26 (s, 9H); ^13^C NMR (100 MHz, CDCl_3_) δ 174.6, 155.1 (dd, ^1^*J*_CF_ = 293.7, 289.5 Hz), 151.6, 128.5, 127.6 (t, ^3^*J*_CF_ = 2.8 Hz), 126.8 (t, ^4^*J*_CF_ = 3.0 Hz), 126.1, 125.9, 117.1, 88.3 (dd, ^2^*J*_CF_ = 17.2, 15.0 Hz), 51.7, 43.3 (d, ^3^*J*_CF_ = 2.4 Hz), 34.7, 31.2, 27.2; ^19^F NMR (564 MHz, CDCl_3_) δ −85.8 (d, *J* = 33.3 Hz, 1F), −87.8 (d, *J* = 34.4 Hz, 1F); HRMS (EI): calcd for C_17_H_19_F_2_N_3_S [M]^+^: 335.1268, found: 335.1263.

(*Z*)-*N*-(3-(2-(Benzo[*d*][1,3]dioxol-5-yl)-3,3-difluoroallyl)thiazolidin-2-ylidene)cyanamide (**4xc**). White solid, m.p. 123.5–124.9 °C, yield 68% (219.9 mg); ^1^H NMR (400 MHz, CDCl_3_) δ 6.82–6.76 (m, 3H), 5.99 (s, 2H), 4.43 (t, *J* = 2.0 Hz, 2H), 3.71 (t, *J* = 7.6 Hz, 2H), 3.24 (t, *J* = 7.6 Hz, 2H); ^13^C NMR (100 MHz, CDCl_3_) δ 174.6, 154.9 (dd, ^1^*J*_CF_ = 292.5, 290.3 Hz), 148.1, 147.7, 123.4 (t, ^3^*J*_CF_ = 3.0 Hz), 121.9 (t, ^4^*J*_CF_ = 2.9 Hz), 117.1, 108.7, 108.5 (t, ^4^*J*_CF_ = 3.3 Hz), 101.5, 88.3 (dd, ^2^*J*_CF_ = 18.1, 16.0 Hz), 51.8, 43.6 (d, ^3^*J*_CF_ = 2.8 Hz), 27.2; ^19^F NMR (564 MHz, CDCl_3_) δ −85.8 (d, *J* = 35.5 Hz, 1F), −88.1 (d, *J* = 35.0 Hz, 1F); HRMS (EI): calcd for C_14_H_11_F_2_N_3_O_2_S [M]^+^: 323.0540, found: 323.0543.

### 3.4. Procedure for the Synthesis of the Target Compound ***6***

To a glass tube charged with a stirring bar were added DBN (1.5 mmol, 1.5 equiv.), 2-bromo-3,3,3-trifluoroprop-1-ene **5** (1.0 mmol, 1.0 equiv.), 2-nitroimino-imidazolidine **2a** (1.0 mmol, 1.0 equiv.), and CH_3_CN (3 mL). The reaction was stirred for 6 h under room temperature (monitored by TLC). After the completion of reaction, the reaction mixture was quenched with a saturated aqueous solution of NH_4_Cl (15 mL) and extracted with ethyl acetate (3 × 15 mL). The organic layer was separated and dried over Na_2_SO_4_, filtered and concentrated in vacuo. The residue was purified by column chromatography on silica gel using *n*-hexane/ethyl acetate (3/1) as eluent to afford the target compound **6**.

(*E*)-*N*-(1-(2-Bromo-3,3,3-trifluoropropyl)imidazolidin-2-ylidene)nitramide (**6**). White solid, m.p. 114.5–115.3 °C, yield 63% (191.3 mg); ^1^H NMR (400 MHz, acetone-*d*_6_) δ 8.64 (s, 1H), 5.05–4.99 (m, 1H), 4.02–3.97 (m, 1H), 3.90–3.83 (m, 5H); ^13^C NMR (100 MHz, acetone-*d*_6_) δ 161.5, 123.9 (q, ^1^*J*_CF_ = 275.8 Hz), 46.4, 45.8 (d, ^3^*J*_CF_ = 1.8 Hz), 43.7 (q, ^2^*J*_CF_ = 30.8 Hz), 41.9; ^19^F NMR (564 MHz, acetone-*d*_6_) δ −71.4 (d, *J* = 7.9 Hz, 3F); HRMS (EI): calcd for C_6_H_8_BrF_3_N_4_O_2_ [M]^+^: 303.9783, found: 303.9785.

## 4. Conclusions

In summary, two novel series of fluorinated analogues of neonicotinoids were synthesized. The hydroamination and defluorinative amination of α-(trifluoromethyl)styrenes can be controlled by the subtle choice of reaction conditions and nitrogen nucleophiles. The hydroamination of α-(trifluoromethyl)styrenes with 2-nitroimino-imidazolidine (**2a**), 2-(nitromethylene)imidazolidine (**2b**), 2-cyanoimino-thiazolidine (**2c**), and (*E*)-1-methyl-2-nitroguanidine (**2d**) proceeded efficiently in the presence of DBN and was completed at room temperature within 0.5–6 h, affording a number of structurally diverse β-trifluoromethyl-β-arylethyl analogues of neonicotinoids in moderate to good yields. The γ,γ-difluoro-β-arylallyl analogues of neonicotinoids were also successfully synthesized via defluorination of α-(trifluoromethyl)styrenes with 2-nitroimino-imidazolidine (**2a**) and 2-cyanoimino-thiazolidine (**2c**) using NaH as base at an elevated temperature together with a prolonged reaction time of 12 h. The preliminary insecticidal activity tests indicated that only compounds **3ta**, **3tc**, and **3ca** displayed moderate insecticidal activity against cowpea aphids (*Aphis craccivora*). The mortalities of **3ta**, **3tc**, and **3ca** were 55%, 42%, and 38% at 250 mg/L, respectively. These preliminary results further demonstrated that flexible linkage such as the methylene group (–CH_2_–) in imidacloprid, plays a key role in the insecticidal activity. Increasing the length of carbon chain might be unfavorable for retaining insecticidal activity. The insecticidal evaluation of the target compounds against other insects such as armyworm and carmine spider, is underway in our laboratory.

## Figures and Tables

**Table 1:** Optimization of reaction conditions *^a^*.

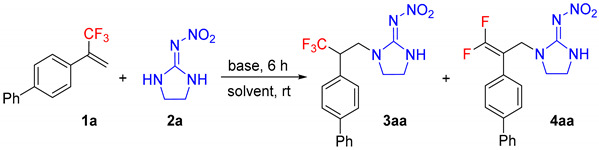				
Entry	Base (equiv.)	Solvent	Temp (°C)	3aa/4aa (%) *^b^*
1	LiHMDS (3.0)	CH_3_CN	25	0/0
2	KOH (3.0)	CH_3_CN	25	2/9
3	Cs_2_CO_3_ (3.0)	CH_3_CN	25	15/21
4	KO*t*Bu (3.0)	CH_3_CN	25	27/23
5	Et_3_N (3.0)	CH_3_CN	25	0/0
6	TMEDA (3.0) *^c^*	CH_3_CN	25	0/0
7	DIPEA (3.0) *^c^*	CH_3_CN	25	0/0
8	DMAP (3.0) *^c^*	CH_3_CN	25	0/0
9	DABCO (3.0) *^c^*	CH_3_CN	25	0/0
10	TMG (3.0) *^c^*	CH_3_CN	25	60/0
11	TBD (3.0) *^c^*	CH_3_CN	25	76/0
12	DBU (3.0) *^c^*	CH_3_CN	25	78/0
13	DBN (3.0) *^c^*	CH_3_CN	25	97/0
14	DBN (3.0)	MeOH	25	2/0
15	DBN (3.0)	toluene	25	17/0
16	DBN (3.0)	CH_2_Cl_2_	25	57/0
17	DBN (3.0)	DMSO	25	82/0
18	DBN (3.0)	NMP	25	93/0
19	DBN (3.0)	THF	25	95/0
20	DBN (2.5)	CH_3_CN	25	97/0
21	DBN (2.0)	CH_3_CN	25	82/0
22 *^d^*	NaH (3.0)	CH_3_CN	25	0/30
23 *^d^*	NaH (3.0)	CH_3_CN	40	0/43
24 *^d^*	NaH (3.0)	CH_3_CN	60	0/71

*^a^* Reaction conditions: **1a** (0.1 mmol), **2a** (0.1 mmol), solvent (0.5 mL), rt, 6 h. *^b^* Yields were determined by HPLC analysis based on **1a**. *^c^* TMEDA = *N*,*N*,*N′*,*N′*-Tetramethylethylenediamine, DIPEA = *N*,*N*-Diisopropylethylamine, DMAP = 4-Dimethylaminopyridine, DABCO = 1,4-Diazabicyclo[2.2.2]octane, TMG = Tetramethylguanidine, TBD = 1,5,7-Triazabicyclo[4.4.0]dec-5-ene, DBU = 1,8-Diazabicyclo [5.4.0]undec-7-ene, DBN = 1,5-Diazabicyclo[4.3.0]non-5-ene. *^d^* The reaction time was 12 h.

## Data Availability

The data presented in this study are available in this article.

## Supplementary Materials

The following are available online at https://www.mdpi.com/article/10.3390/molecules28083530/s1, ^1^H, ^13^C, ^19^F NMR and HRMS (EI/ESI) spectra of the target compounds.

## Sample Availability

Samples of the compounds are not available from the authors.
