# Supplementary material for: Controllable Synthesis of Trifluoromethyl- or gem-Difluorovinyl-containing Analogues of Neonicotinoids by the Reaction of α-(Trifluoromethyl)styrenes with 2-Nitroimino-imidazolidine
Source: Molecules. 2023 Apr 17;28(8):3530. doi: 10.3390/molecules28083530 (PMC10145784; doi:10.3390/molecules28083530)

Supporting Information

Controllable Synthesis of Trifluoromethyl- or *gem*-Difluorovinyl-containing Analogues of Neonicotinoids by the Reaction of α-(Trifluoromethyl)styrenes with 2-Nitroimino-imidazolidine

Jingjing He [1], Zhudi Sun [1], Yupian Deng [1], Ying Liu [1], Pai Zheng [1] and Song Cao [1,2,]*

[1]*Shanghai Key Laboratory of Chemical Biology, School of Pharmacy, East China University of Science and Technology (ECUST), Shanghai 200237, China*

[2]*Key Laboratory of Organofluorine Chemistry, Shanghai Institute of Organic Chemistry, Chinese Academy of Sciences, Shanghai 200032, China*

^{1}H, ^{13}C, ^{19}F NMR and HRMS (EI/ESI) spectra of the target compounds

^{1}H NMR spectrum of 3aa (400 MHz, CDCl$_3$)

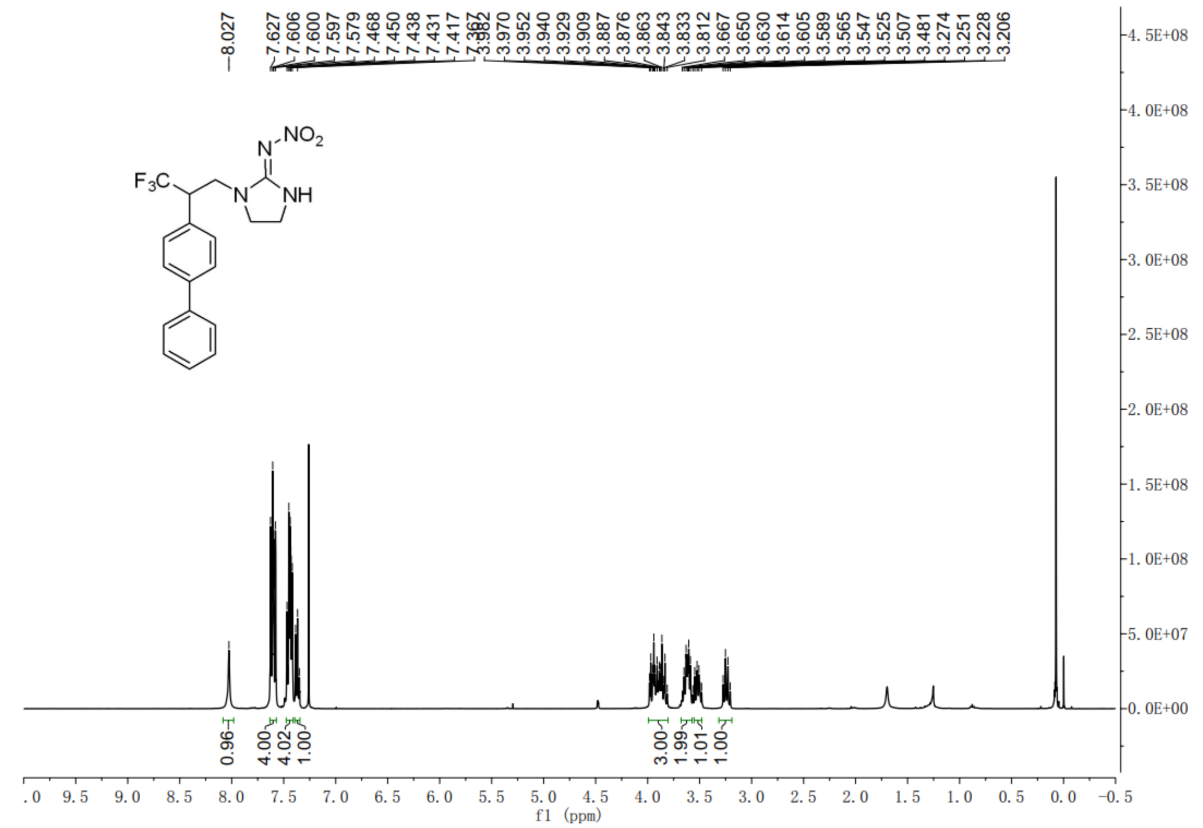

^{13}C NMR spectrum of 3aa (100 MHz, CDCl$_3$)

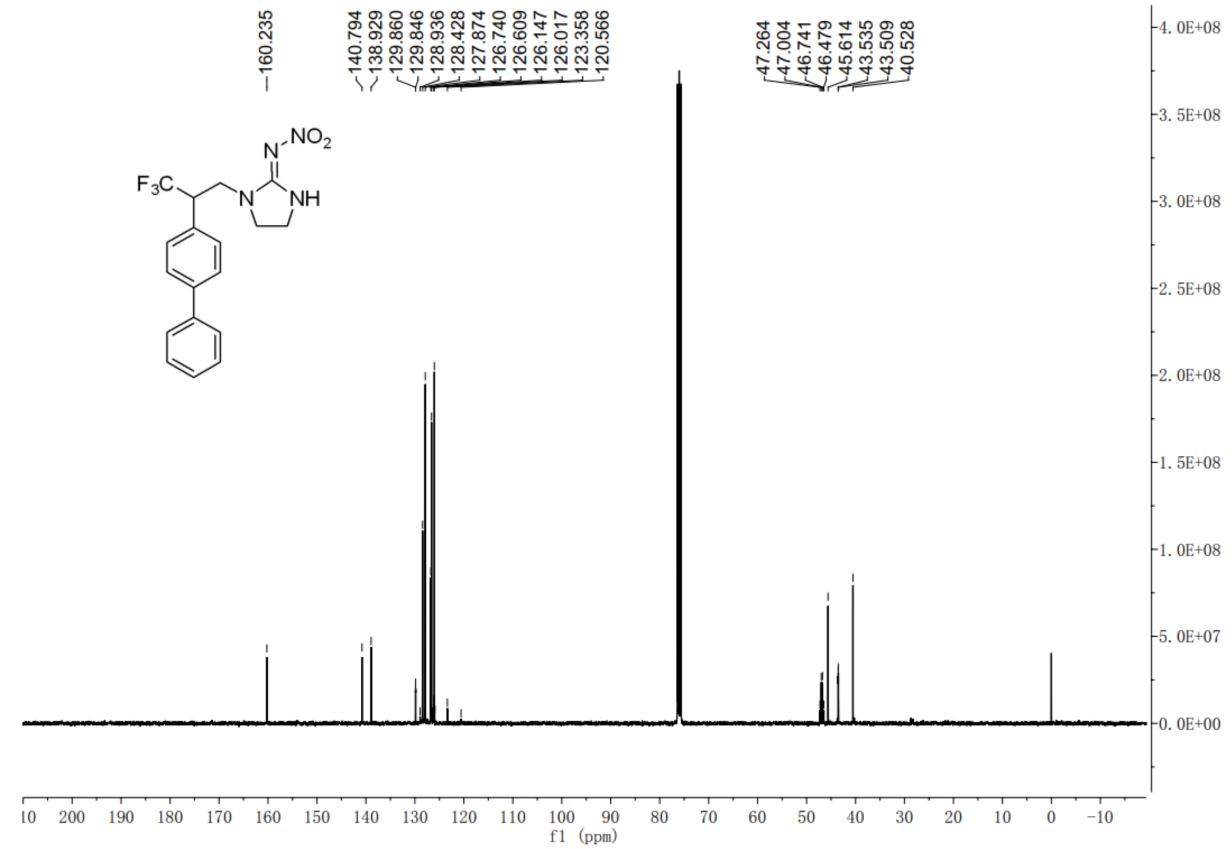

^{19}F NMR spectrum of 3aa (564 MHz, CDCl$_3$)

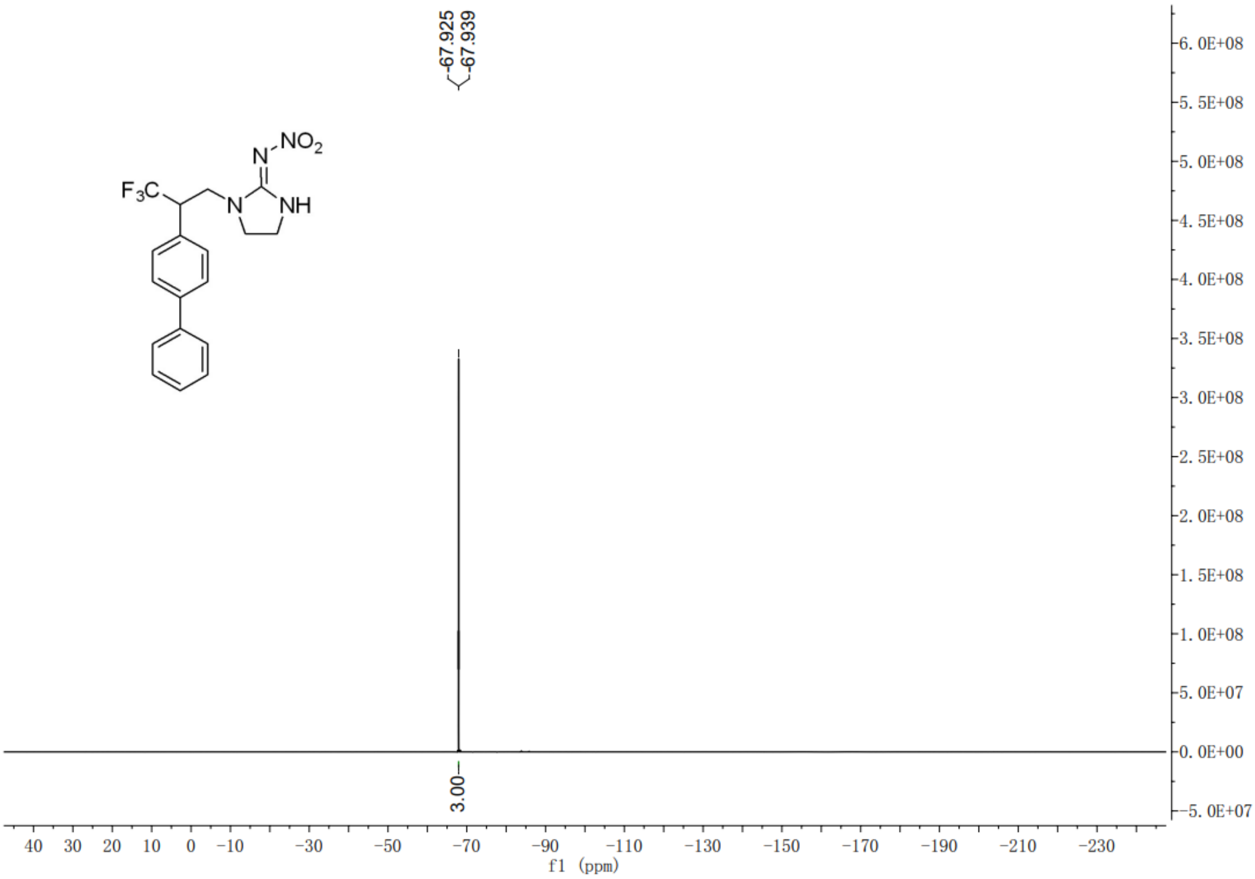

HRMS (EI) spectrum of 3aa

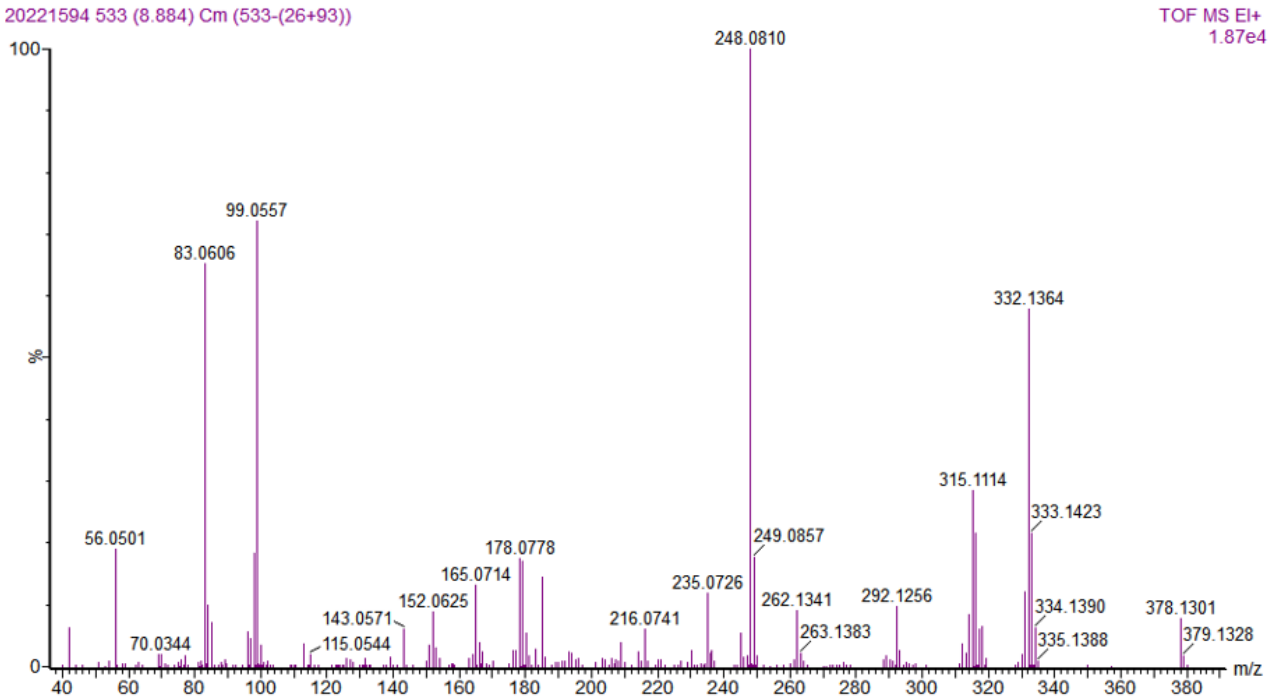

^{1}H NMR spectrum of 3ba (400 MHz, $CDCl_3$)

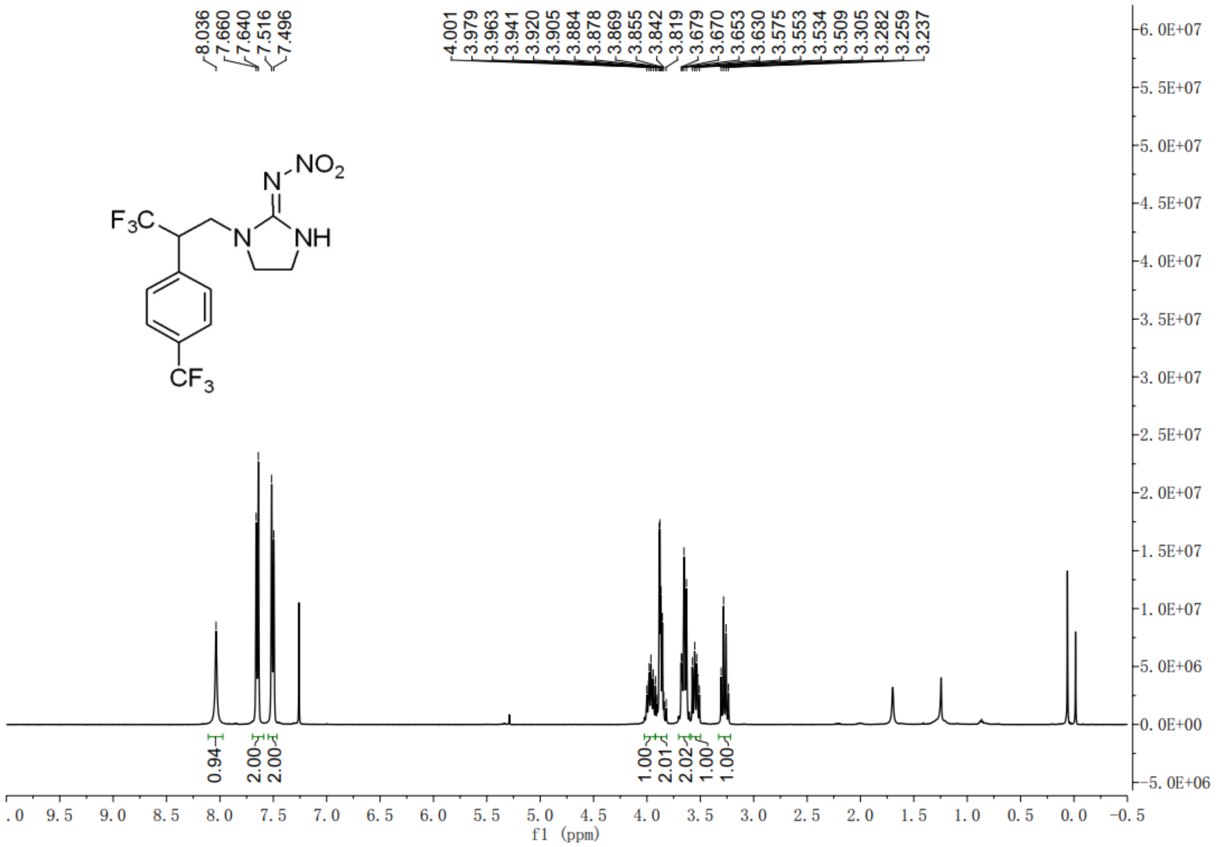

^{13}C NMR spectrum of 3ba (100 MHz, $CDCl_3$)

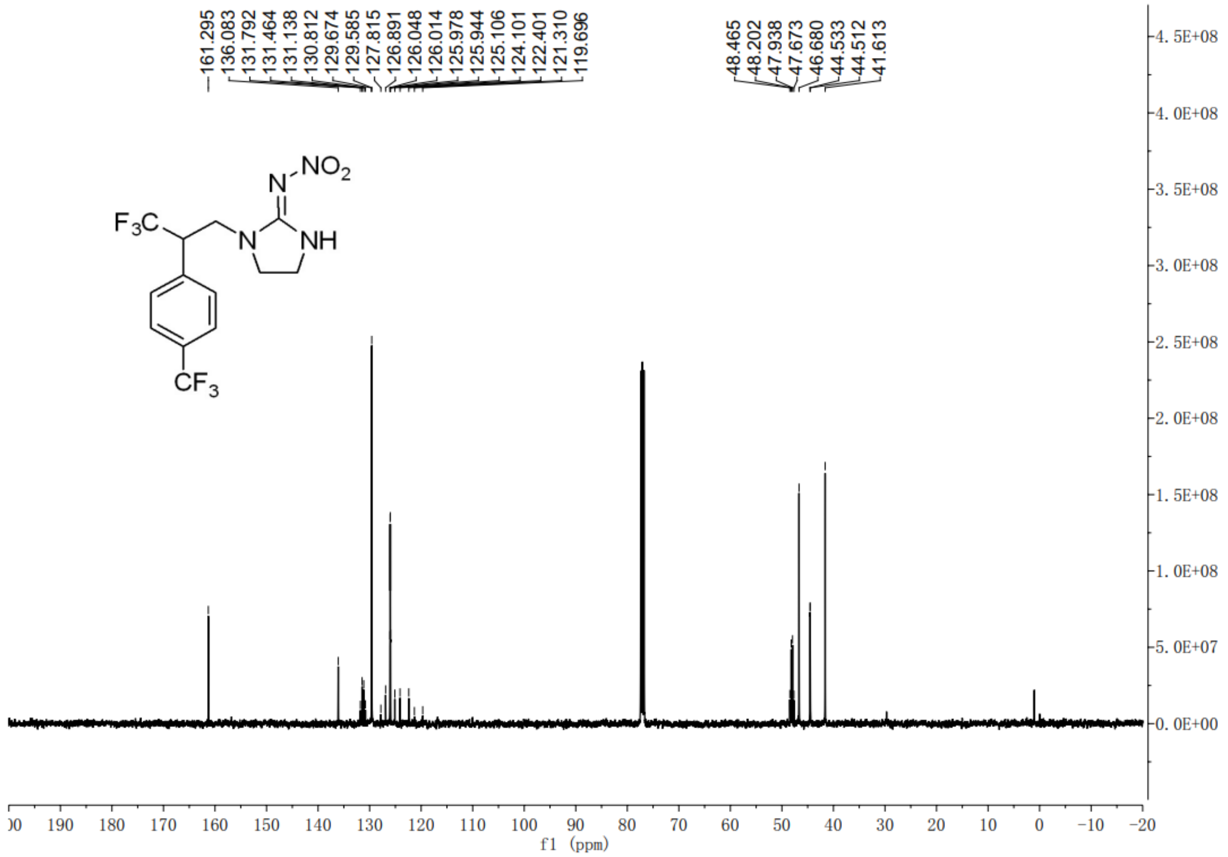

^{19}F NMR spectrum of 3ba (564 MHz, $CDCl_3$)

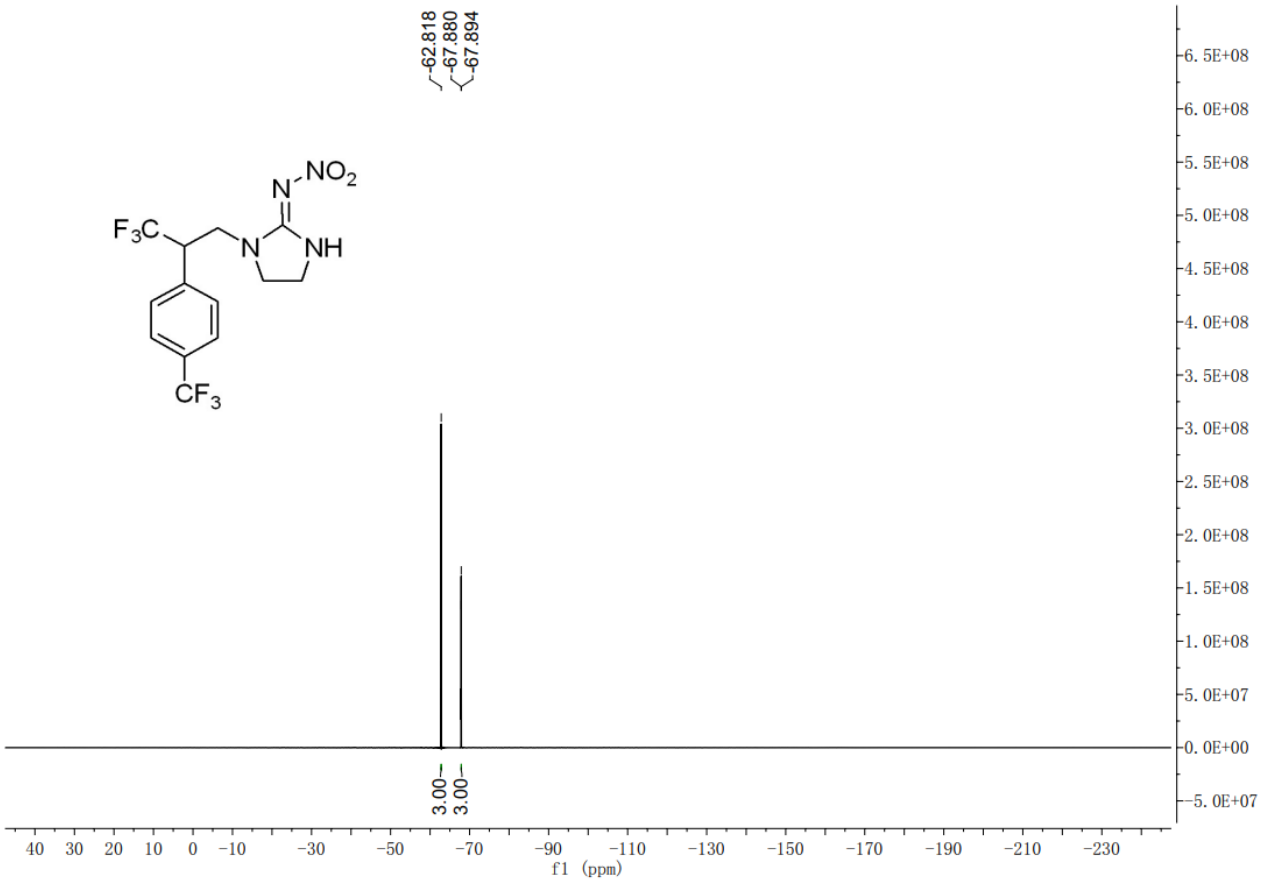

HRMS (EI) spectrum of 3ba

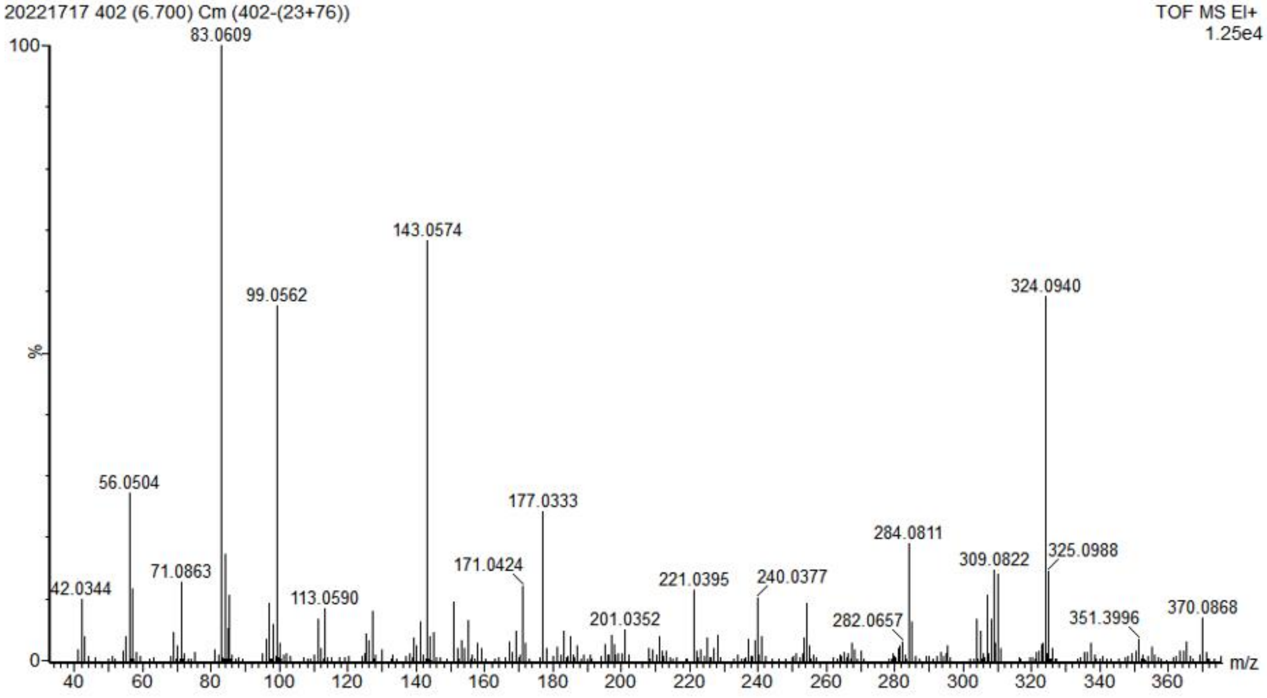

^{1}H NMR spectrum of 3ca (400 MHz, $CDCl_3$)

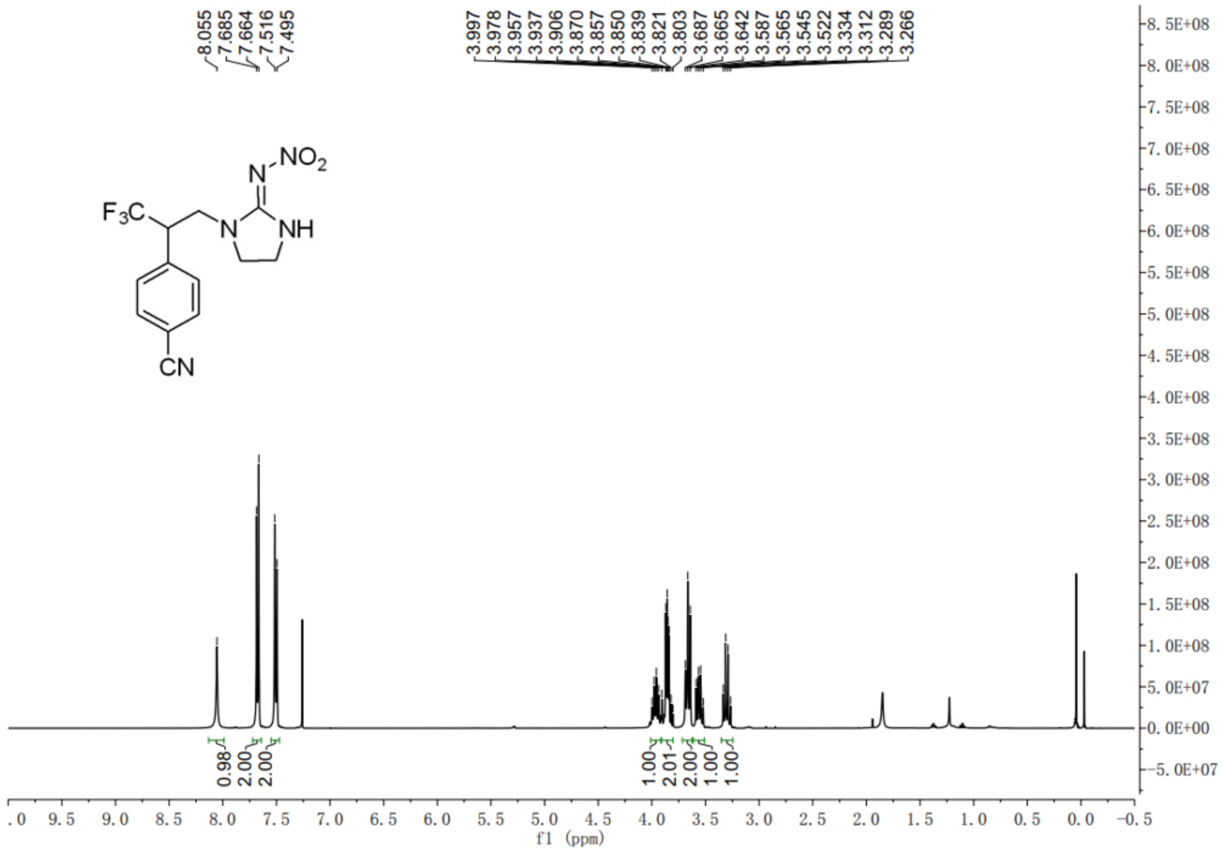

^{13}C NMR spectrum of 3ca (100 MHz, $CDCl_3$)

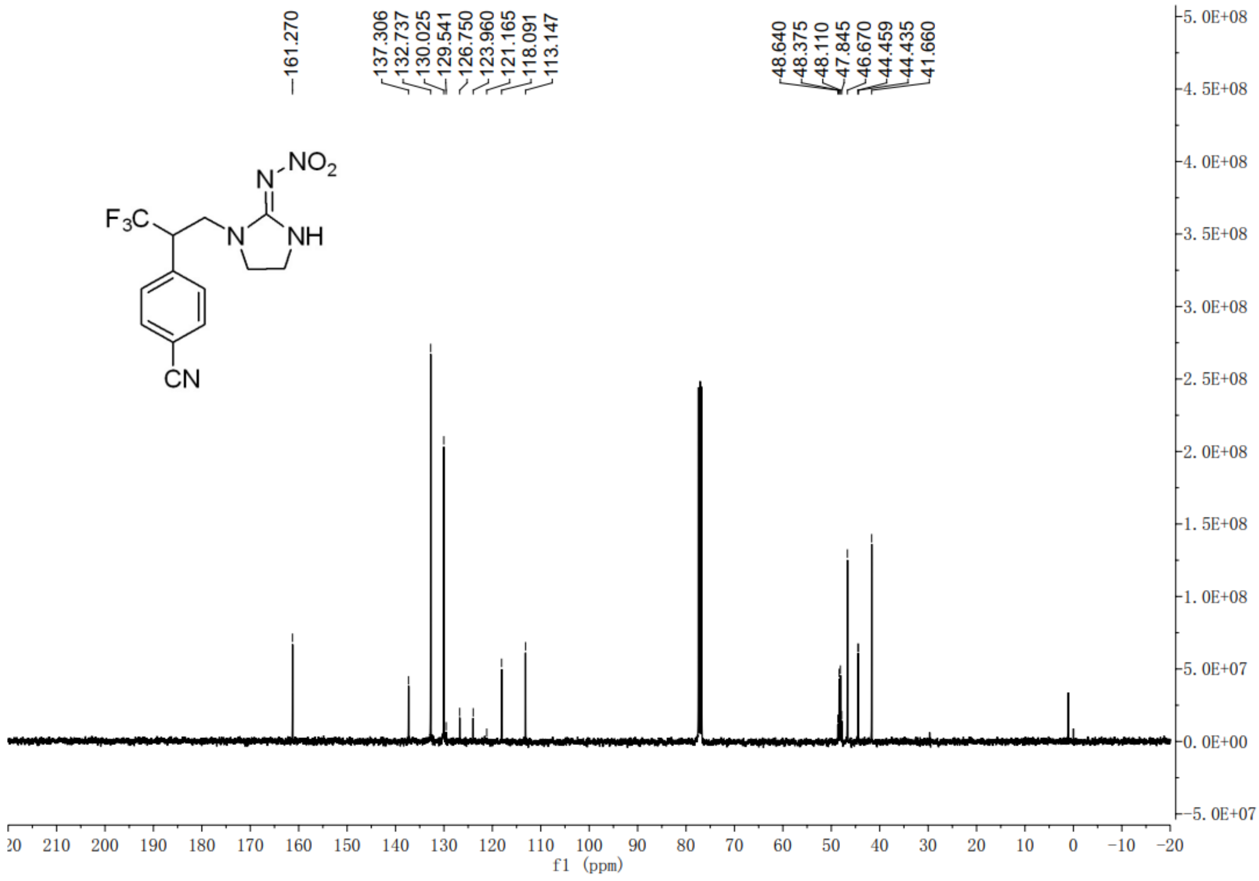

^{19}F NMR spectrum of 3ca (564 MHz, $CDCl_3$)

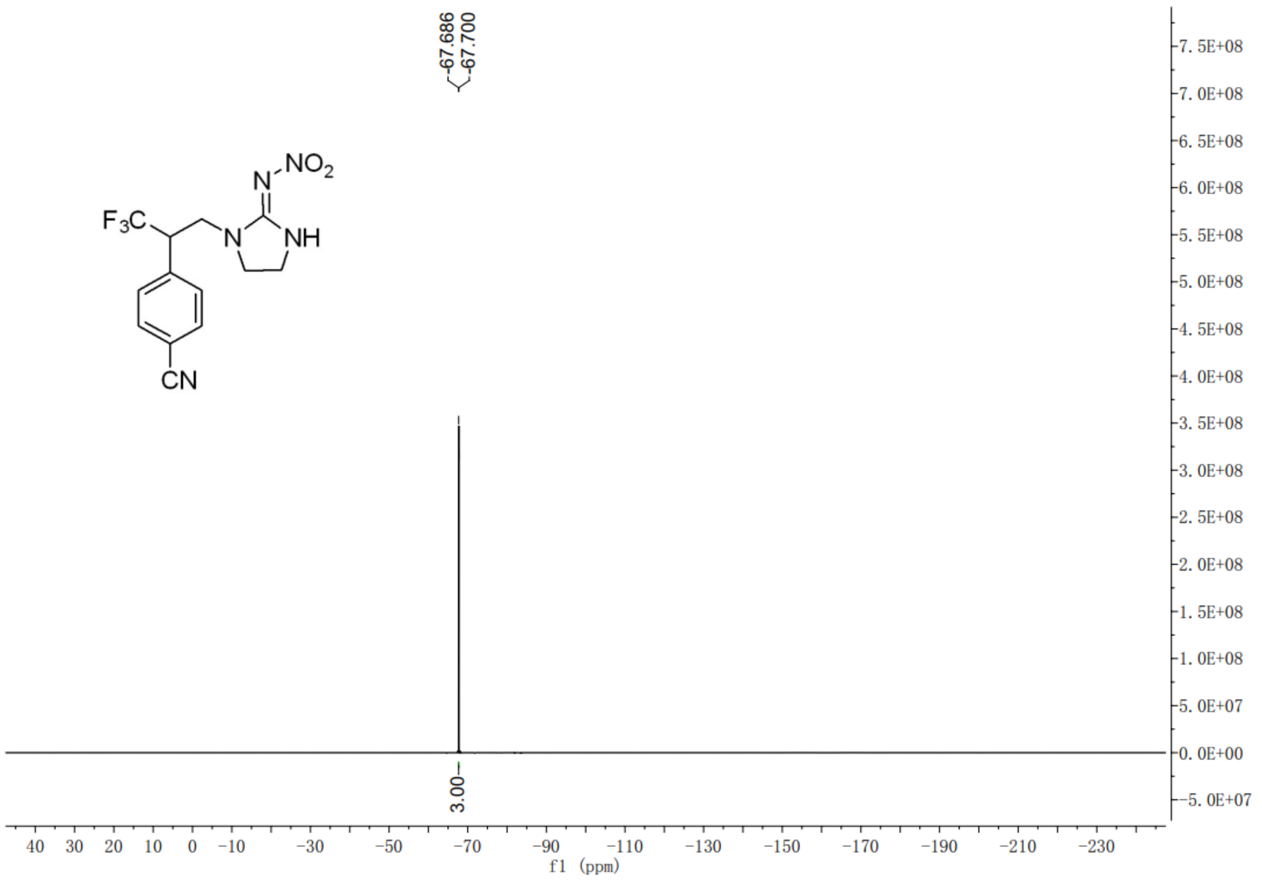

HRMS (EI) spectrum of 3ca

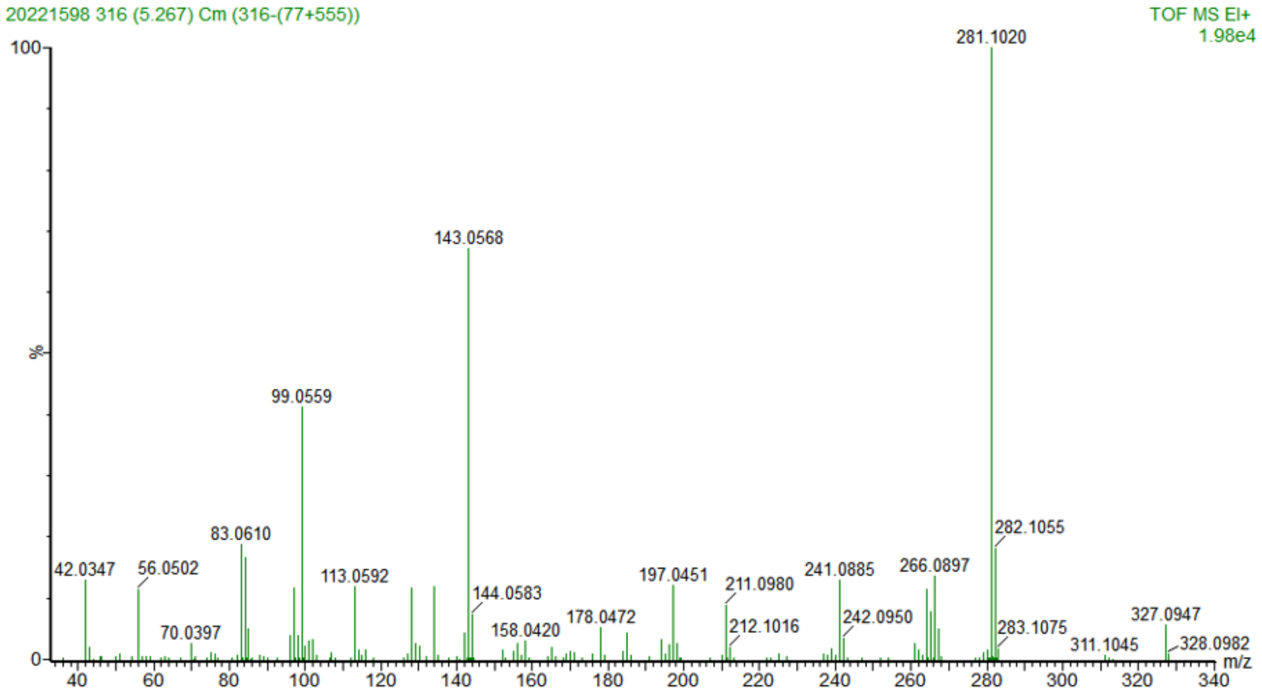

^{1}H NMR spectrum of 3da (400 MHz, $CDCl_3$)

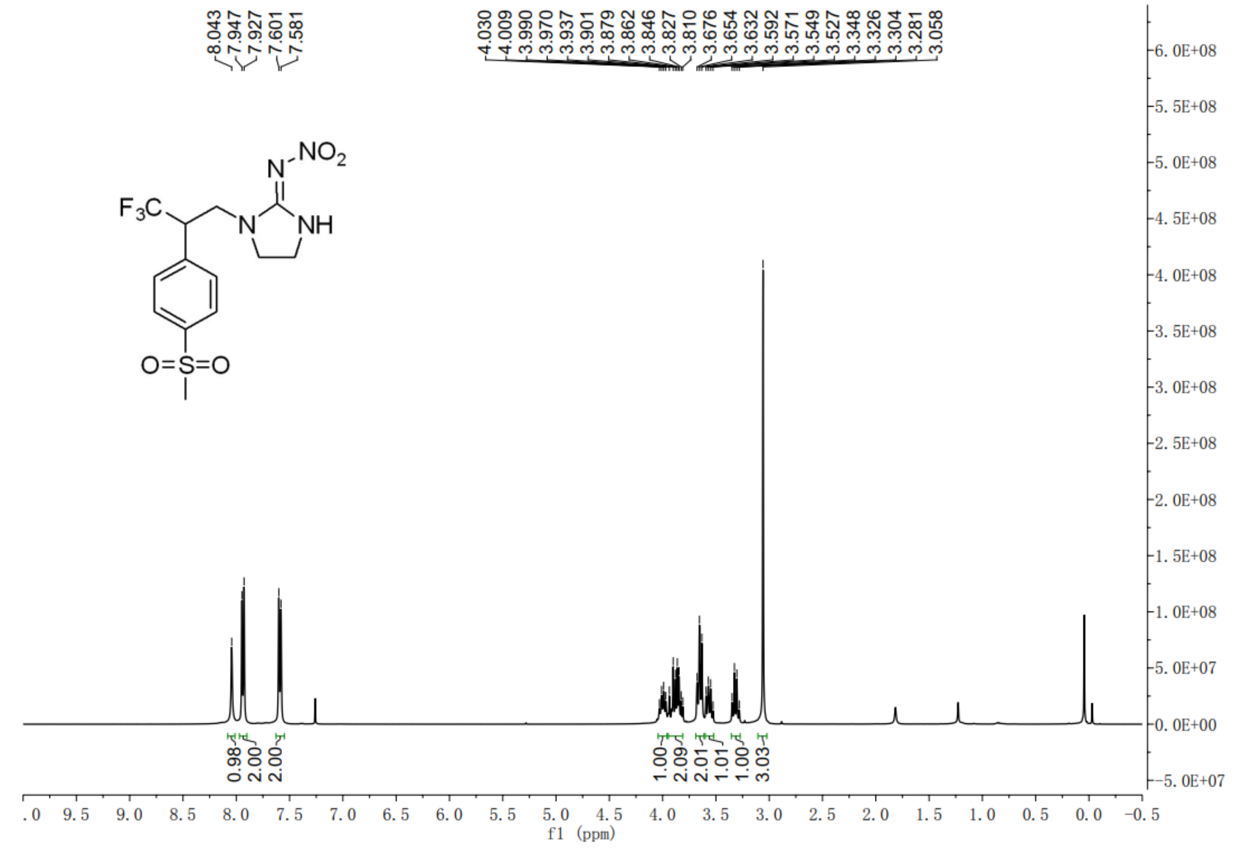

^{13}C NMR spectrum of 3da (100 MHz, $CDCl_3$)

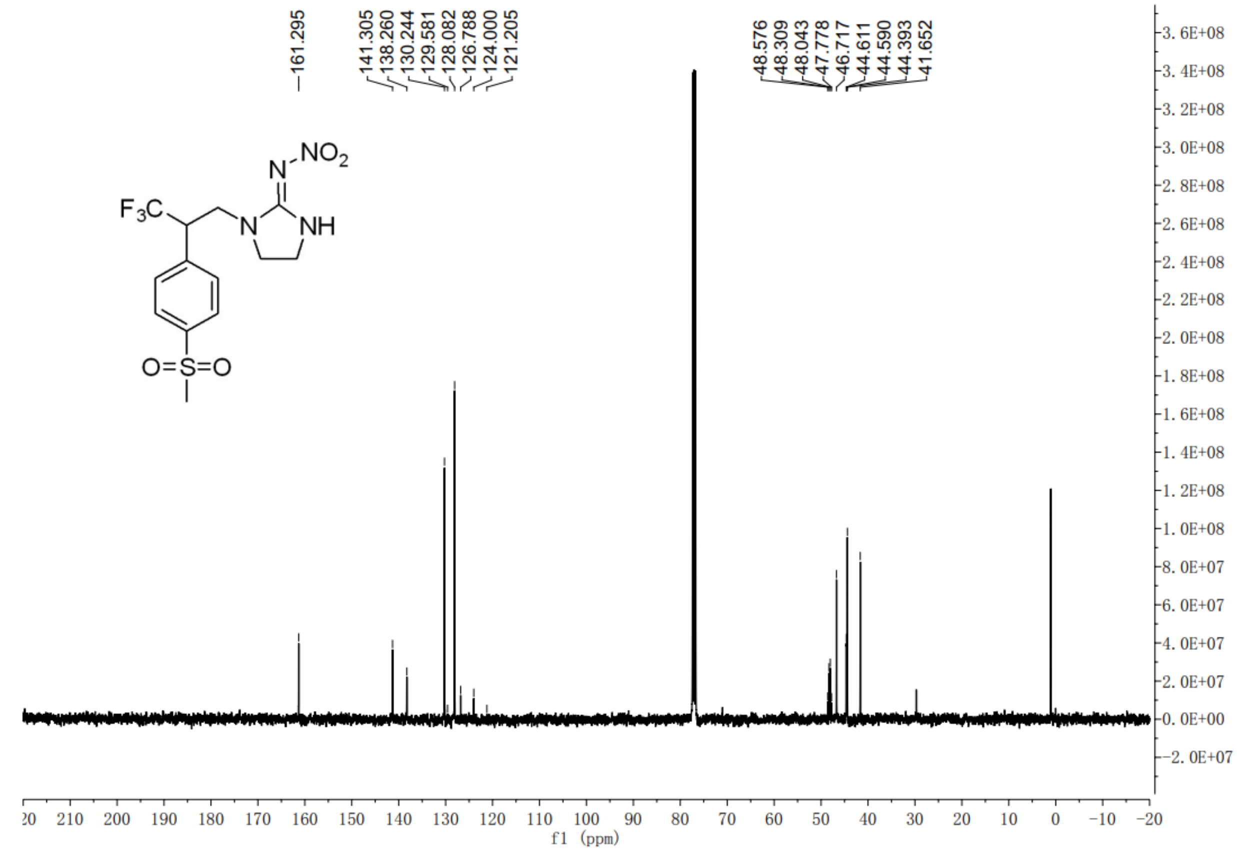

^{19}F NMR spectrum of 3da (564 MHz, CDCl$_3$)

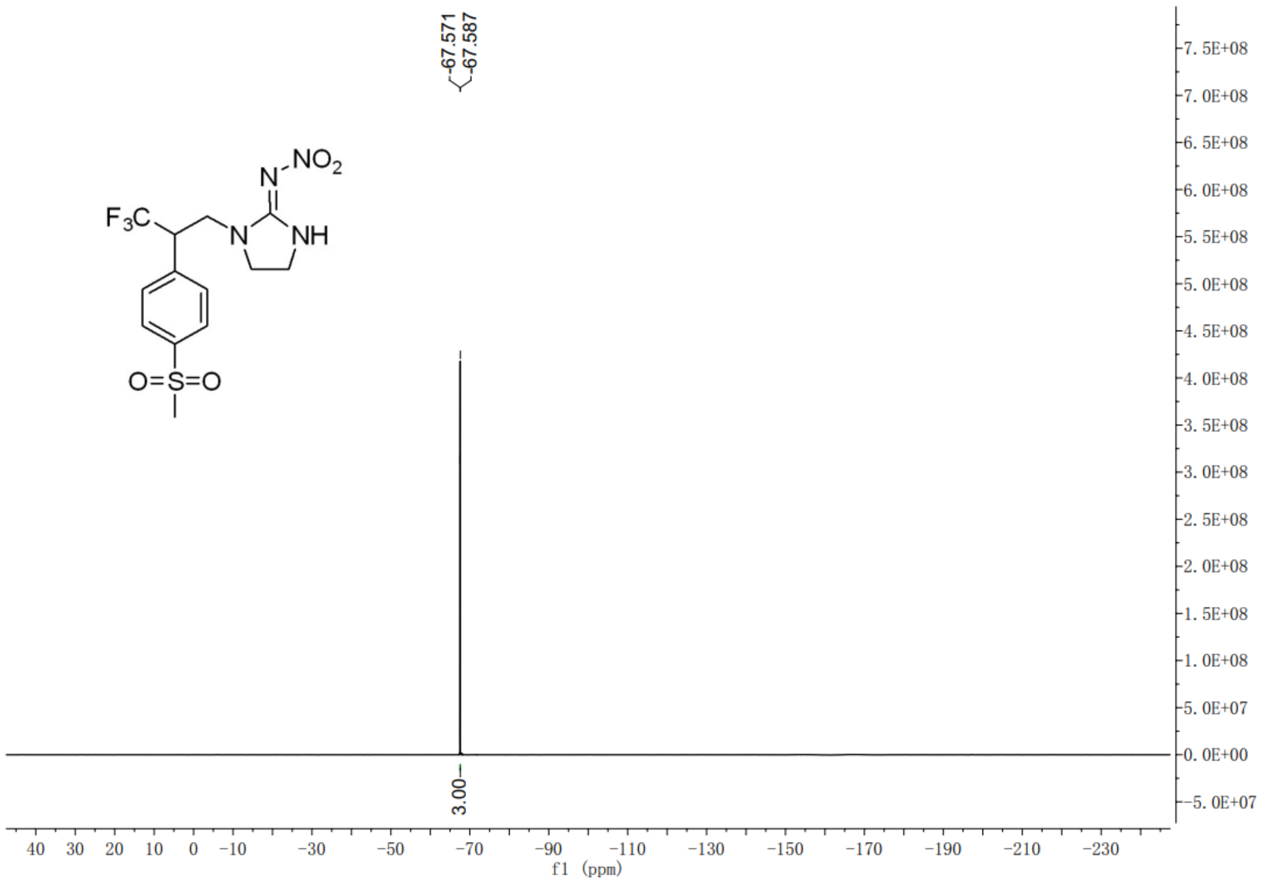

HRMS (EI) spectrum of 3da

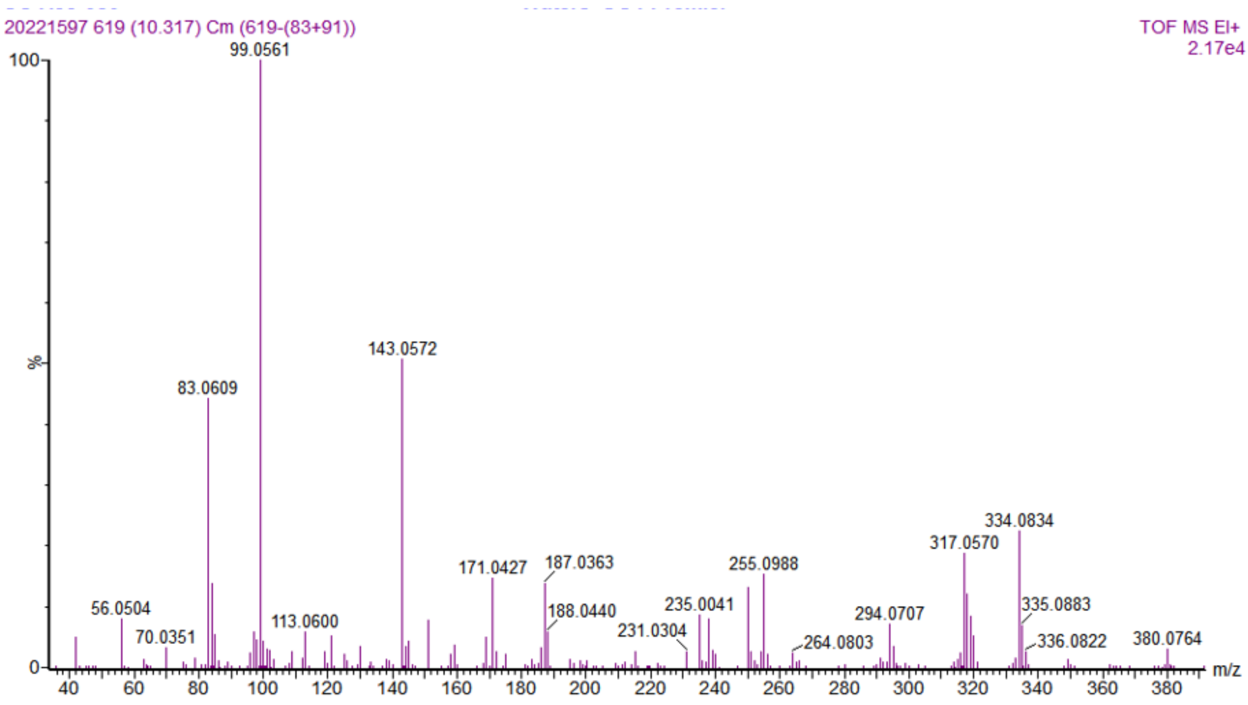

^{1}H NMR spectrum of 3ea (400 MHz, $CDCl_3$)

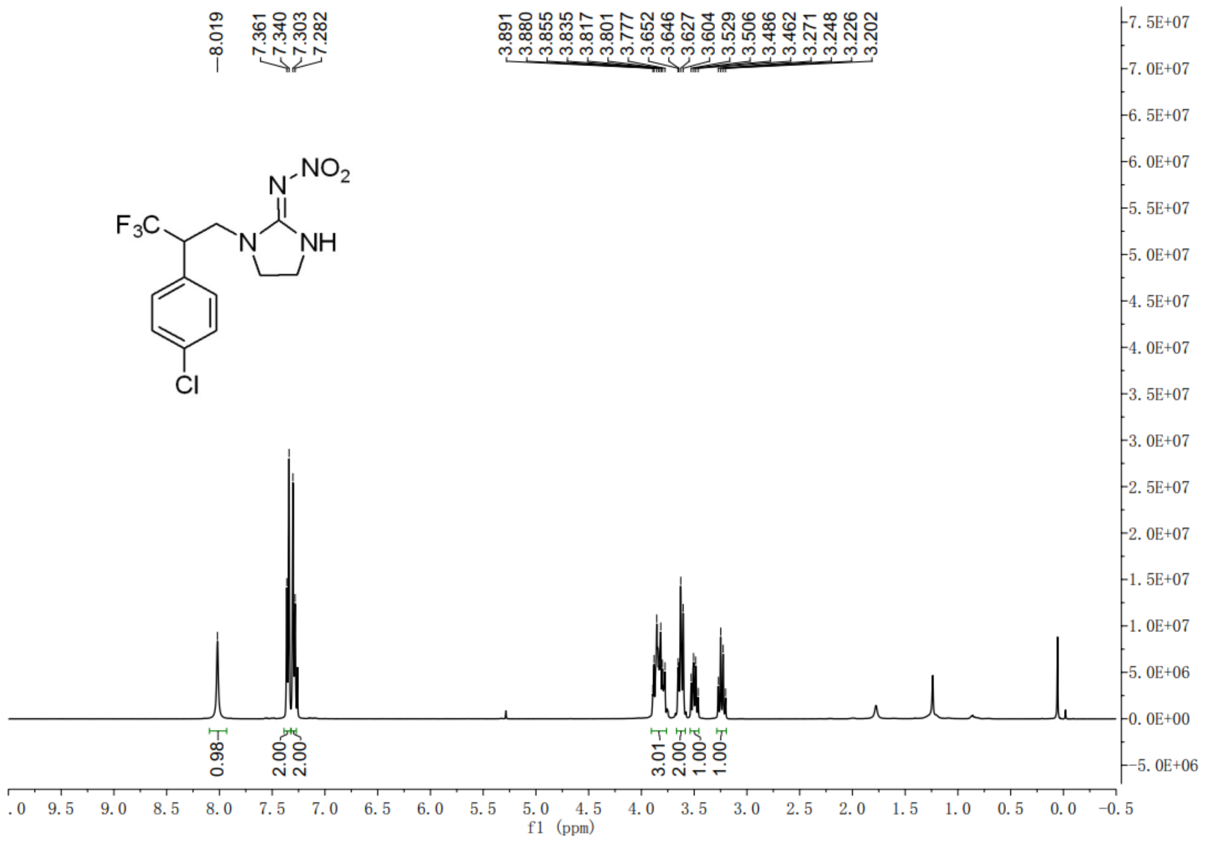

^{13}C NMR spectrum of 3ea (100 MHz, $CDCl_3$)

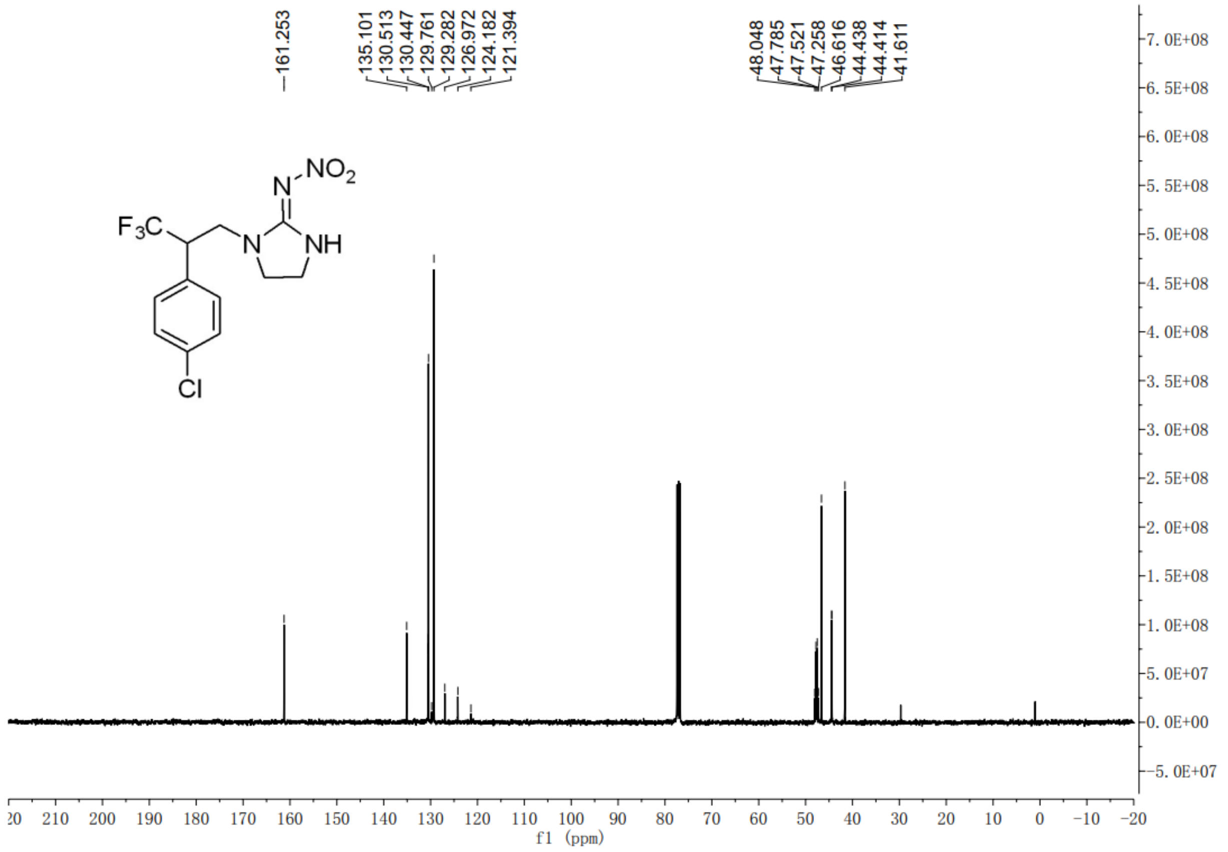

^{19}F NMR spectrum of 3ea (564 MHz, $CDCl_3$)

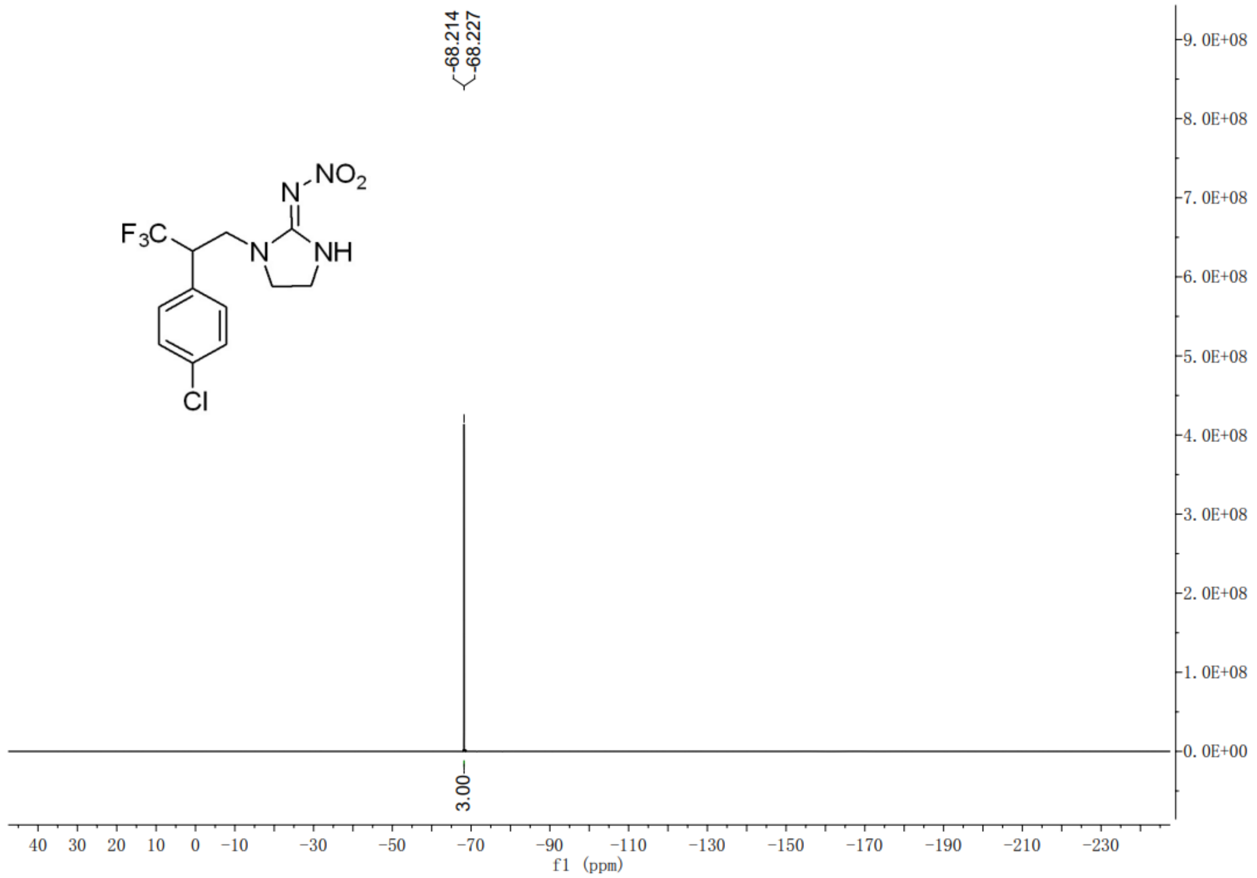

HRMS (EI) spectrum of 3ea

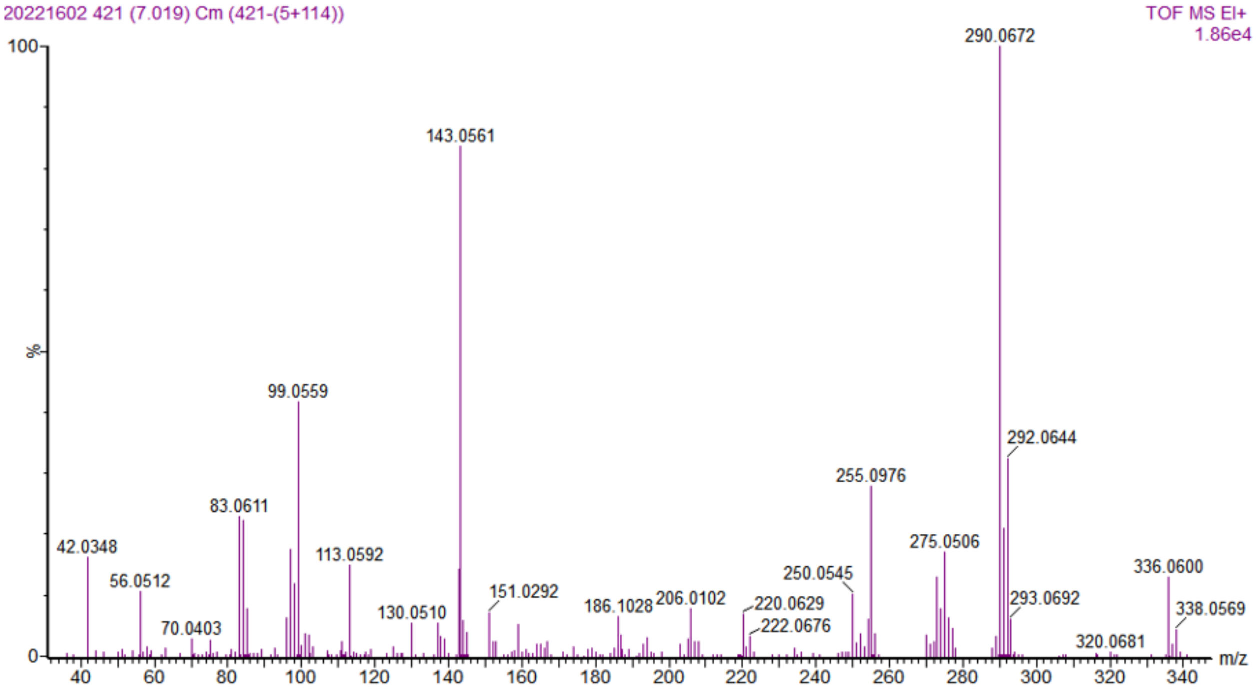

^{1}H NMR spectrum of 3fa (400 MHz, $CDCl_3$)

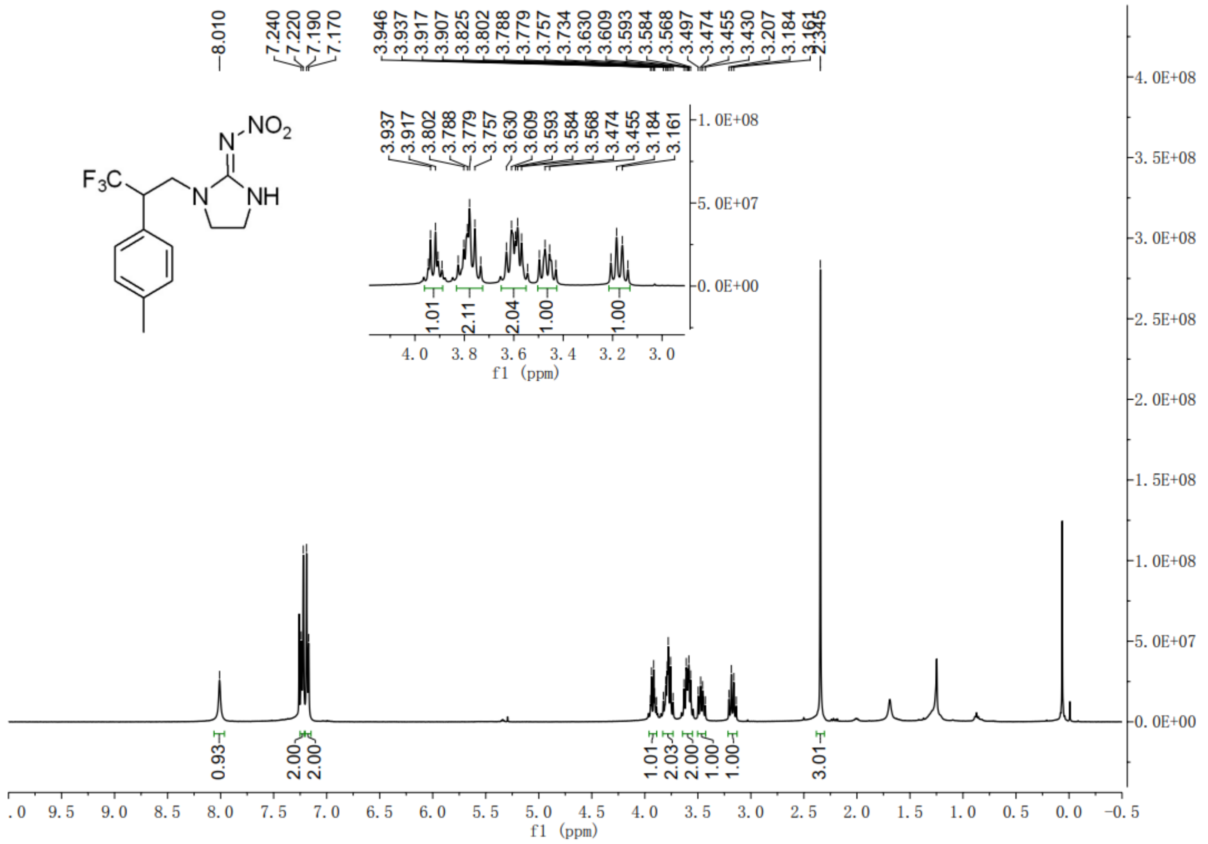

^{13}C NMR spectrum of 3fa (100 MHz, $CDCl_3$)

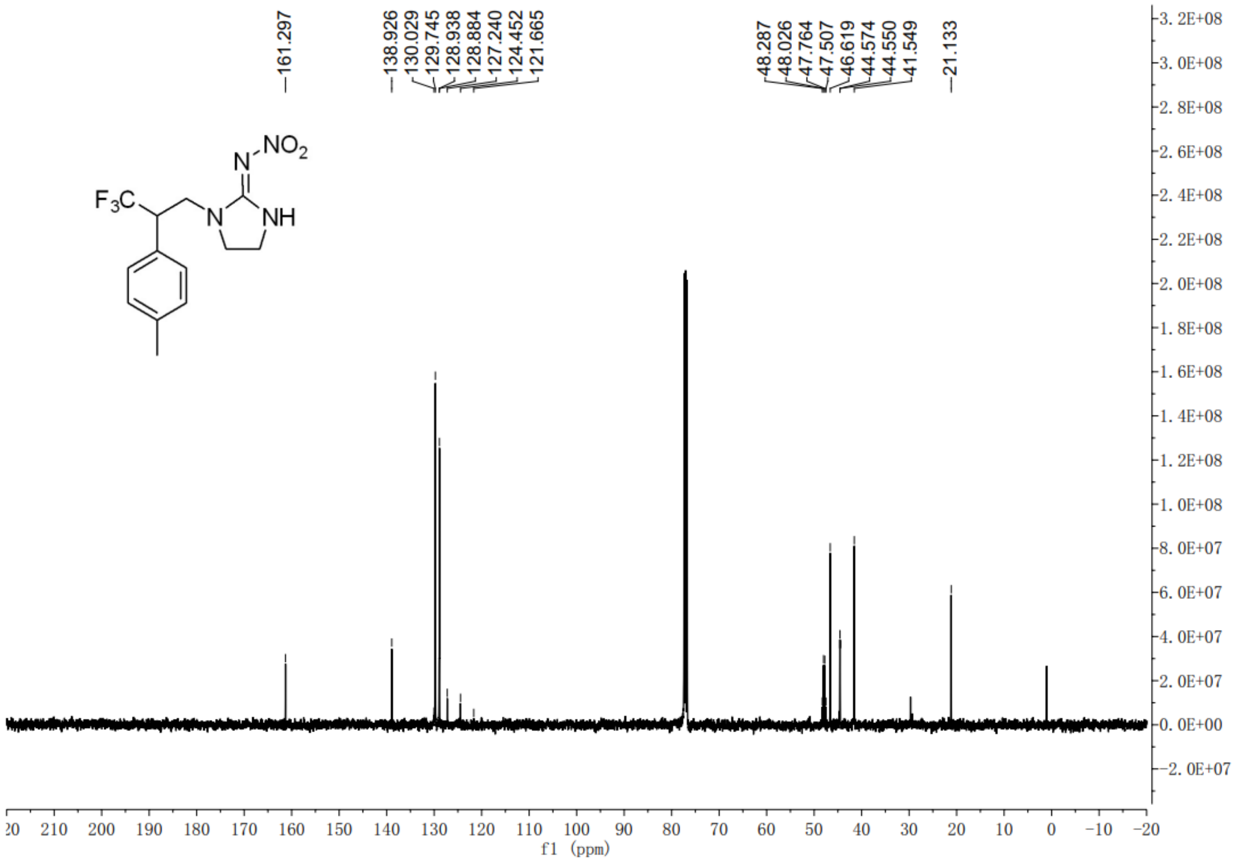

^{19}F NMR spectrum of 3fa (564 MHz, CDCl$_3$)

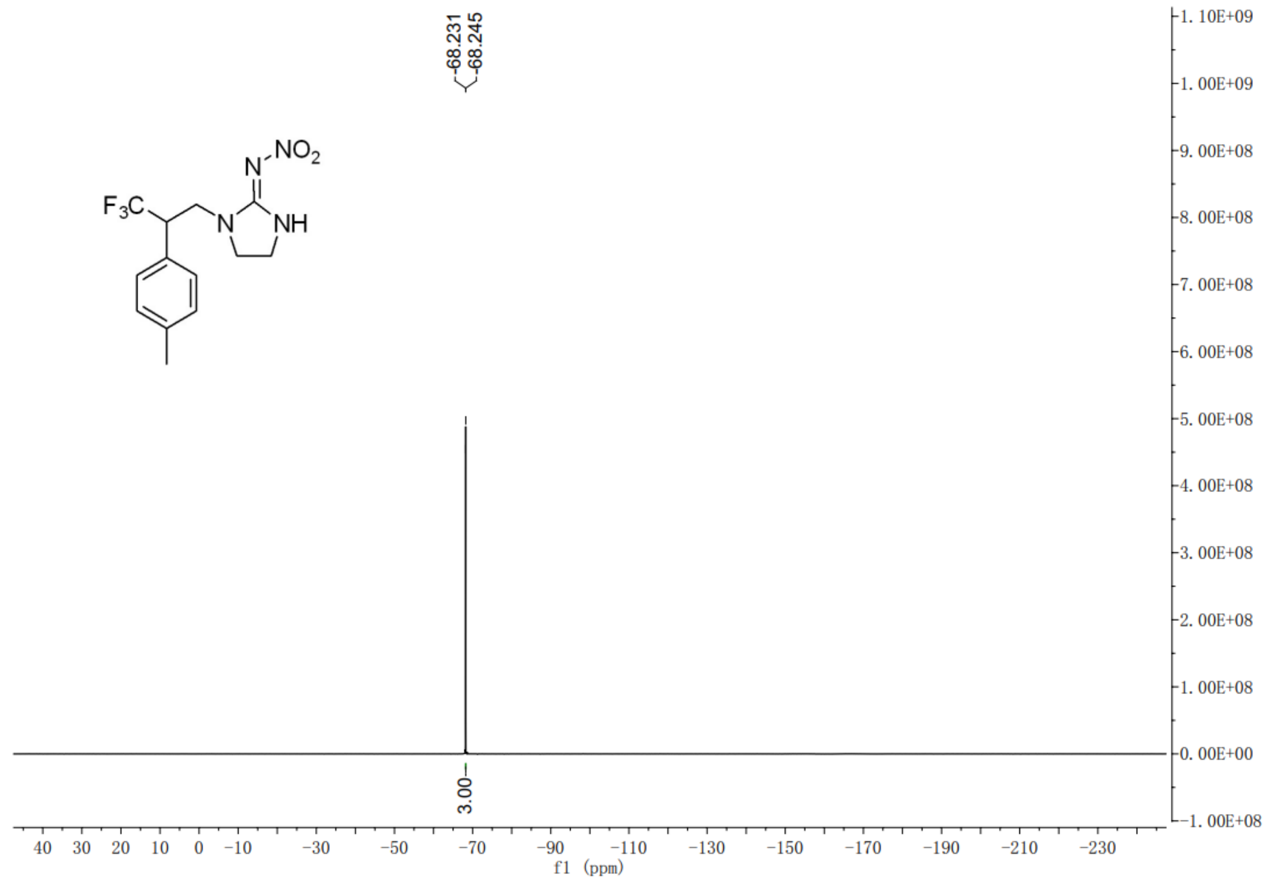

HRMS (EI) spectrum of 3fa

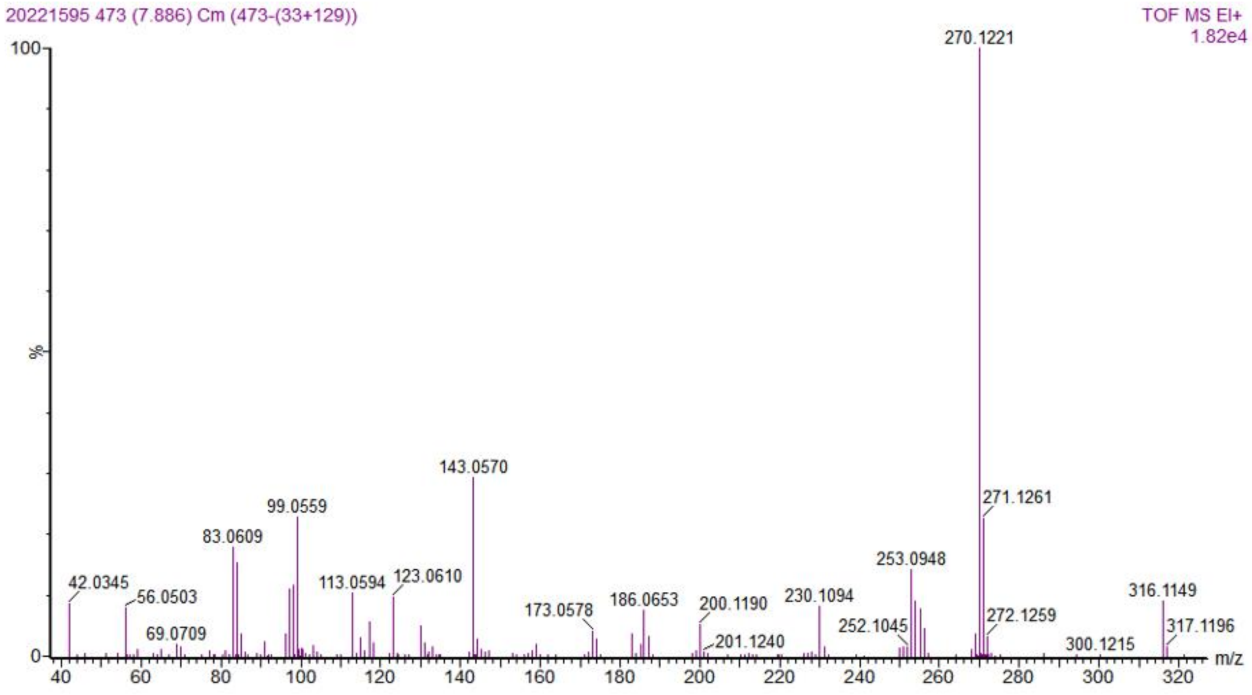

^{1}H NMR spectrum of 3ga (400 MHz, CDCl$_3$)

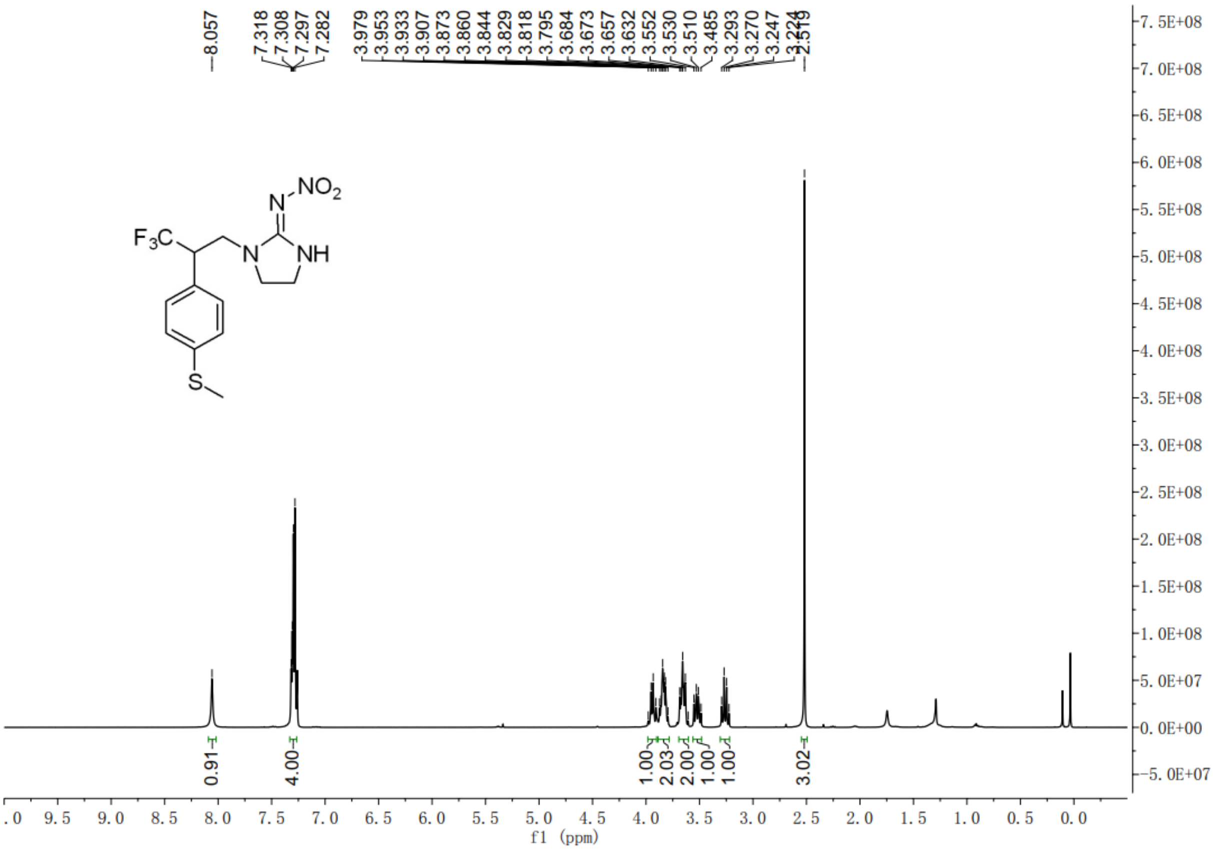

^{13}C NMR spectrum of 3ga (100 MHz, CDCl$_3$)

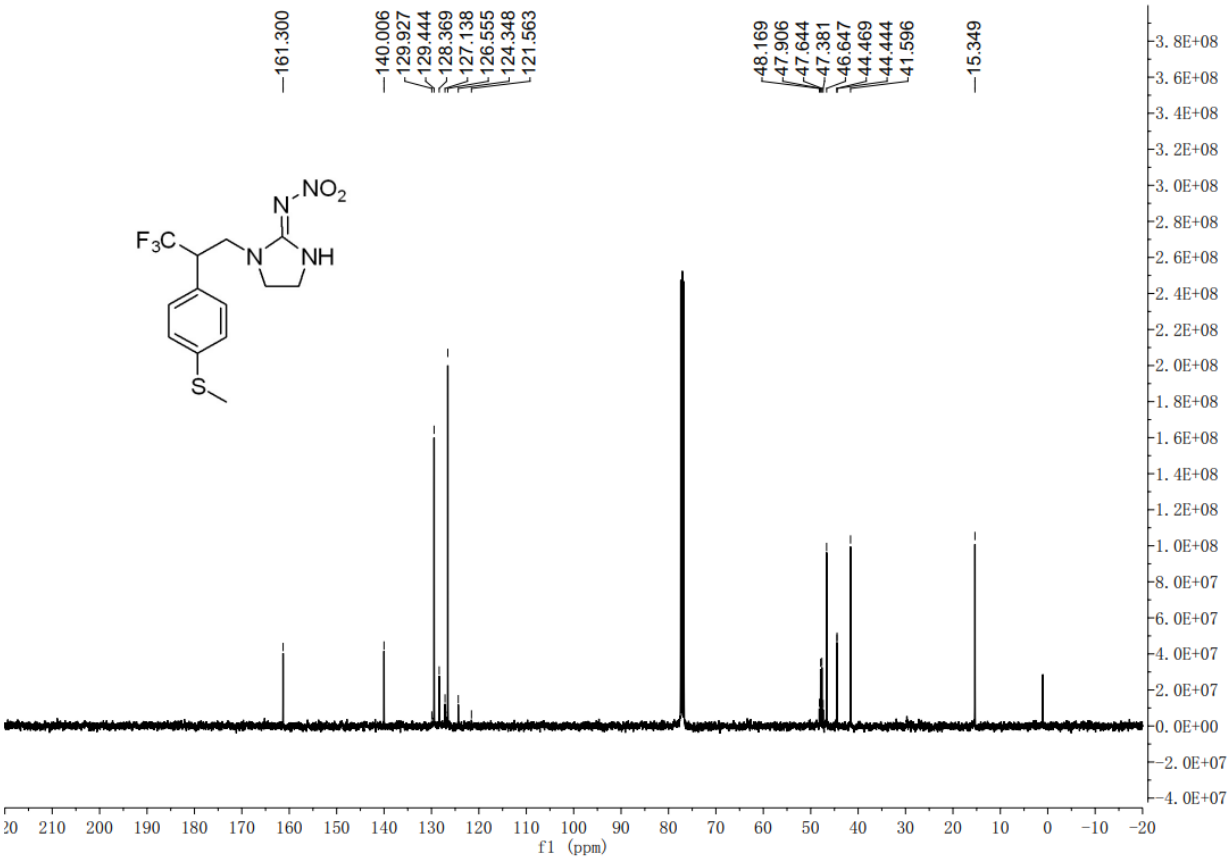

^{19}F NMR spectrum of 3ga (564 MHz, $CDCl_3$)

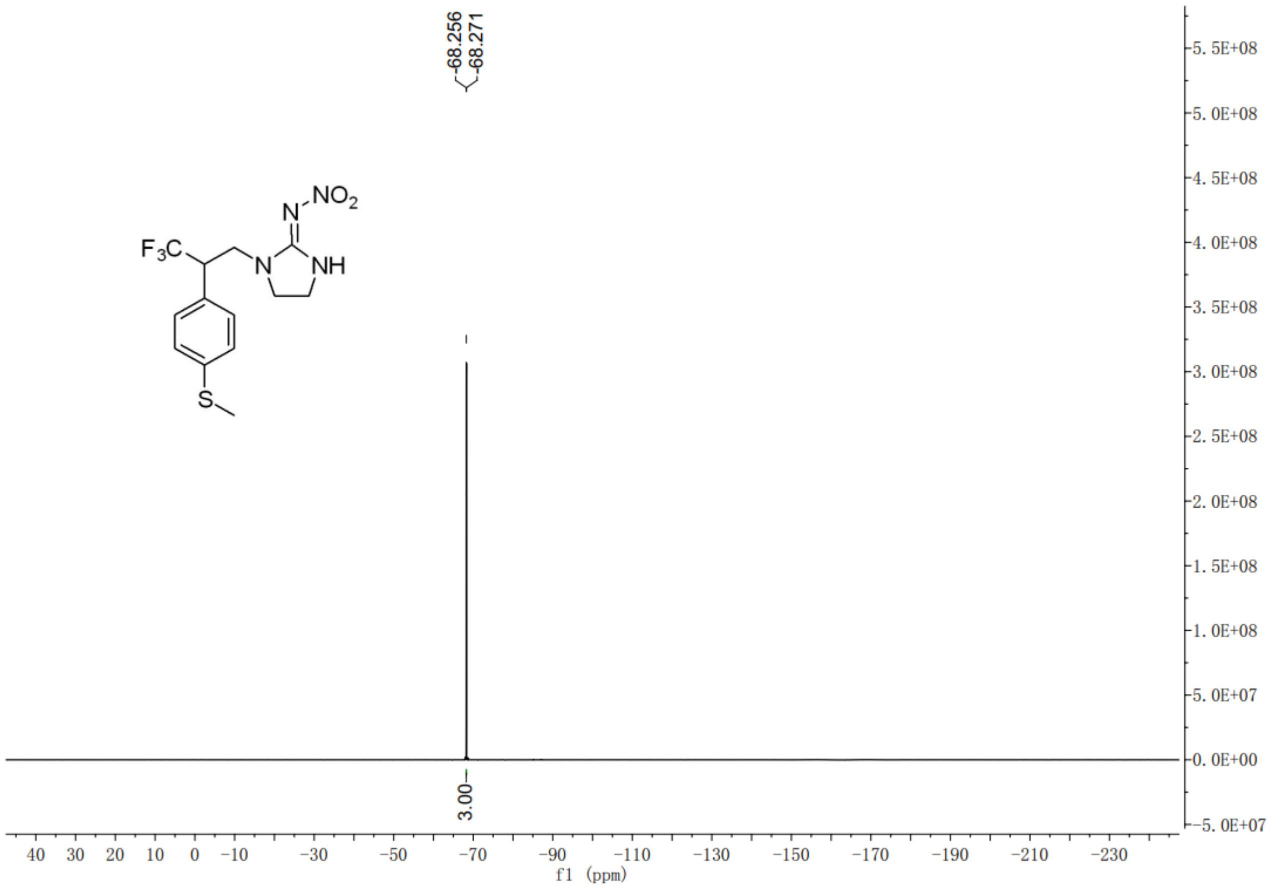

HRMS (EI) spectrum of 3ga

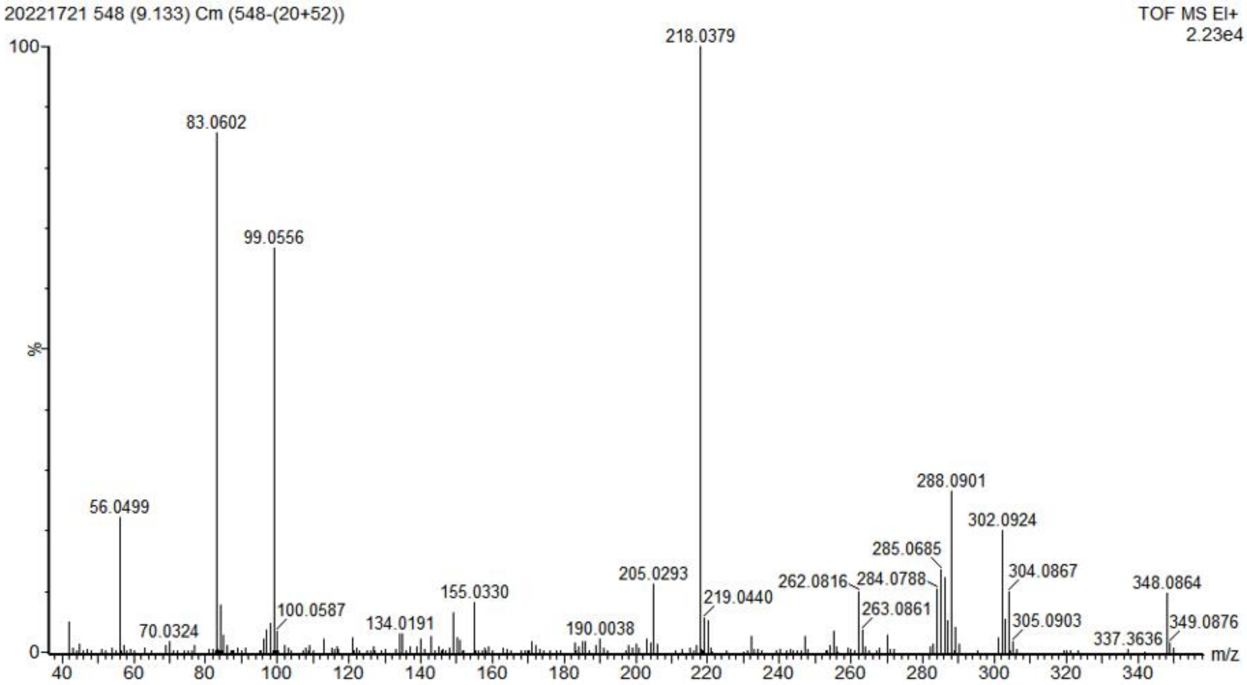

^{1}H NMR spectrum of 3ha (400 MHz, $CDCl_3$)

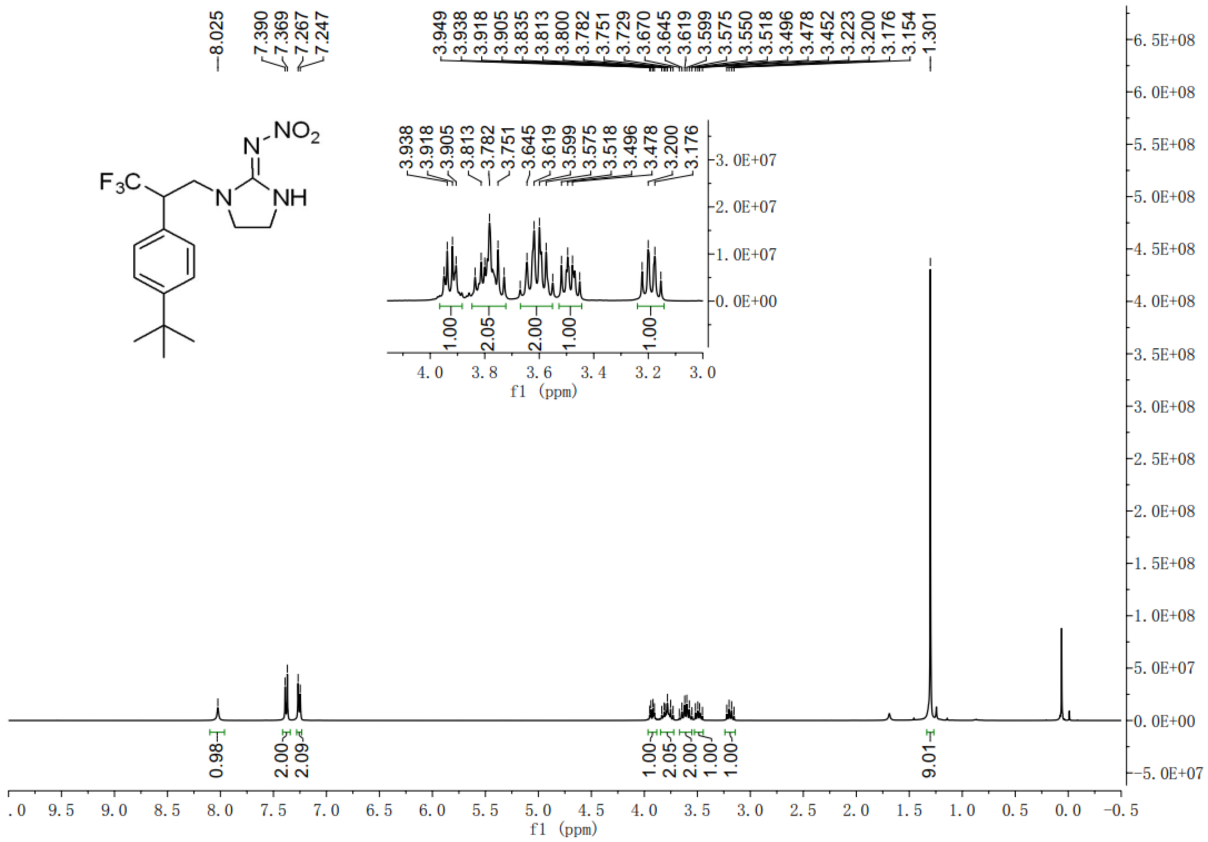

^{13}C NMR spectrum of 3ha (100 MHz, $CDCl_3$)

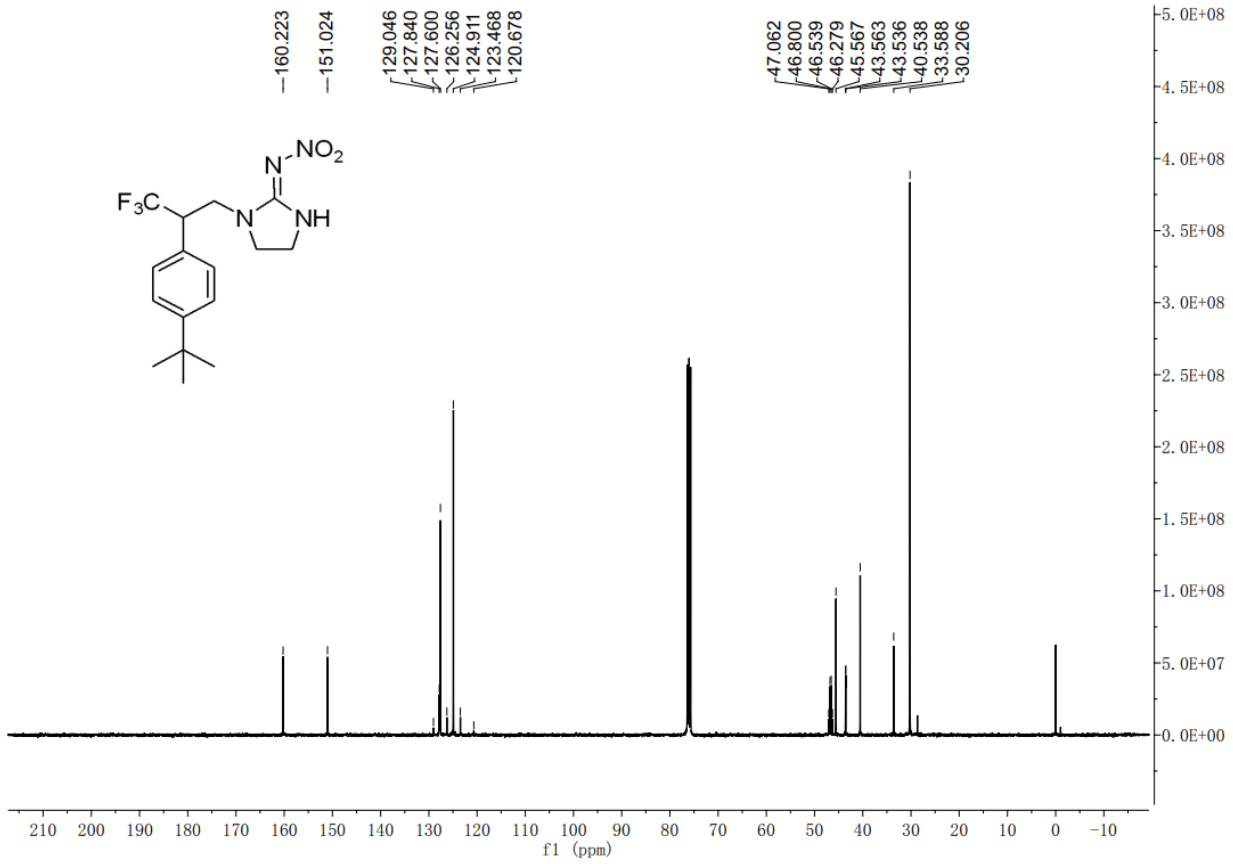

^{19}F NMR spectrum of 3ha (564 MHz, $CDCl_3$)

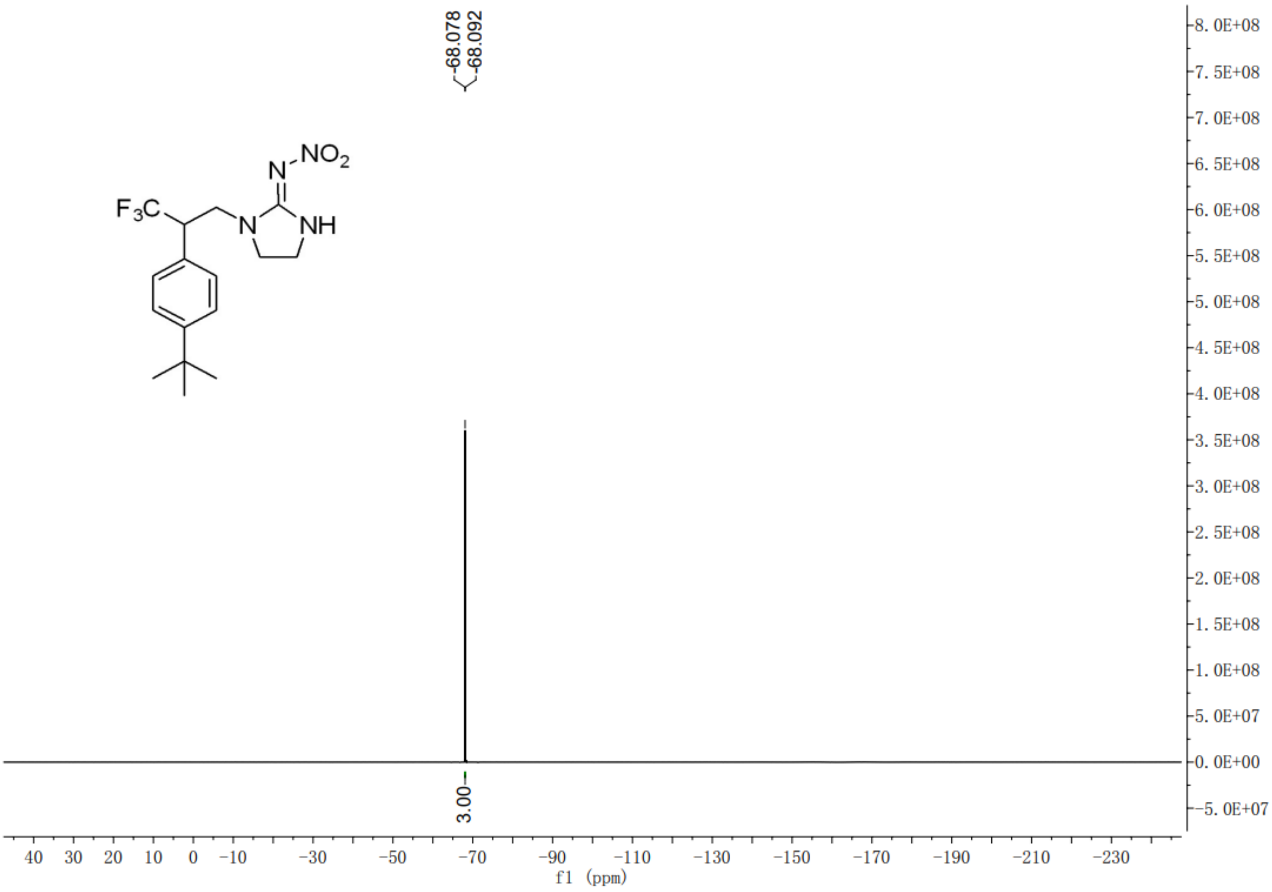

HRMS (EI) spectrum of 3ha

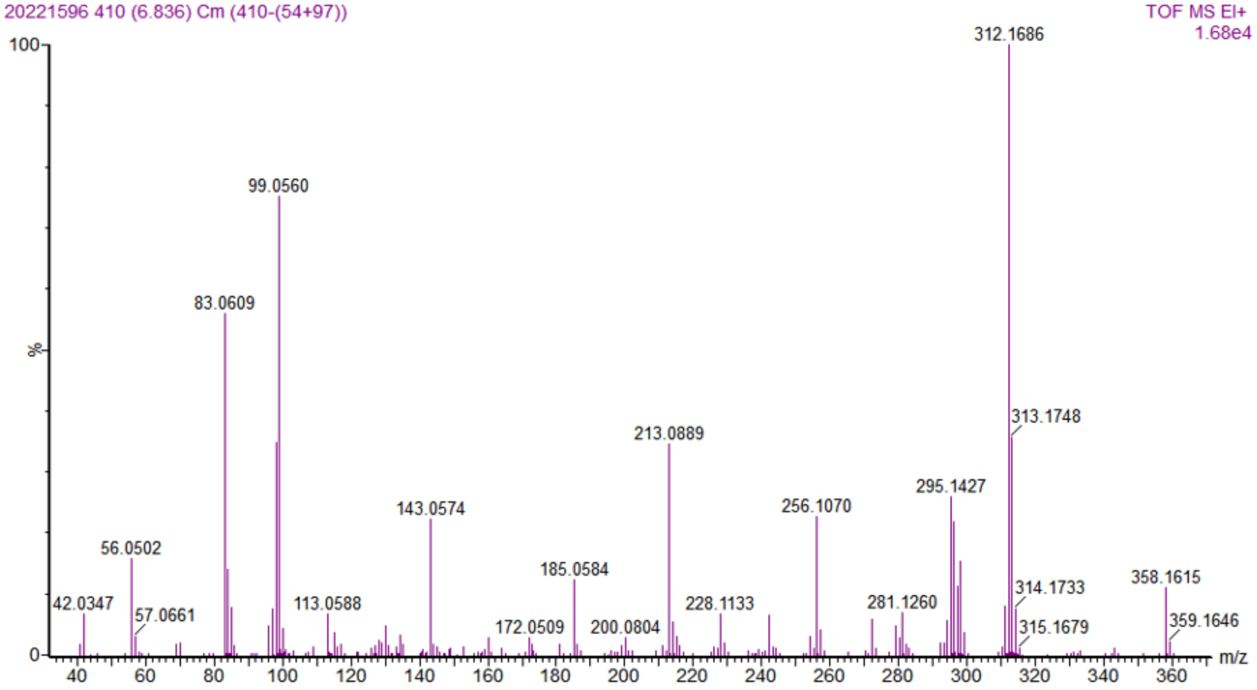

^{1}H NMR spectrum of 3ia (400 MHz, $CDCl_3$)

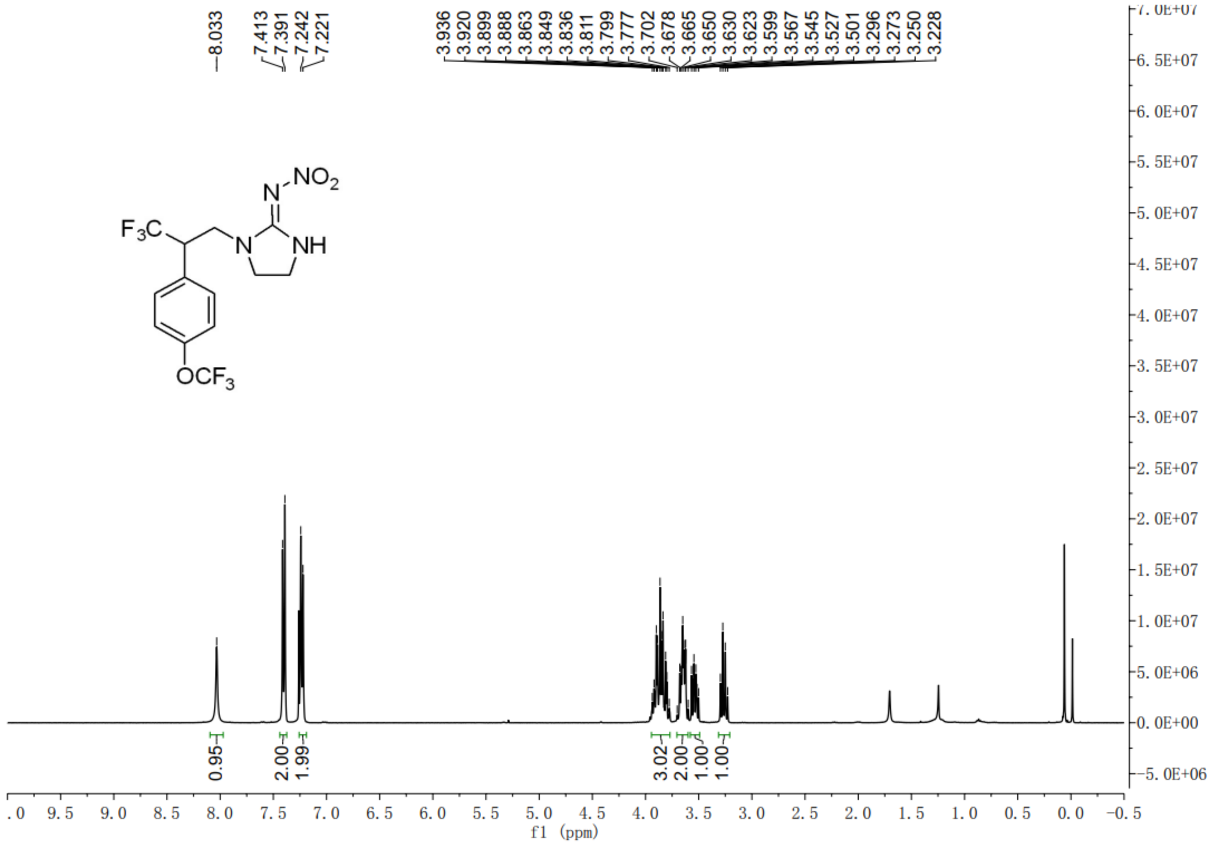

^{13}C NMR spectrum of 3ia (100 MHz, $CDCl_3$)

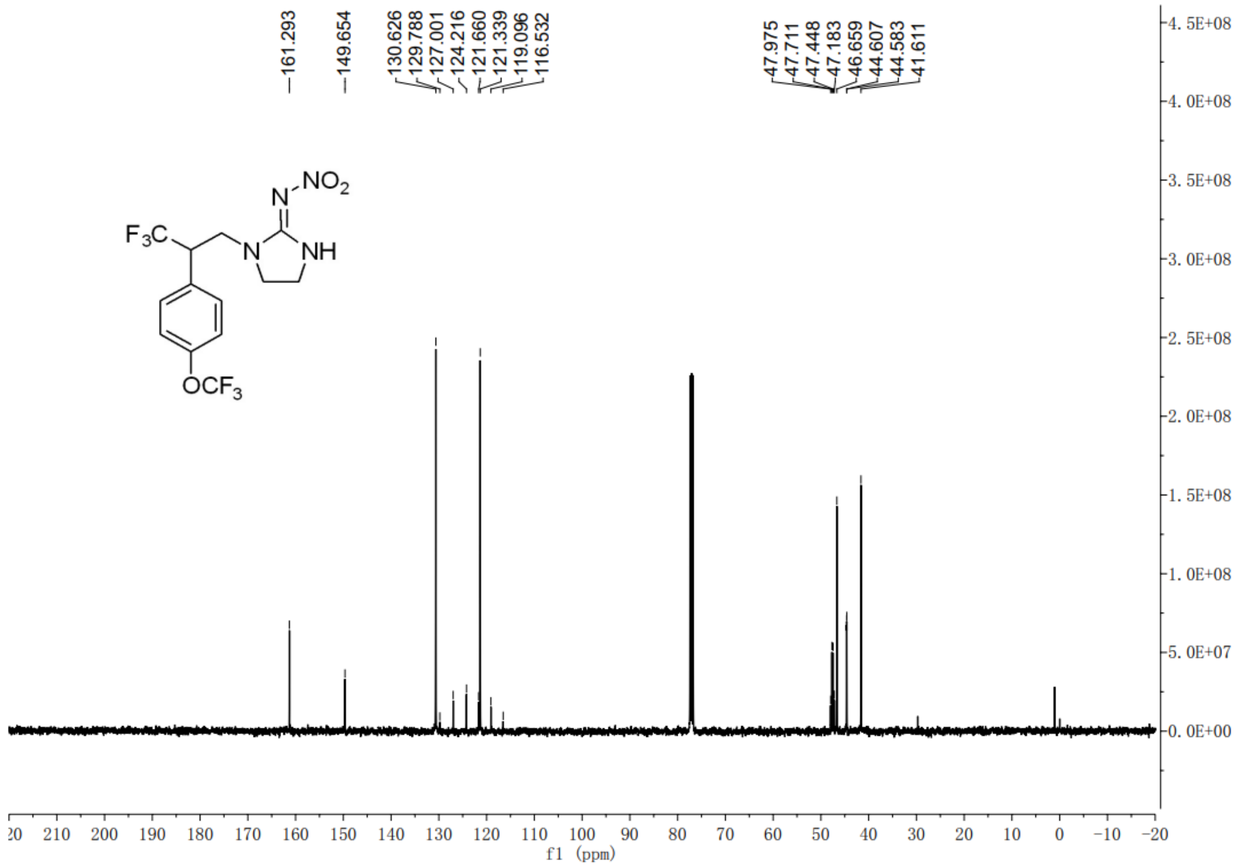

^{19}F NMR spectrum of 3ia (564 MHz, $CDCl_3$)

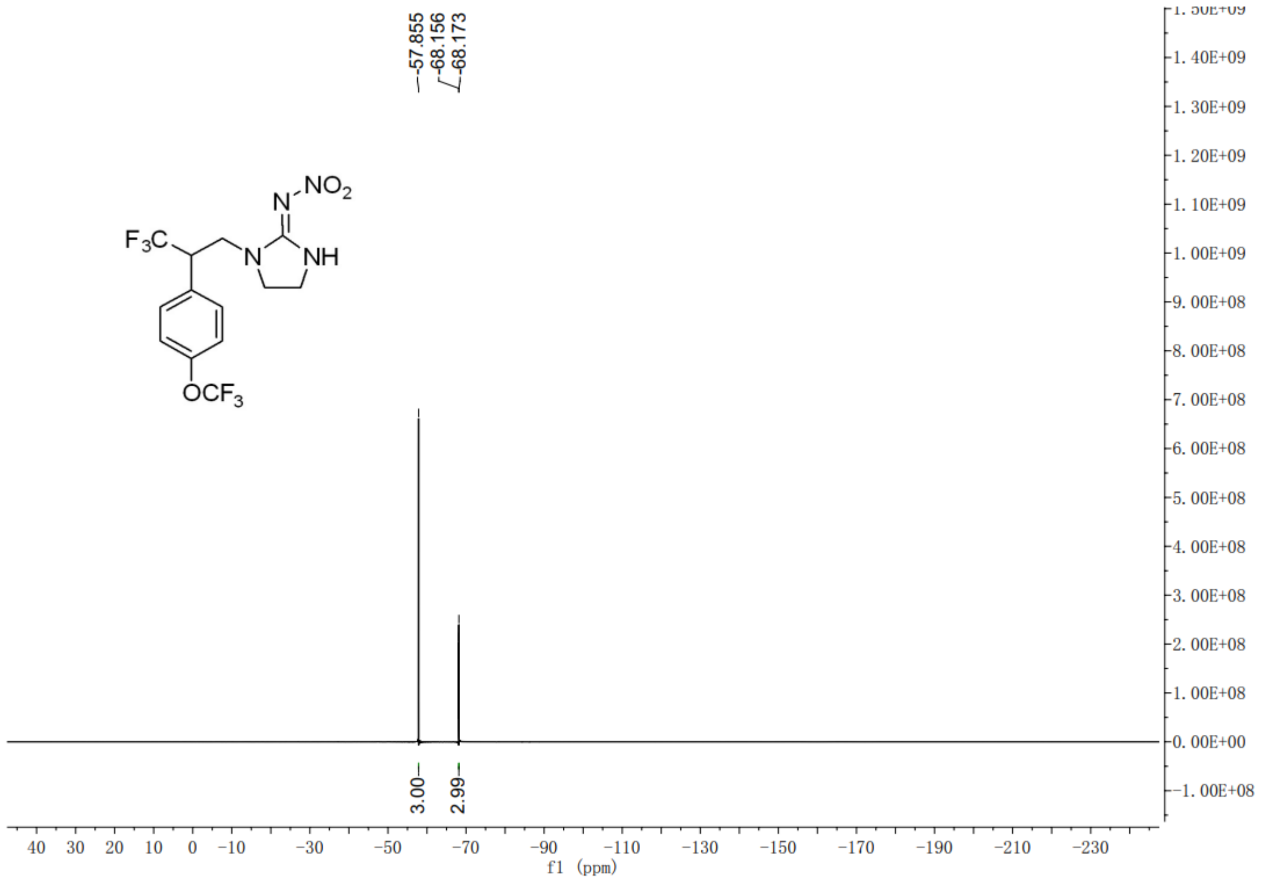

HRMS (EI) spectrum of 3ia

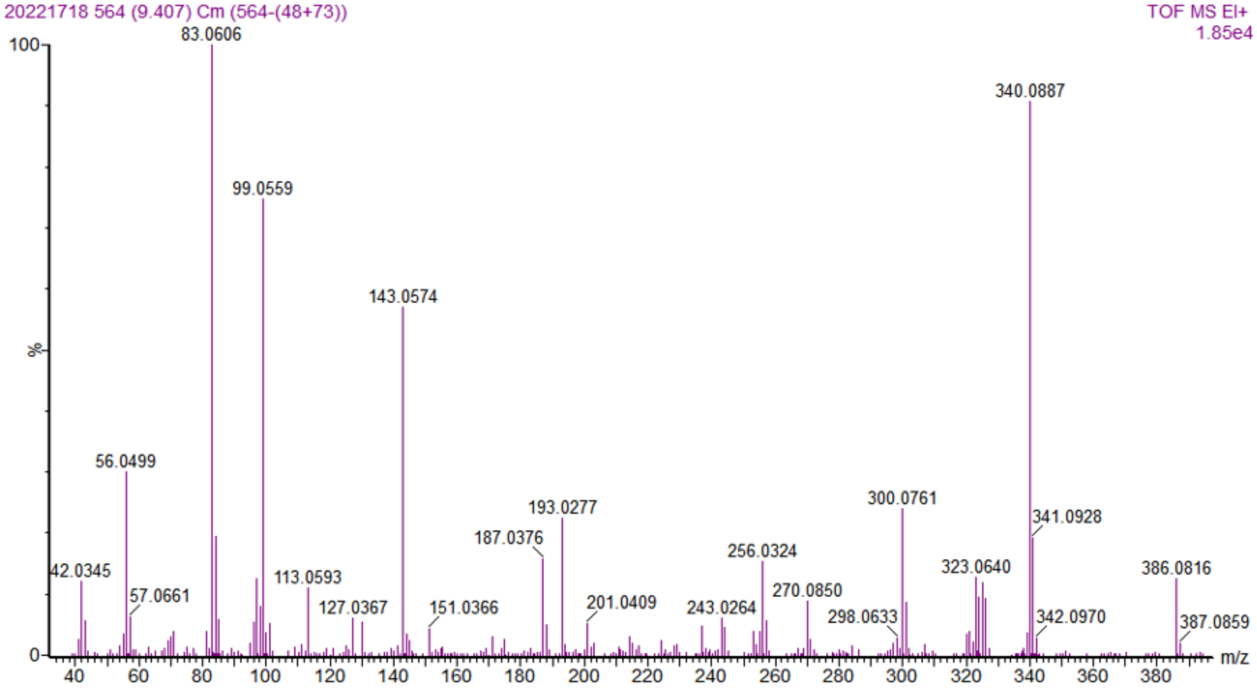

^{1}H NMR spectrum of 3ja (400 MHz, $CDCl_3$)

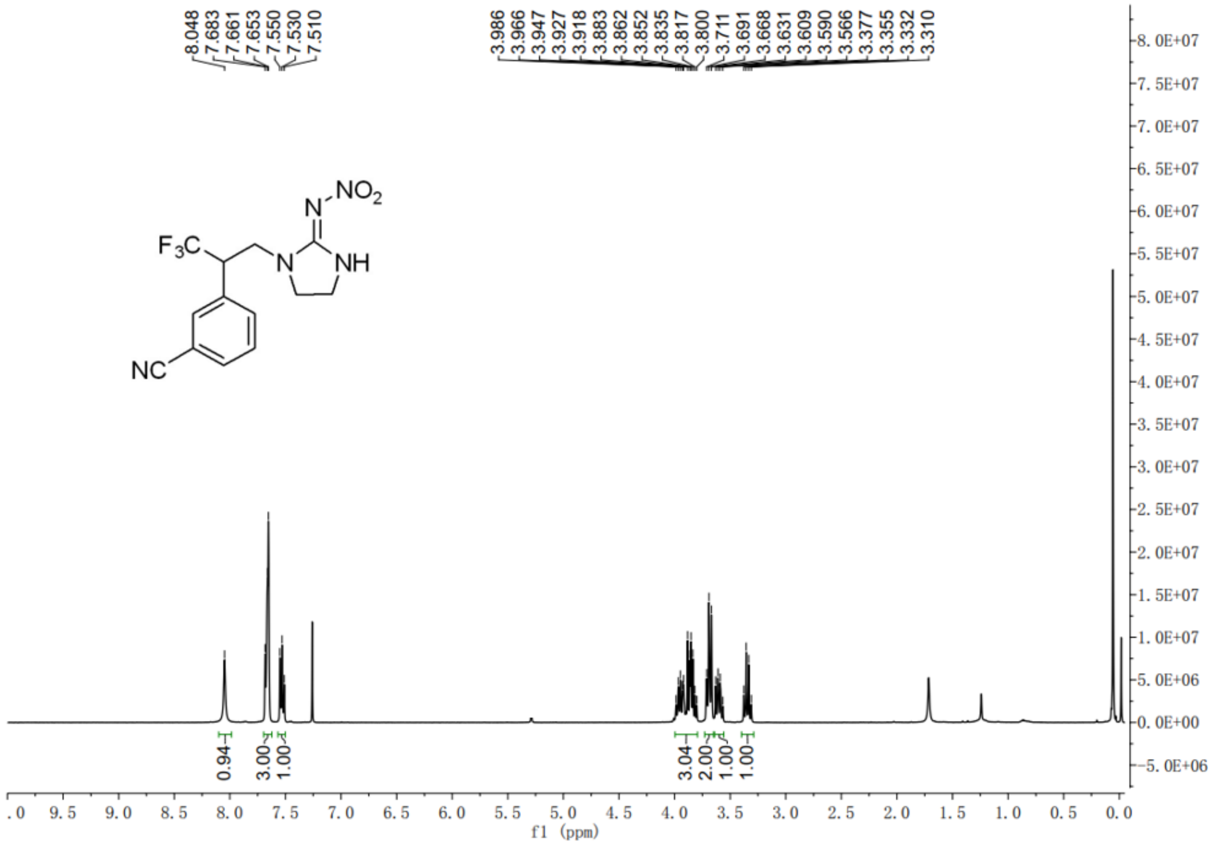

^{13}C NMR spectrum of 3ja (100 MHz, $CDCl_3$)

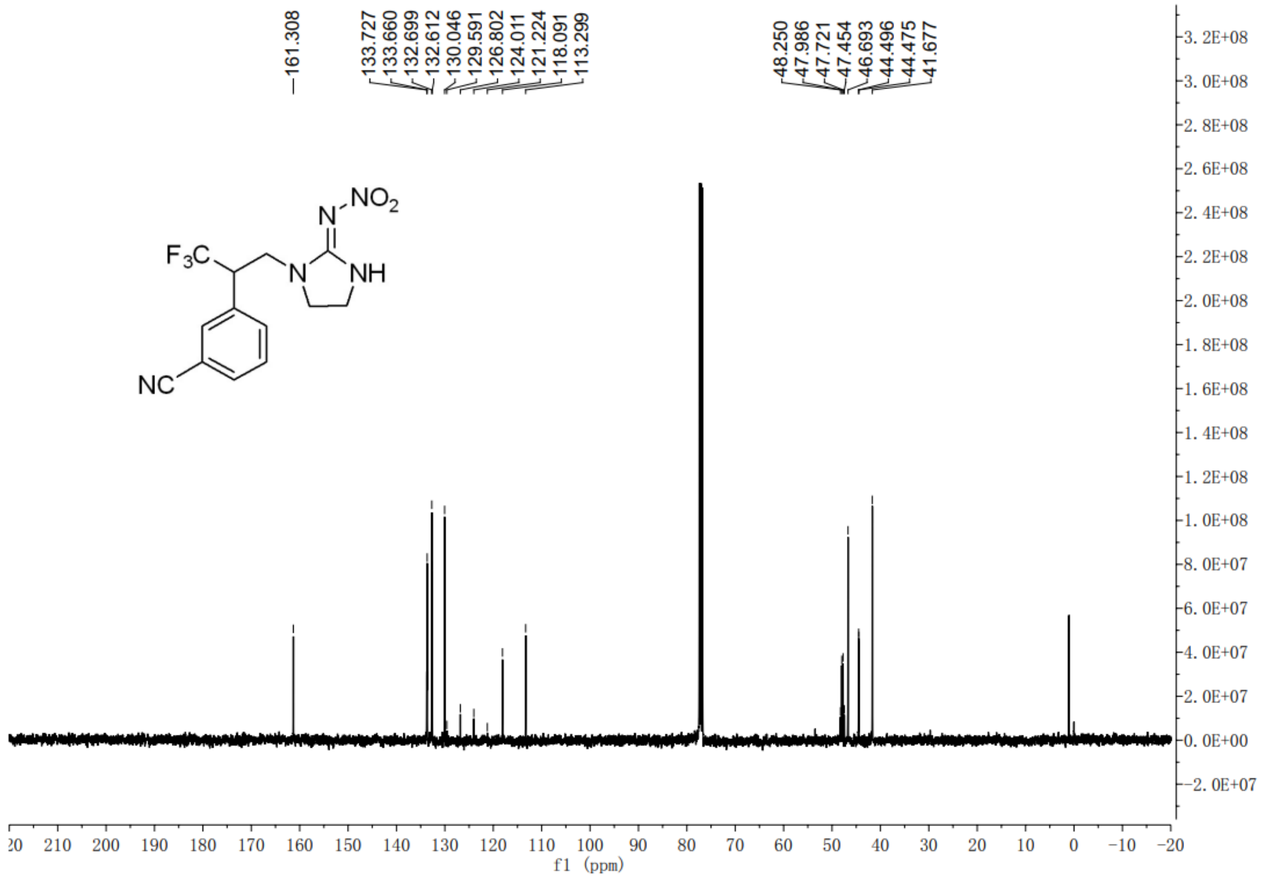

^{19}F NMR spectrum of 3ja (564 MHz, $CDCl_3$)

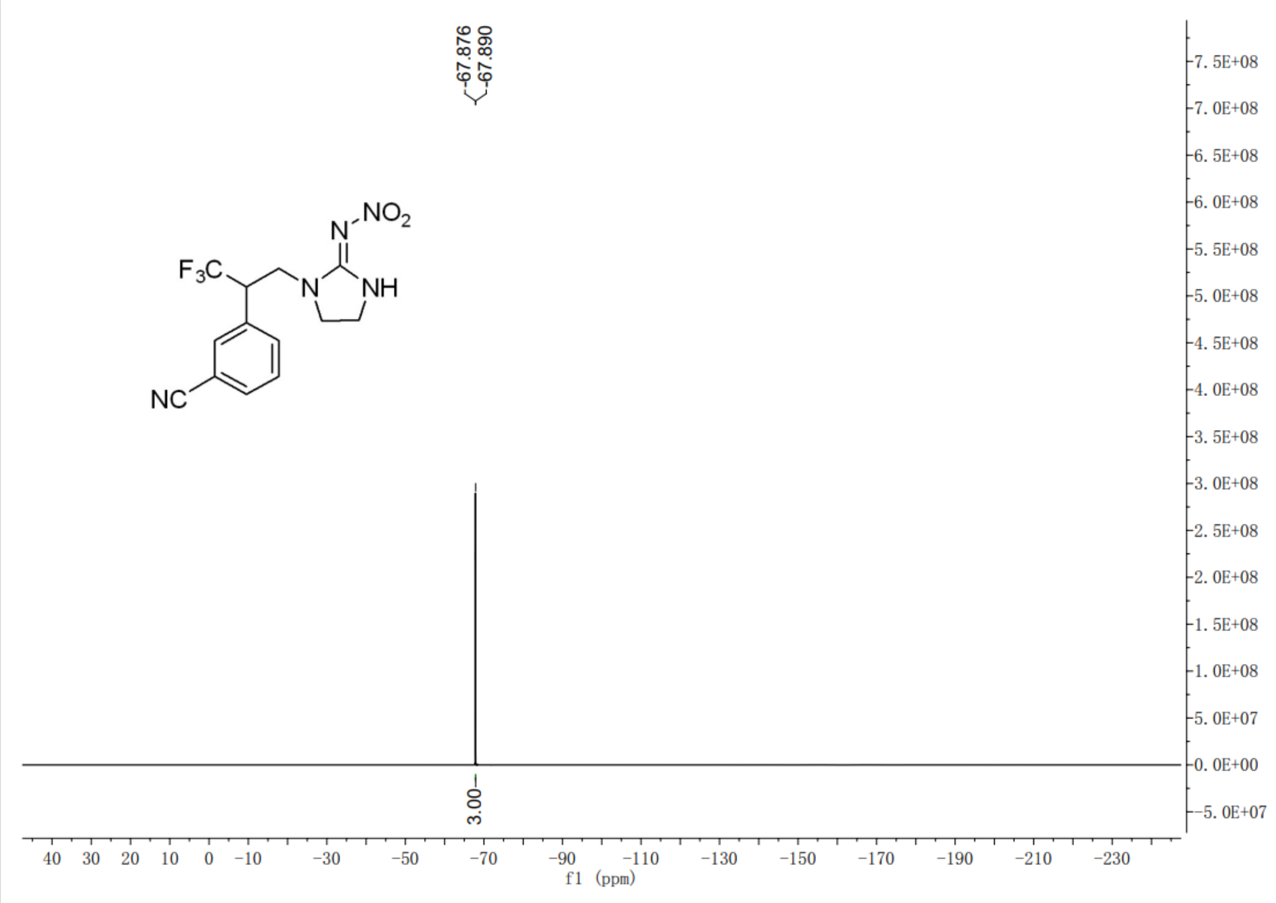

HRMS (EI) spectrum of 3ja

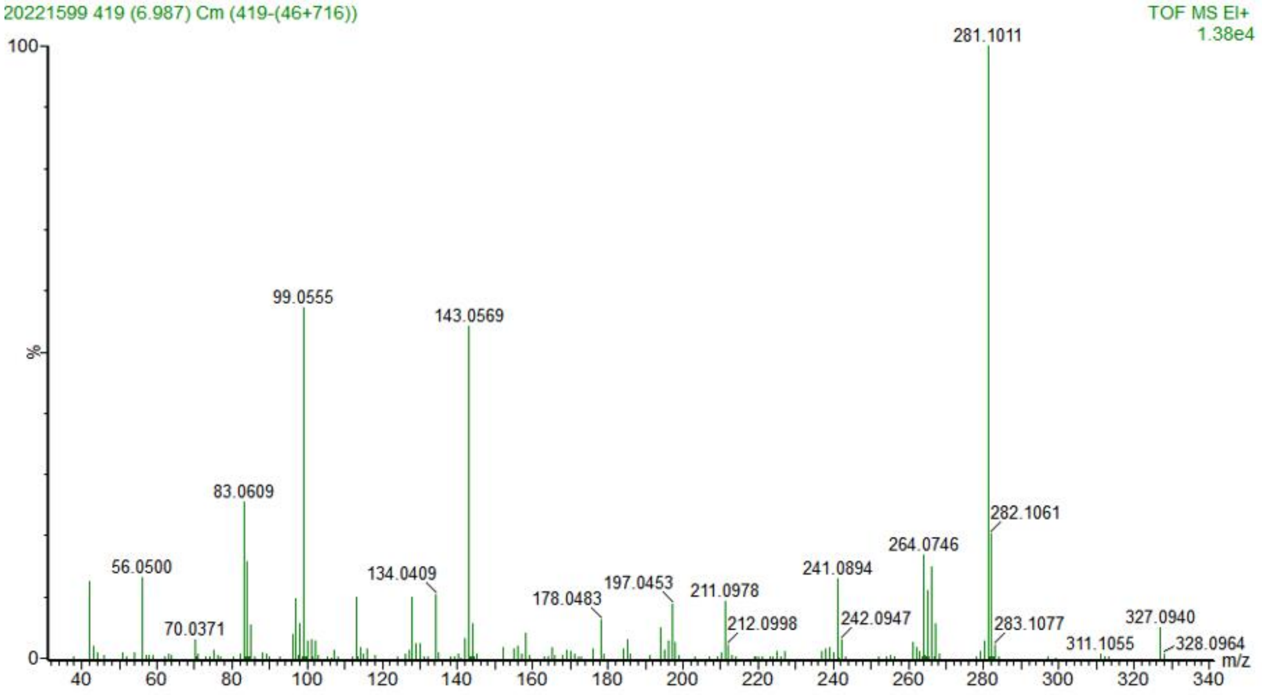

^{1}H NMR spectrum of 3ka (400 MHz, $CDCl_3$)

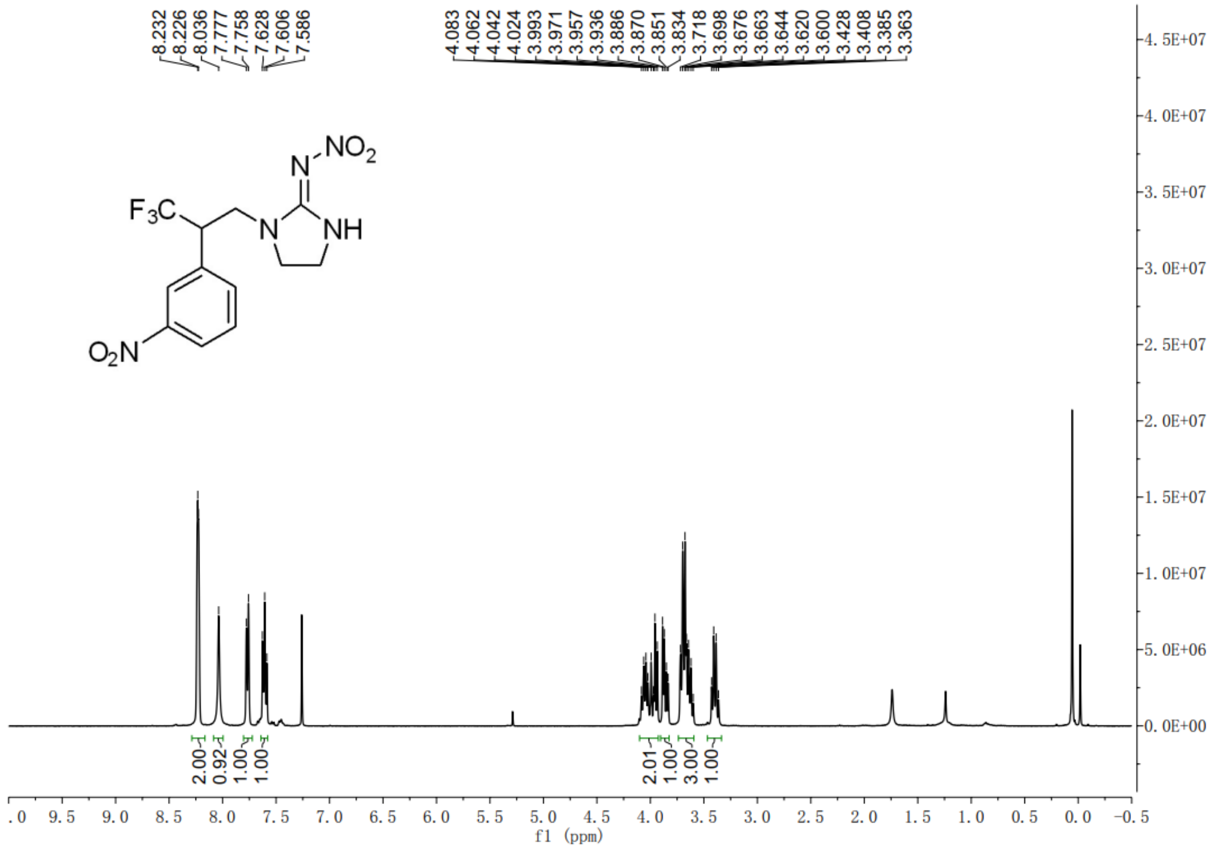

^{13}C NMR spectrum of 3ka (100 MHz, $CDCl_3$)

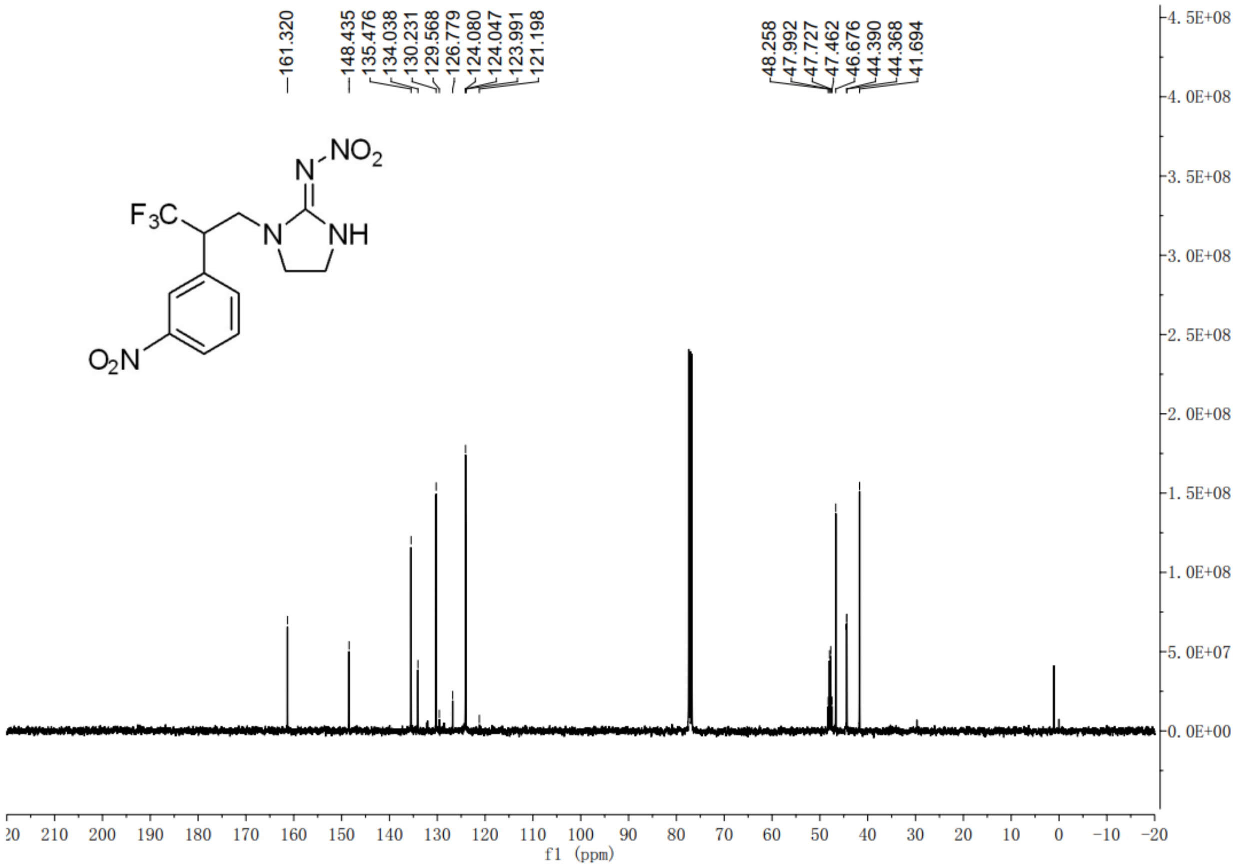

^{19}F NMR spectrum of 3ka (564 MHz, $CDCl_3$)

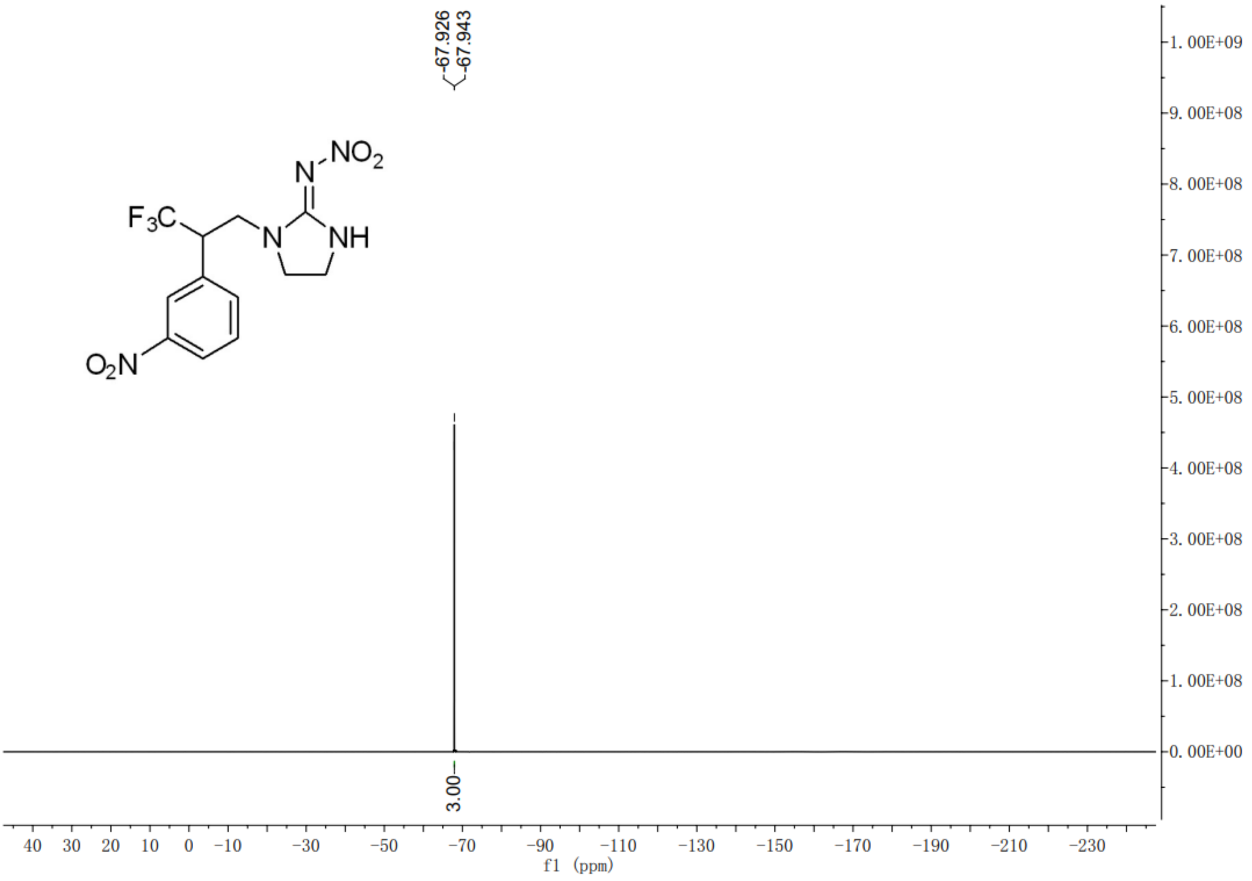

HRMS (EI) spectrum of 3ka

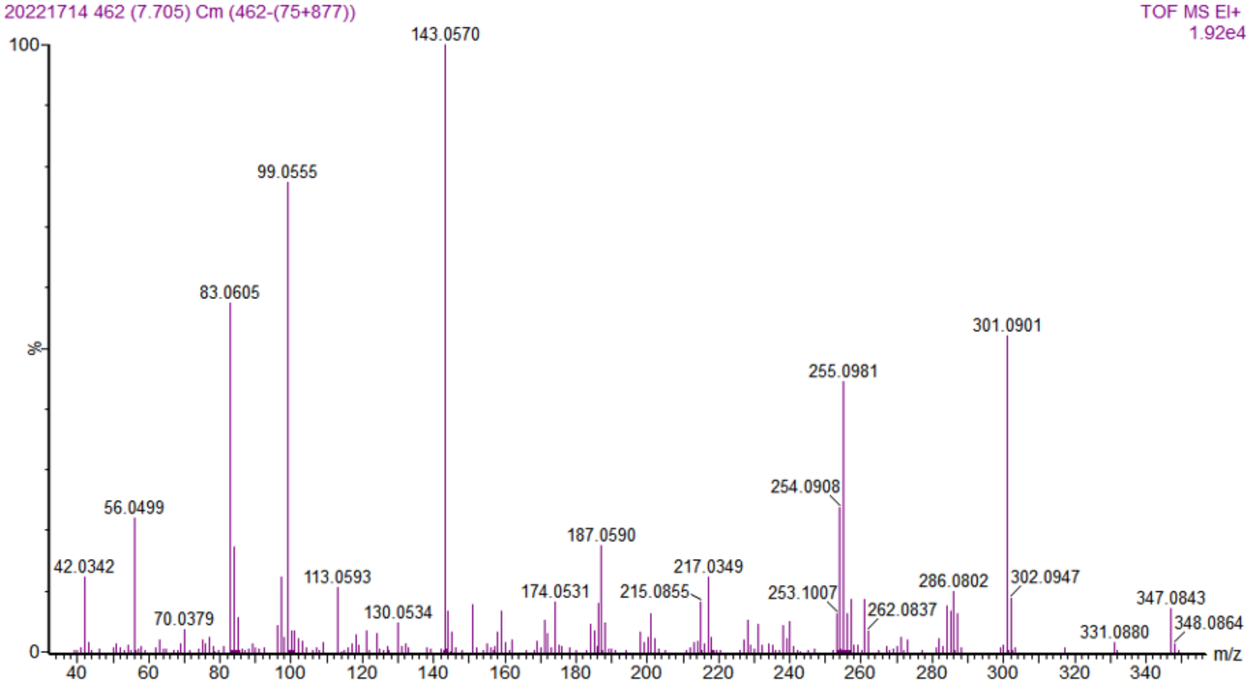

^{1}H NMR spectrum of 3la (400 MHz, $CDCl_3$)

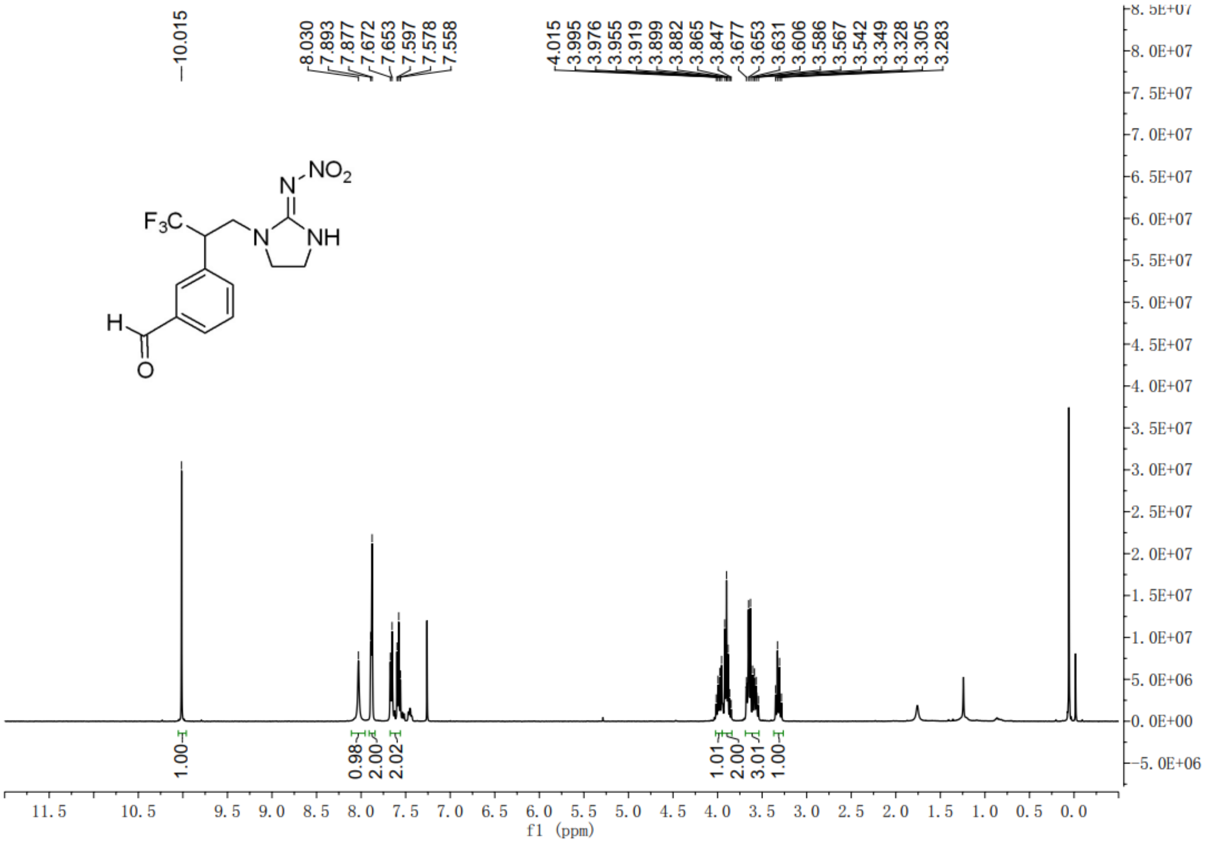

^{13}C NMR spectrum of 3la (100 MHz, $CDCl_3$)

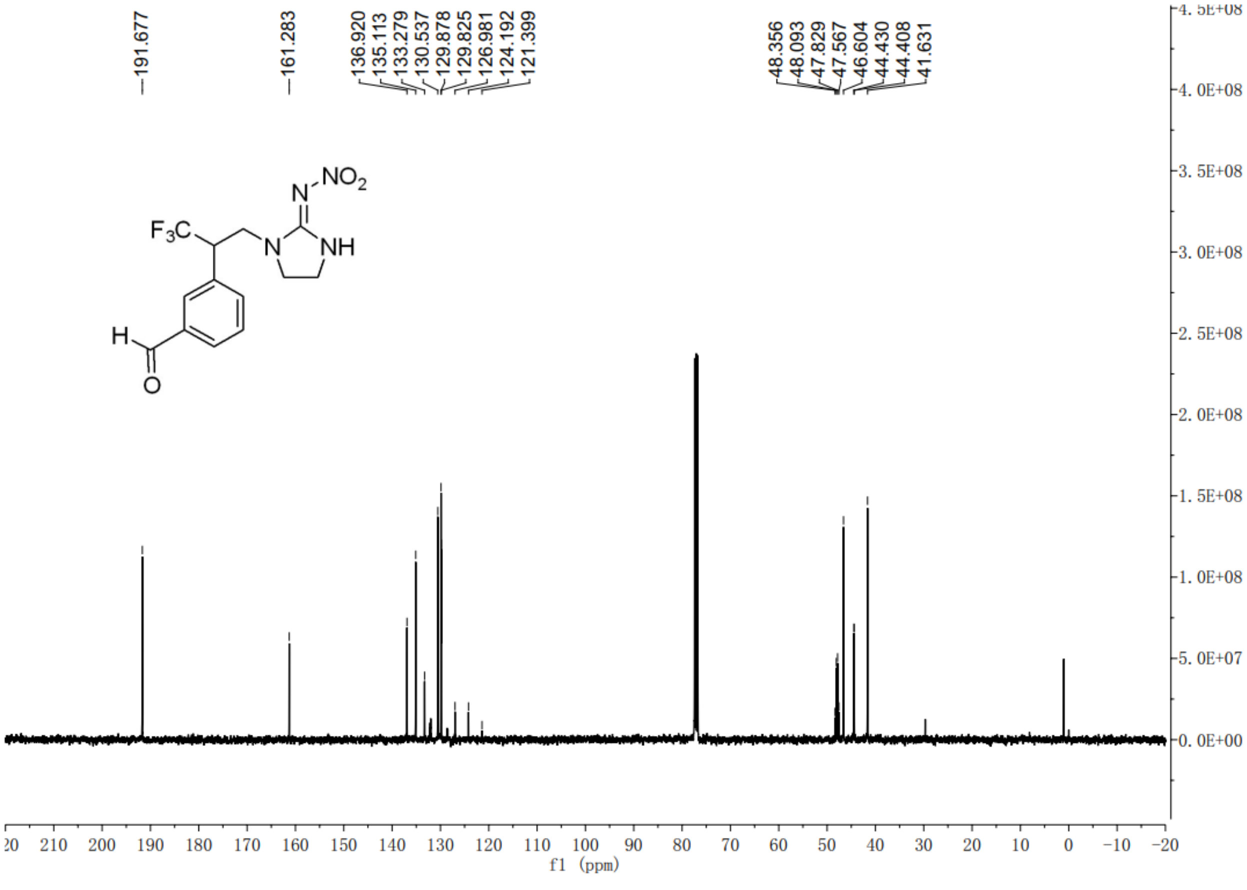

^{19}F NMR spectrum of 3la (564 MHz, $CDCl_3$)

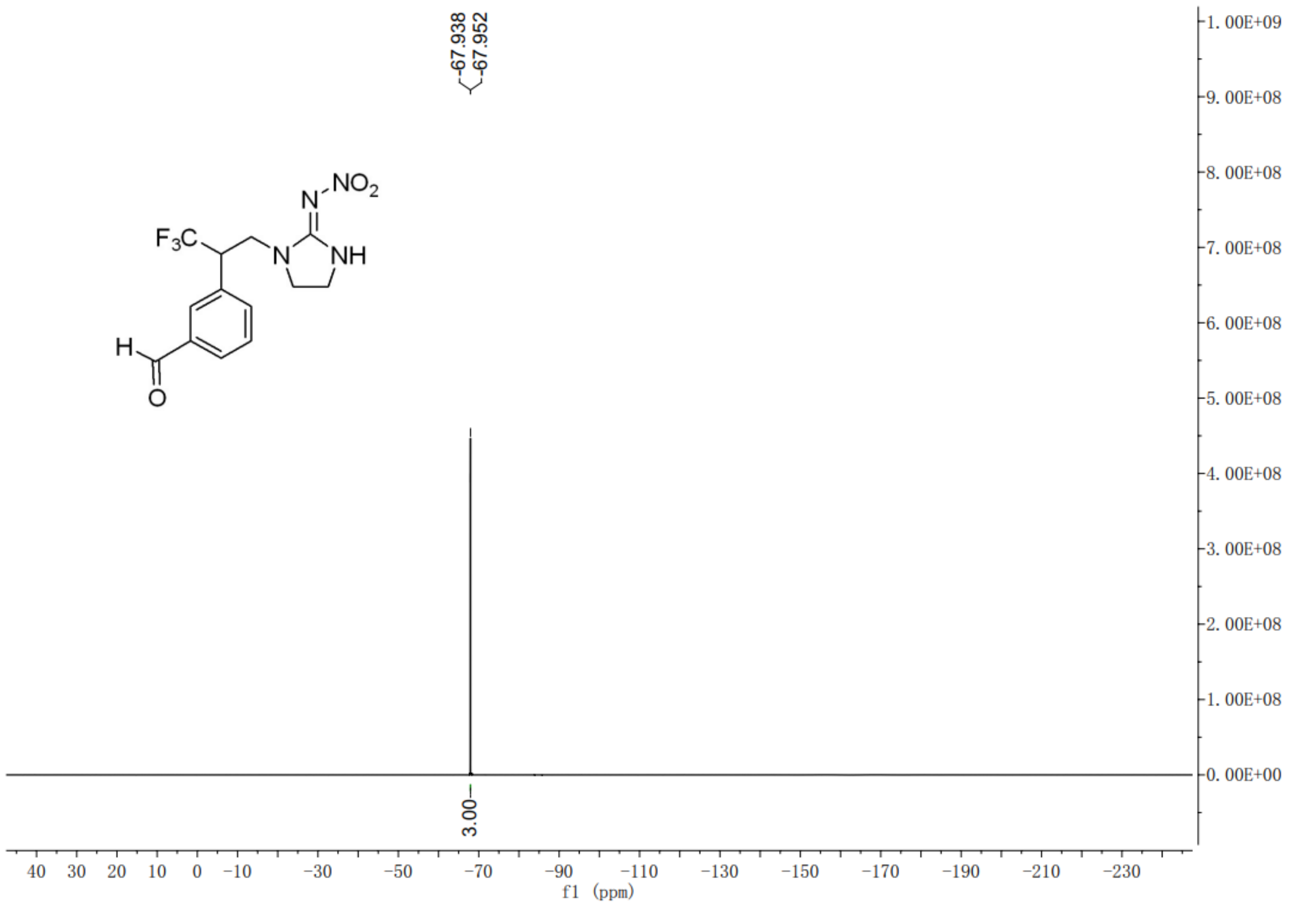

HRMS (EI) spectrum of 3la

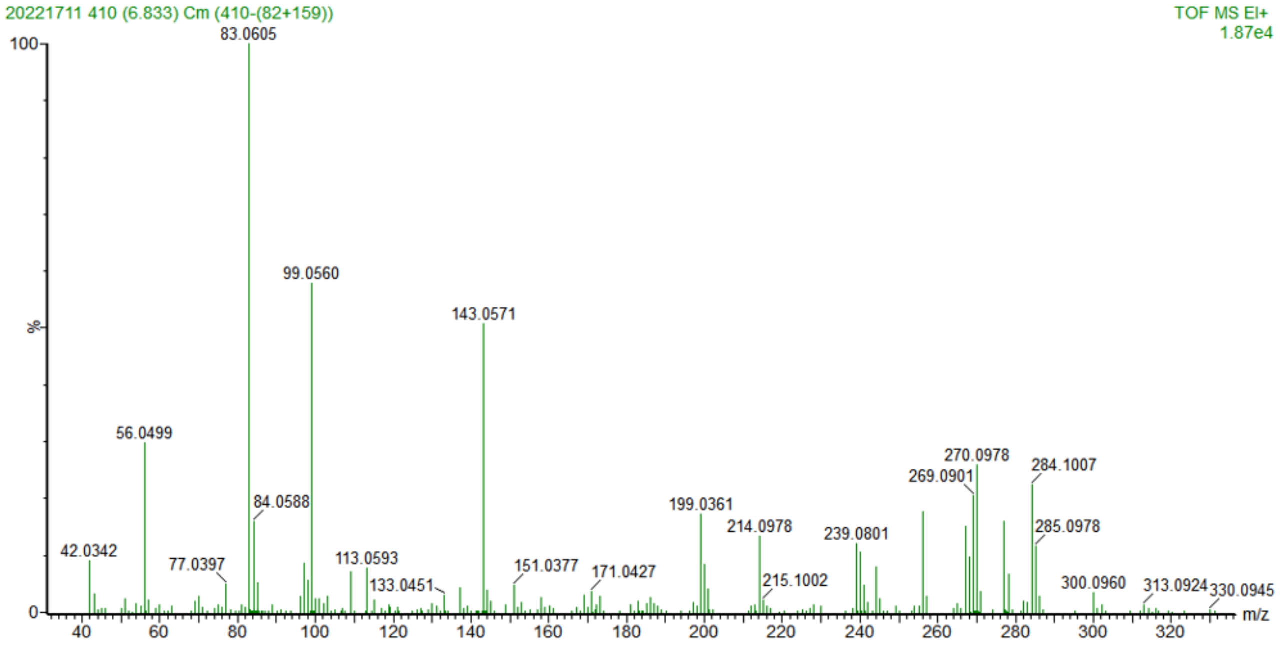

^{1}H NMR spectrum of 3ma (400 MHz, CDCl$_3$)

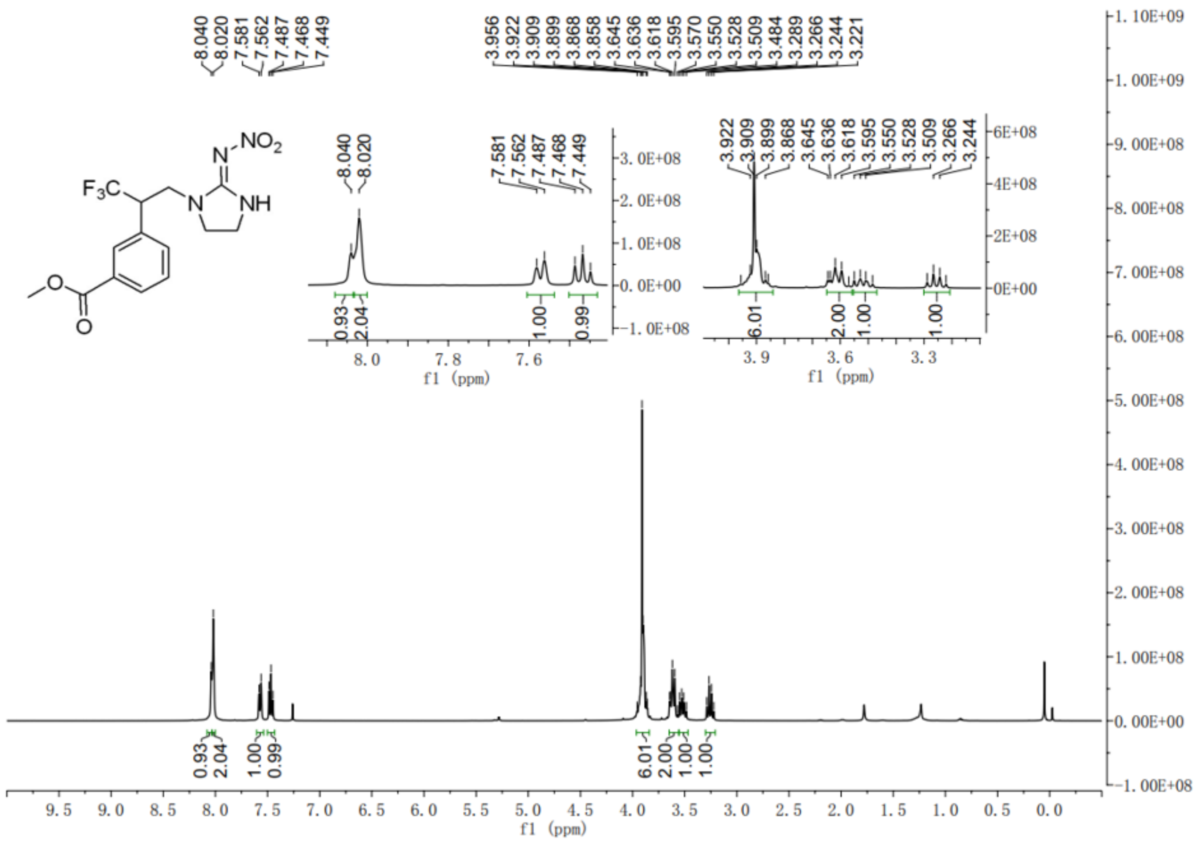

^{13}C NMR spectrum of 3ma (100 MHz, CDCl$_3$)

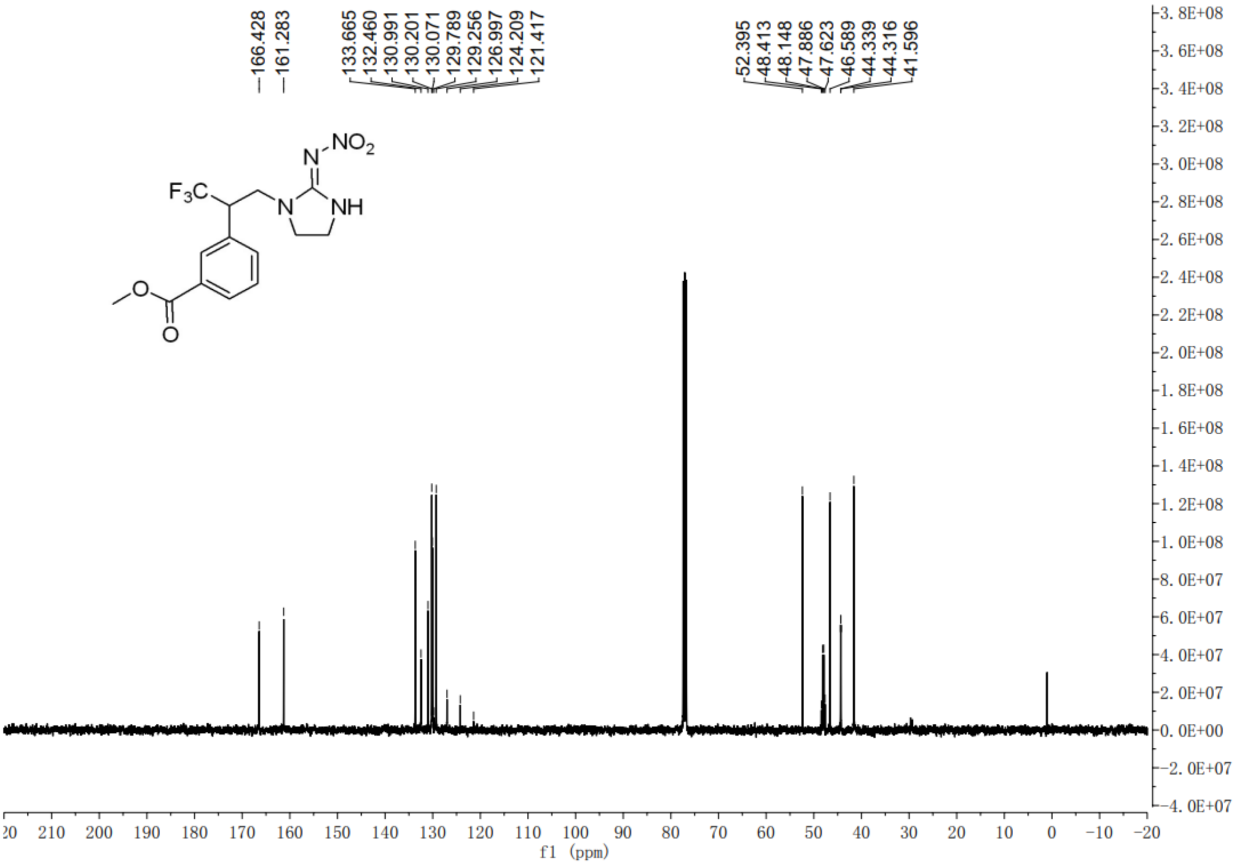

^{19}F NMR spectrum of 3ma (564 MHz, CDCl$_3$)

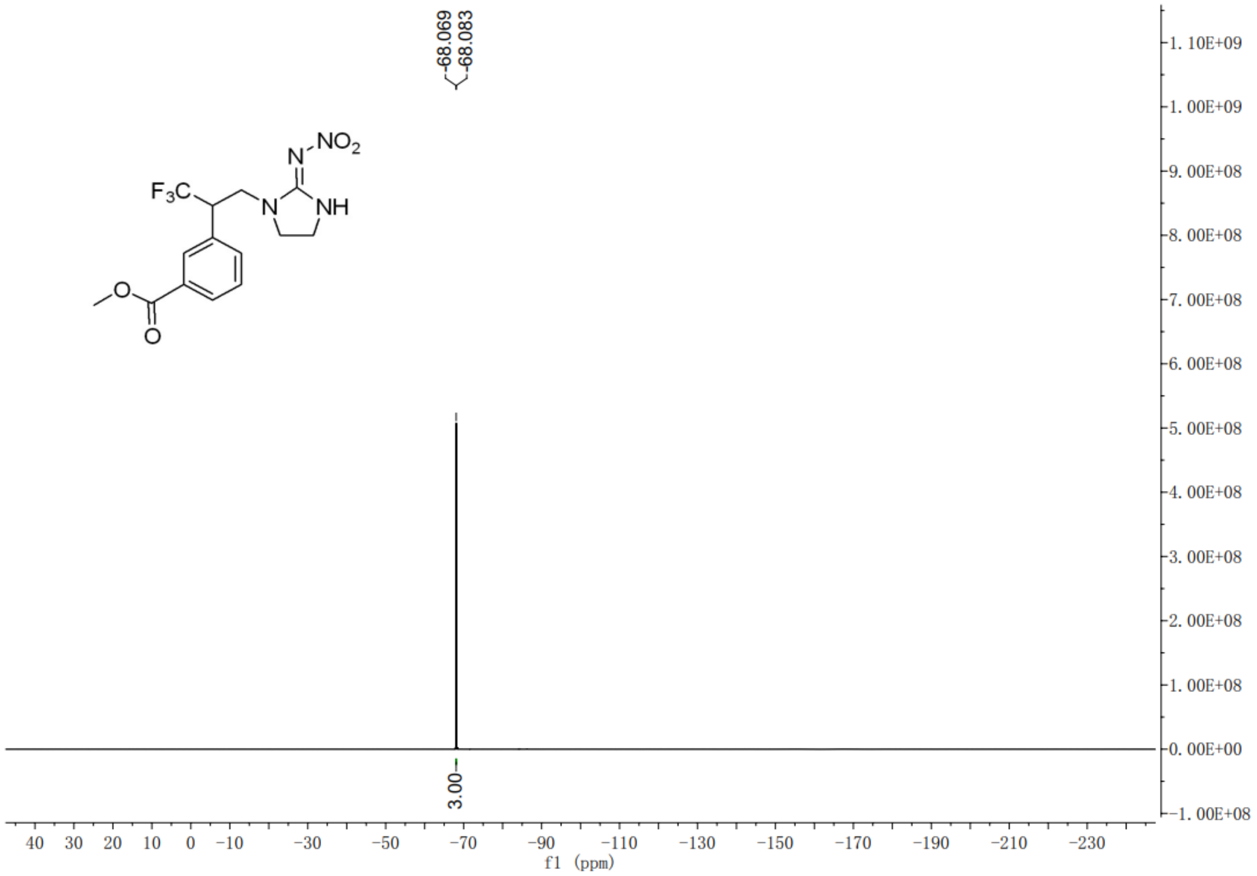

HRMS (EI) spectrum of 3ma

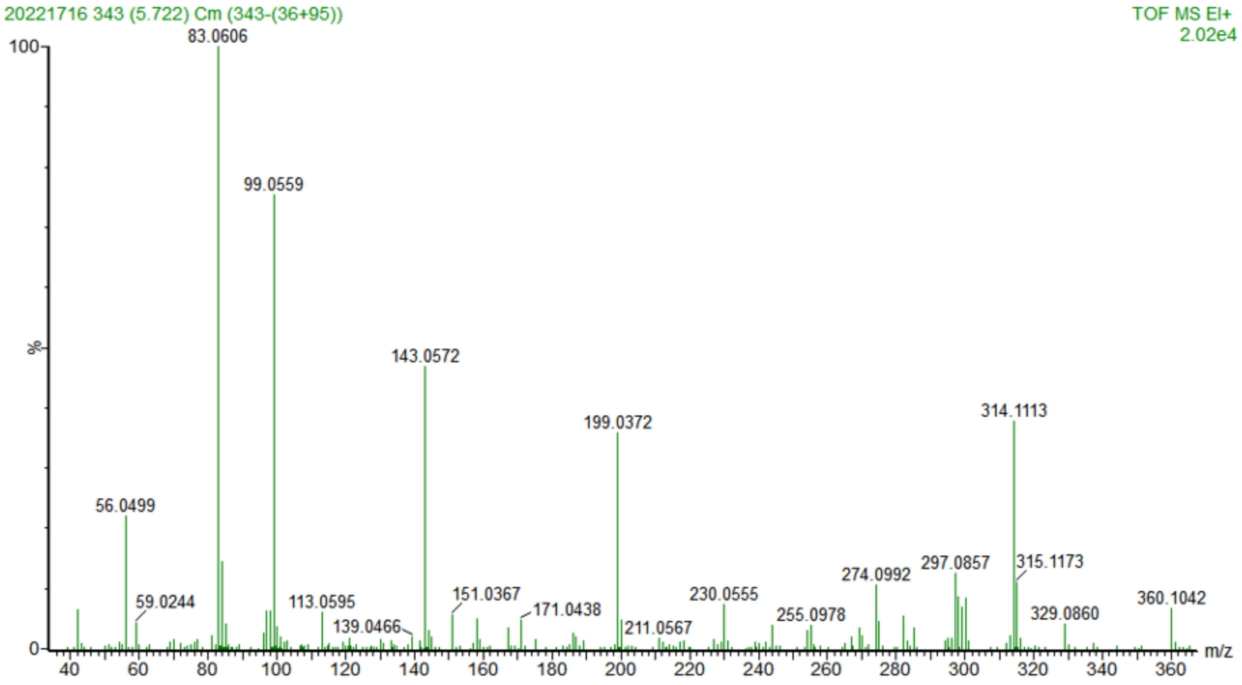

^{1}H NMR spectrum of 3na (400 MHz, $CDCl_3$)

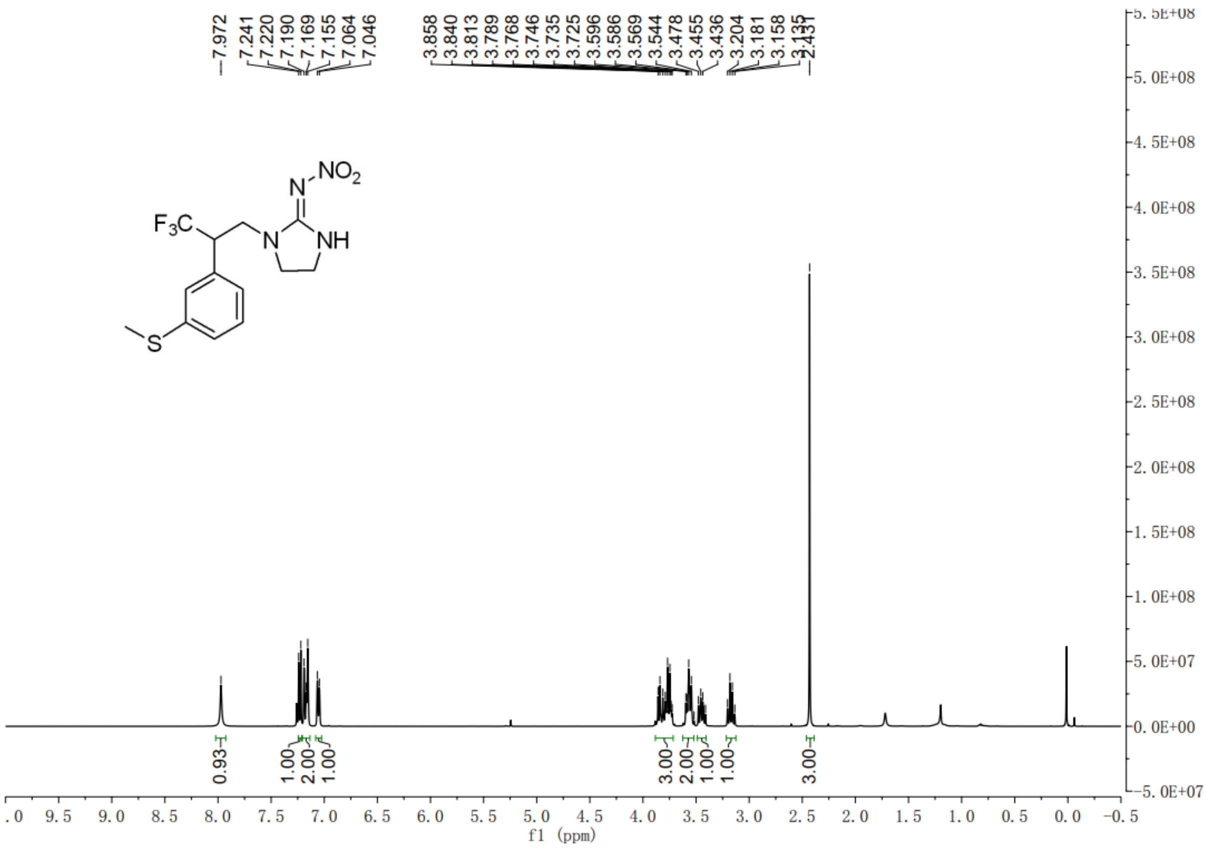

^{13}C NMR spectrum of 3na (100 MHz, $CDCl_3$)

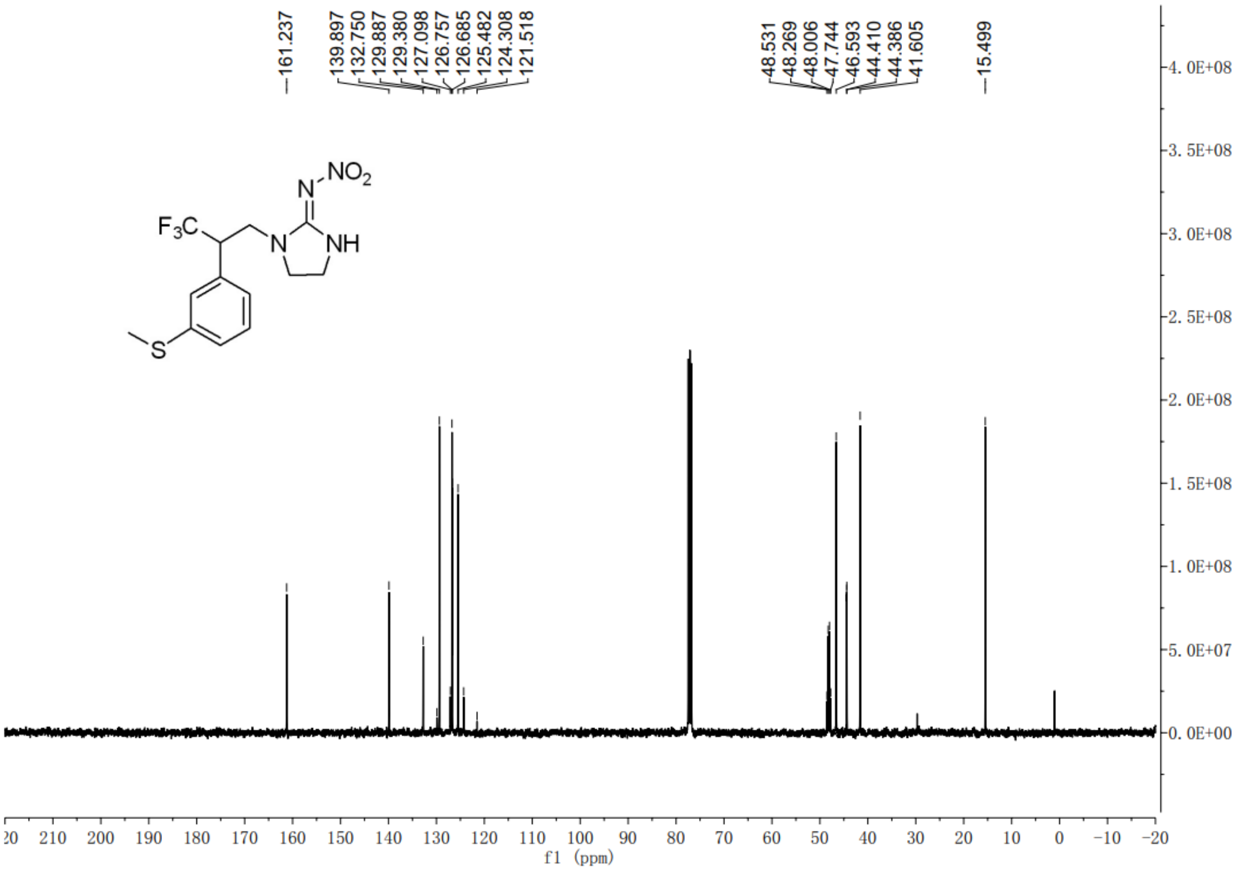

^{19}F NMR spectrum of 3na (564 MHz, $CDCl_3$)

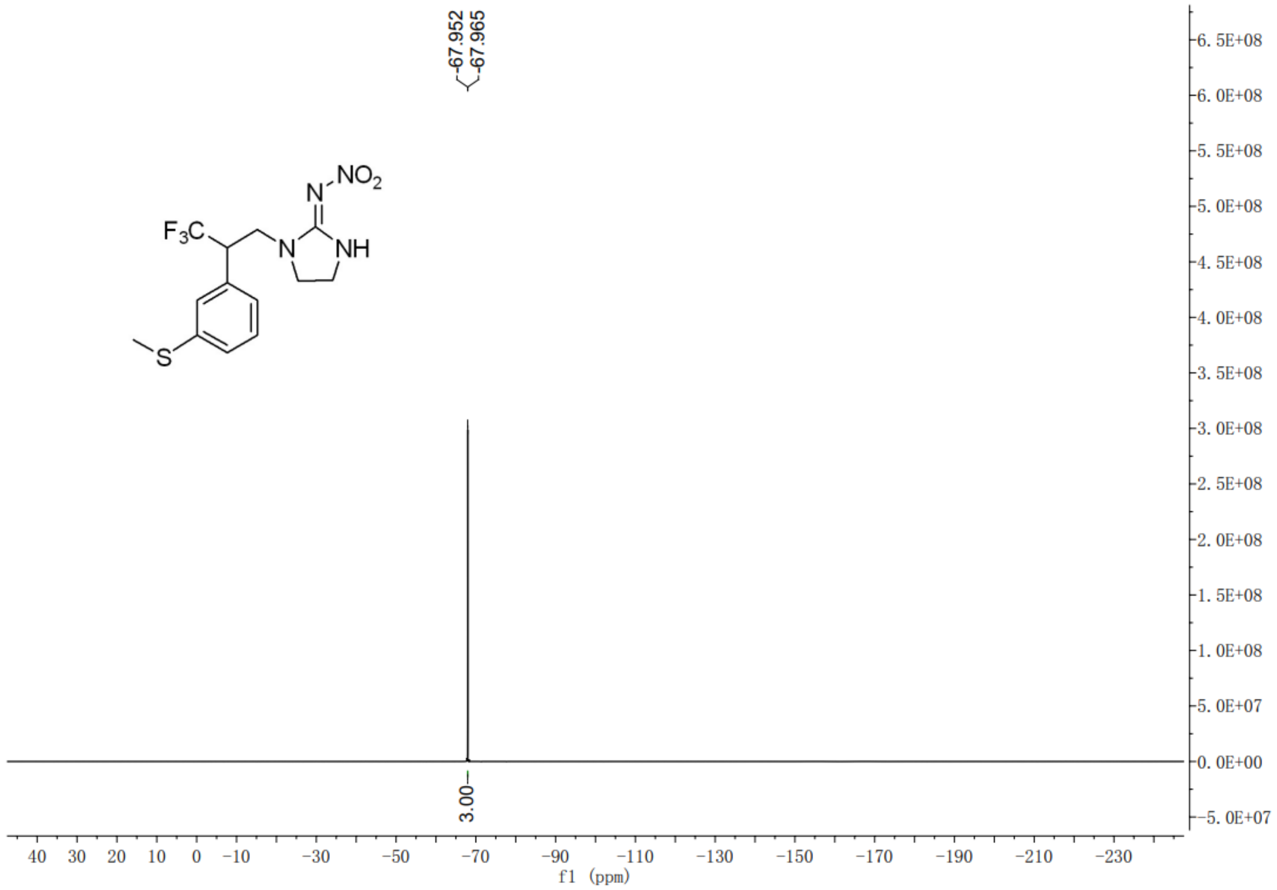

HRMS (EI) spectrum of 3na

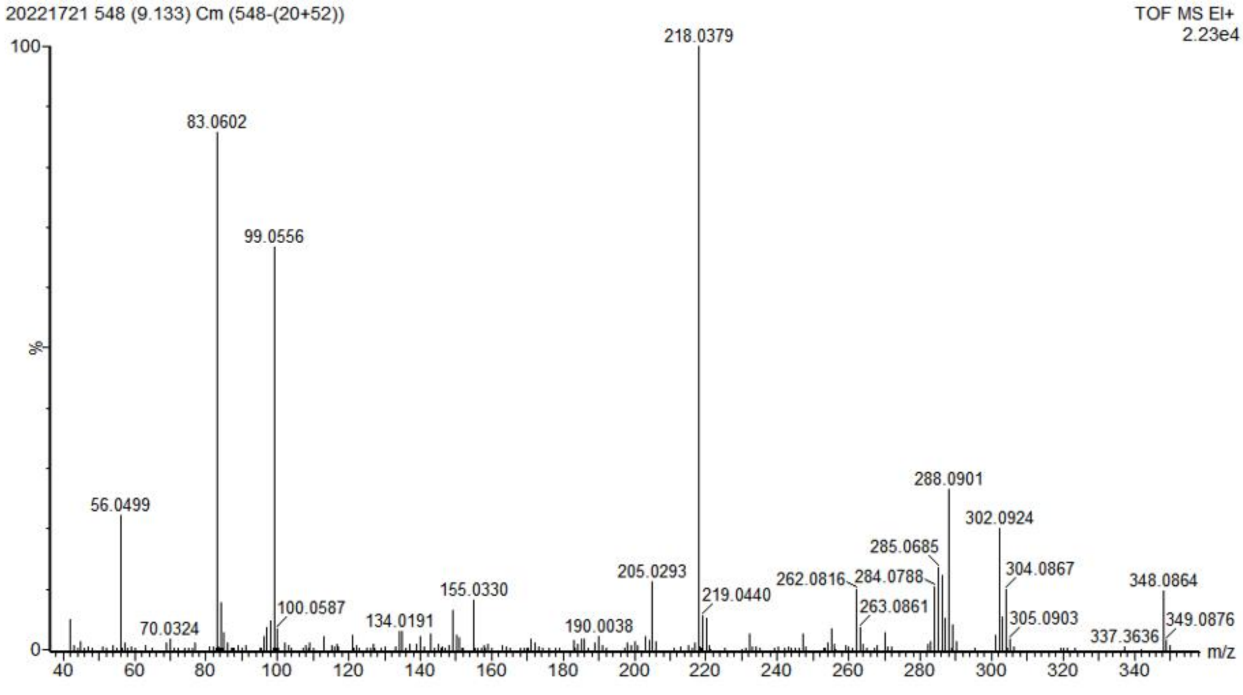

^{1}H NMR spectrum of 3qa (400 MHz, $CDCl_3$)

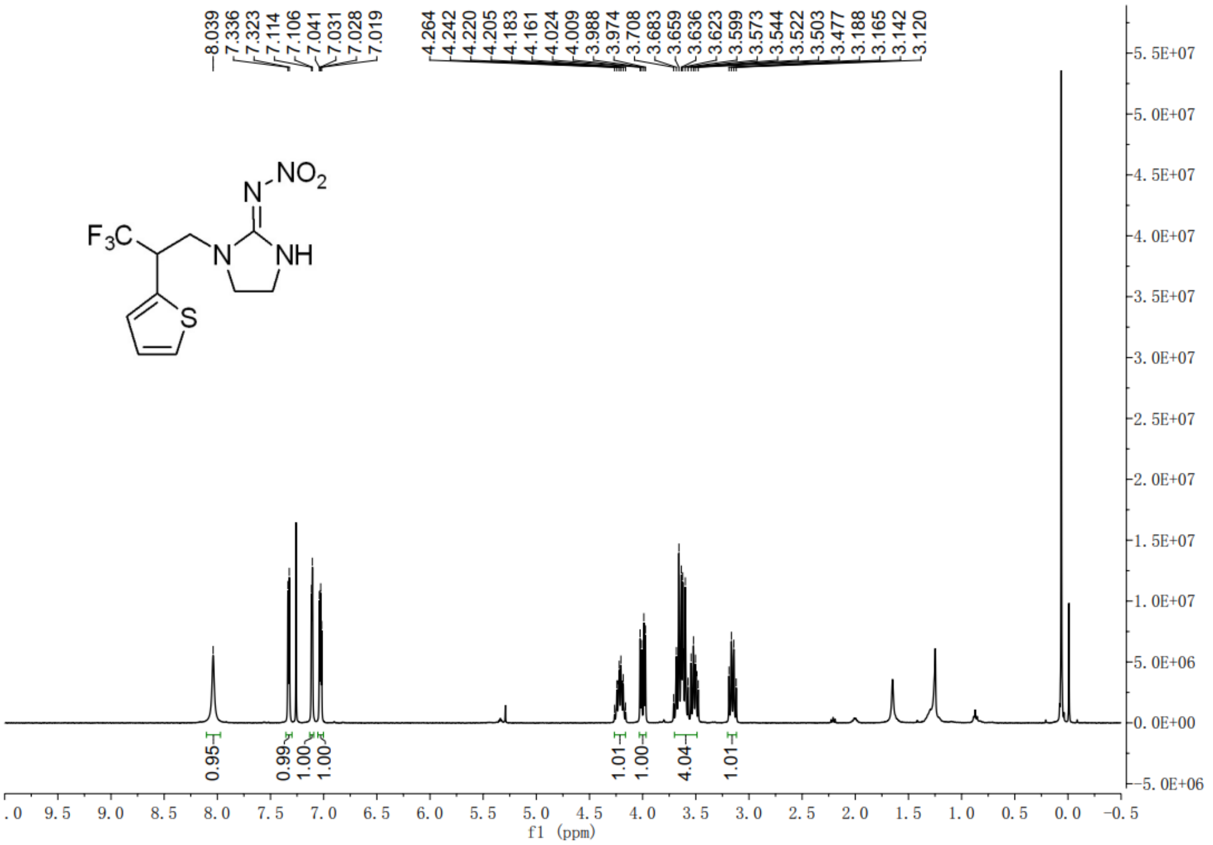

^{13}C NMR spectrum of 3qa (100 MHz, $CDCl_3$)

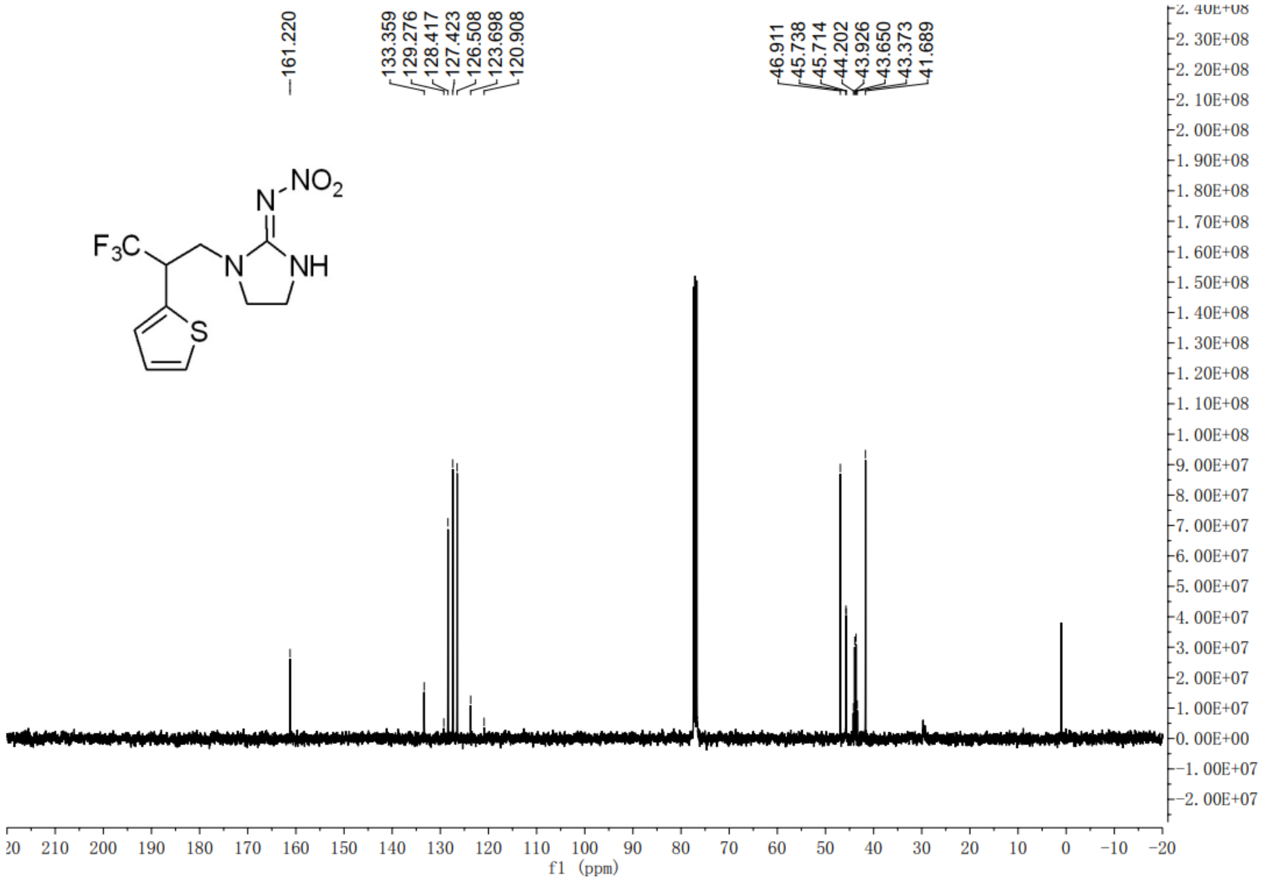

^{19}F NMR spectrum of 3qa (564 MHz, $CDCl_3$)

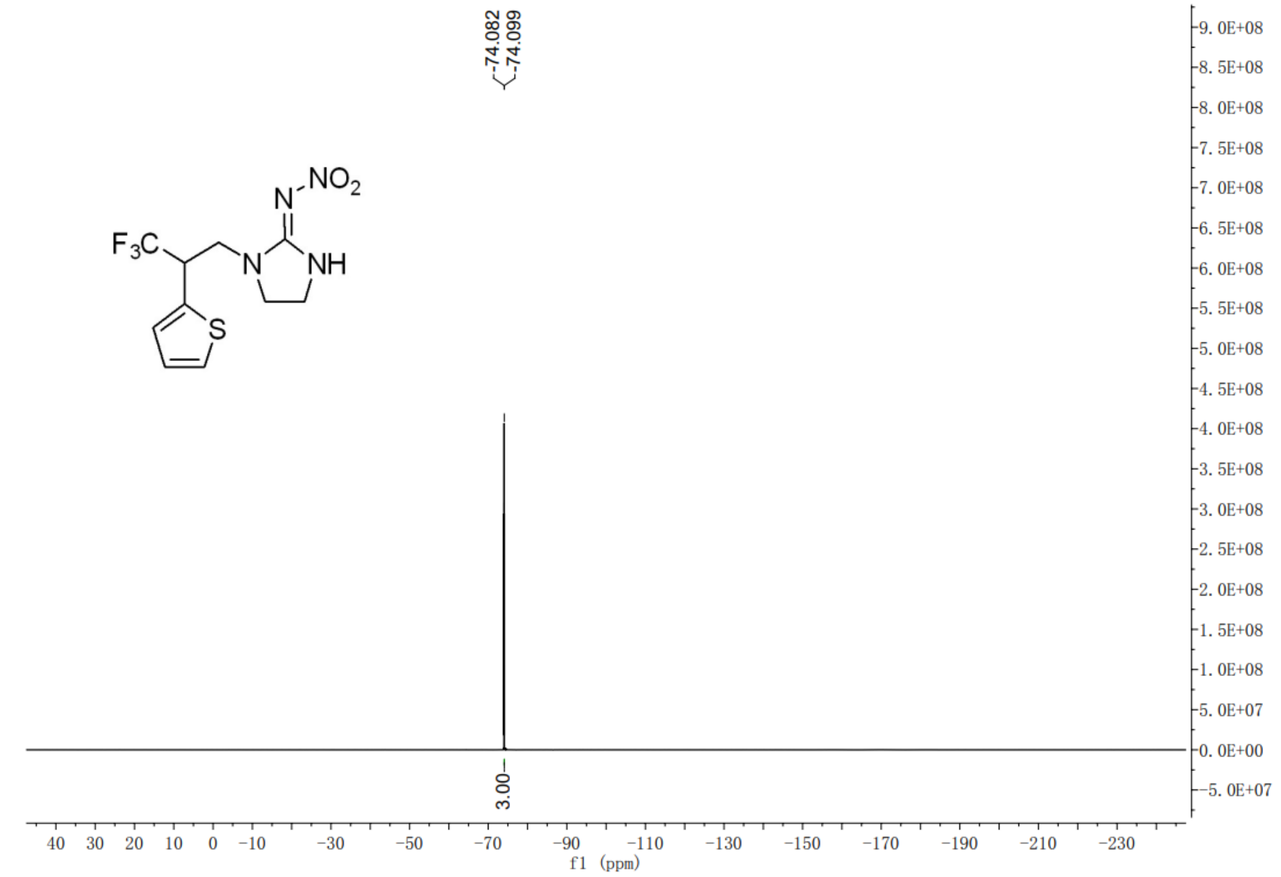

HRMS (EI) spectrum of 3qa

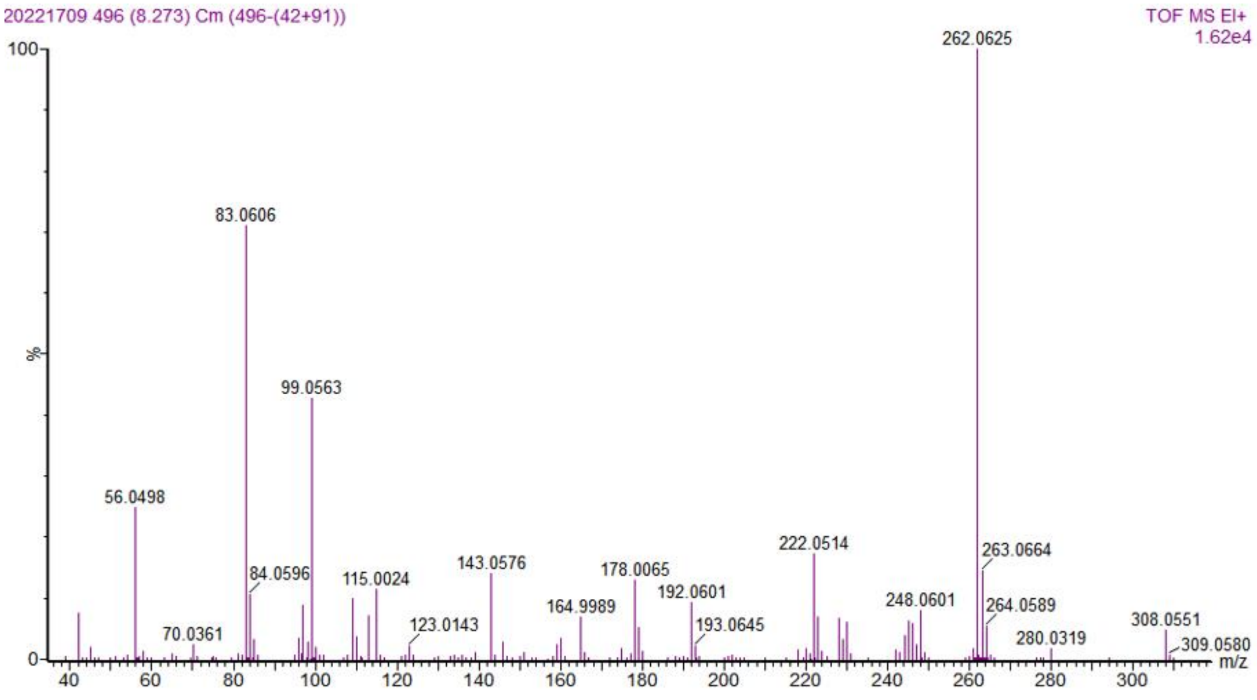

^{1}H NMR spectrum of 3ra (400 MHz, $CDCl_3$)

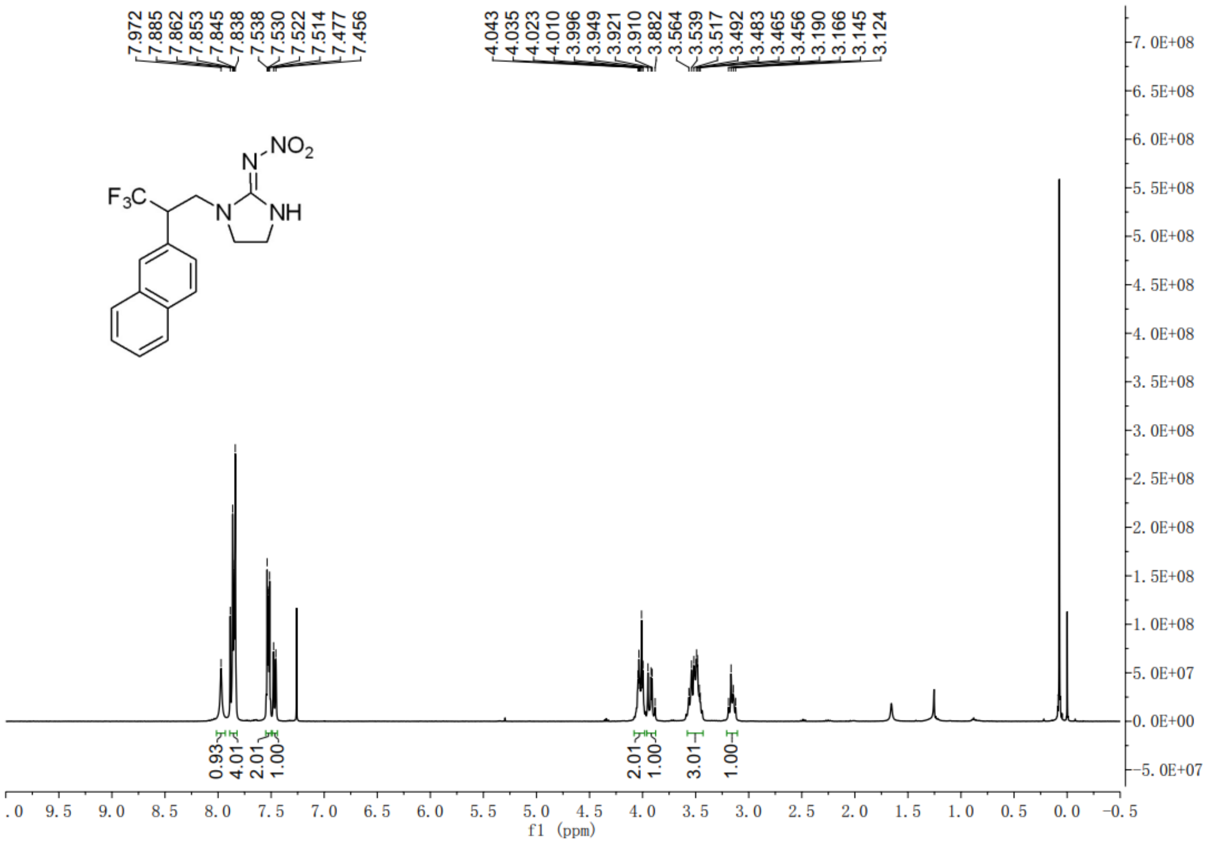

^{13}C NMR spectrum of 3ra (100 MHz, $CDCl_3$)

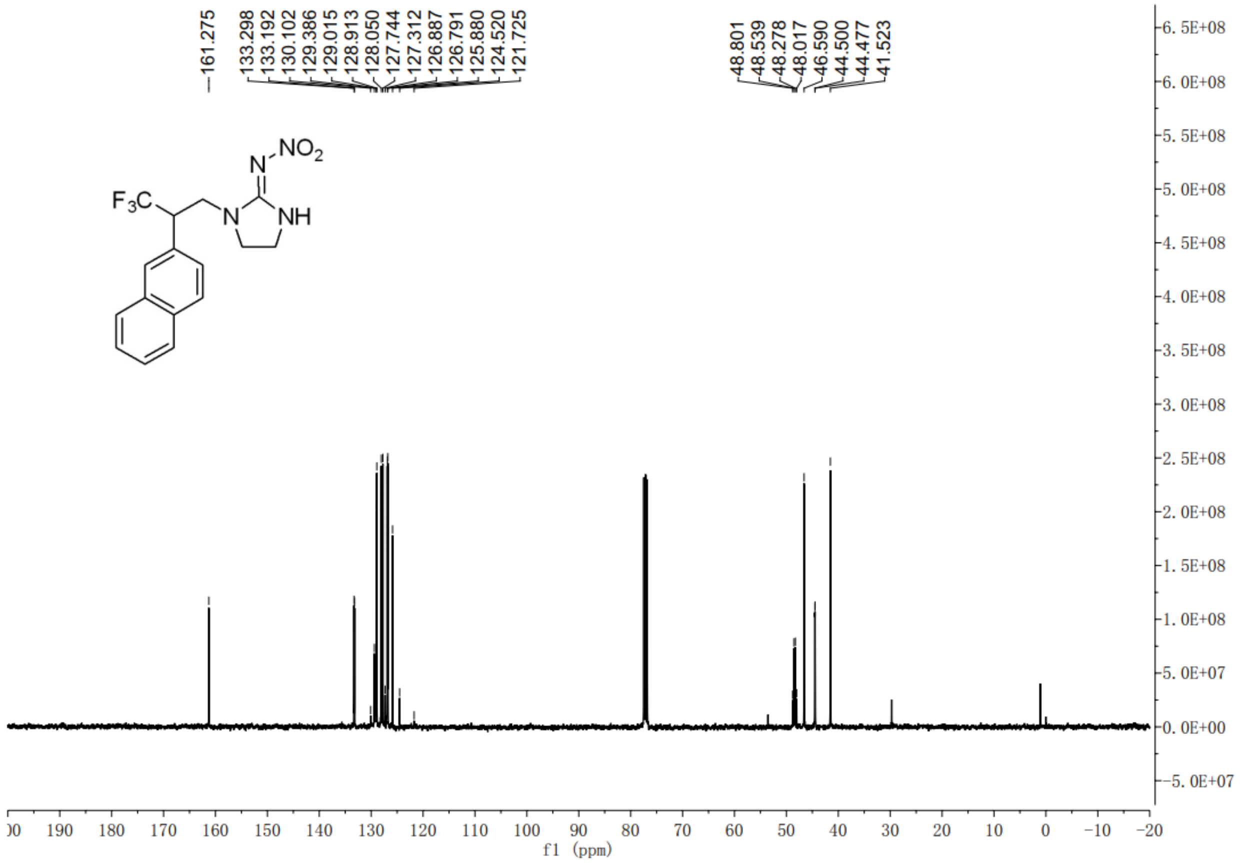

^{19}F NMR spectrum of 3ra (564 MHz, CDCl$_3$)

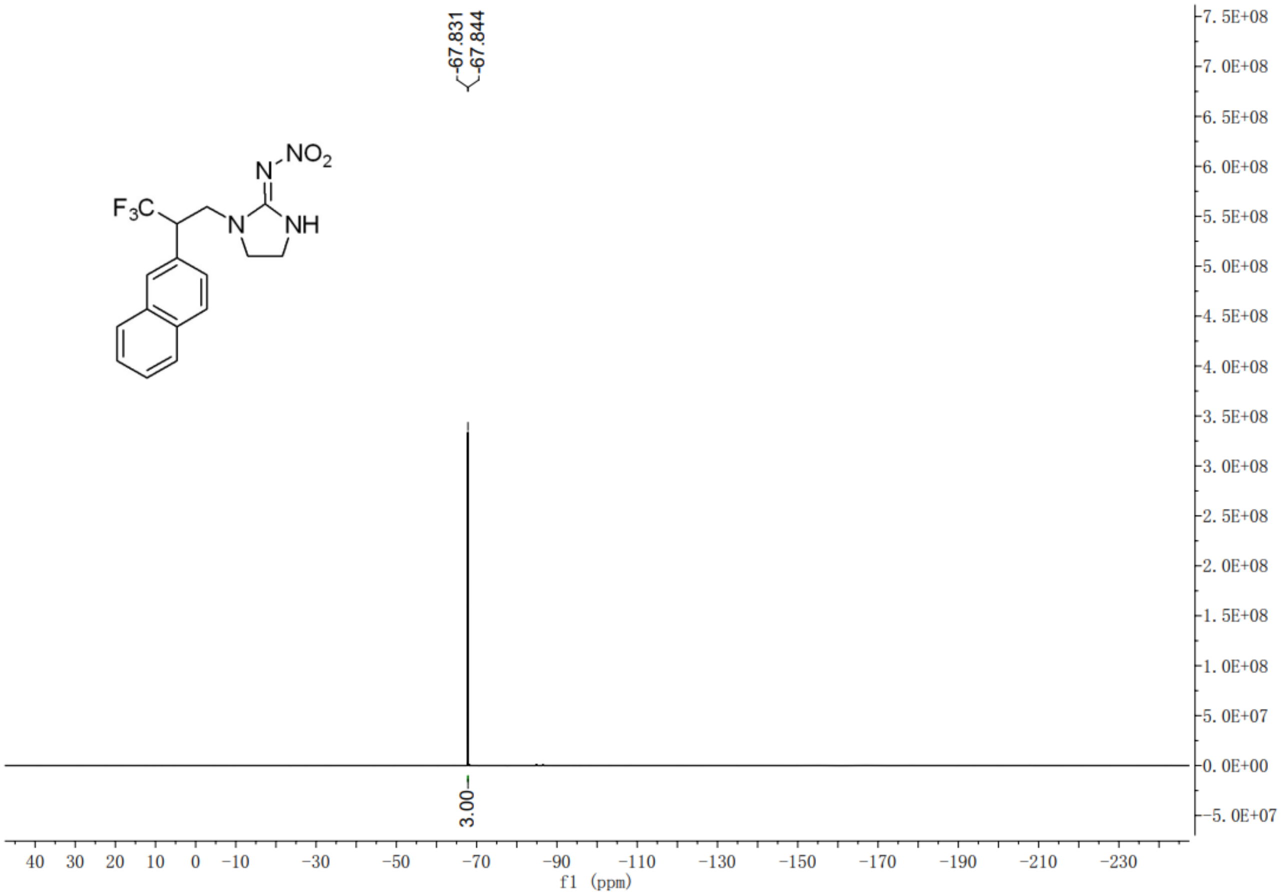

HRMS (EI) spectrum of 3ra

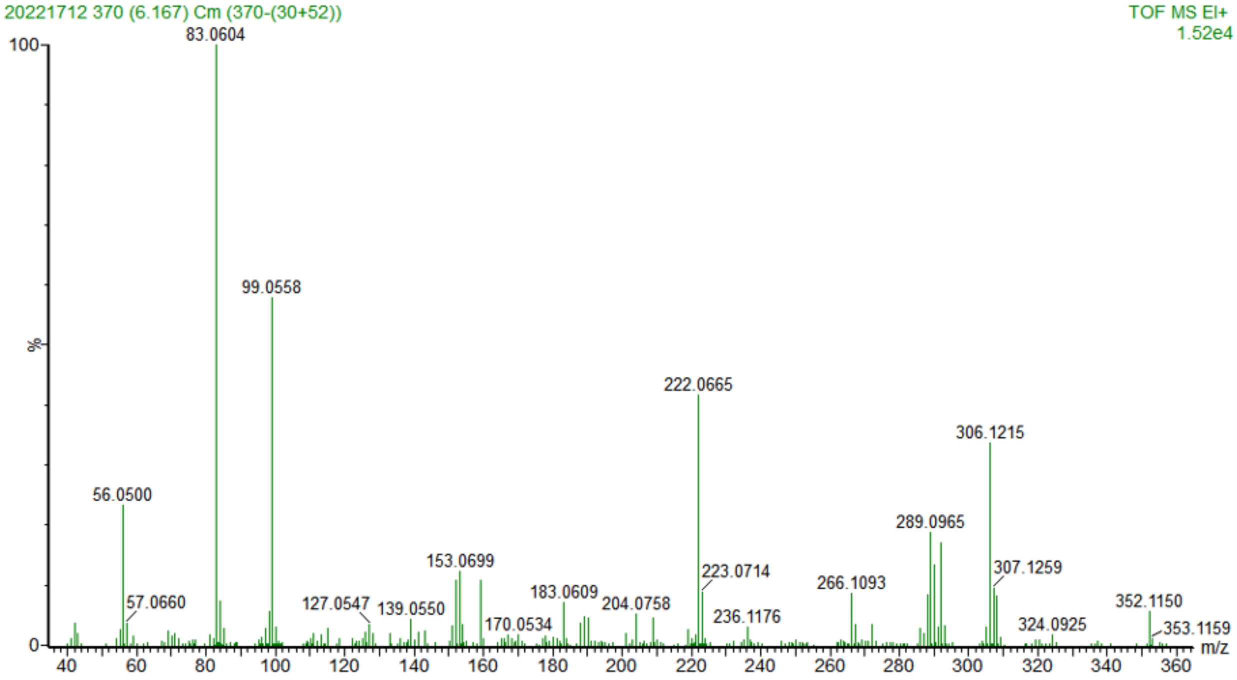

^{1}H NMR spectrum of 3sa (400 MHz, acetone-d_6)

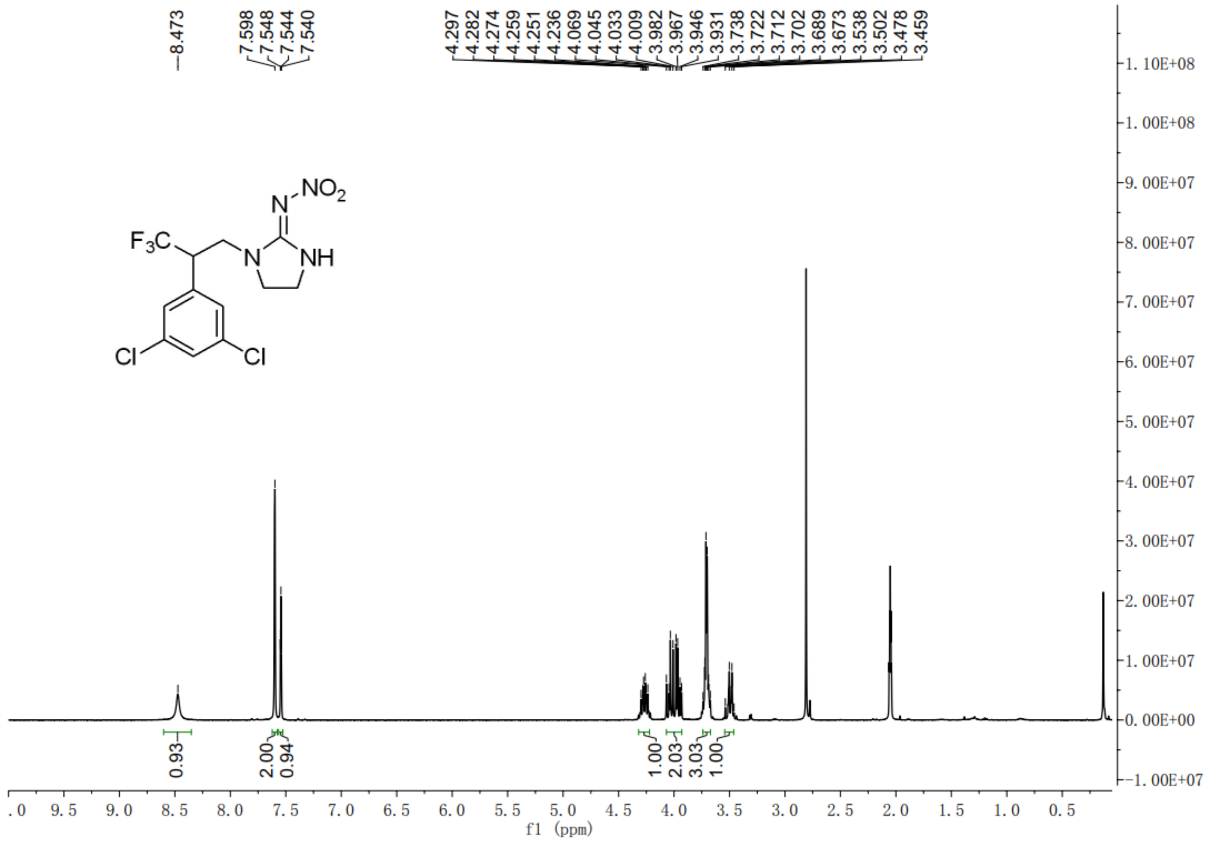

^{13}C NMR spectrum of 3sa (100 MHz, acetone-d_6)

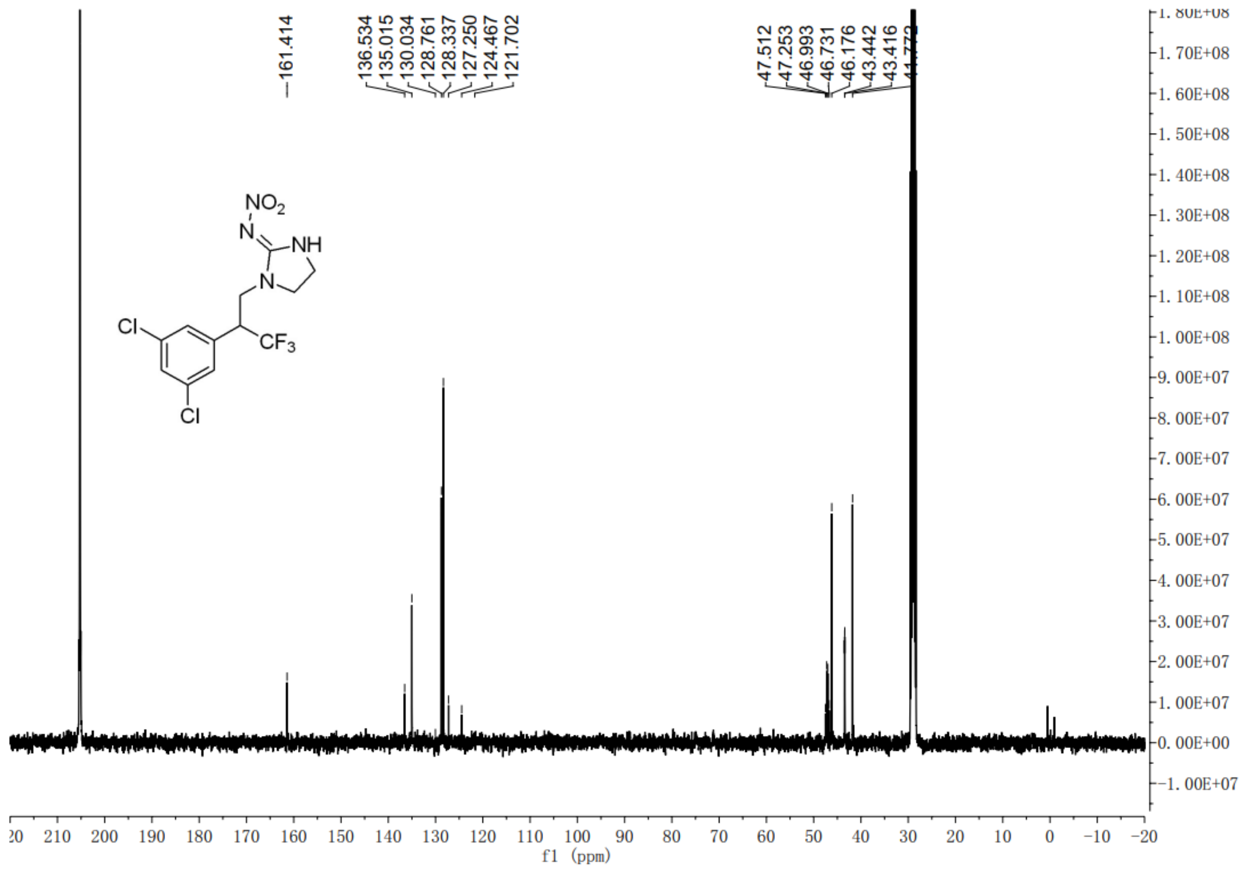

^{19}F NMR spectrum of 3sa (564 MHz, acetone-d_6)

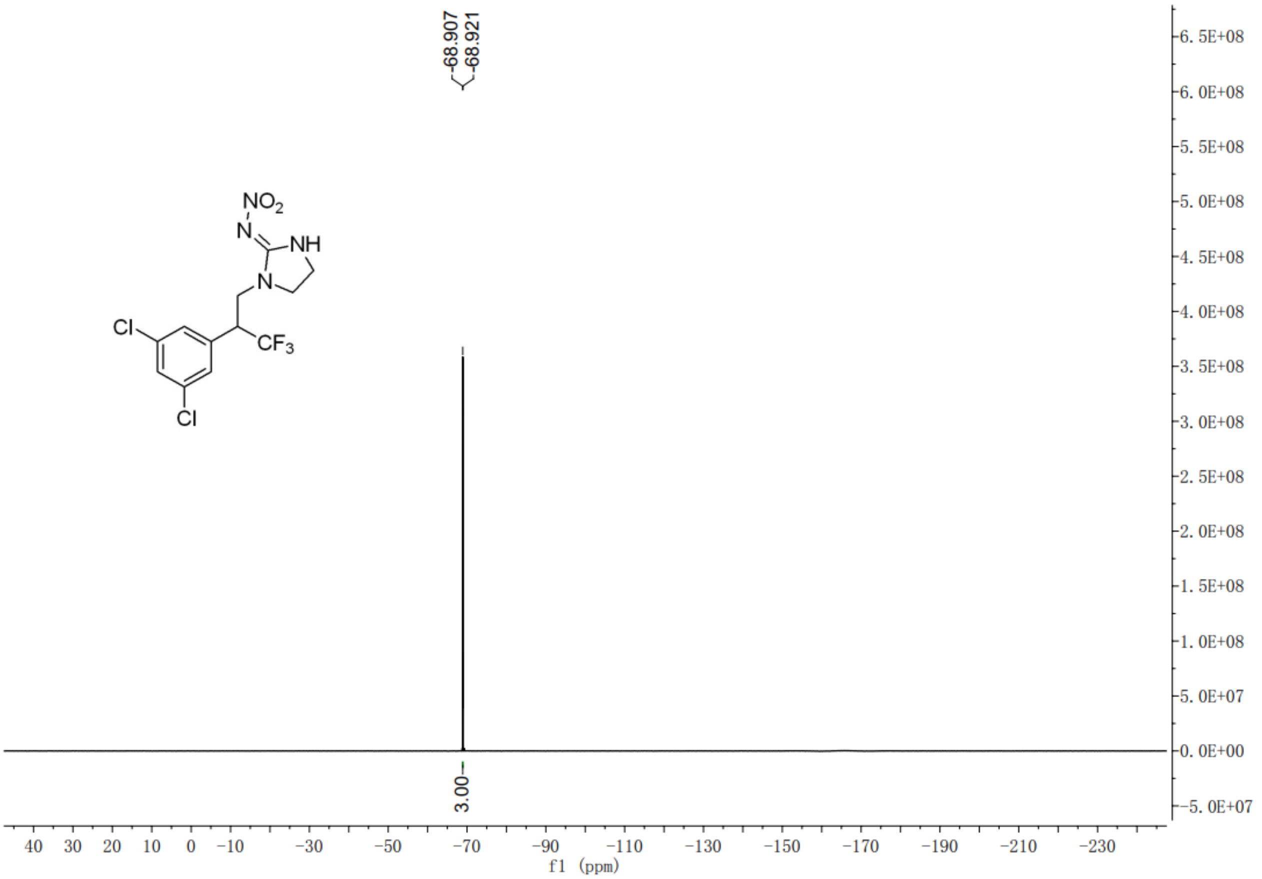

HRMS (EI) spectrum of 3sa

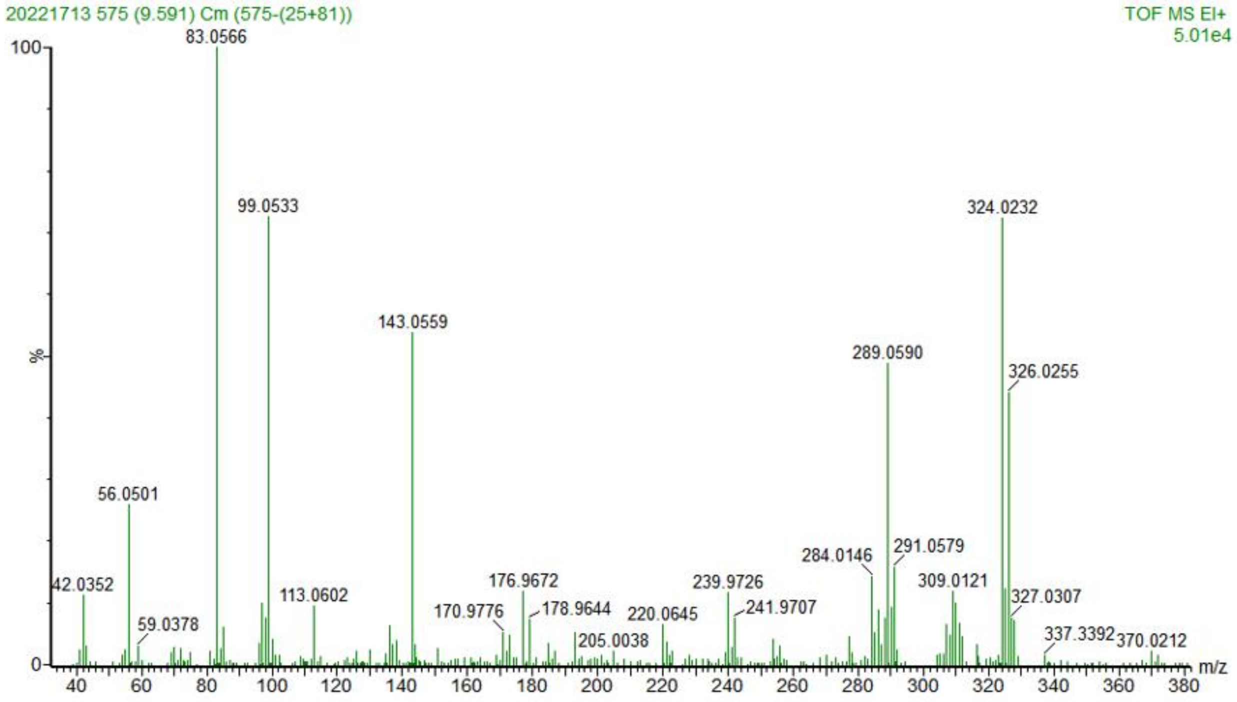

^{1}H NMR spectrum of 3ta (400 MHz, $CDCl_3$)

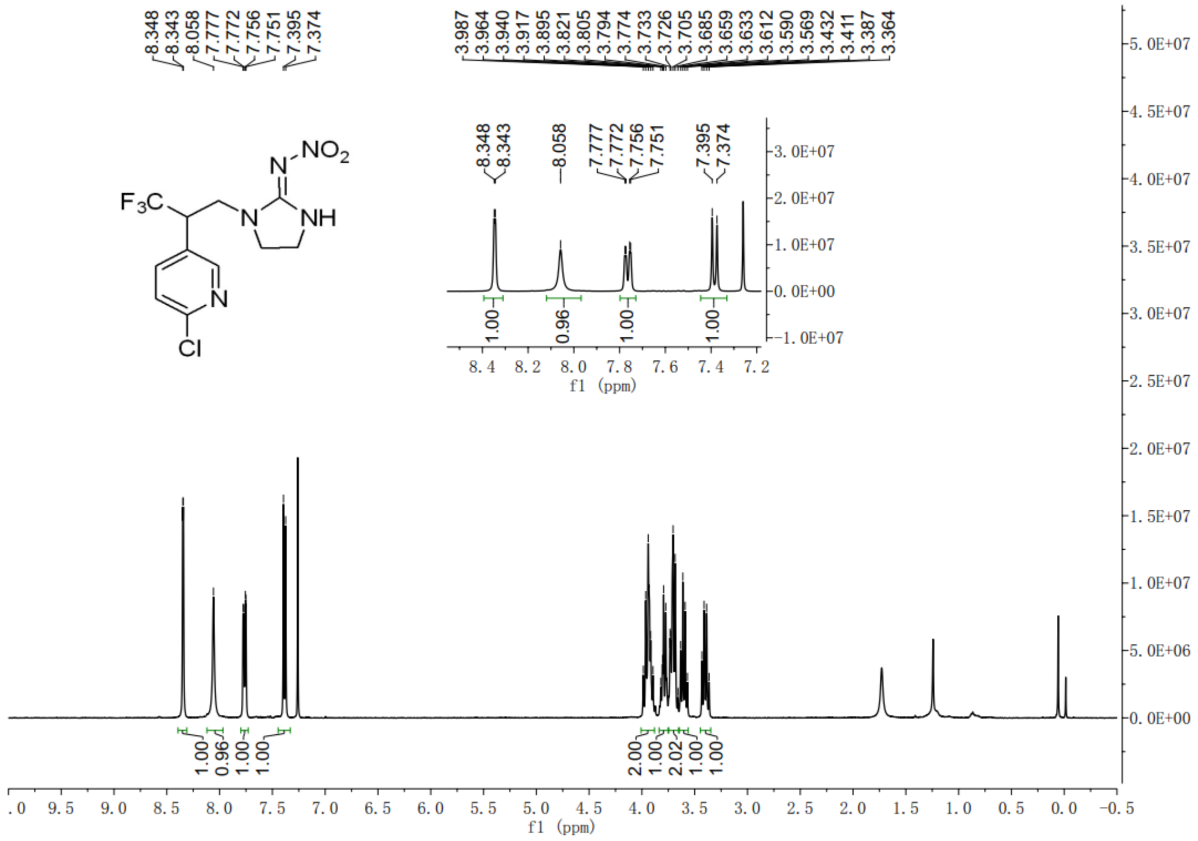

^{13}C NMR spectrum of 3ta (100 MHz, $CDCl_3$)

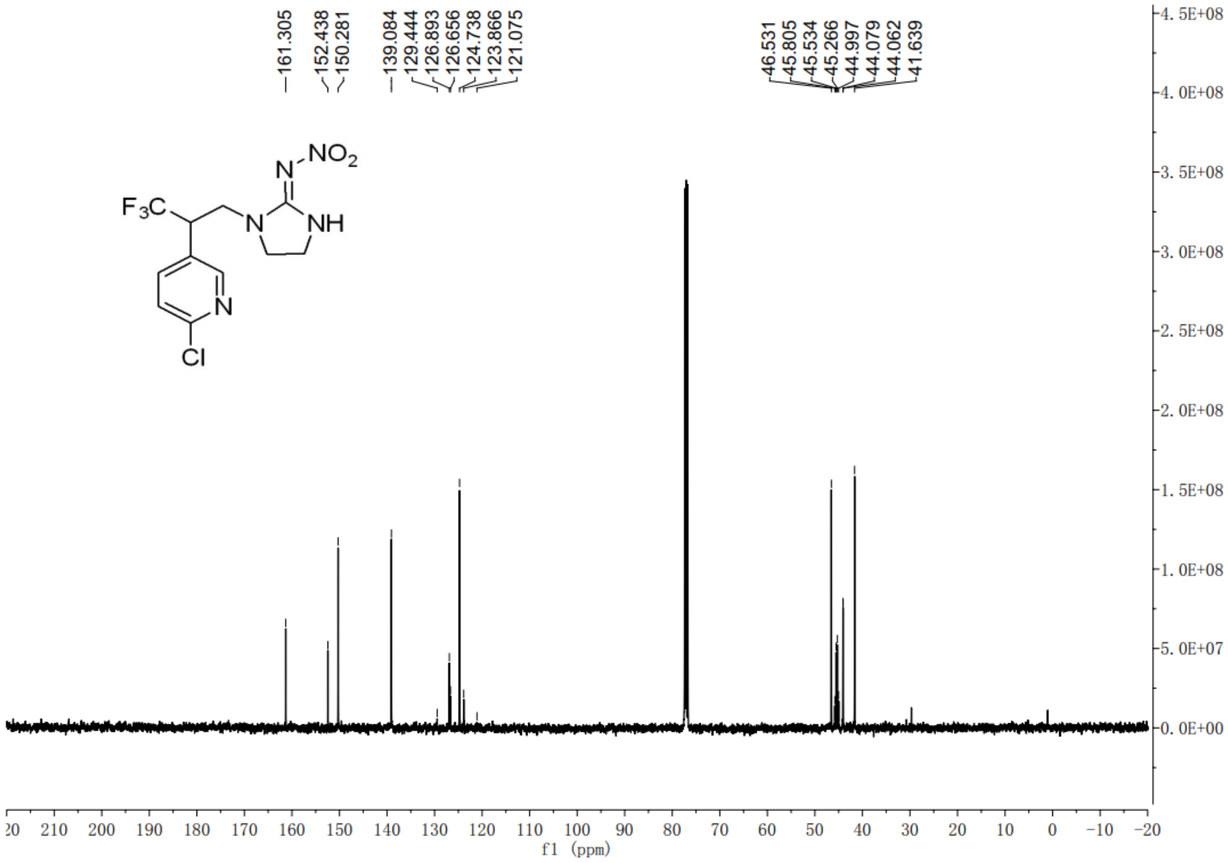

^{19}F NMR spectrum of 3ta (564 MHz, $CDCl_3$)

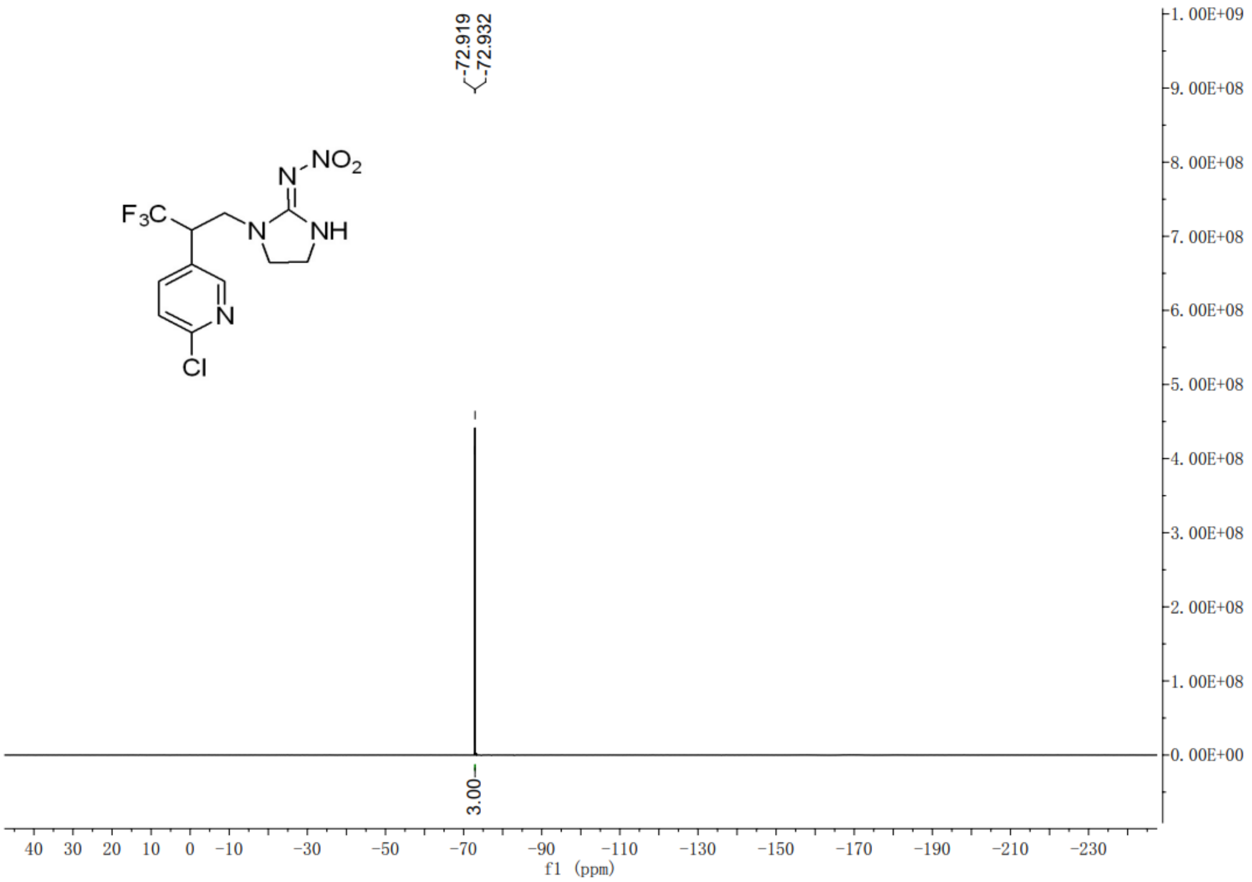

HRMS (EI) spectrum of 3ta

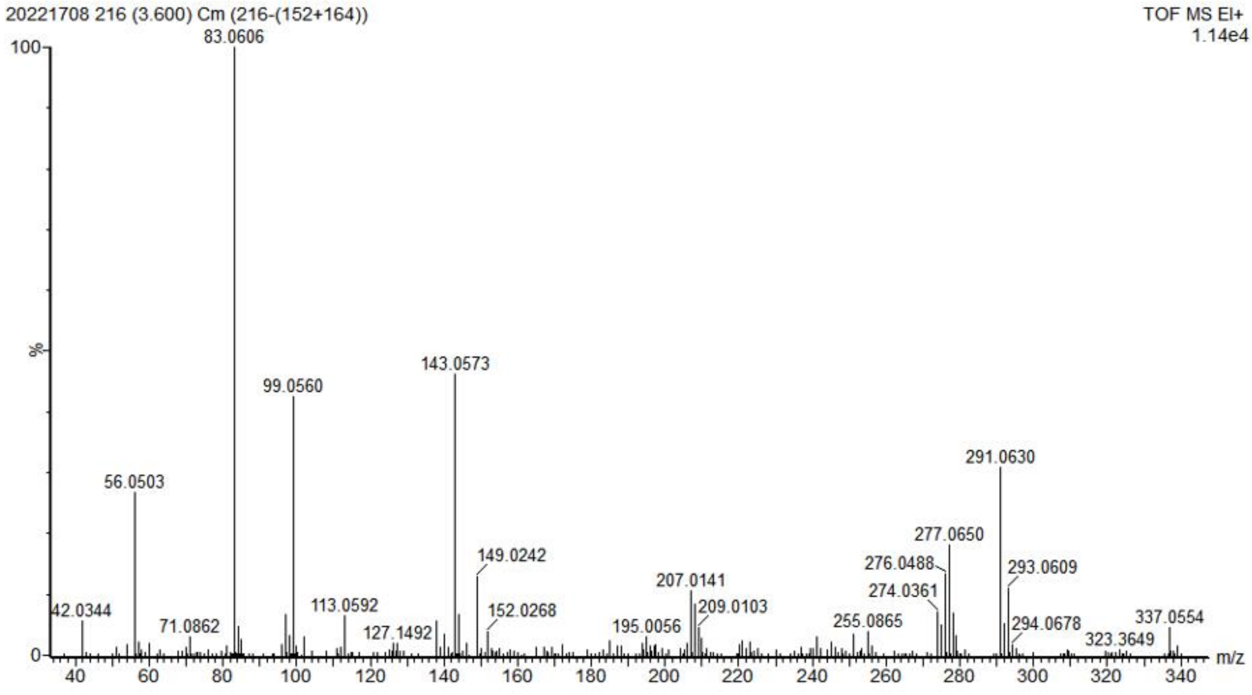

^{1}H NMR spectrum of 3cb (400 MHz, $CDCl_3$)

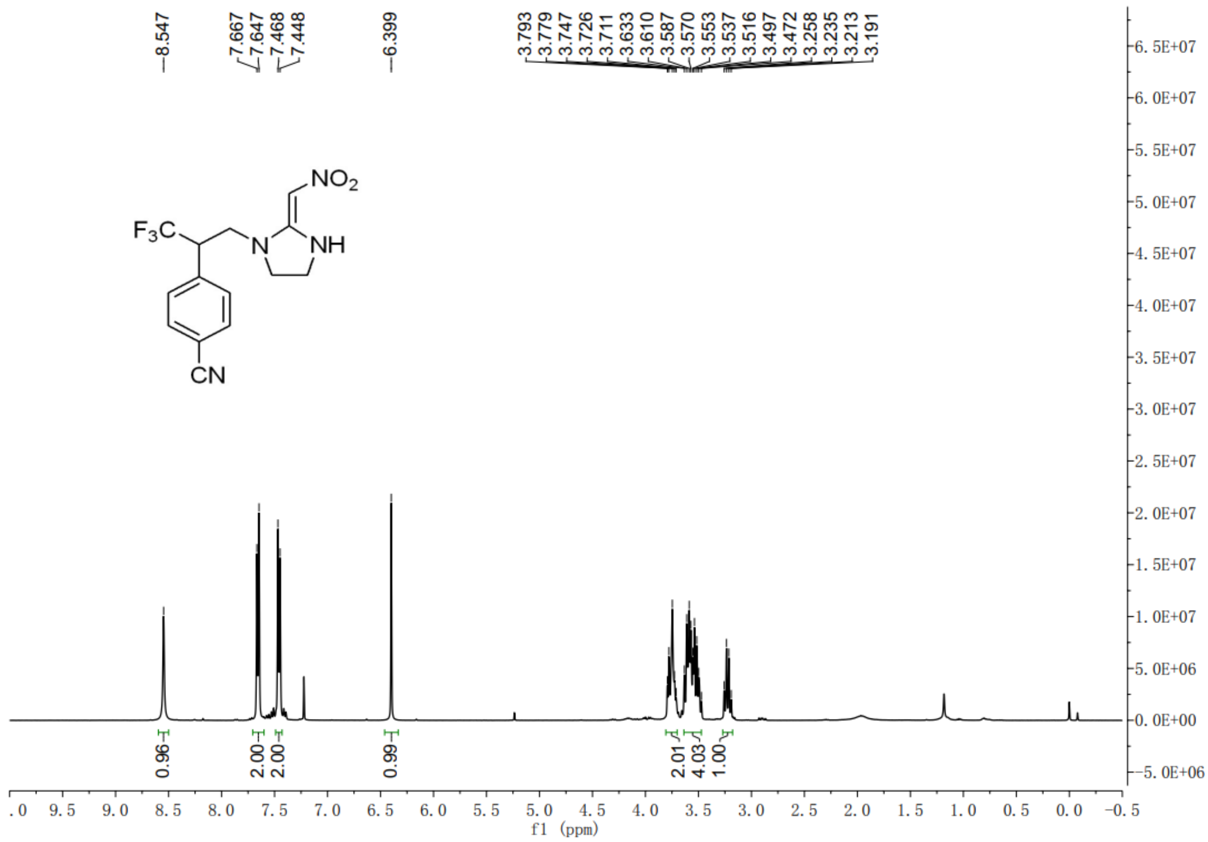

^{13}C NMR spectrum of 3cb (100 MHz, $CDCl_3$)

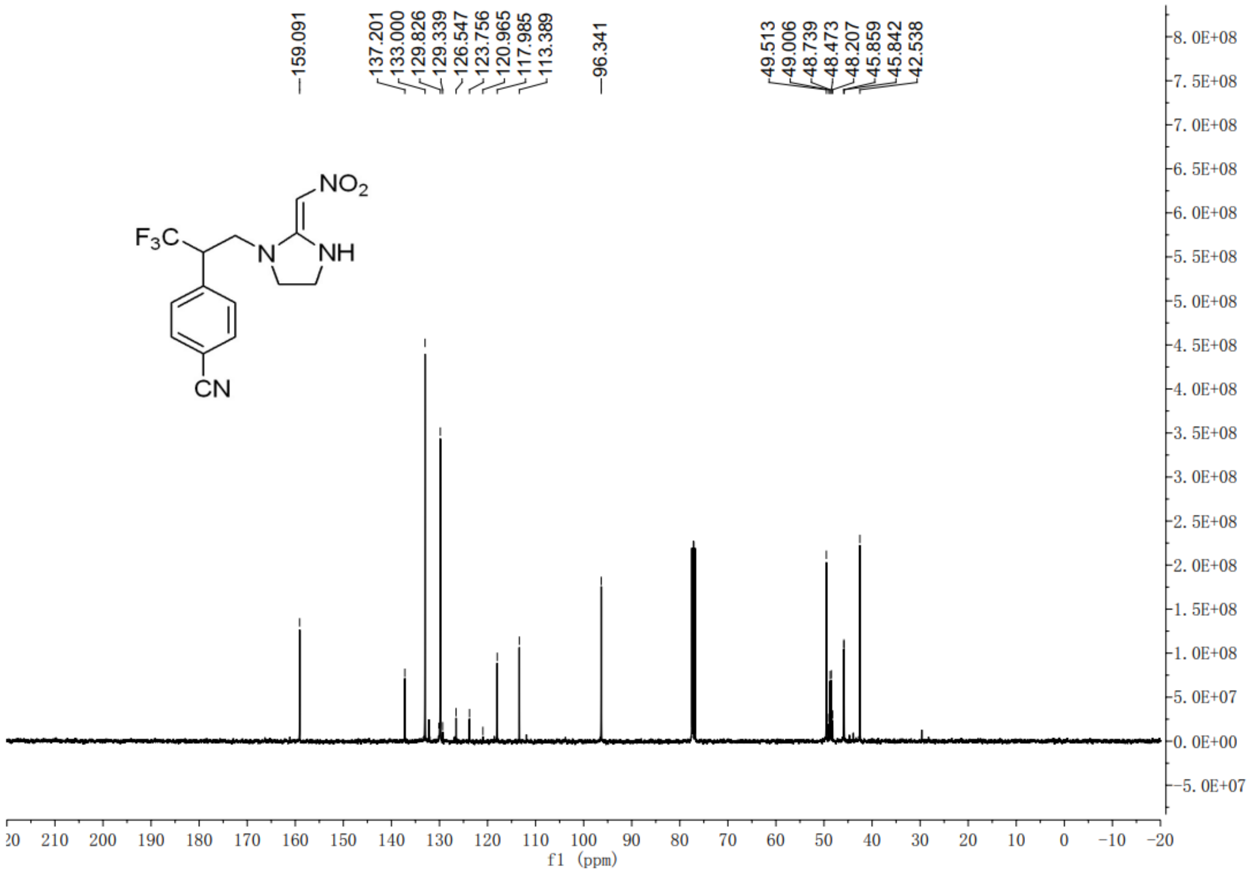

^{19}F NMR spectrum of 3cb (564 MHz, $CDCl_3$)

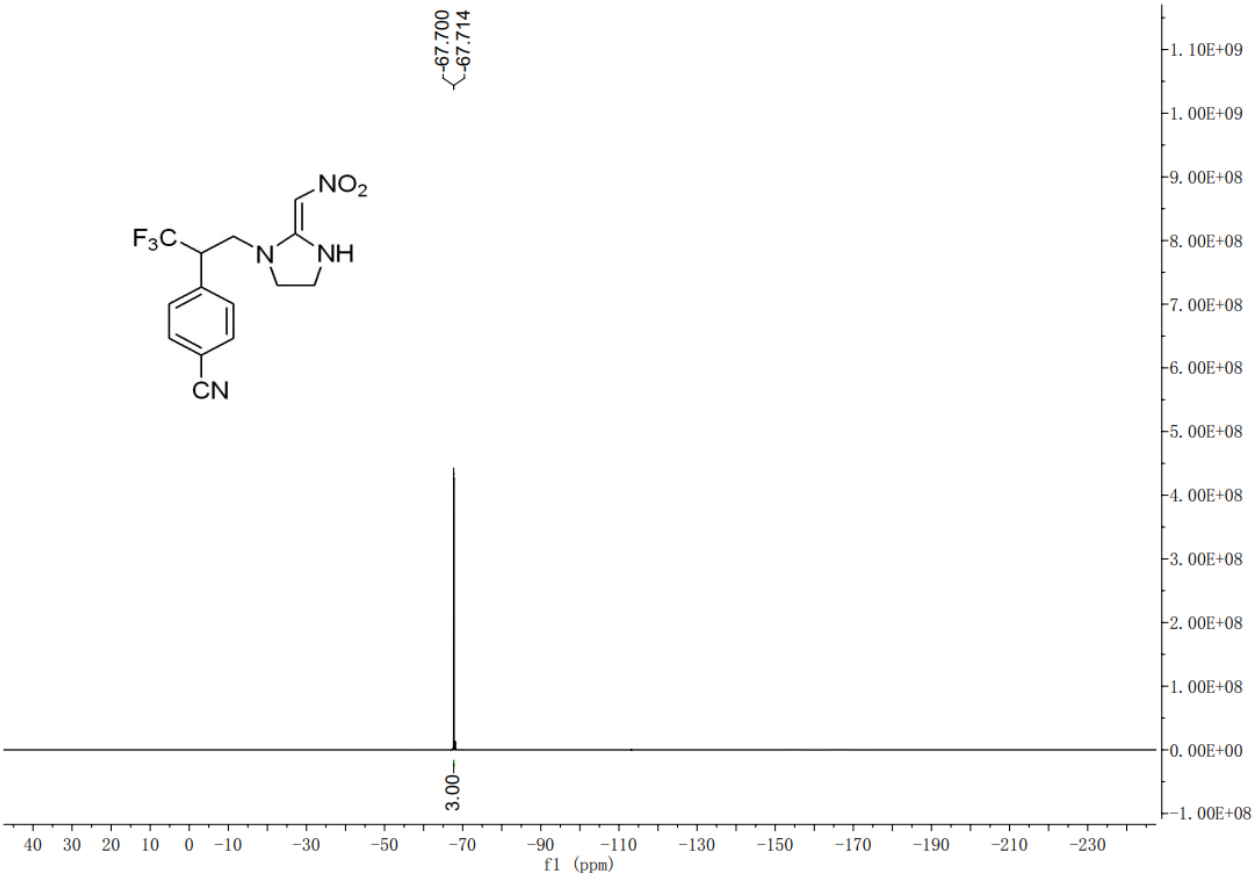

HRMS (EI) spectrum of 3cb

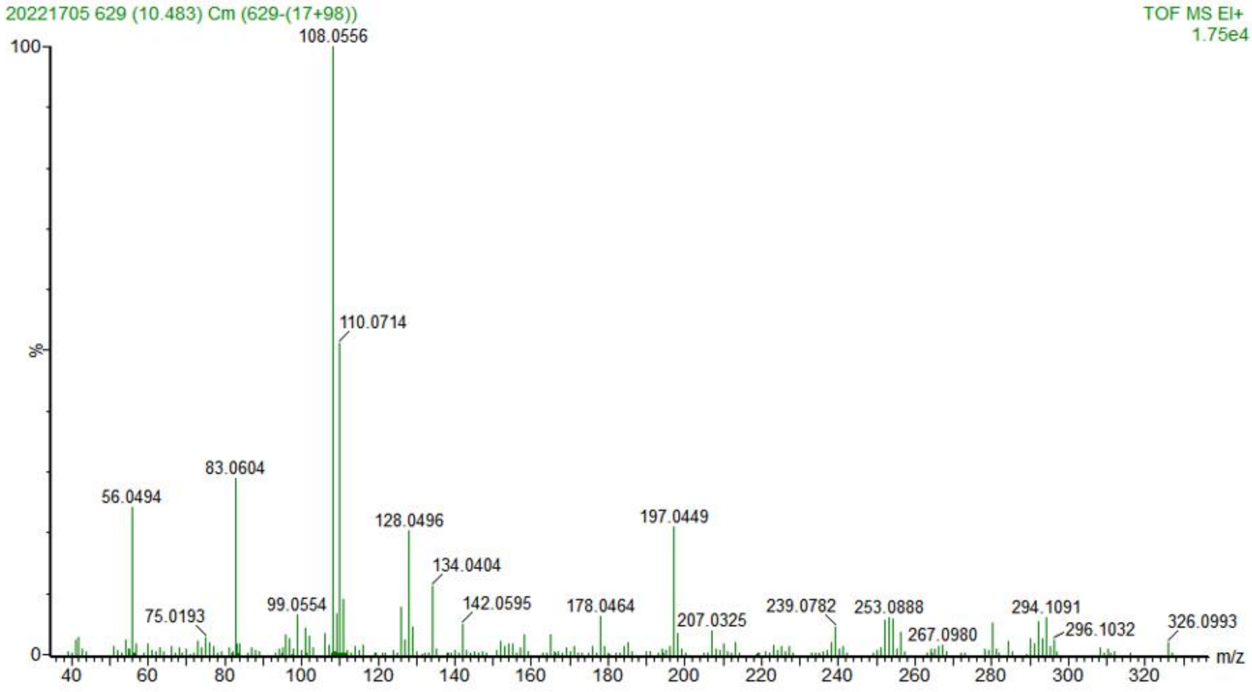

^{1}H NMR spectrum of 3ec (400 MHz, $CDCl_3$)

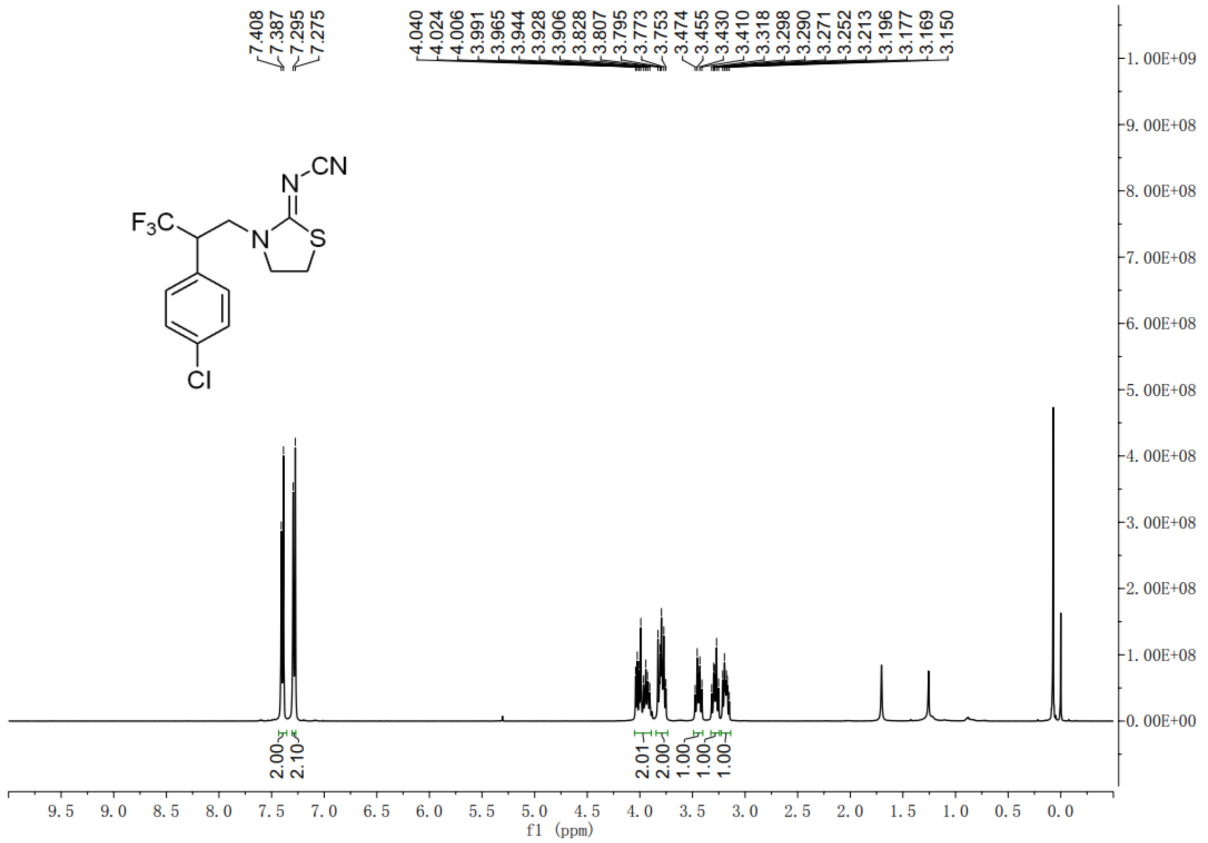

^{13}C NMR spectrum of 3ec (100 MHz, $CDCl_3$)

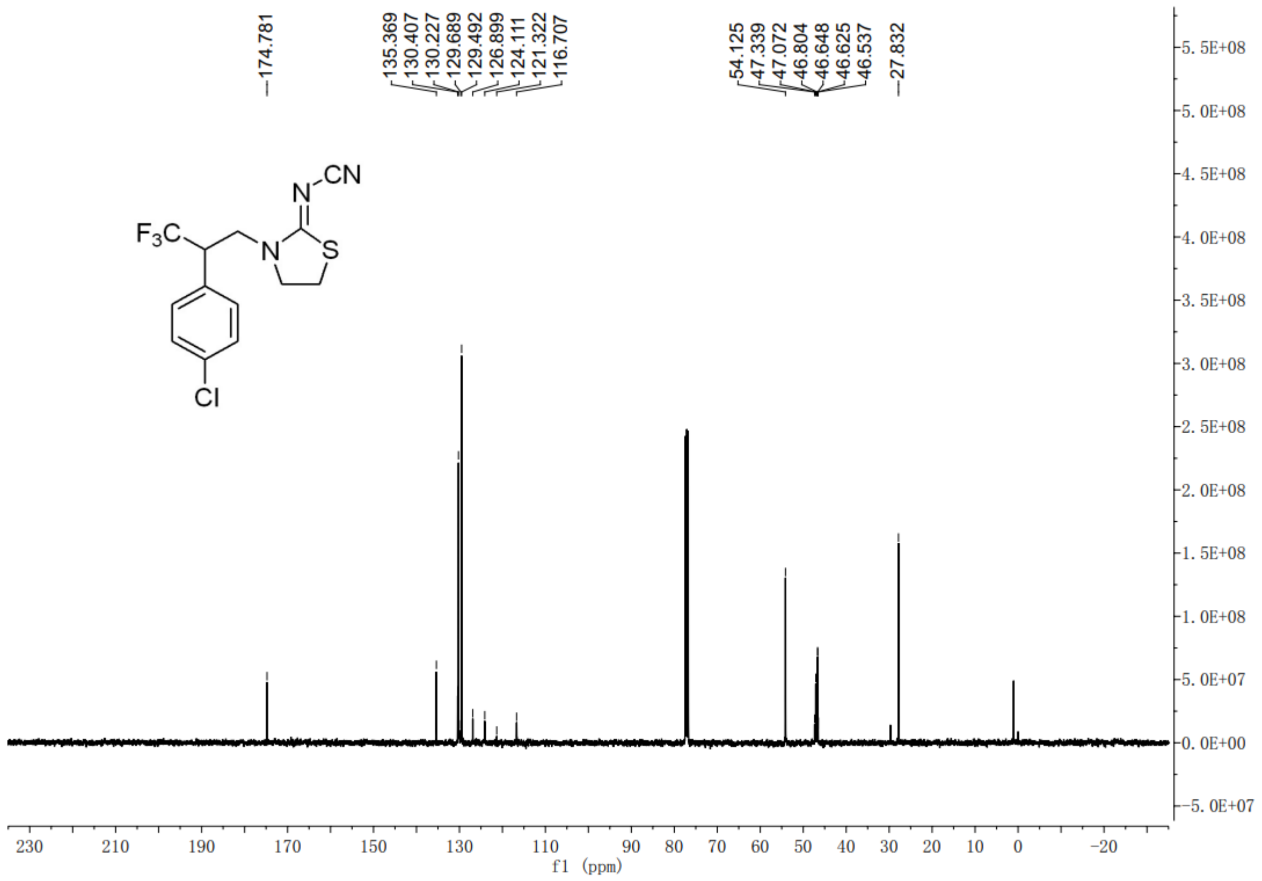

^{19}F NMR spectrum of 3ec (564 MHz, $CDCl_3$)

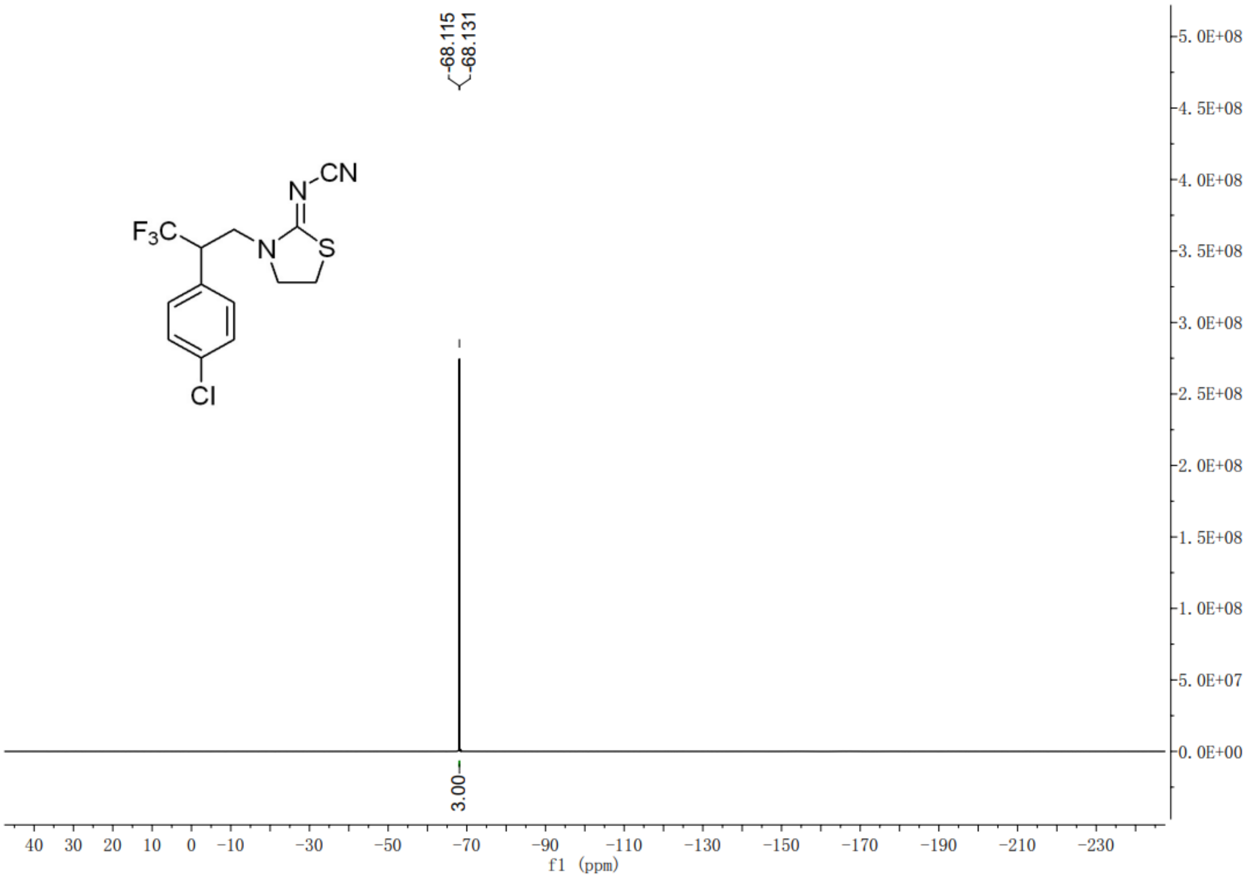

HRMS (EI) spectrum of 3ec

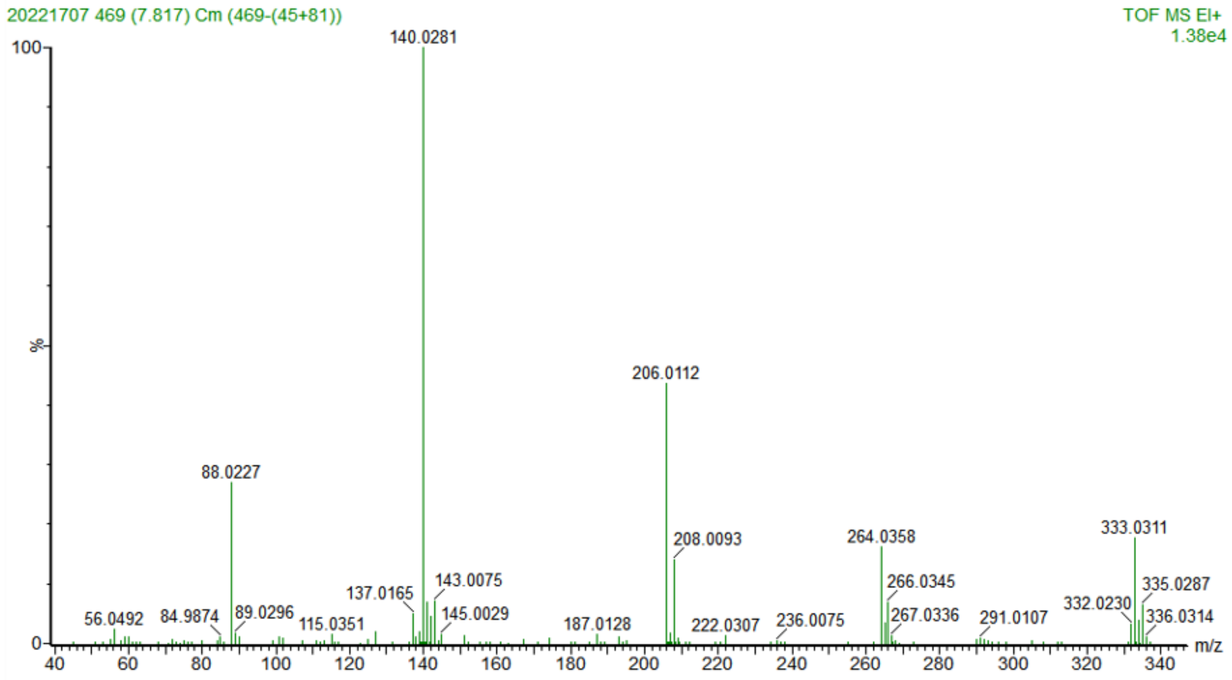

^{1}H NMR spectrum of 3tc (400 MHz, $CDCl_3$)

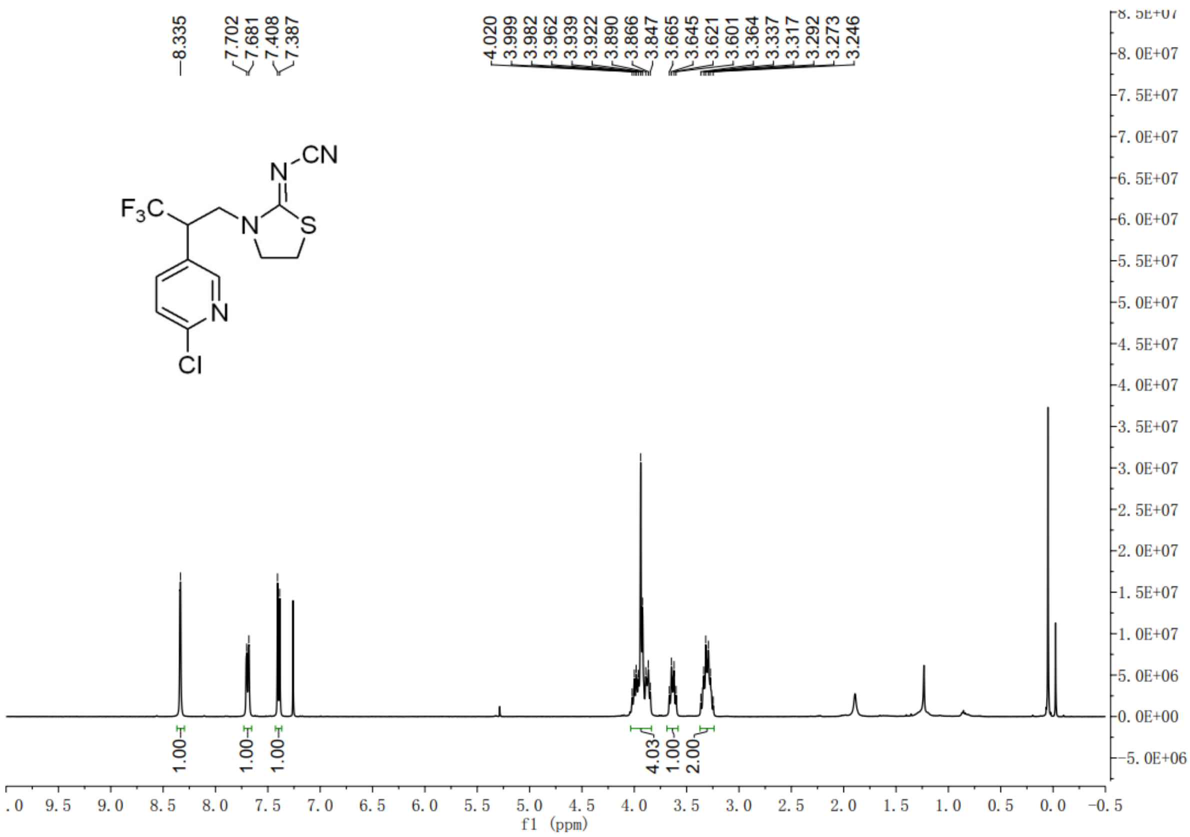

^{13}C NMR spectrum of 3tc (100 MHz, $CDCl_3$)

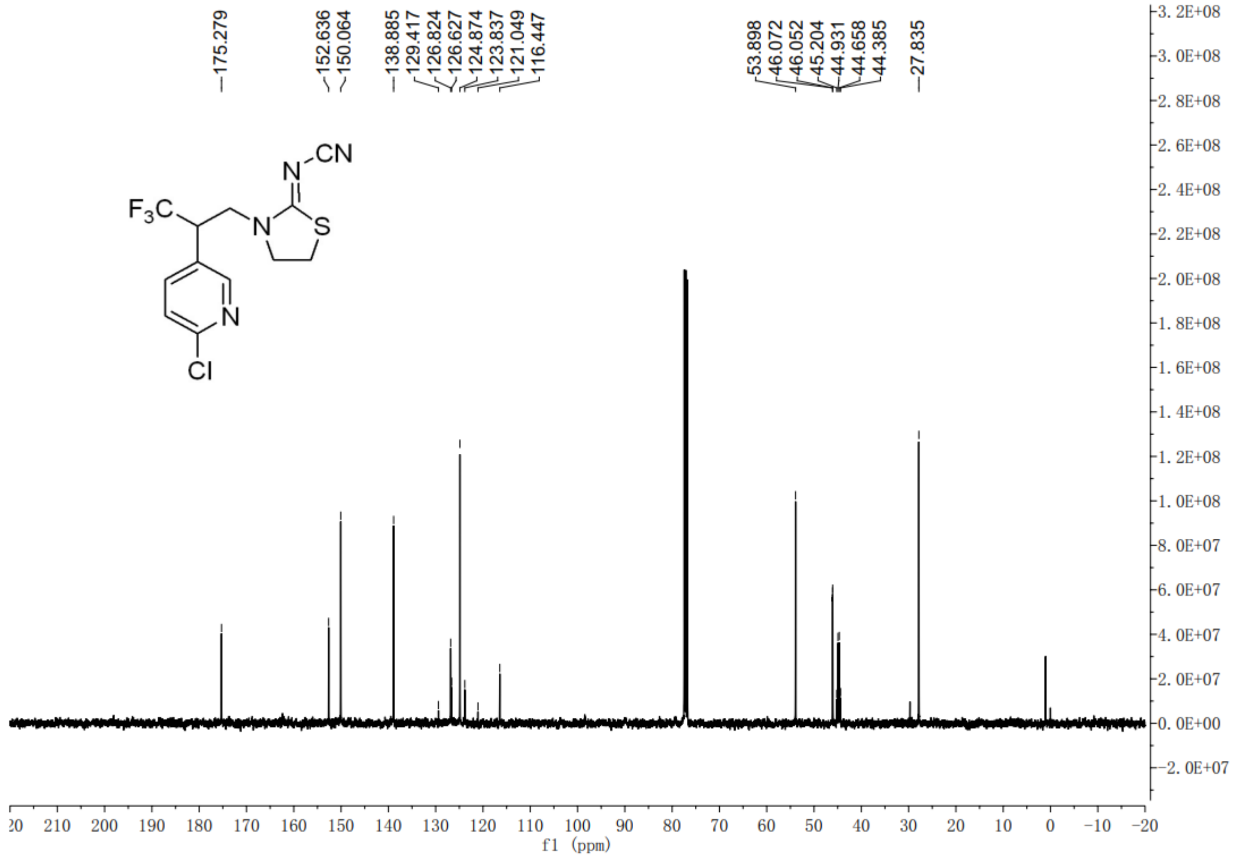

^{19}F NMR spectrum of 3tc (564 MHz, CDCl$_3$)

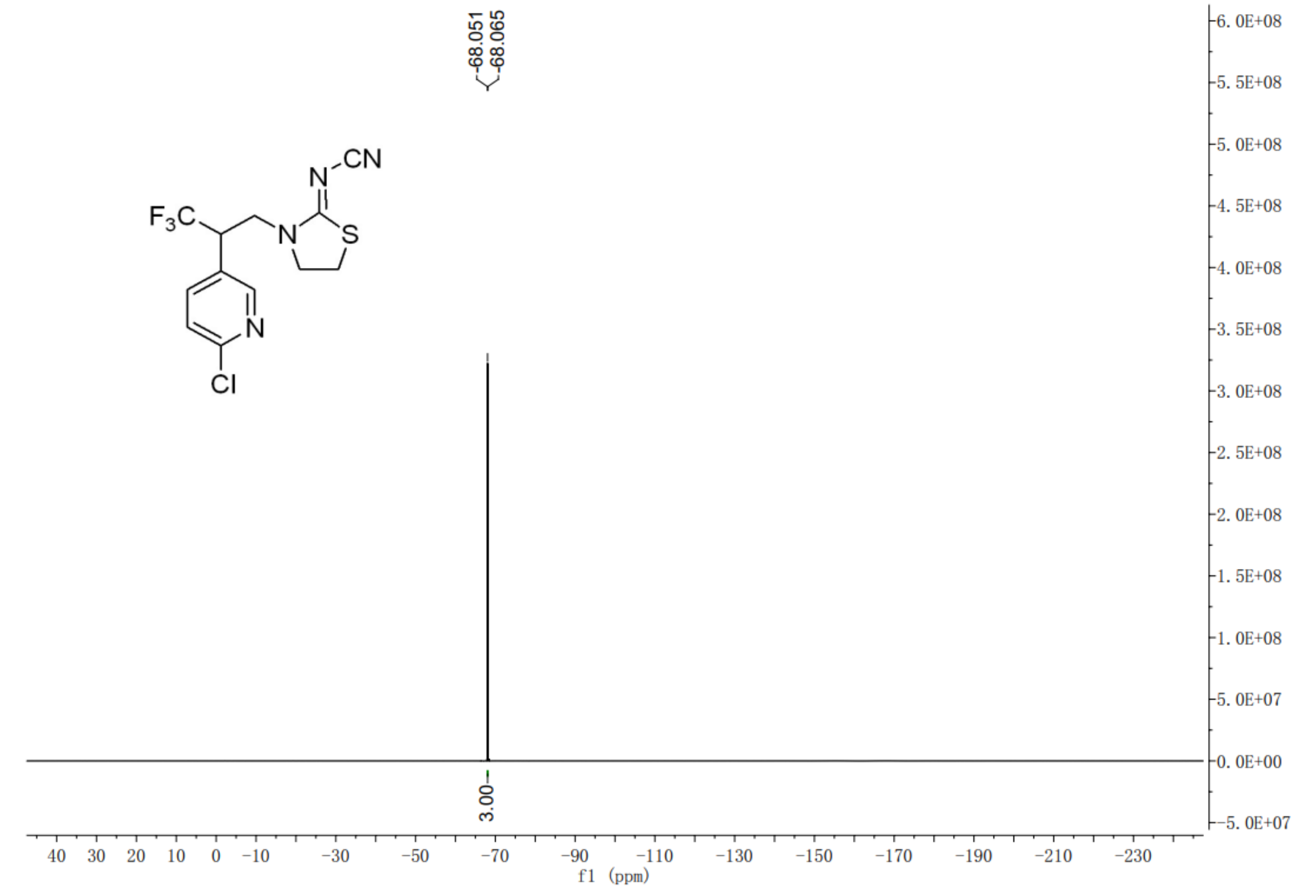

HRMS (EI) spectrum of 3tc

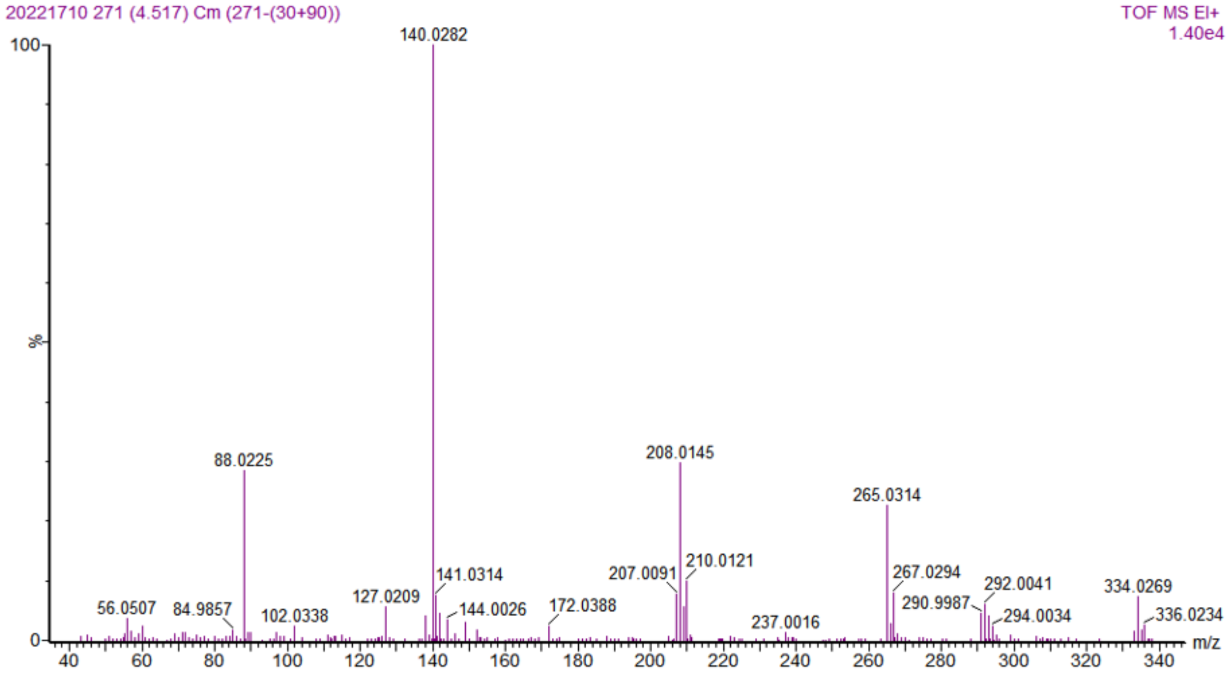

^{1}H NMR spectrum of 3cd (400 MHz, DMSO-d_6)

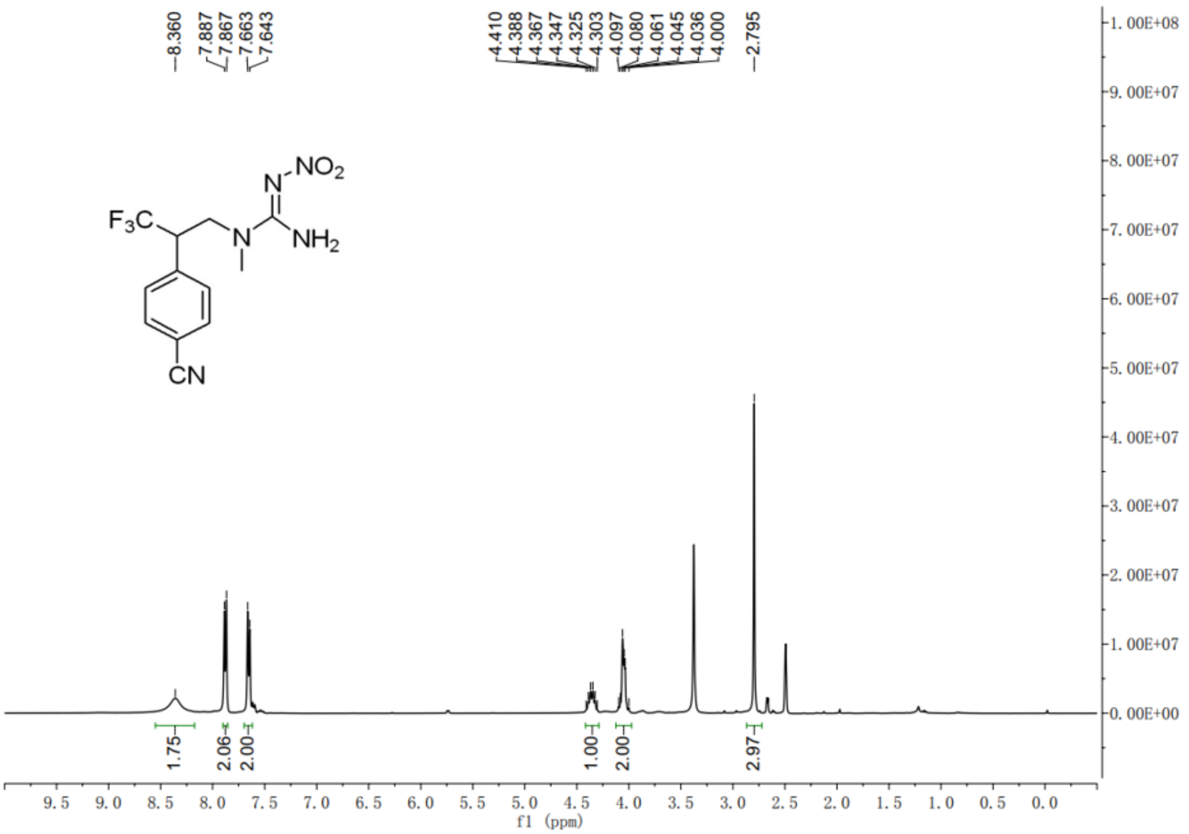

^{13}C NMR spectrum of 3cd (100 MHz, DMSO-d_6)

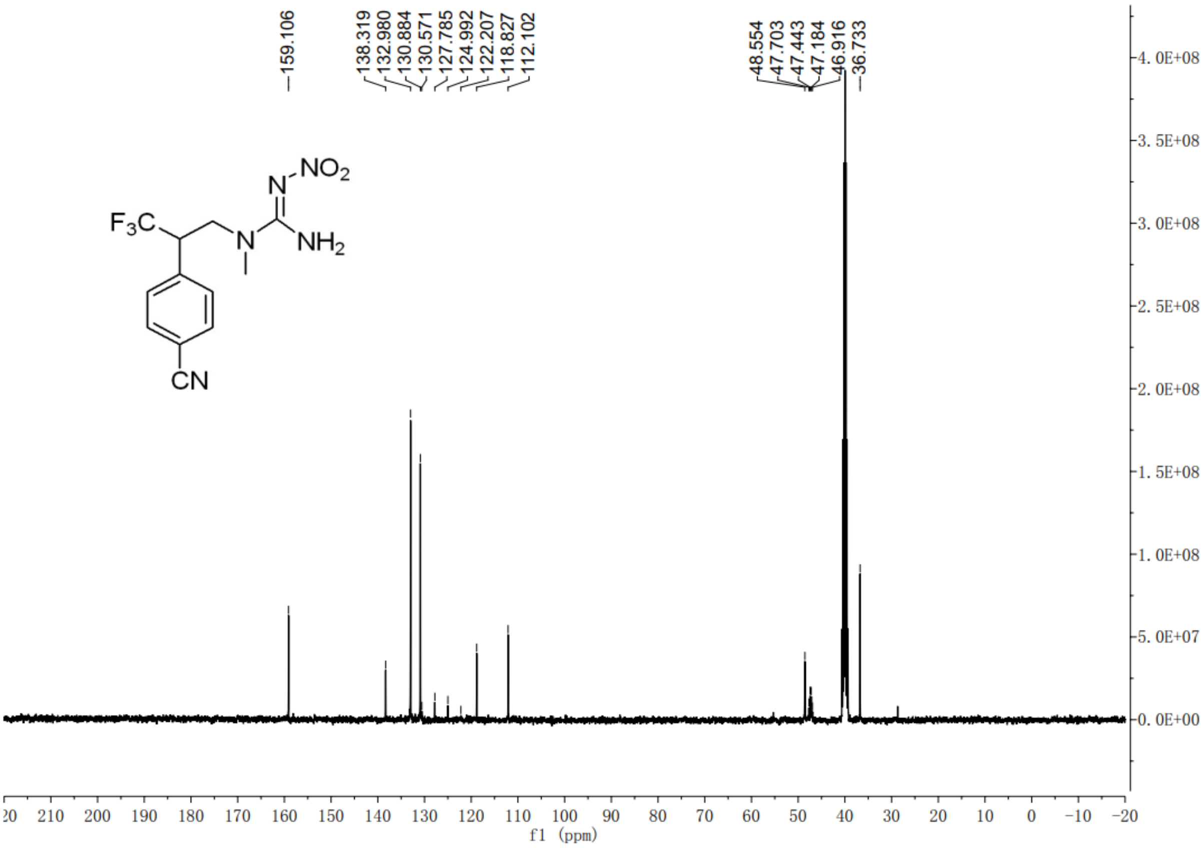

^{19}F NMR spectrum of 3cd (564 MHz, DMSO-d_6)

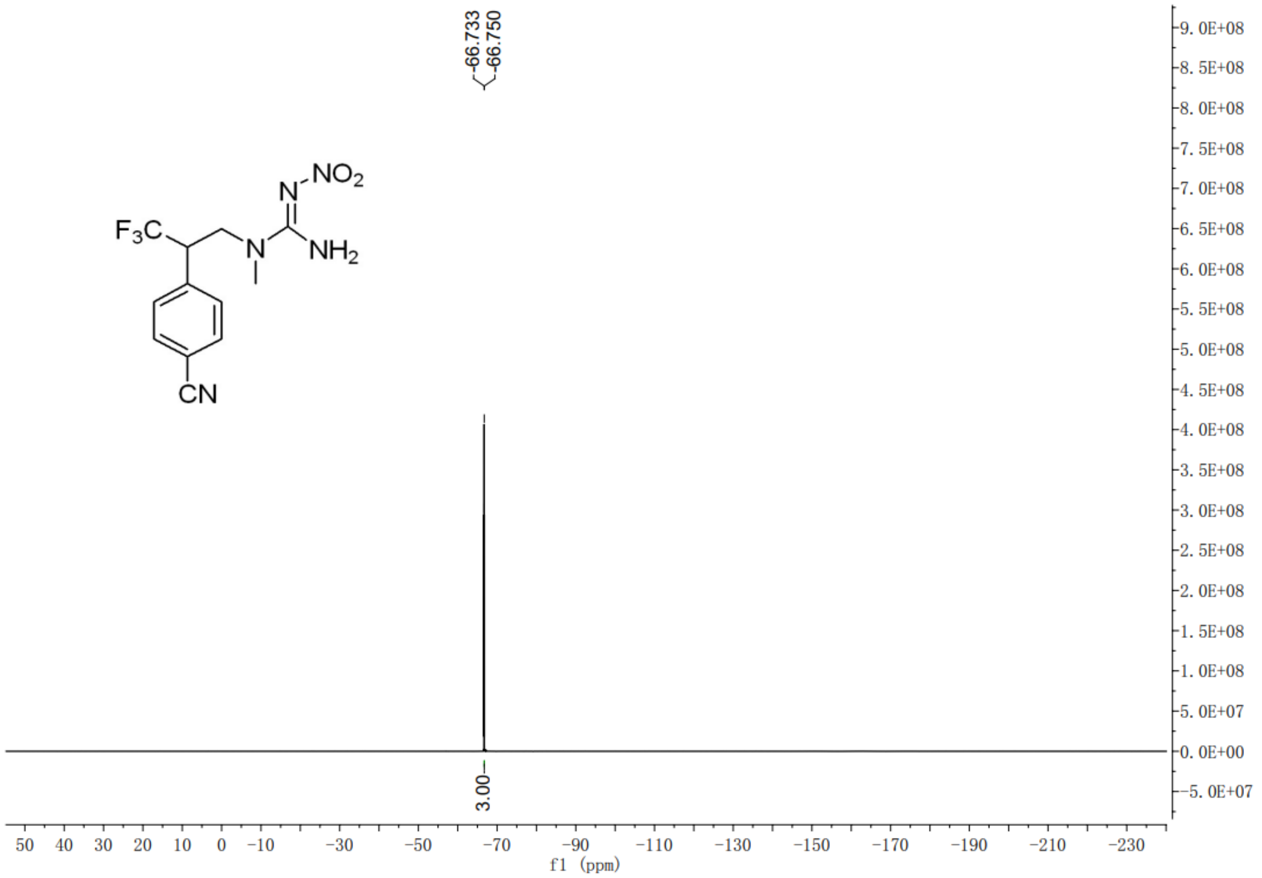

HRMS (EI) spectrum of 3cd

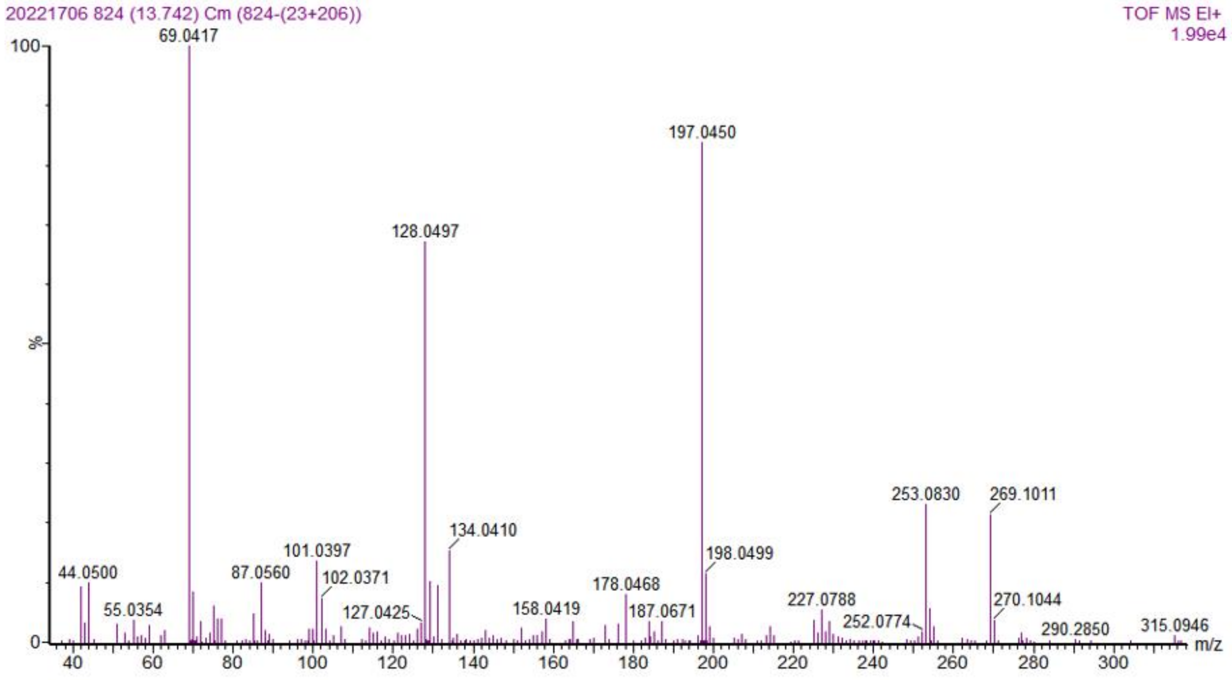

^{1}H NMR spectrum of 3td (400 MHz, DMSO-d_6)

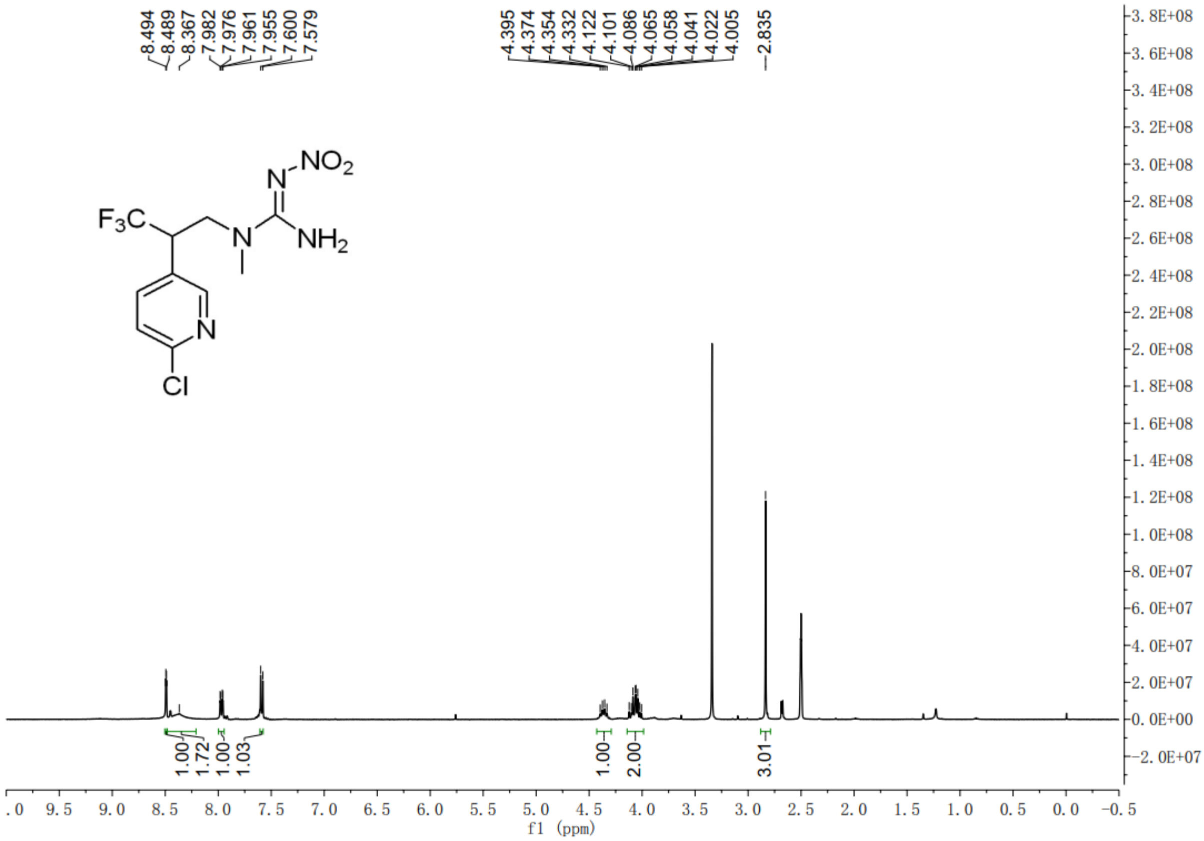

^{13}C NMR spectrum of 3td (150 MHz, DMSO-d_6)

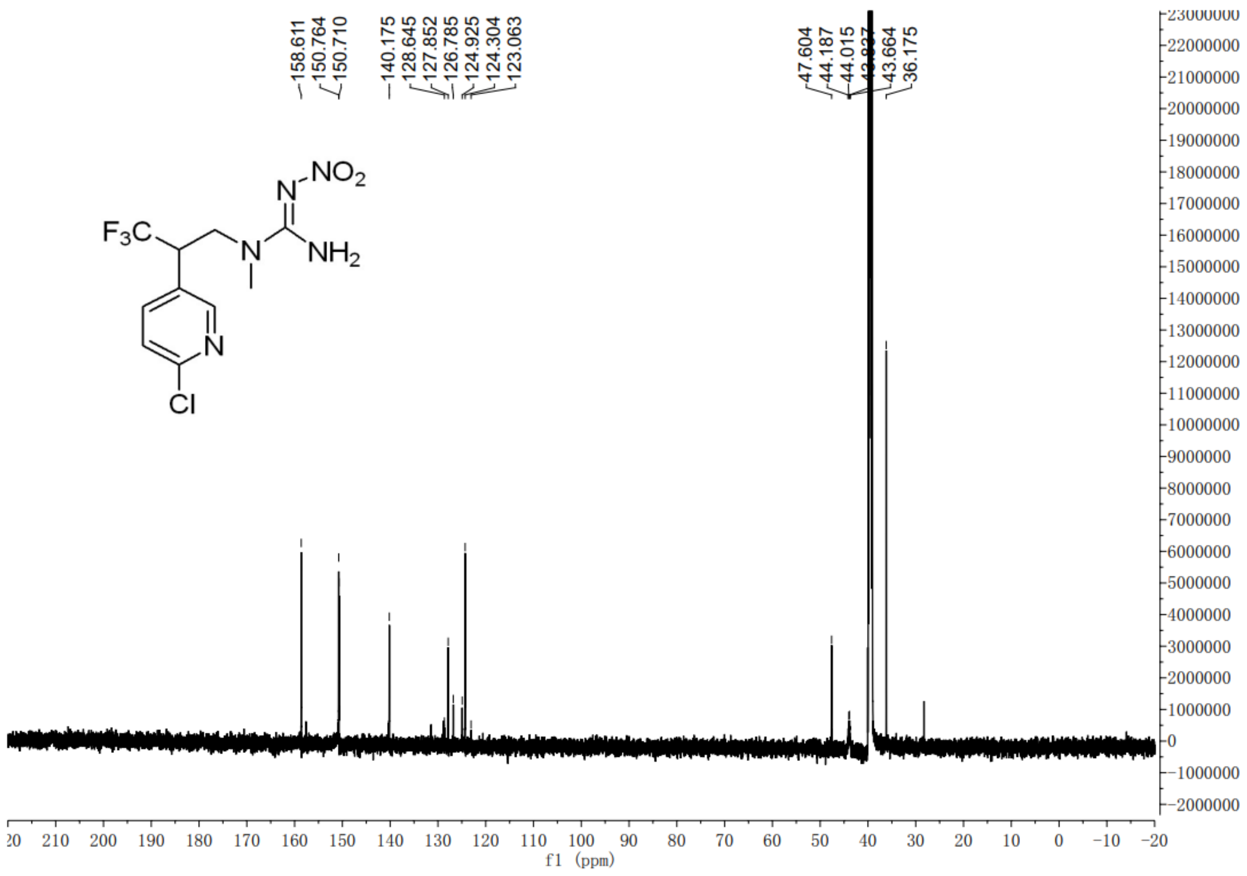

^{19}F NMR spectrum of 3td (564 MHz, DMSO-d_6)

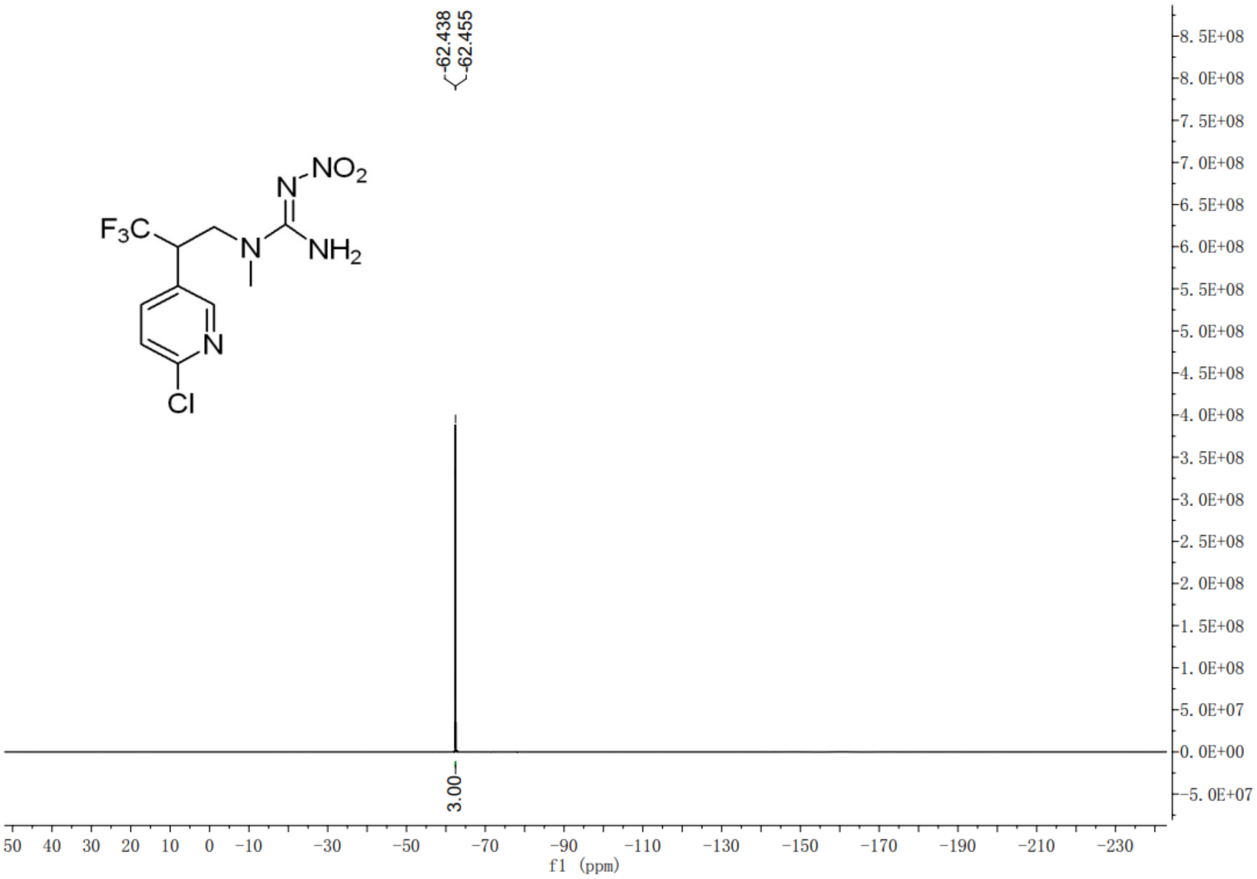

HRMS (EI) spectrum of 3td

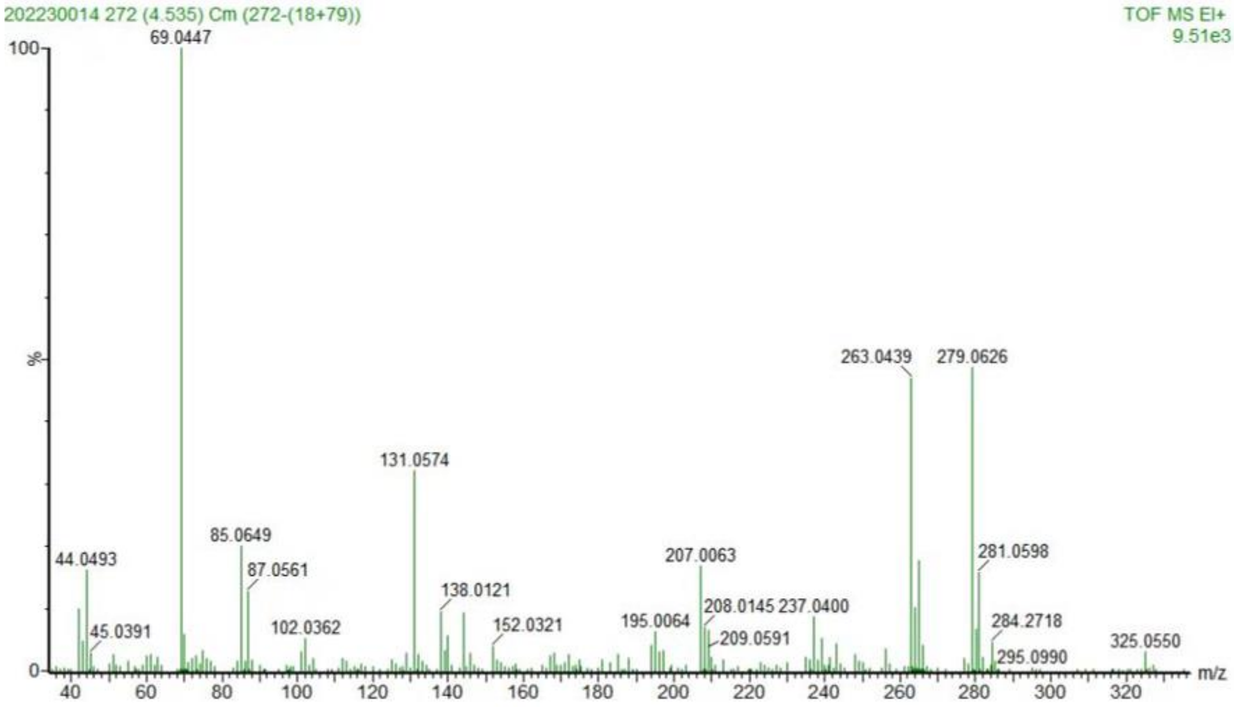

^{1}H NMR spectrum of 4aa (400 MHz, $CDCl_3$)

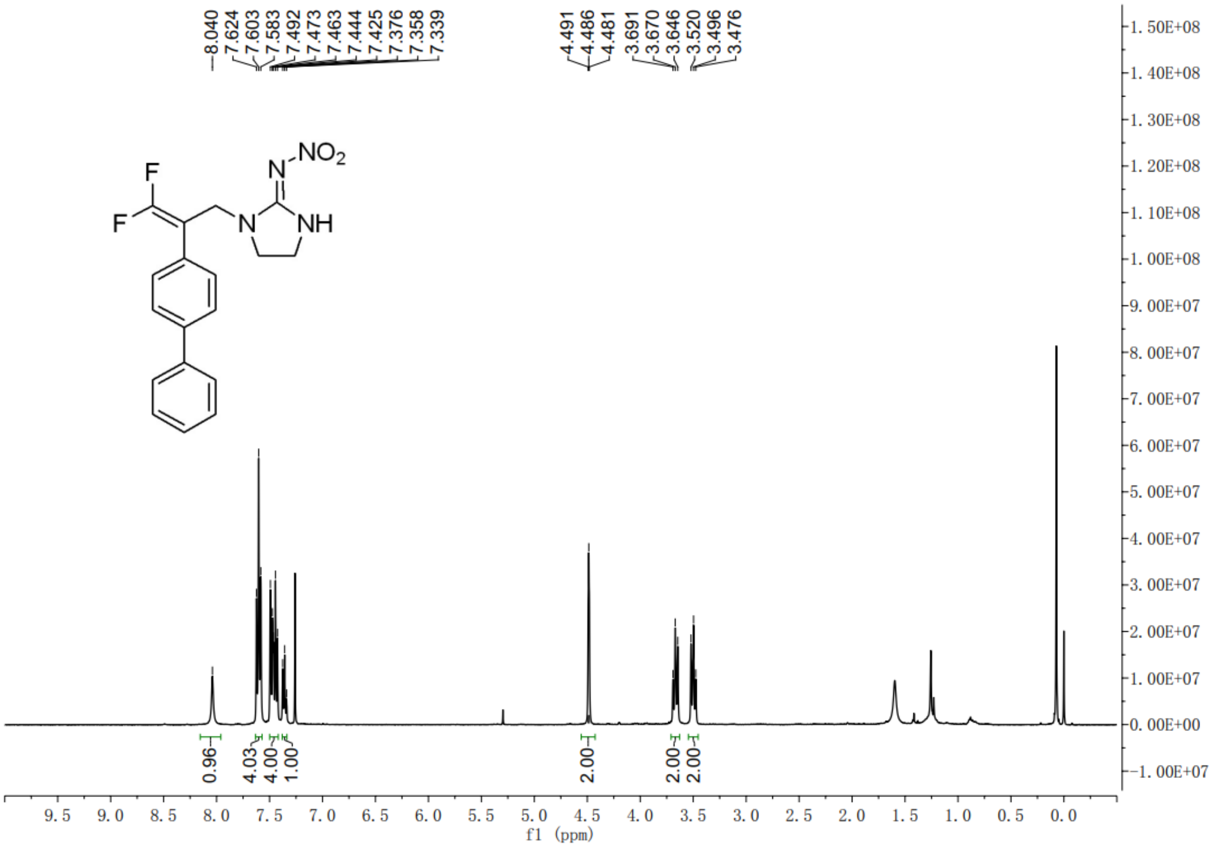

^{13}C NMR spectrum of 4aa (100 MHz, $CDCl_3$)

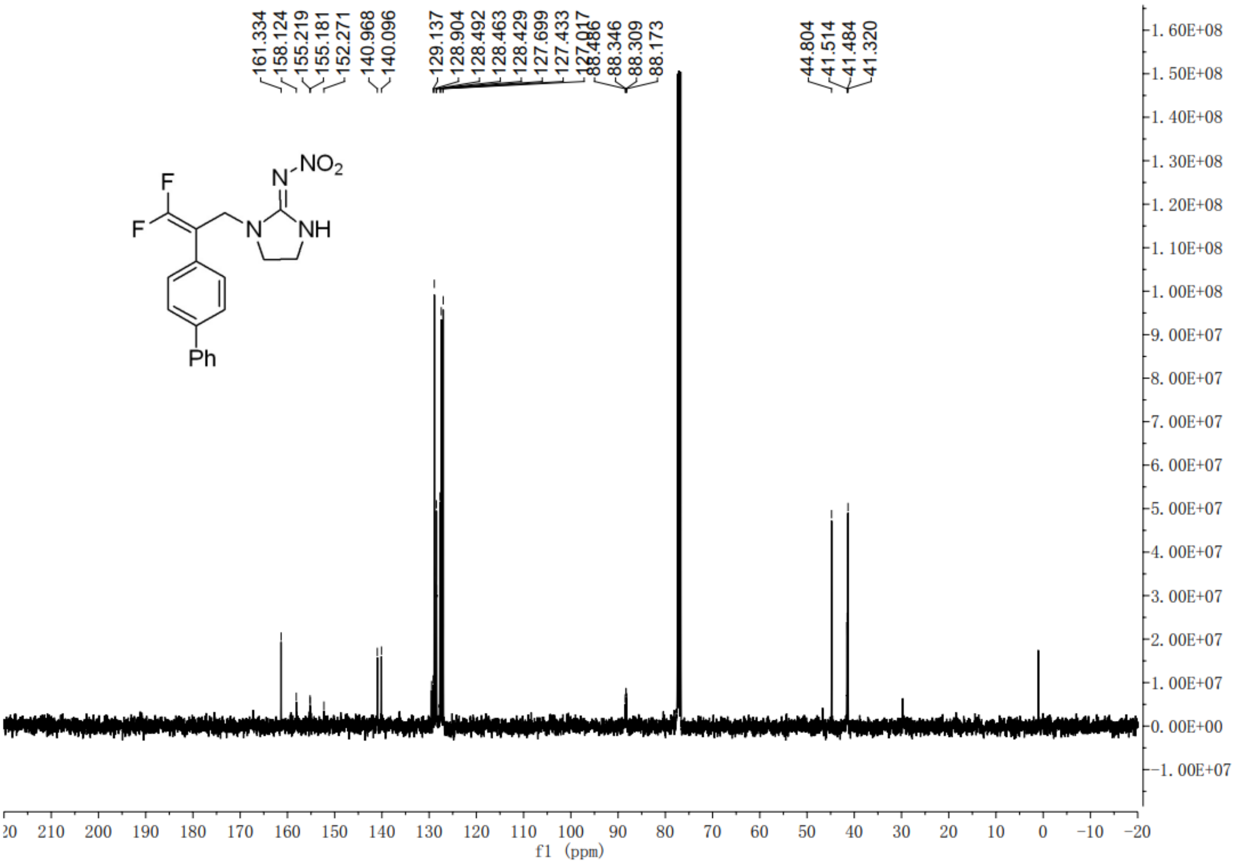

^{19}F NMR spectrum of 4aa (564 MHz, $CDCl_3$)

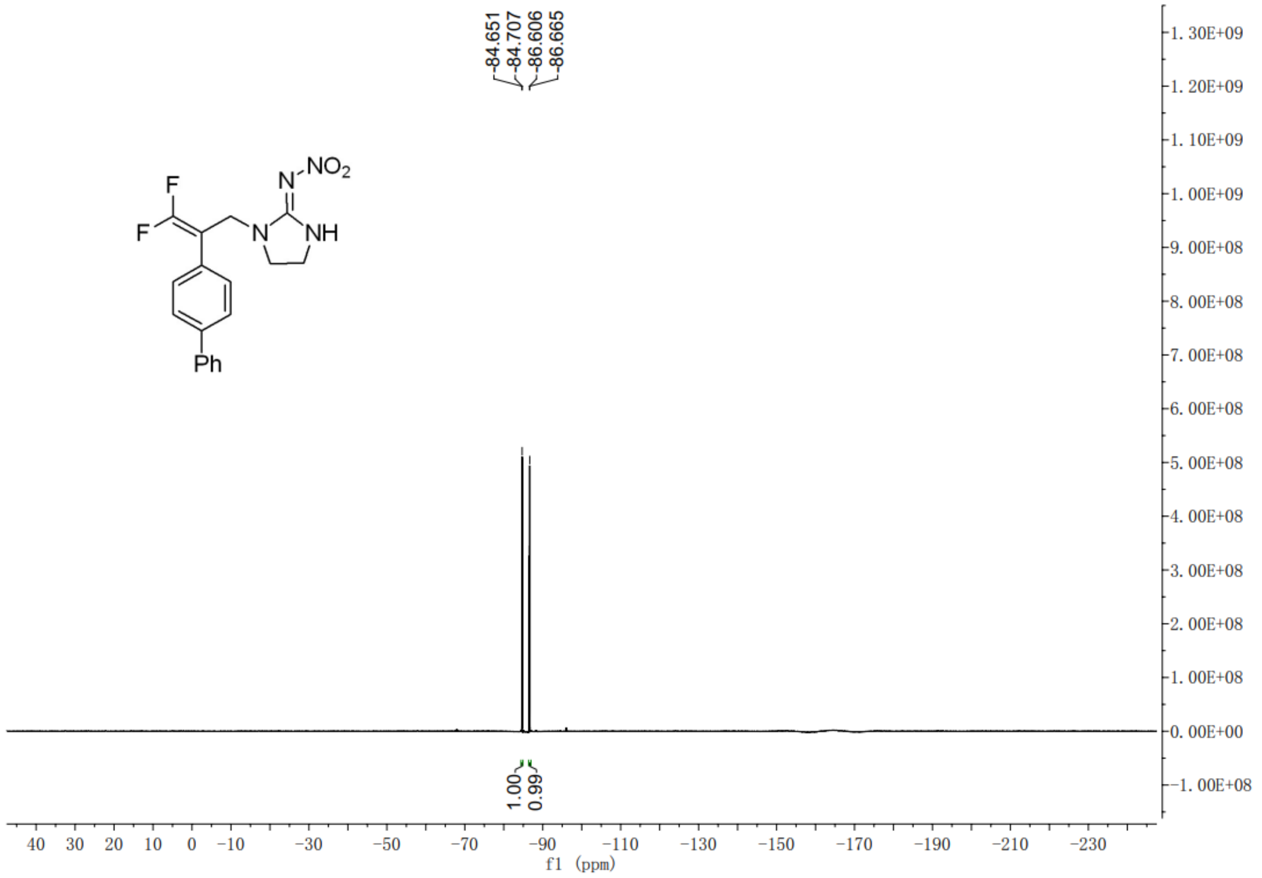

HRMS (ESI) spectrum of 4aa

Monoisotopic Mass, Even Electron Ions
78 formula(e) evaluated with 1 results within limits (all results (up to 1000) for each mass)
Elements Used:
C: 18-18 H: 16-16 N: 0-8 O: 0-6 F: 2-3 Na: 1-1

15
221219-2-1-- 21 (0.135)

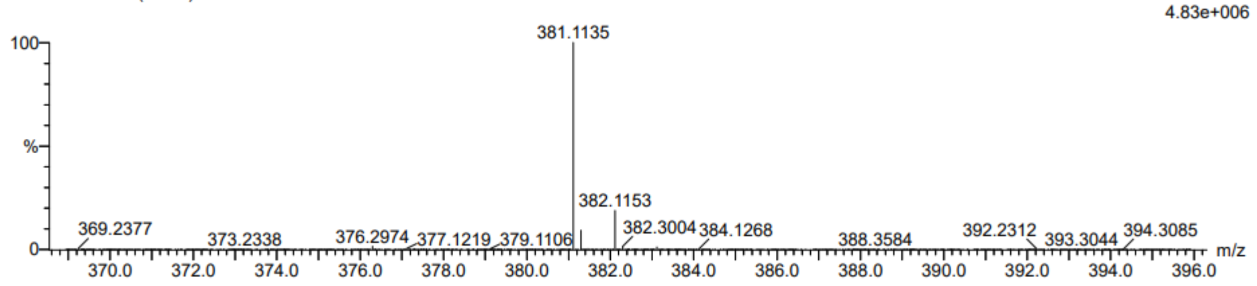

Minimum:				-1.5				
Maximum:		5.0	50.0	50.0				
Mass	Calc. Mass	mDa	PPM	DBE	i-FIT	Norm	Conf(%)	Formula
381.1135	381.1139	-0.4	-1.0	11.5	767.1	n/a	n/a	C18 H16 N4 O2 F2 Na

^{1}H NMR spectrum of 4fa (400 MHz, $CDCl_3$)

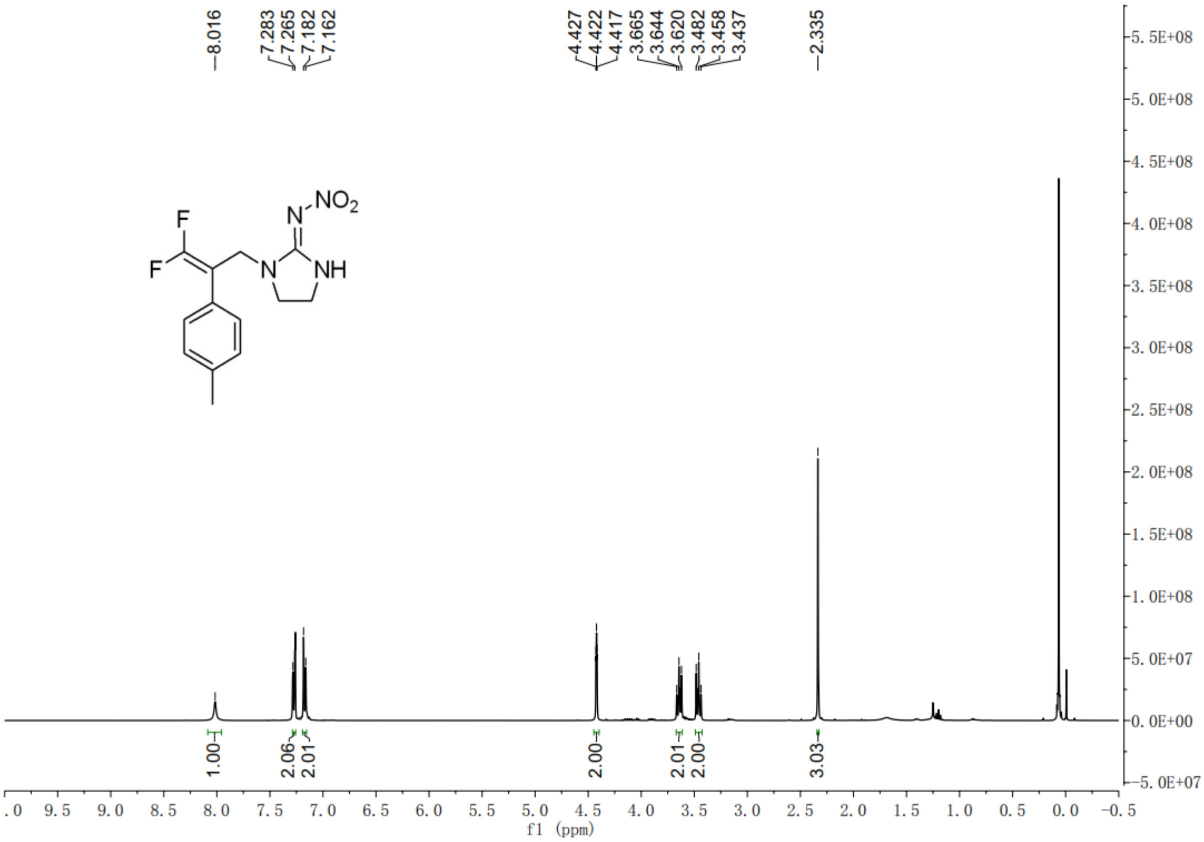

^{13}C NMR spectrum of 4fa (100 MHz, $CDCl_3$)

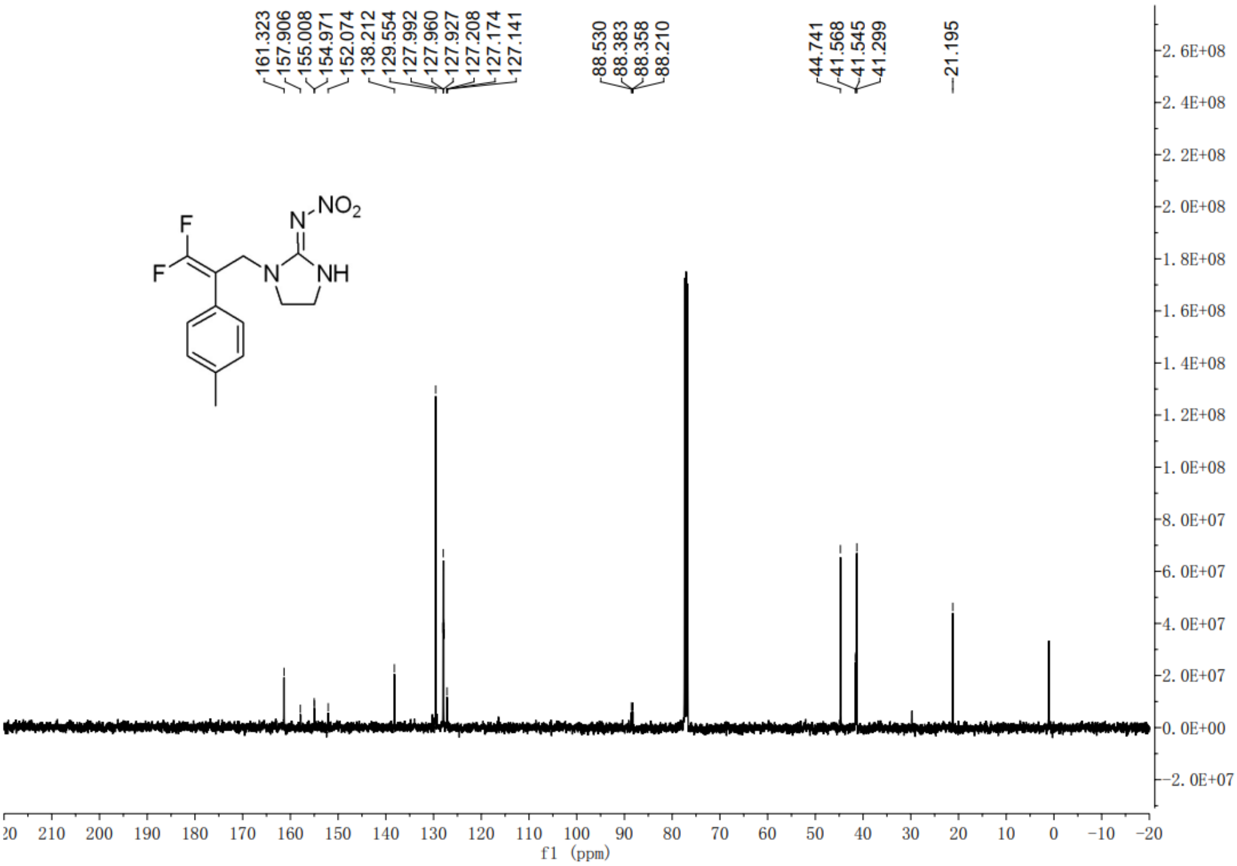

^{19}F NMR spectrum of 4fa (564 MHz, $CDCl_3$)

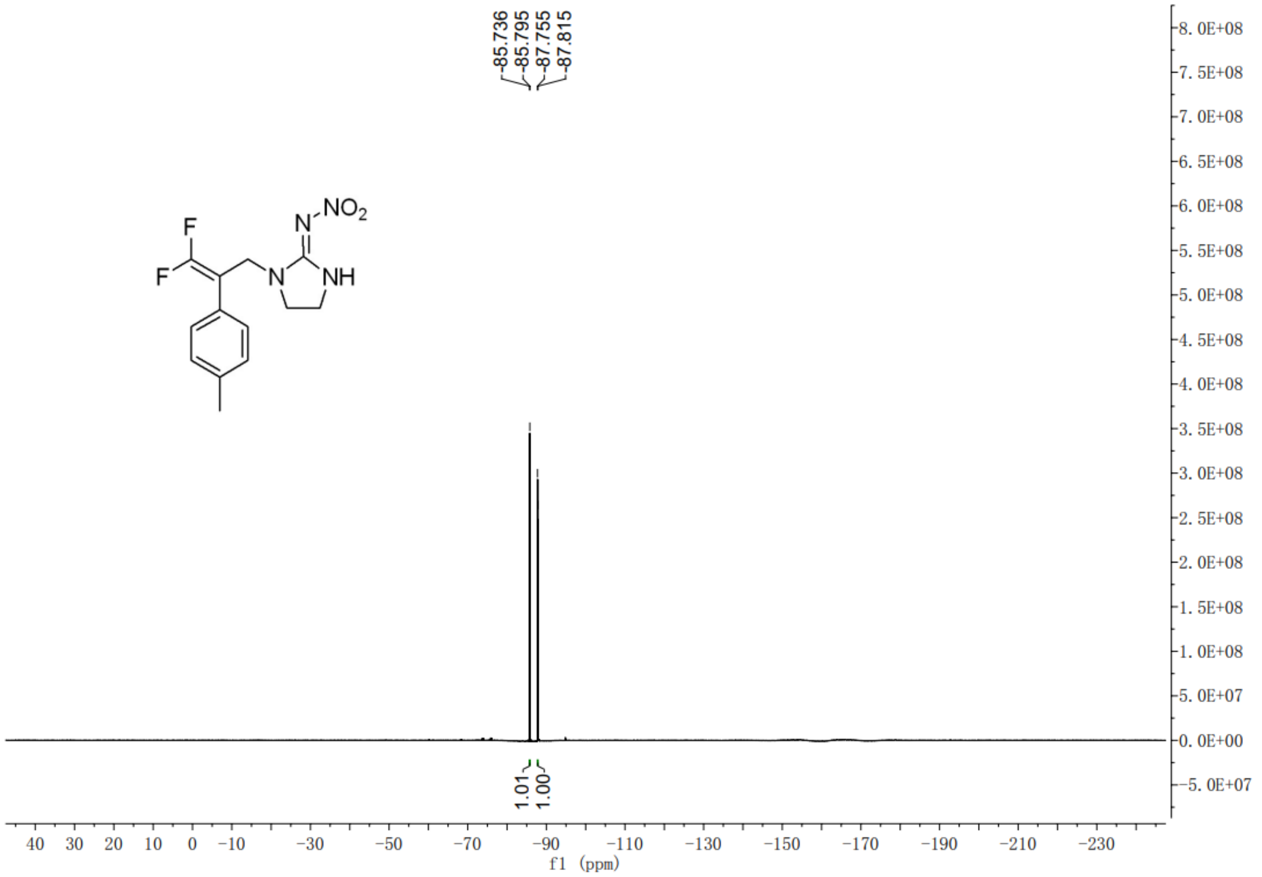

HRMS (ESI) spectrum of 4fa

Monoisotopic Mass, Even Electron Ions
86 formula(e) evaluated with 1 results within limits (all results (up to 1000) for each mass)
Elements Used:
C: 13-13 H: 14-16 N: 0-8 O: 0-6 F: 2-3 Na: 1-1
15
221219-2-4 26 (0.161)

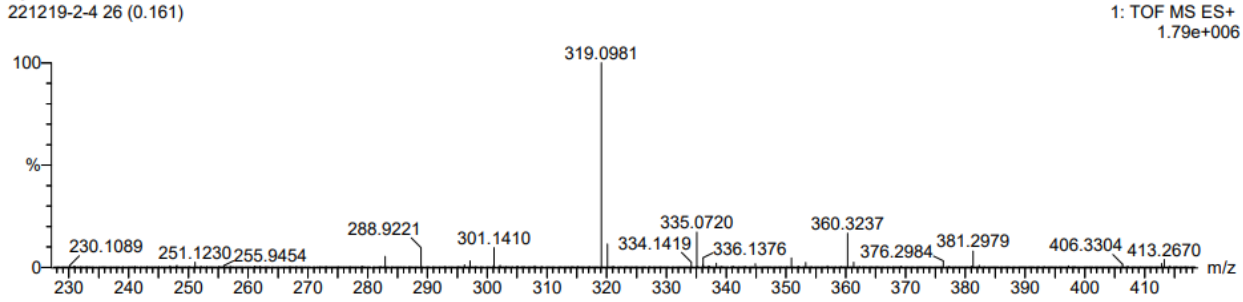

Minimum:				-1.5				
Maximum:		5.0	50.0	50.0				
Mass	Calc. Mass	mDa	PPM	DBE	i-FIT	Norm	Conf(%)	Formula
319.0981	319.0983	-0.2	-0.6	7.5	664.0	n/a	n/a	C13 H14 N4 O2 F2 Na

^{1}H NMR spectrum of 4ua (400 MHz, acetone-d_6)

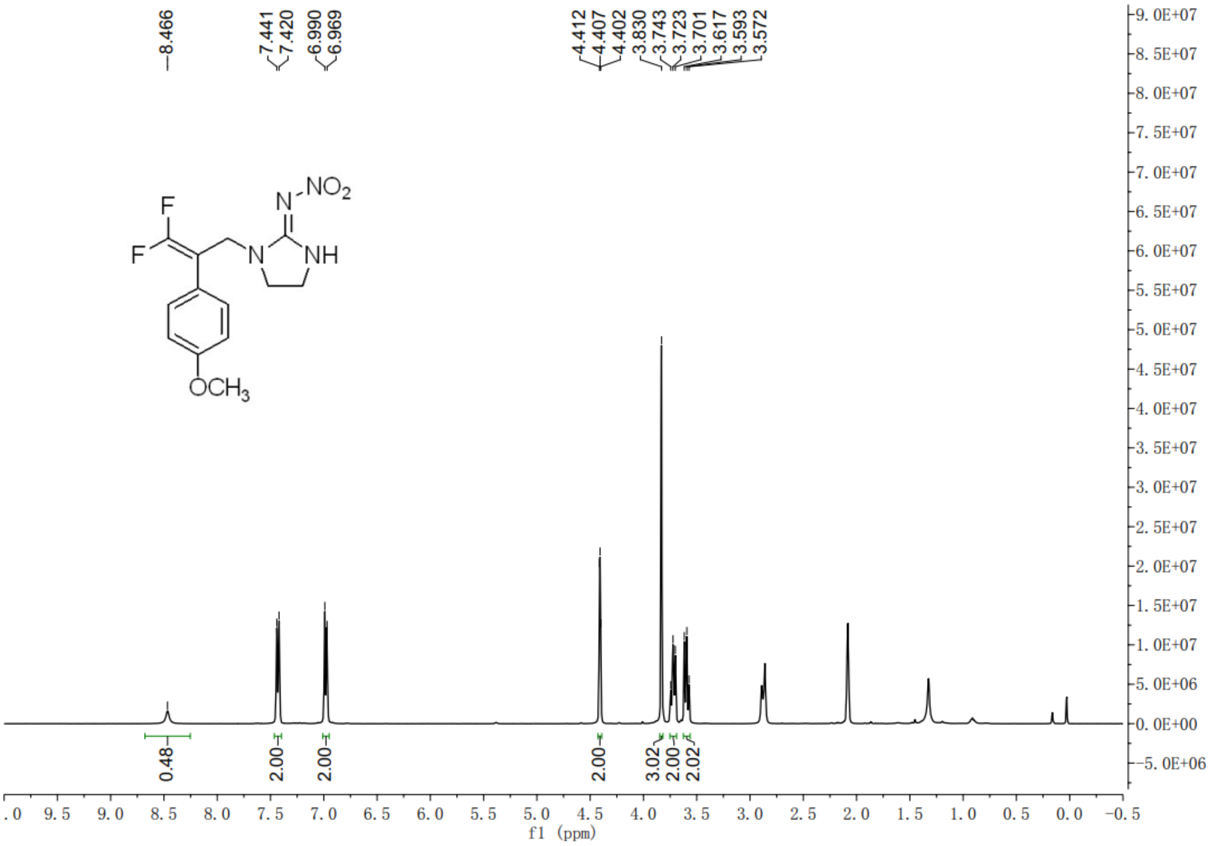

^{13}C NMR spectrum of 4ua (100 MHz, acetone-d_6)

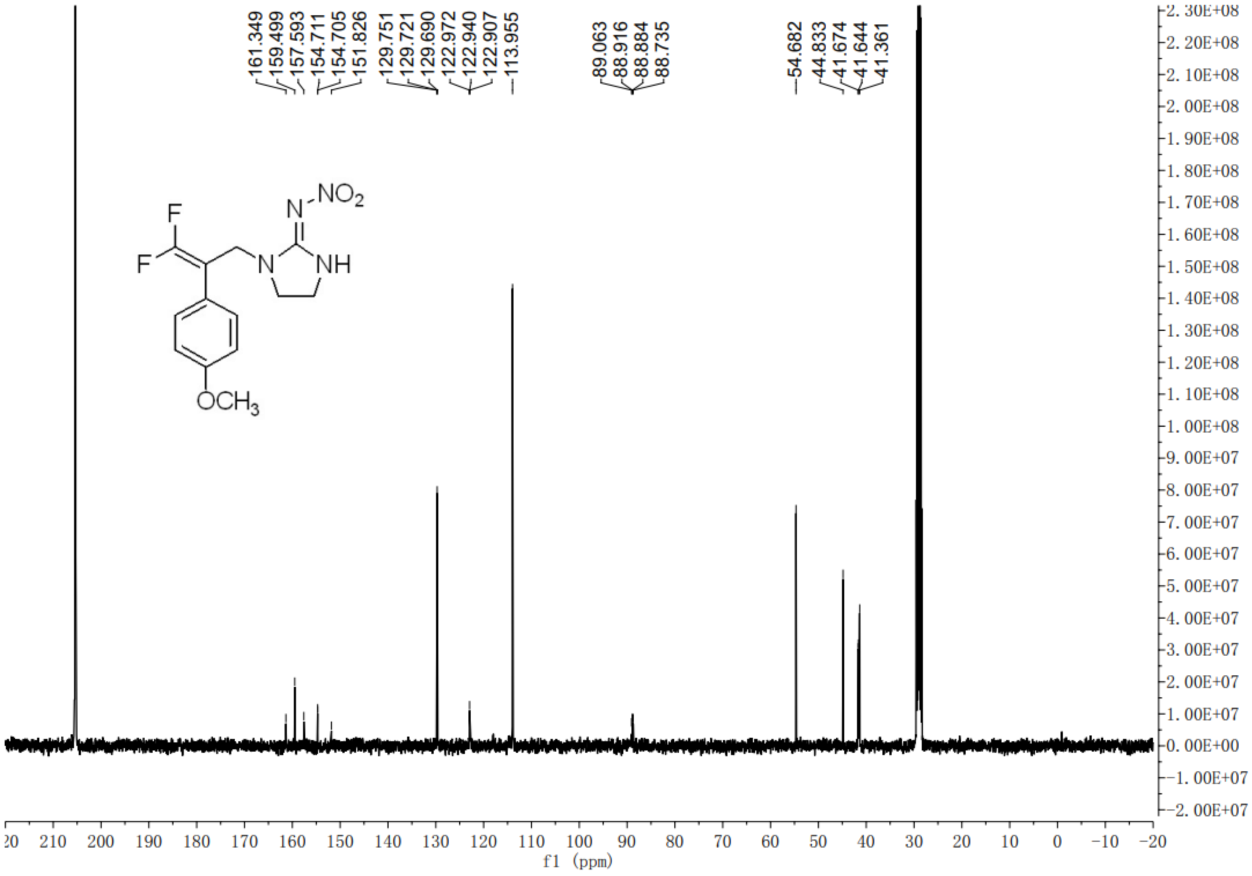

^{19}F NMR spectrum of 4ua (564 MHz, acetone-d_6)

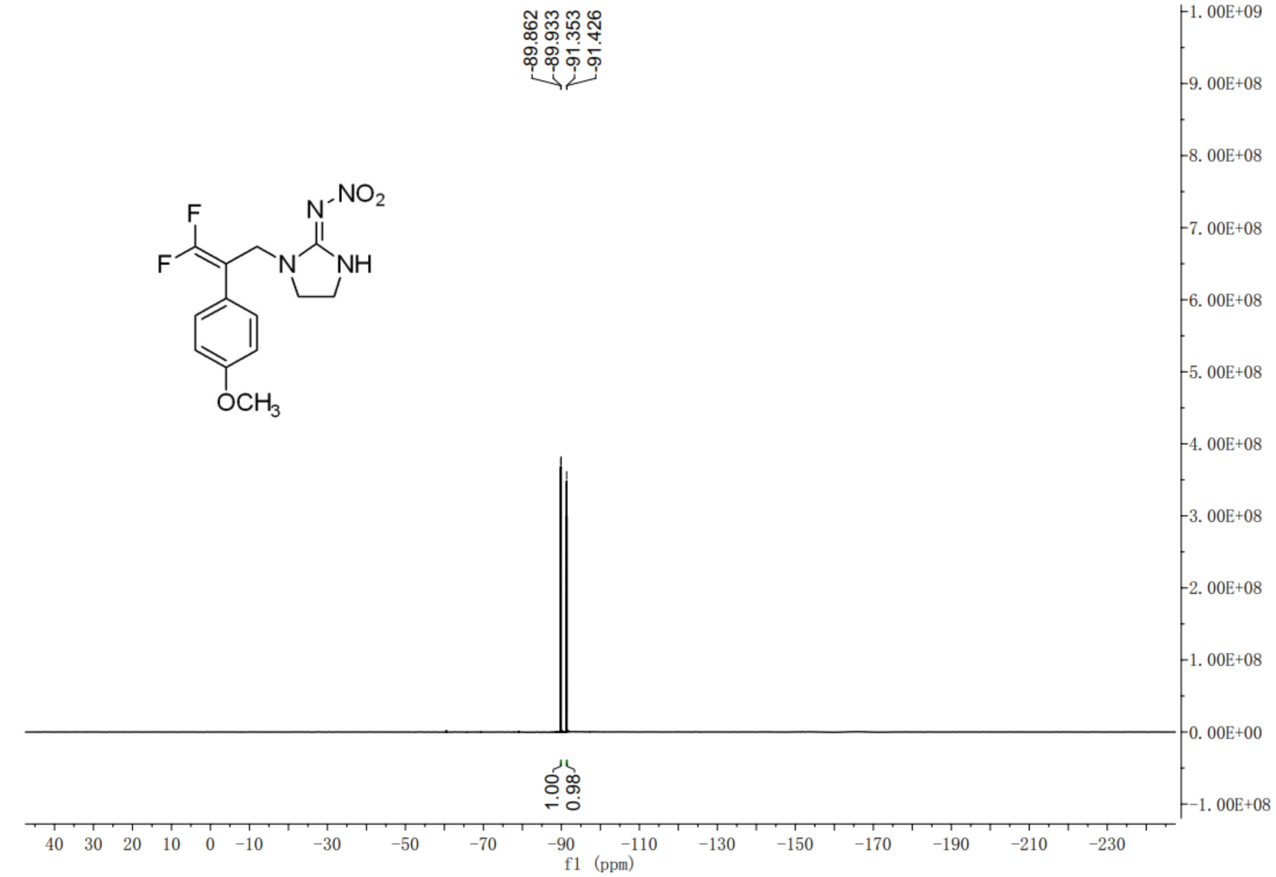

HRMS (ESI) spectrum of 4ua

Monoisotopic Mass, Even Electron Ions
60 formula(e) evaluated with 1 results within limits (all results (up to 1000) for each mass)
Elements Used:
C: 13-13 H: 0-45 N: 0-8 O: 0-6 F: 2-2 Na: 1-1

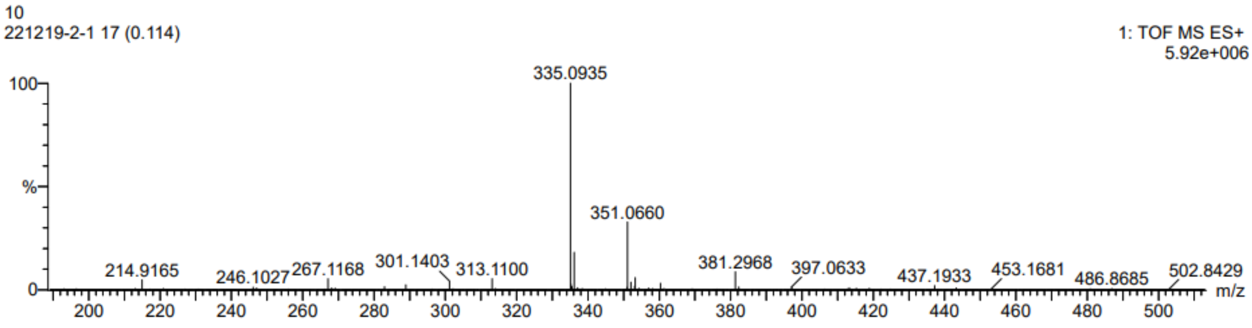

Minimum:				-1.5				
Maximum:		5.0	50.0	50.0				
Mass	Calc. Mass	mDa	PPM	DBE	i-FIT	Norm	Conf(%)	Formula
335.0935	335.0932	0.3	0.9	7.5	701.1	n/a	n/a	C13 H14 N4 O3 F2 Na

^{1}H NMR spectrum of 4va (400 MHz, $CDCl_3$)

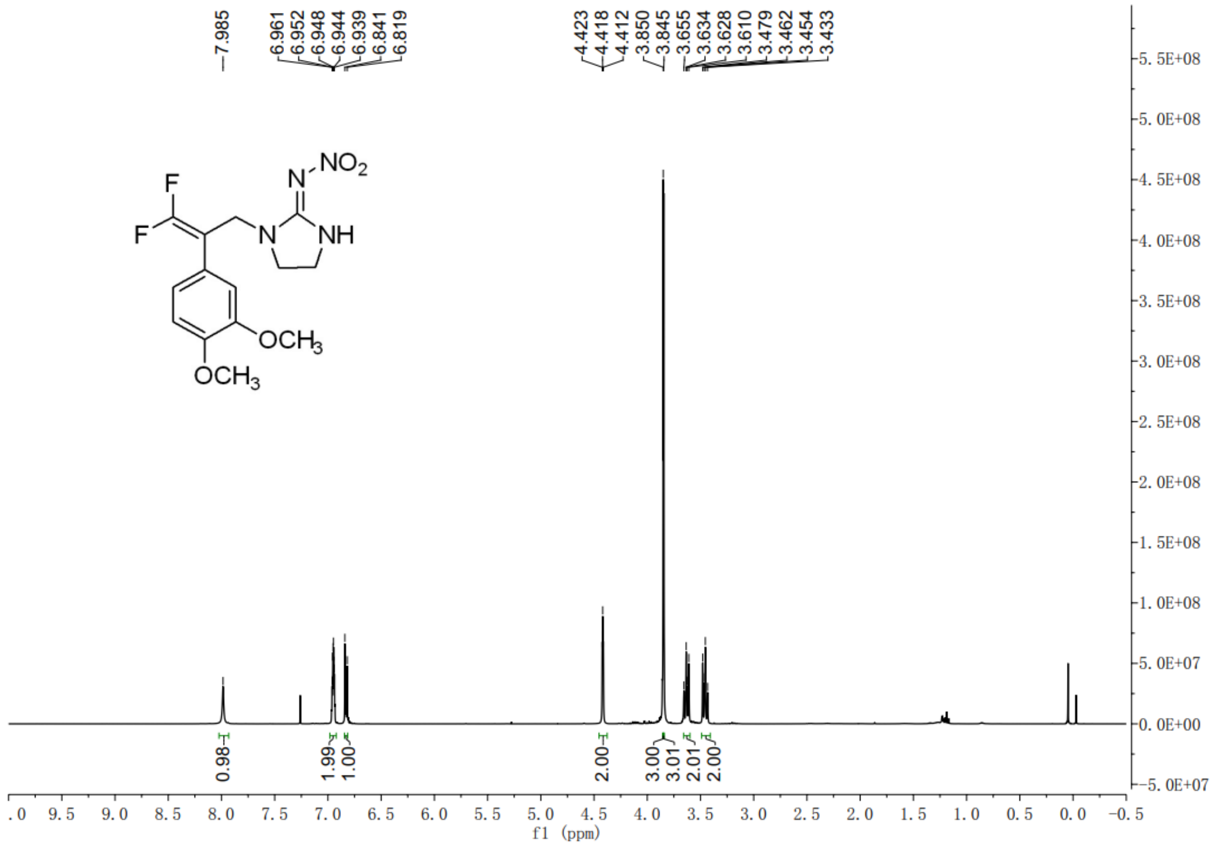

^{13}C NMR spectrum of 4va (100 MHz, $CDCl_3$)

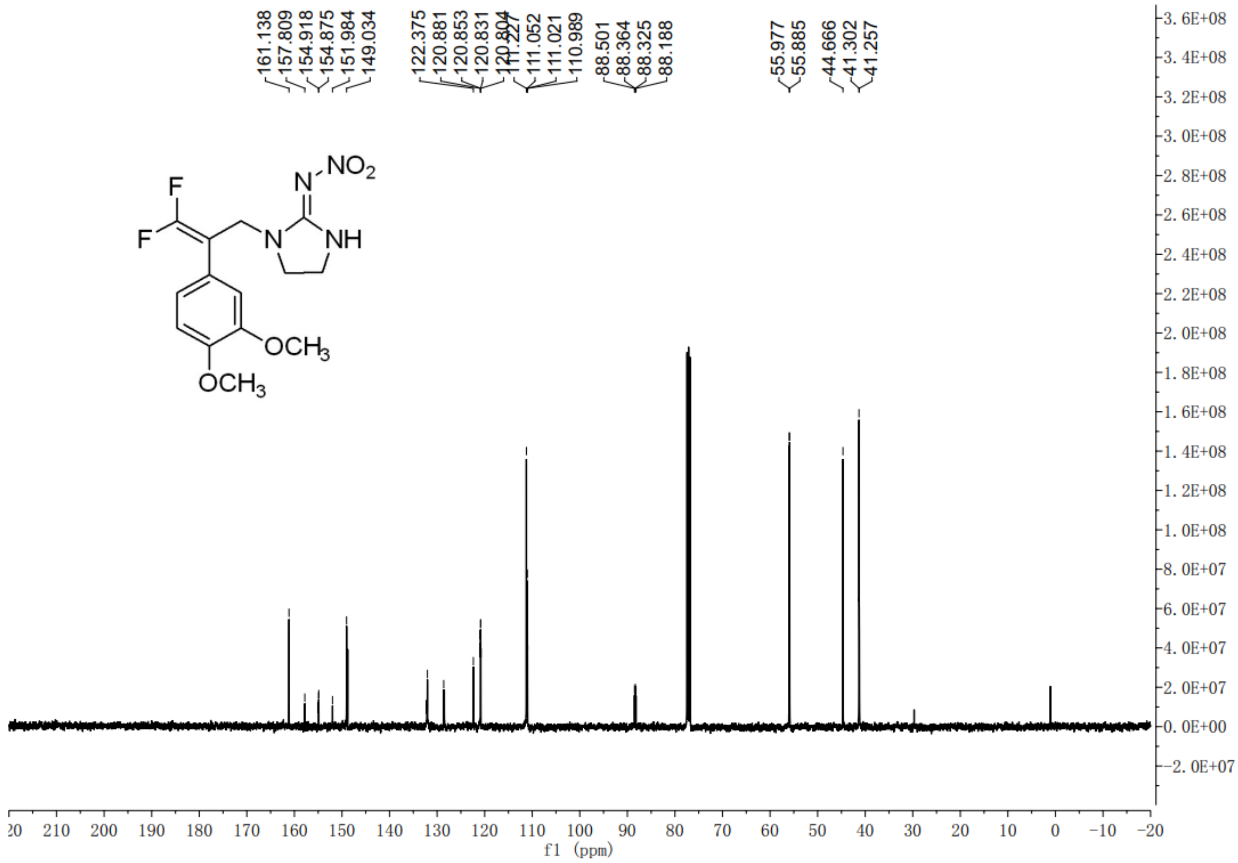

^{19}F NMR spectrum of 4va (564 MHz, $CDCl_3$)

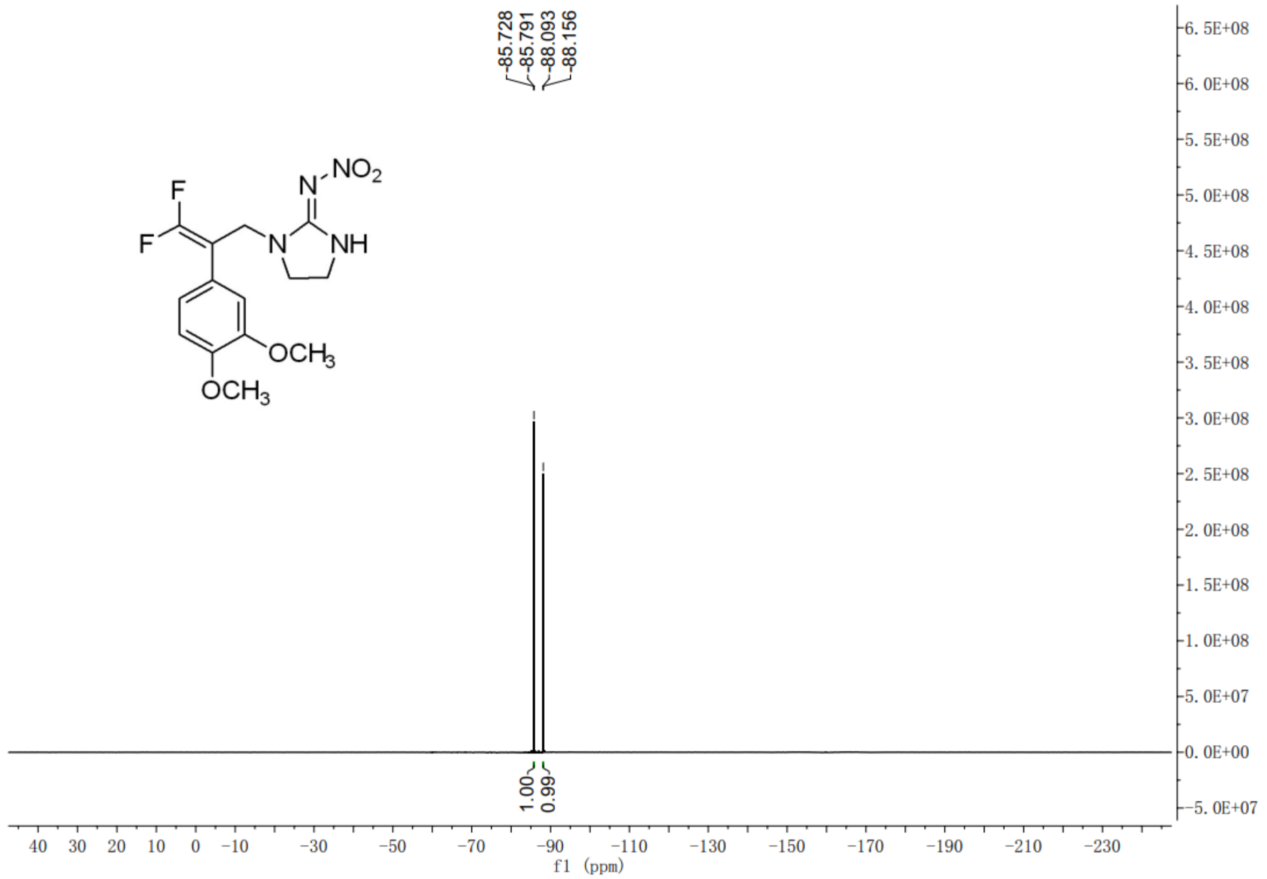

HRMS (ESI) spectrum of 4va

Monoisotopic Mass, Even Electron Ions
78 formula(e) evaluated with 1 results within limits (all results (up to 1000) for each mass)
Elements Used:
C: 14-14 H: 14-16 N: 0-8 O: 0-6 F: 2-3 Na: 1-1
15
221219-2-3 21 (0.135) 1: TOF MS ES+
4.70e+006

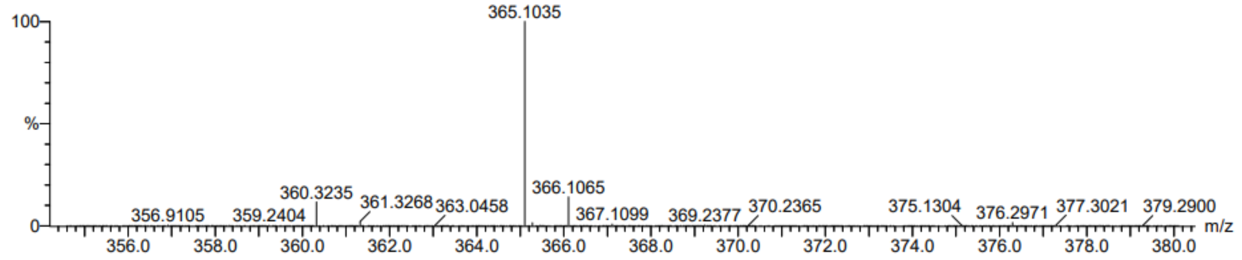

Minimum:				-1.5				
Maximum:		5.0	50.0	50.0				
Mass	Calc. Mass	mDa	PPM	DBE	i-FIT	Norm	Conf(%)	Formula
365.1035	365.1037	-0.2	-0.5	7.5	710.5	n/a	n/a	C14 H16 N4 O4 F2 Na

^{1}H NMR spectrum of 4wa (400 MHz, $CDCl_3$)

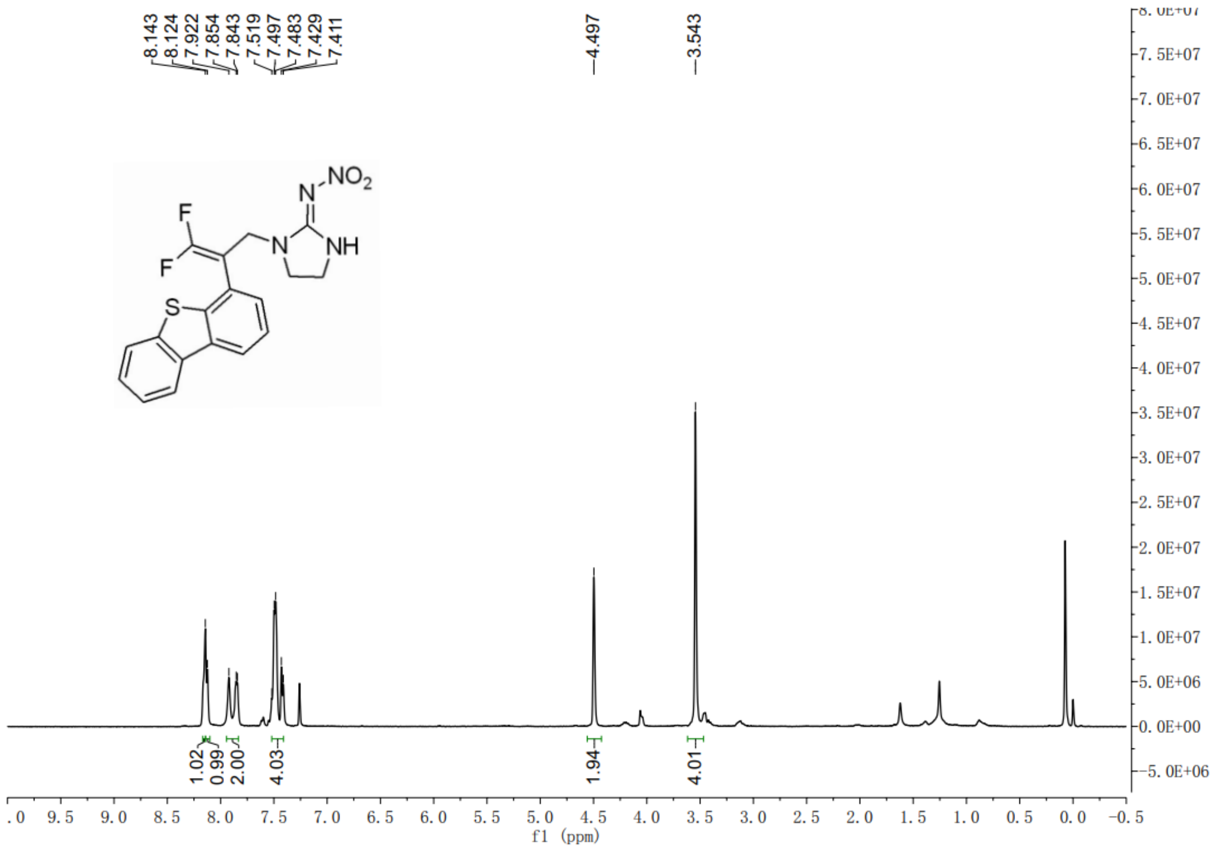

^{13}C NMR spectrum of 4wa (100 MHz, $CDCl_3$)

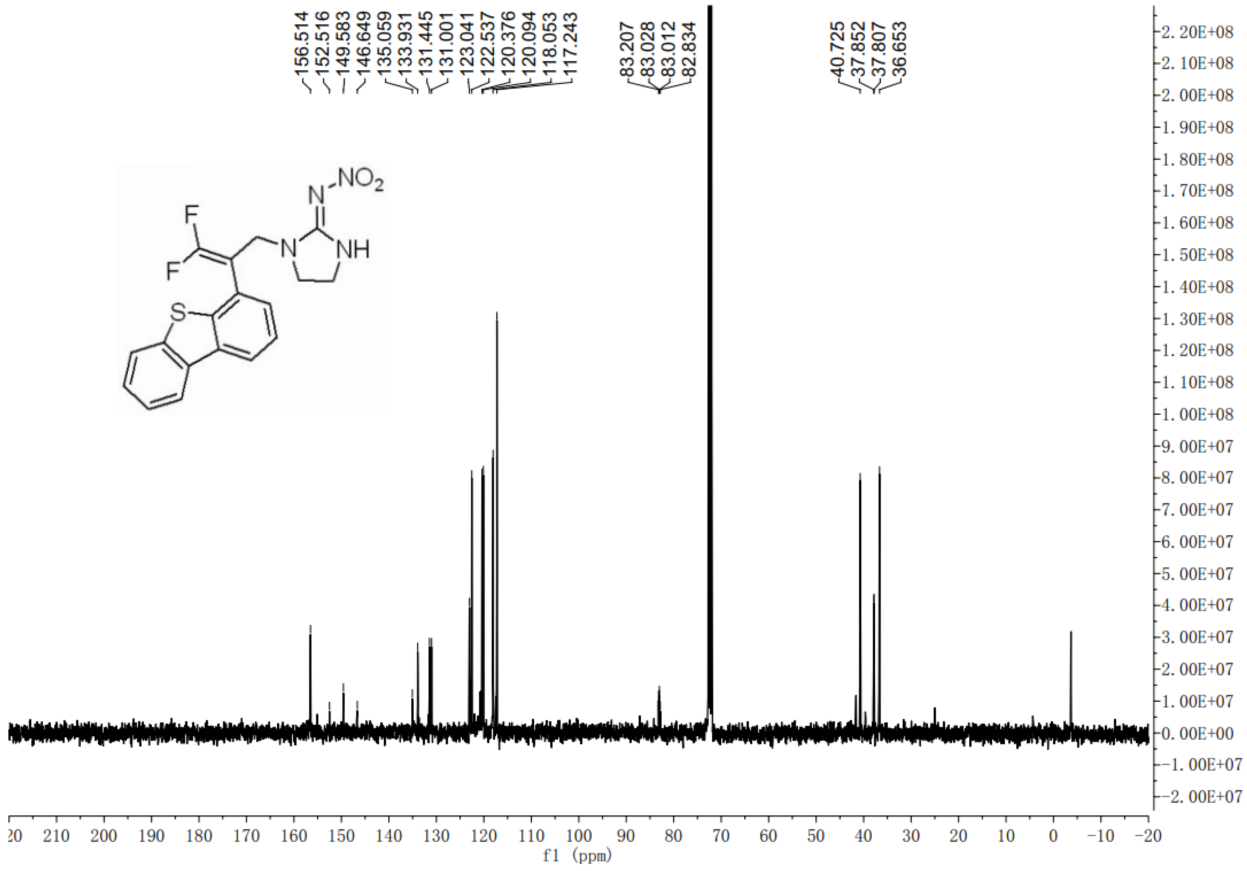

^{19}F NMR spectrum of 4wa (564 MHz, CDCl$_3$)

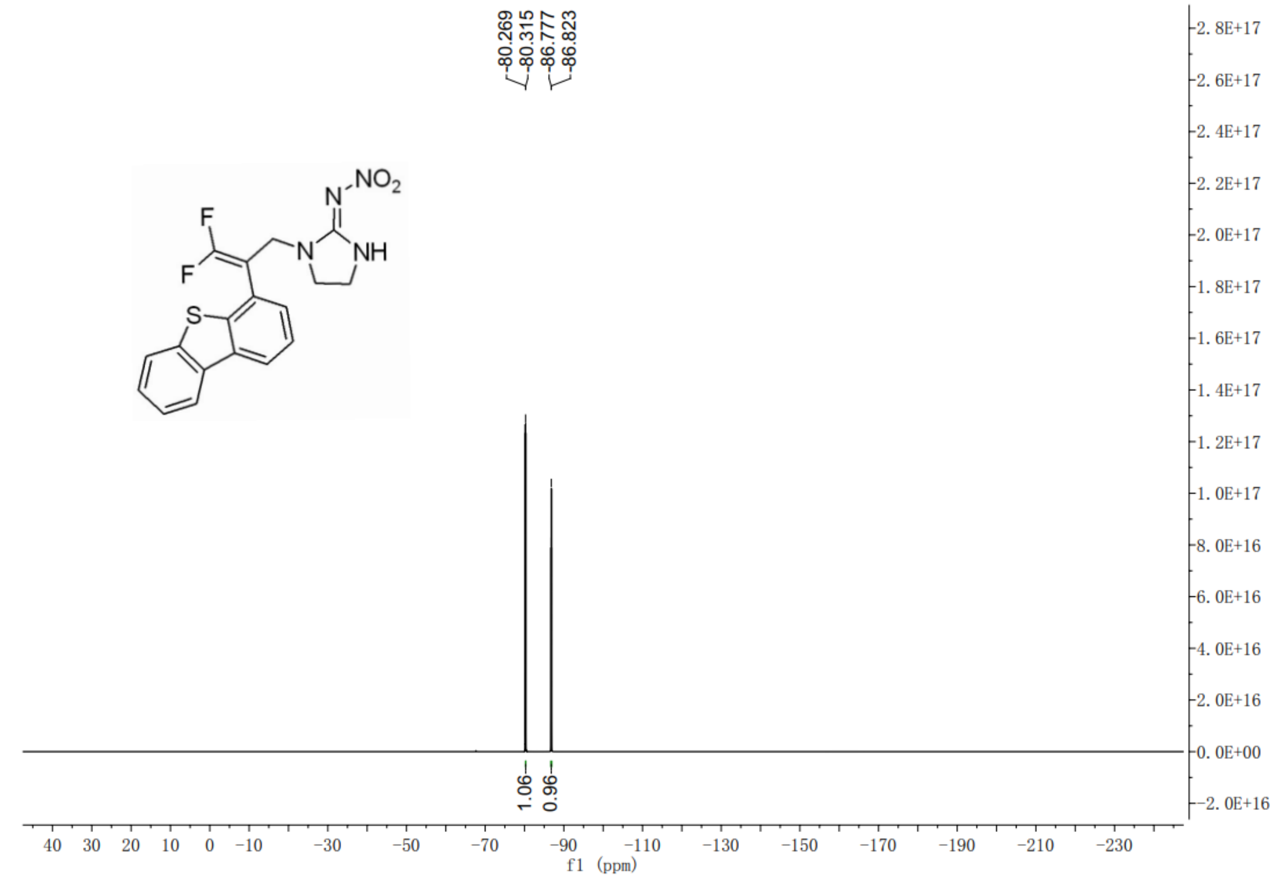

HRMS (ESI) spectrum of 4wa

Monoisotopic Mass, Even Electron Ions
81 formula(e) evaluated with 1 results within limits (all results (up to 1000) for each mass)
Elements Used:
C: 18-18 H: 14-16 N: 0-8 O: 0-6 F: 2-3 Na: 1-1 S: 1-1
15
221219-2-2 20 (0.130)

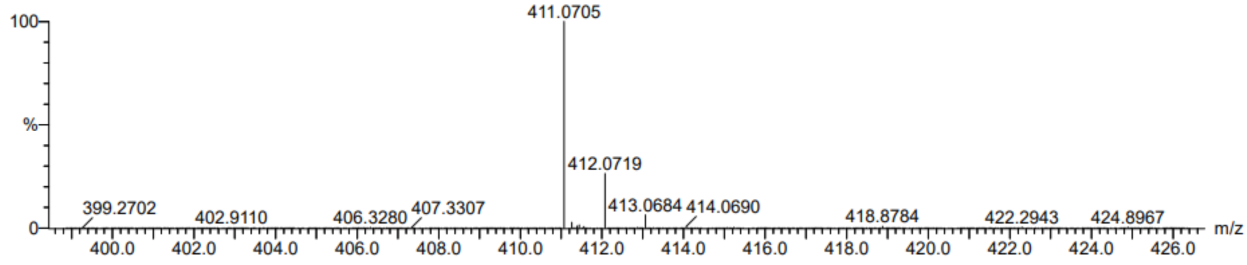

Minimum:				-1.5				
Maximum:		5.0	50.0	50.0				
Mass	Calc. Mass	mDa	PPM	DBE	i-FIT	Norm	Conf(%)	Formula
411.0705	411.0703	0.2	0.5	12.5	571.9	n/a	n/a	C18 H14 N4 O2 F2 Na S

^{1}H NMR spectrum of 4gc (400 MHz, $CDCl_3$)

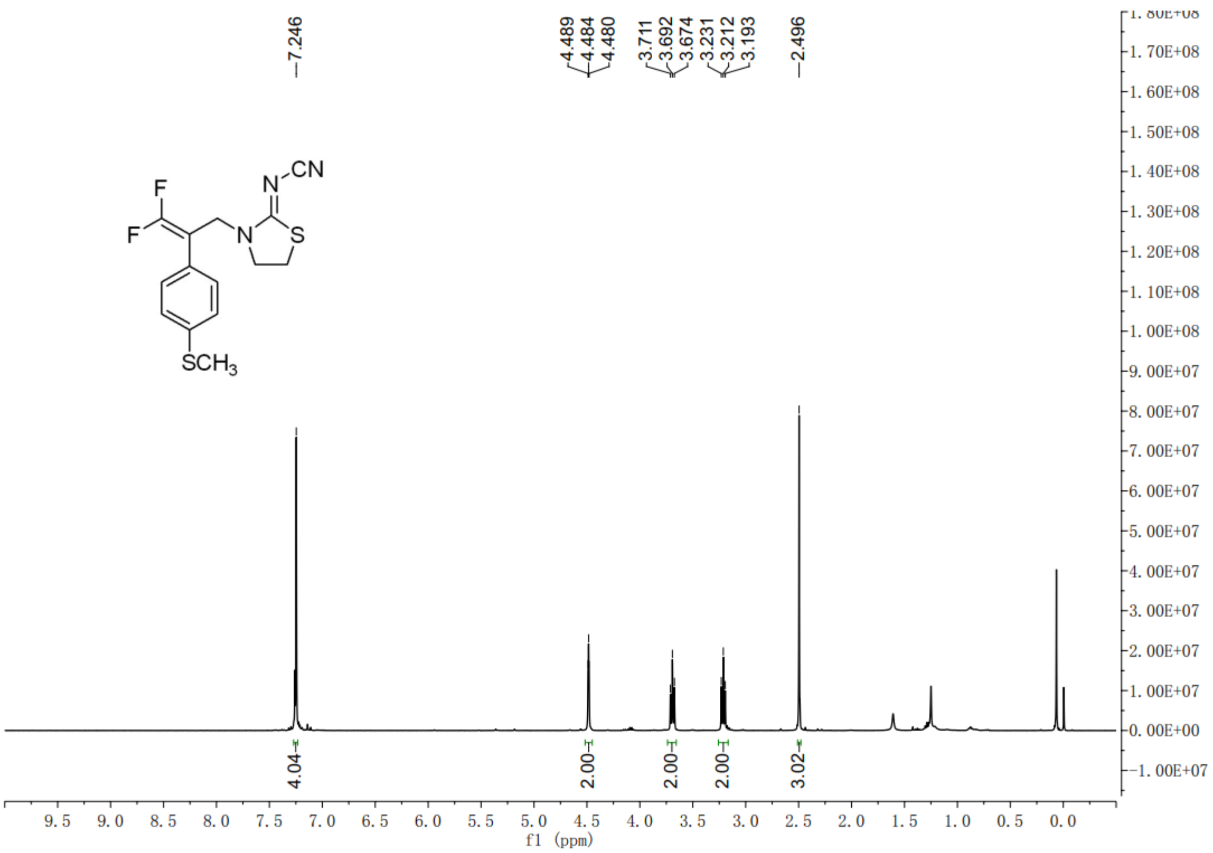

^{13}C NMR spectrum of 4gc (100 MHz, $CDCl_3$)

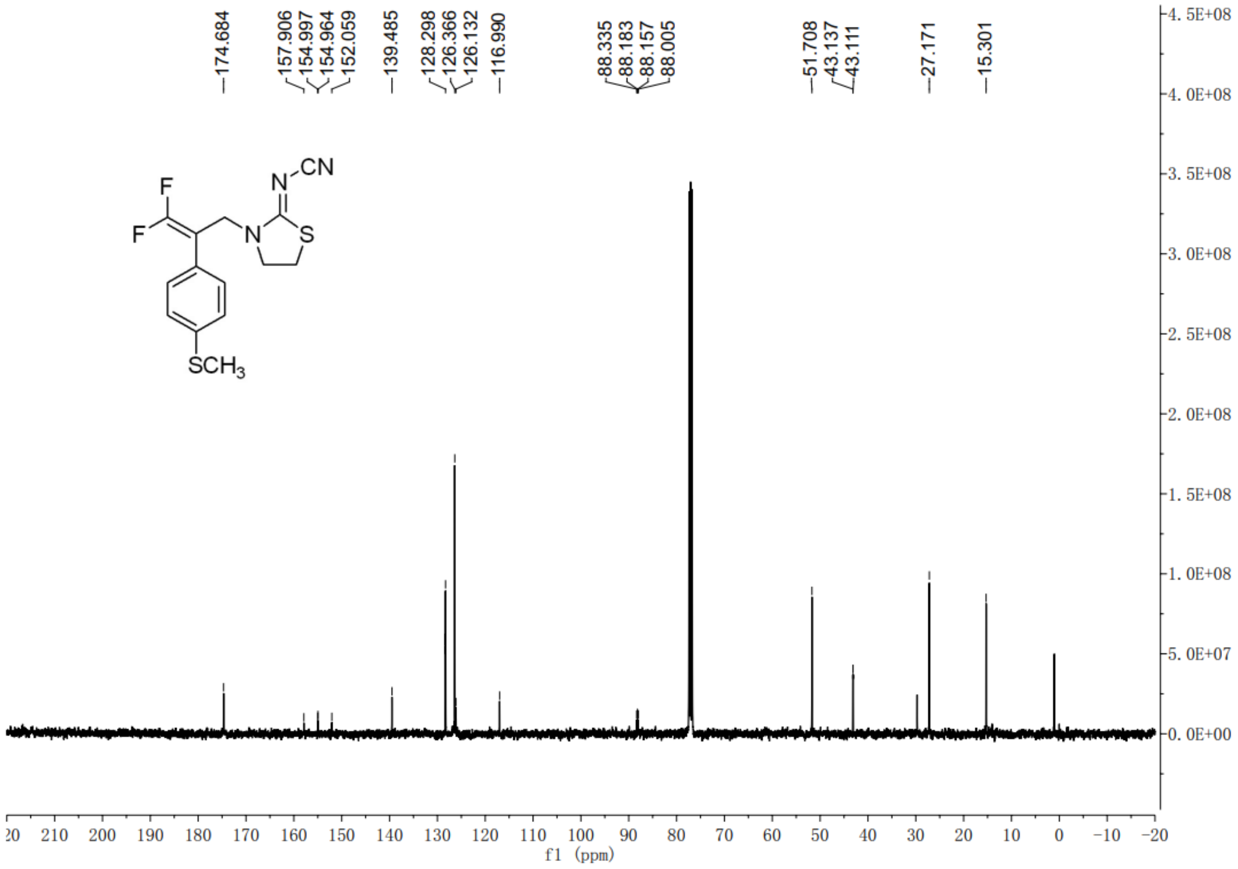

^{19}F NMR spectrum of 4gc (564 MHz, $CDCl_3$)

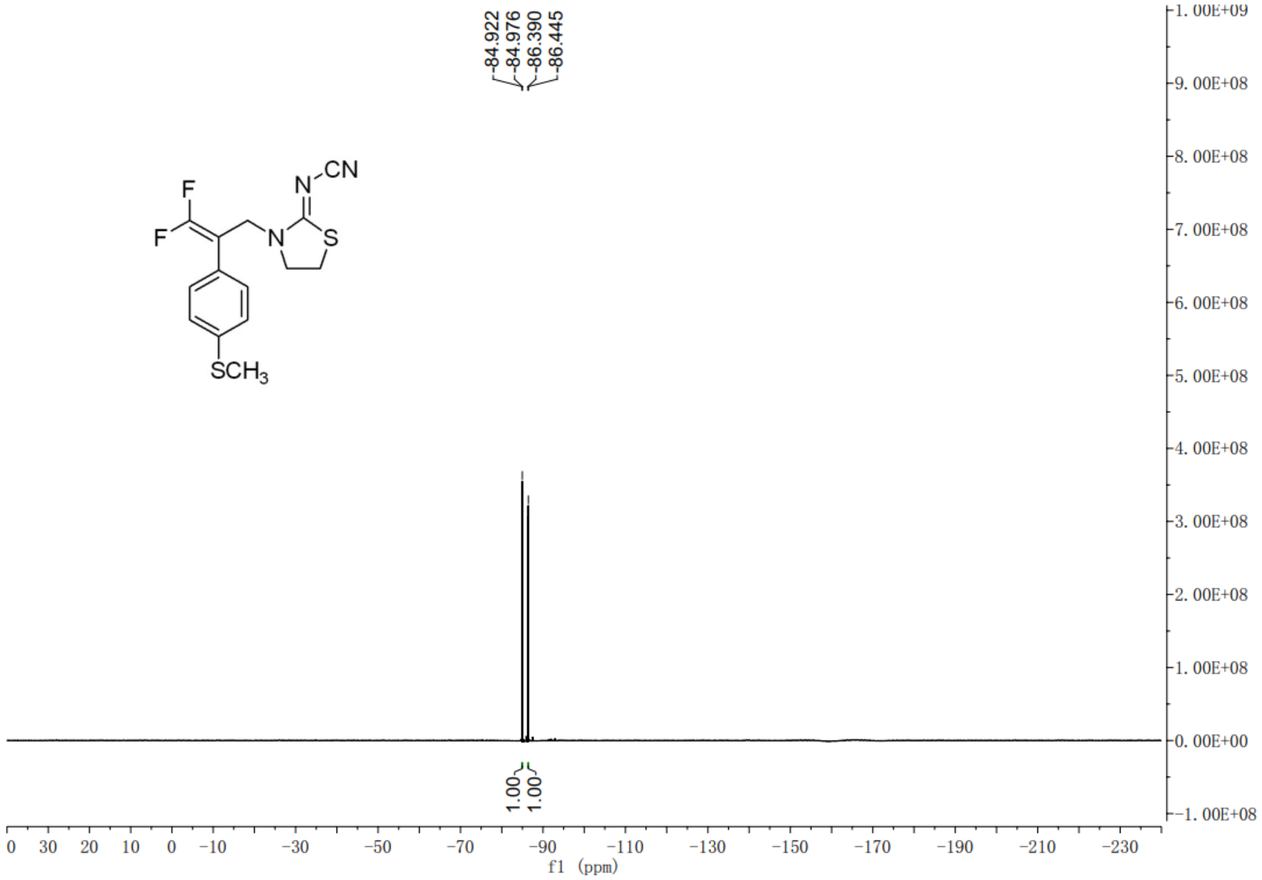

HRMS (EI) spectrum of 4gc

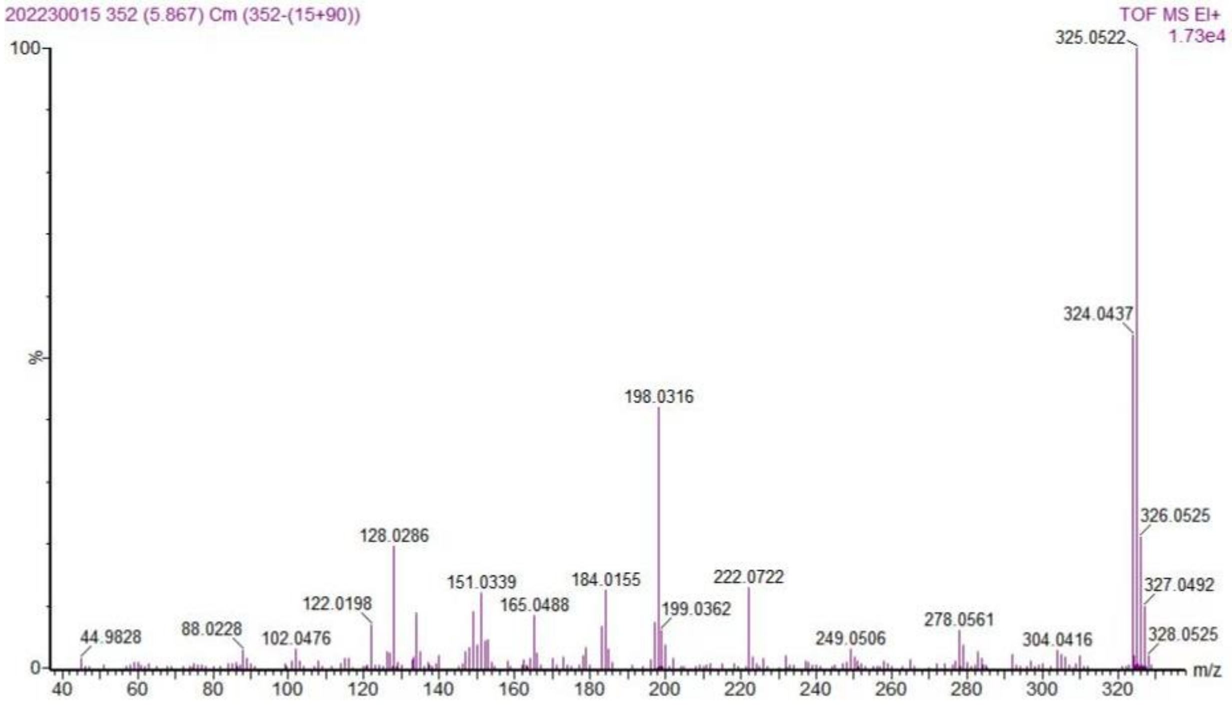

^{1}H NMR spectrum of 4hc (400 MHz, $CDCl_3$)

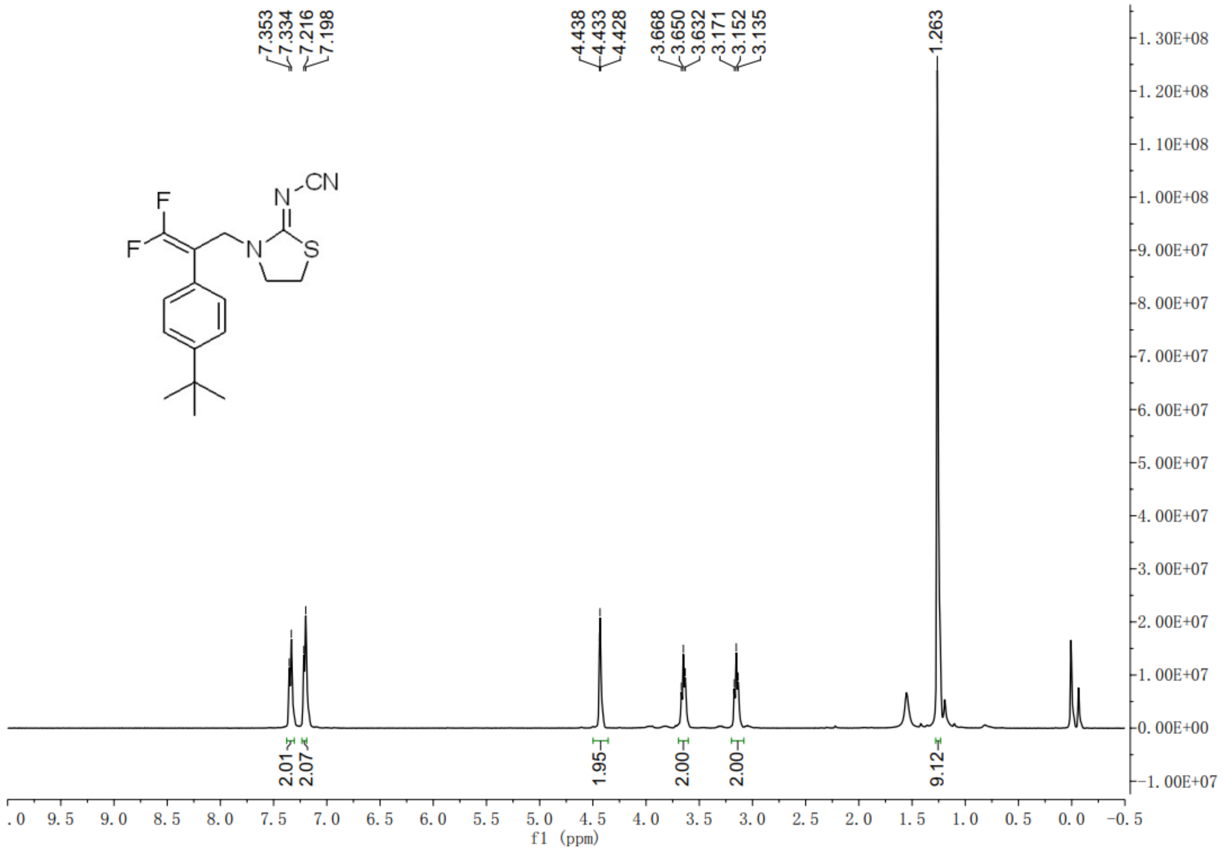

^{13}C NMR spectrum of 4hc (100 MHz, $CDCl_3$)

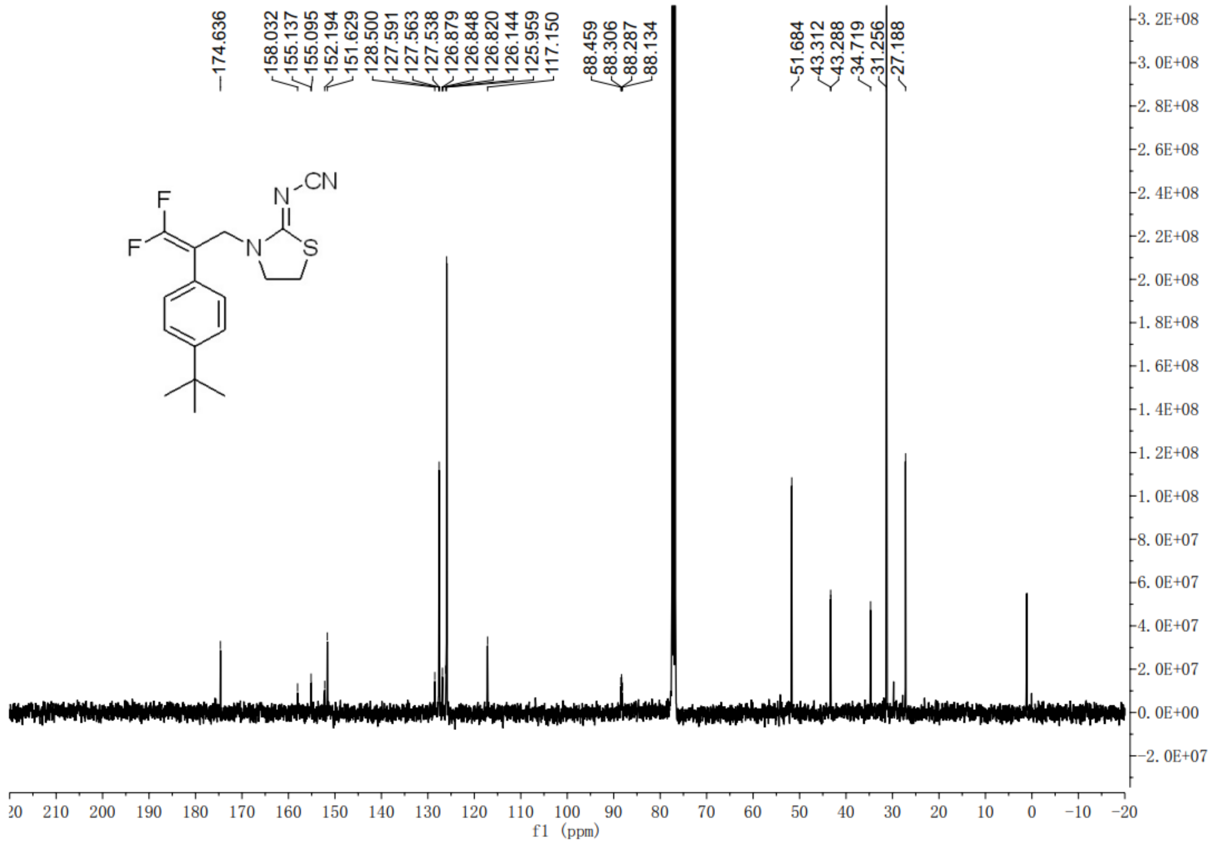

^{19}F NMR spectrum of 4hc (564 MHz, $CDCl_3$)

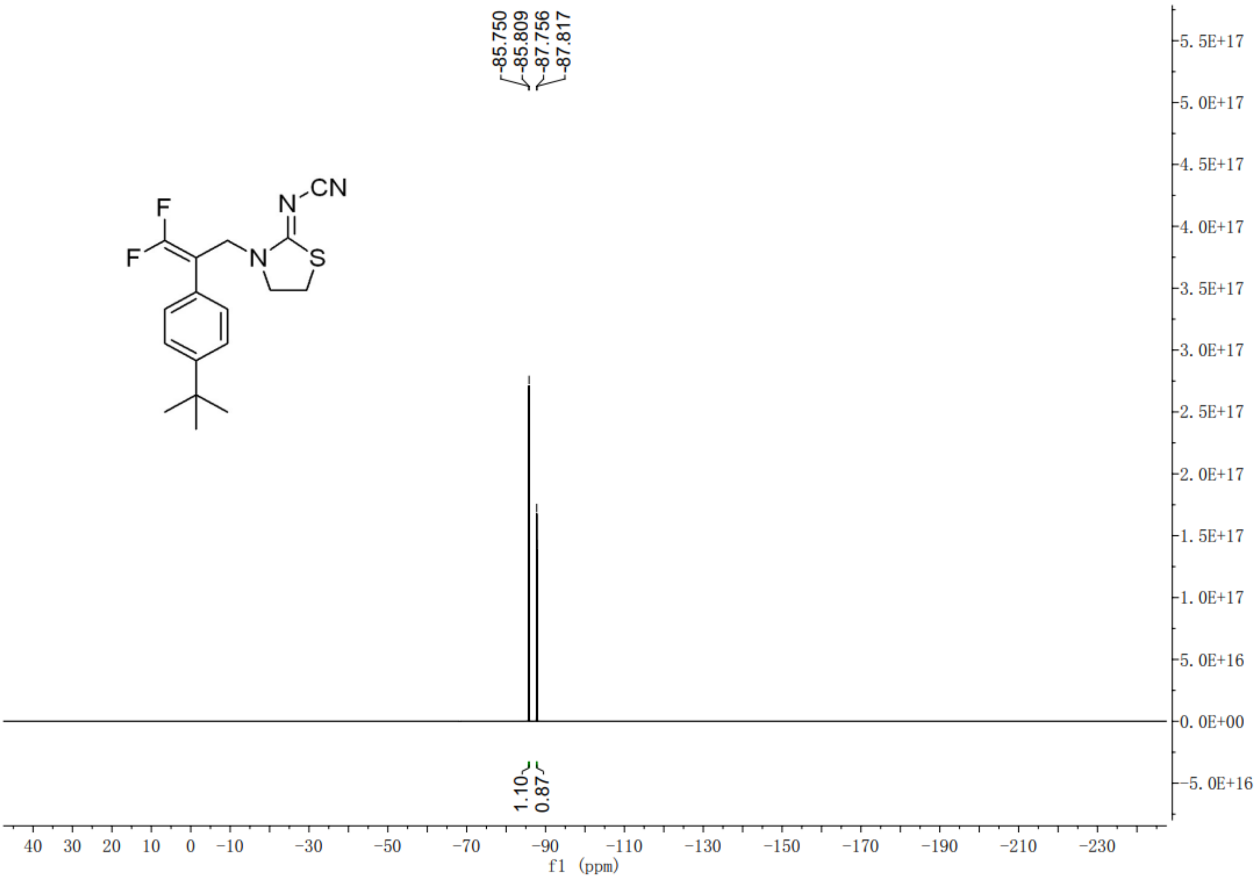

HRMS (EI) spectrum of 4hc

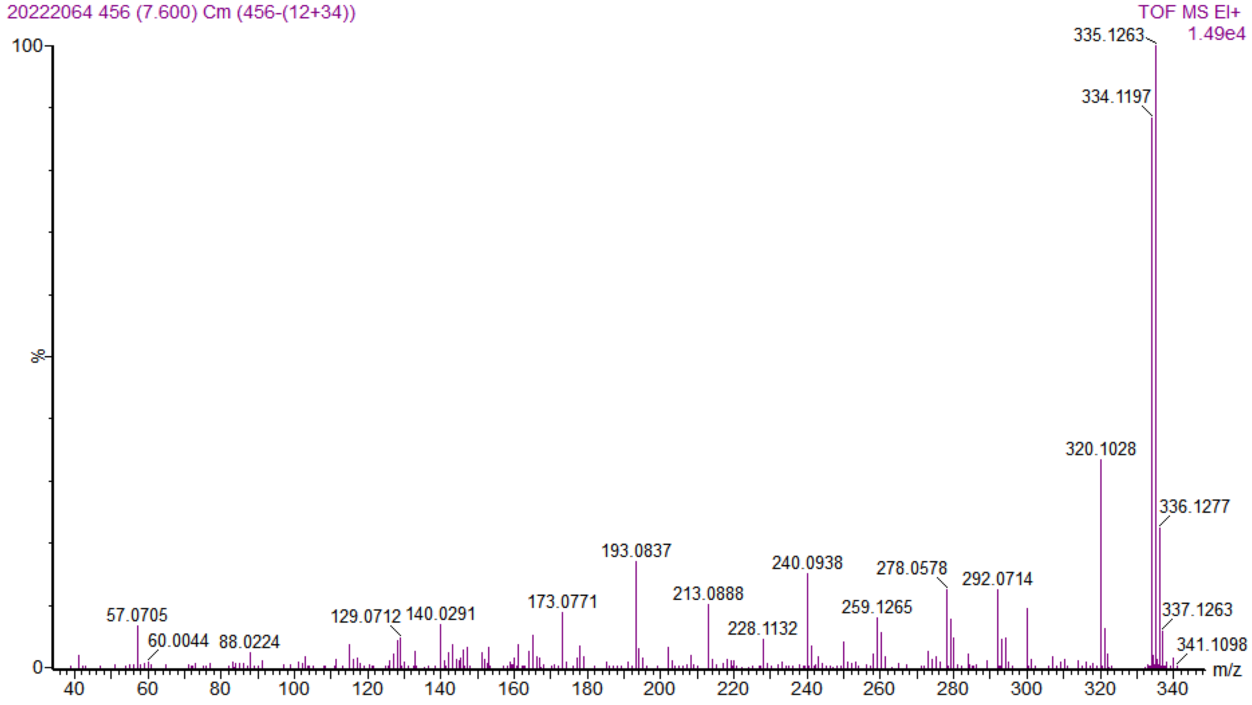

^{1}H NMR spectrum of 4xc (400 MHz, CDCl$_3$)

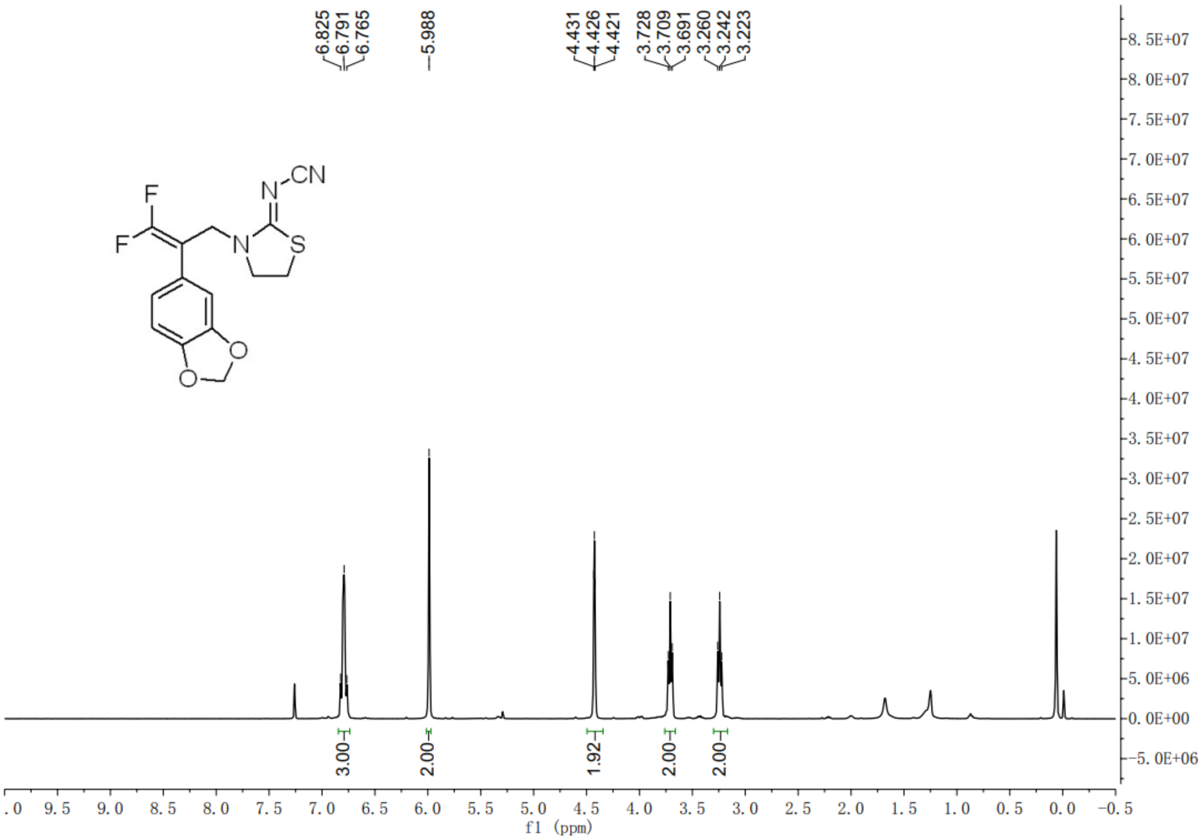

^{13}C NMR spectrum of 4xc (100 MHz, CDCl$_3$)

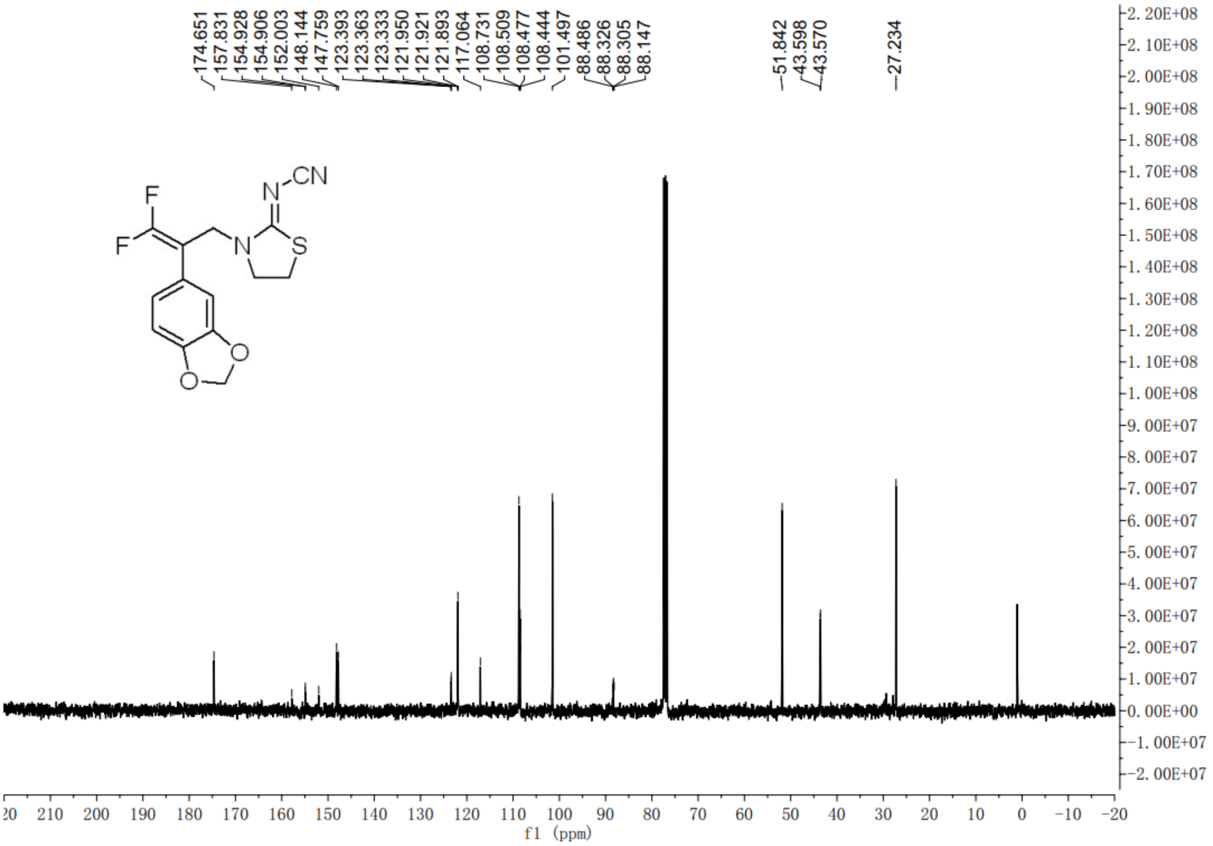

^{19}F NMR spectrum of 4xc (564 MHz, $CDCl_3$)

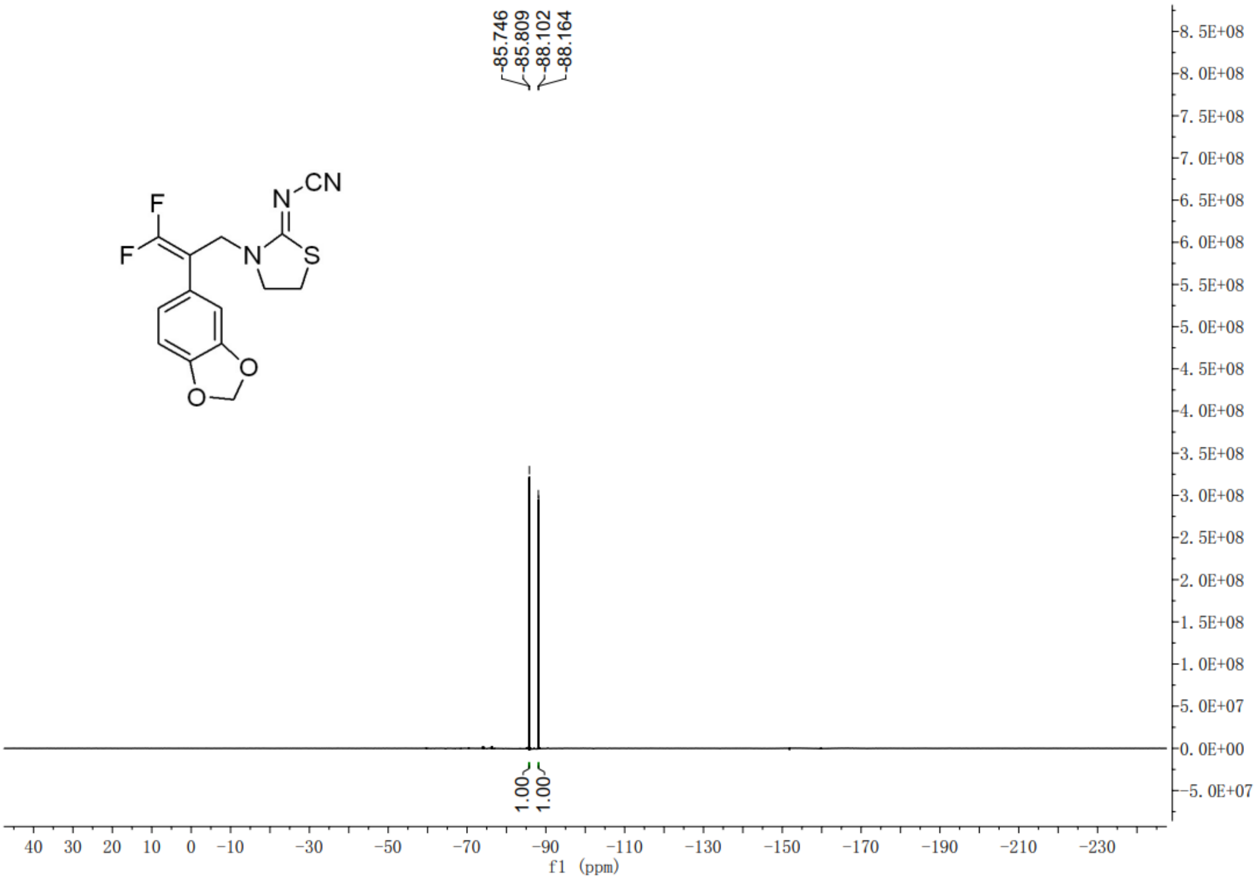

HRMS (EI) spectrum of 4xc

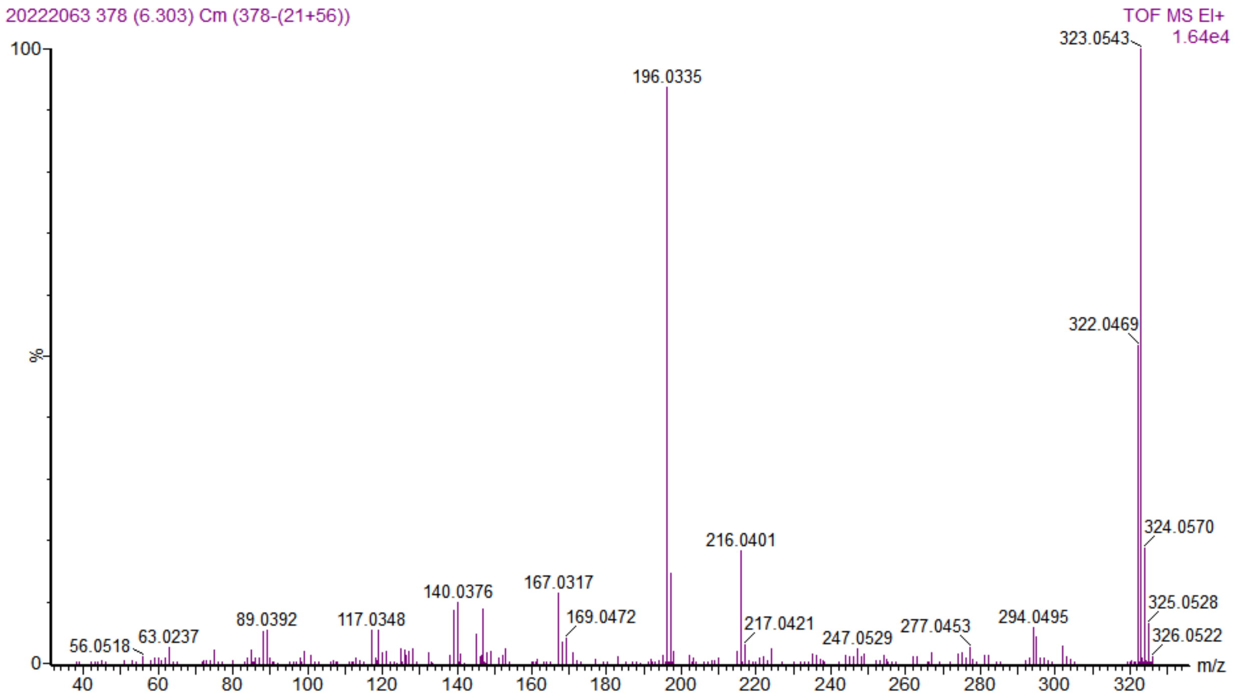

^{1}H NMR spectrum of 6 (400 MHz, acetone-d_6)

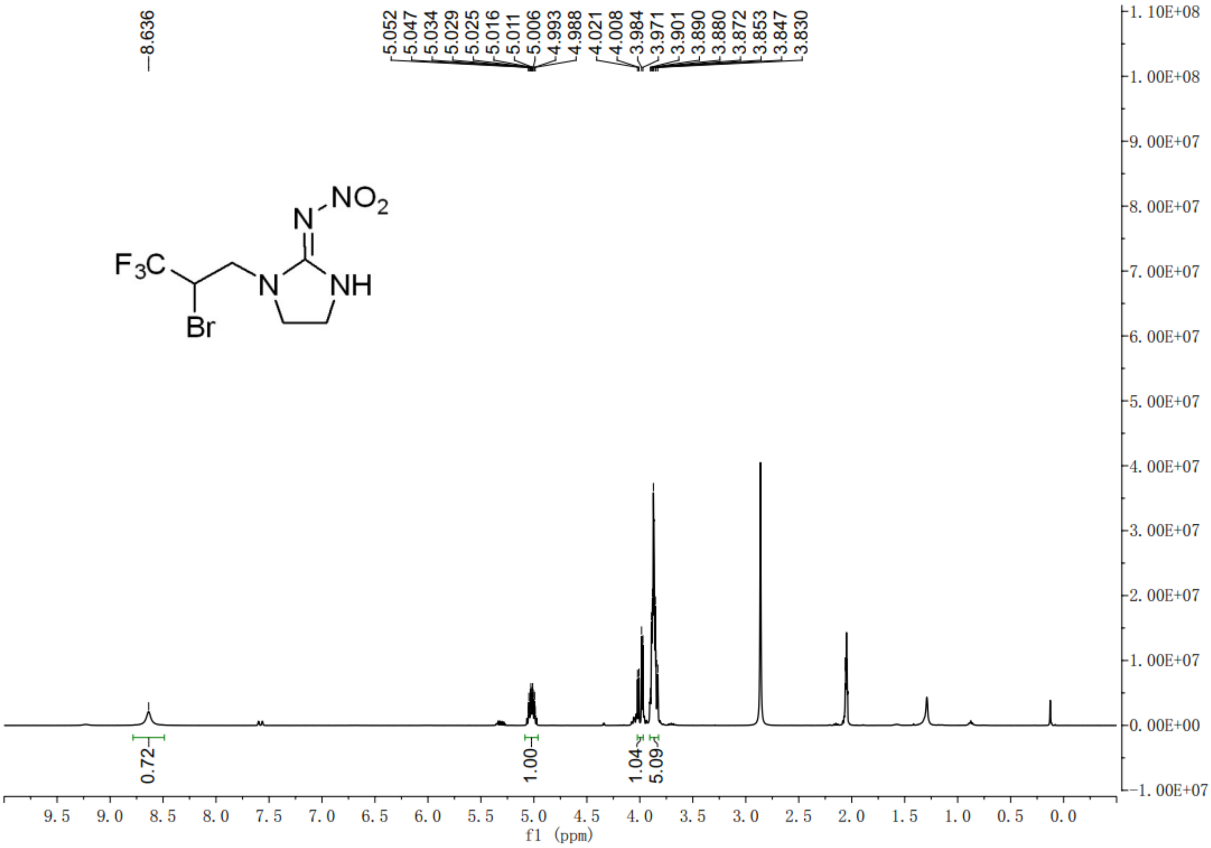

^{13}C NMR spectrum of 6 (100 MHz, acetone-d_6)

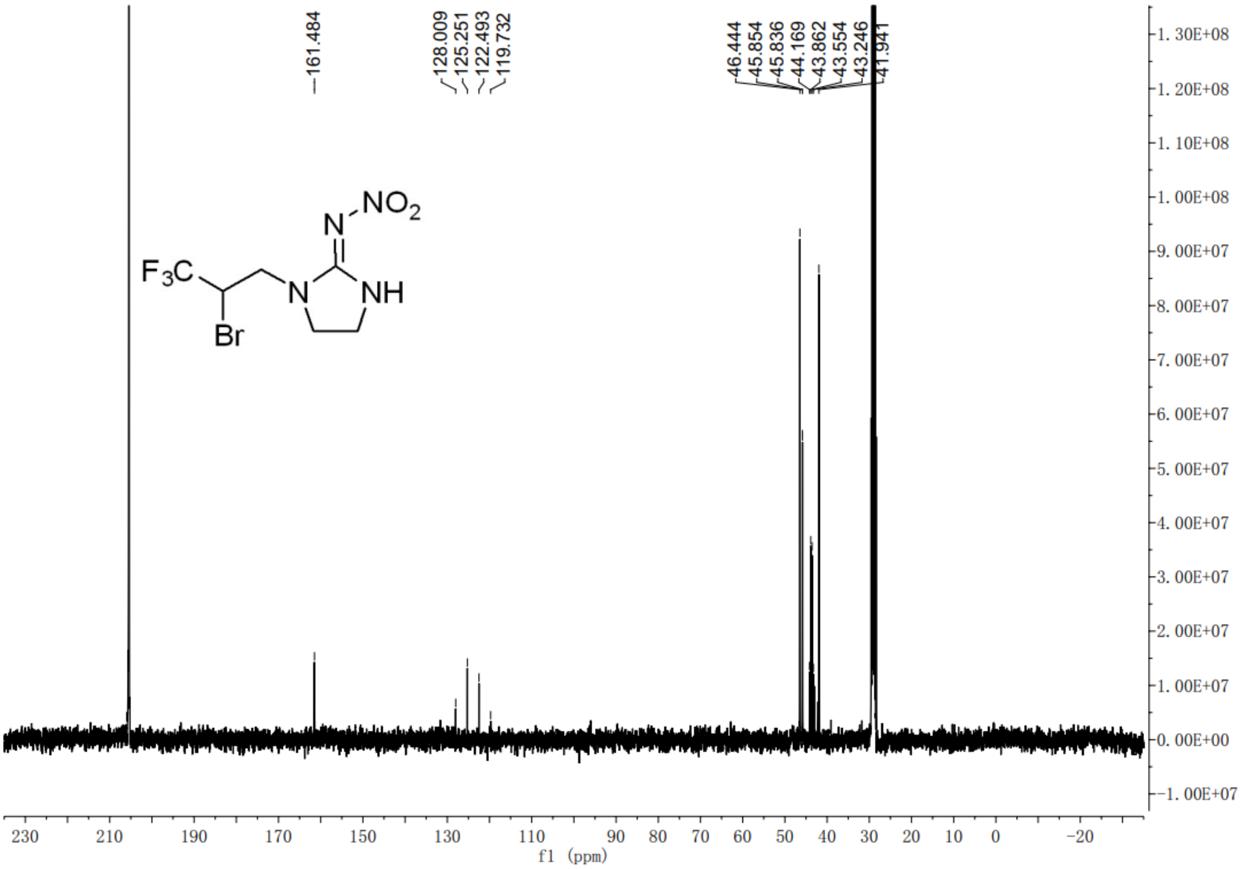

^{19}F NMR spectrum of 6 (564 MHz, acetone-d_6)

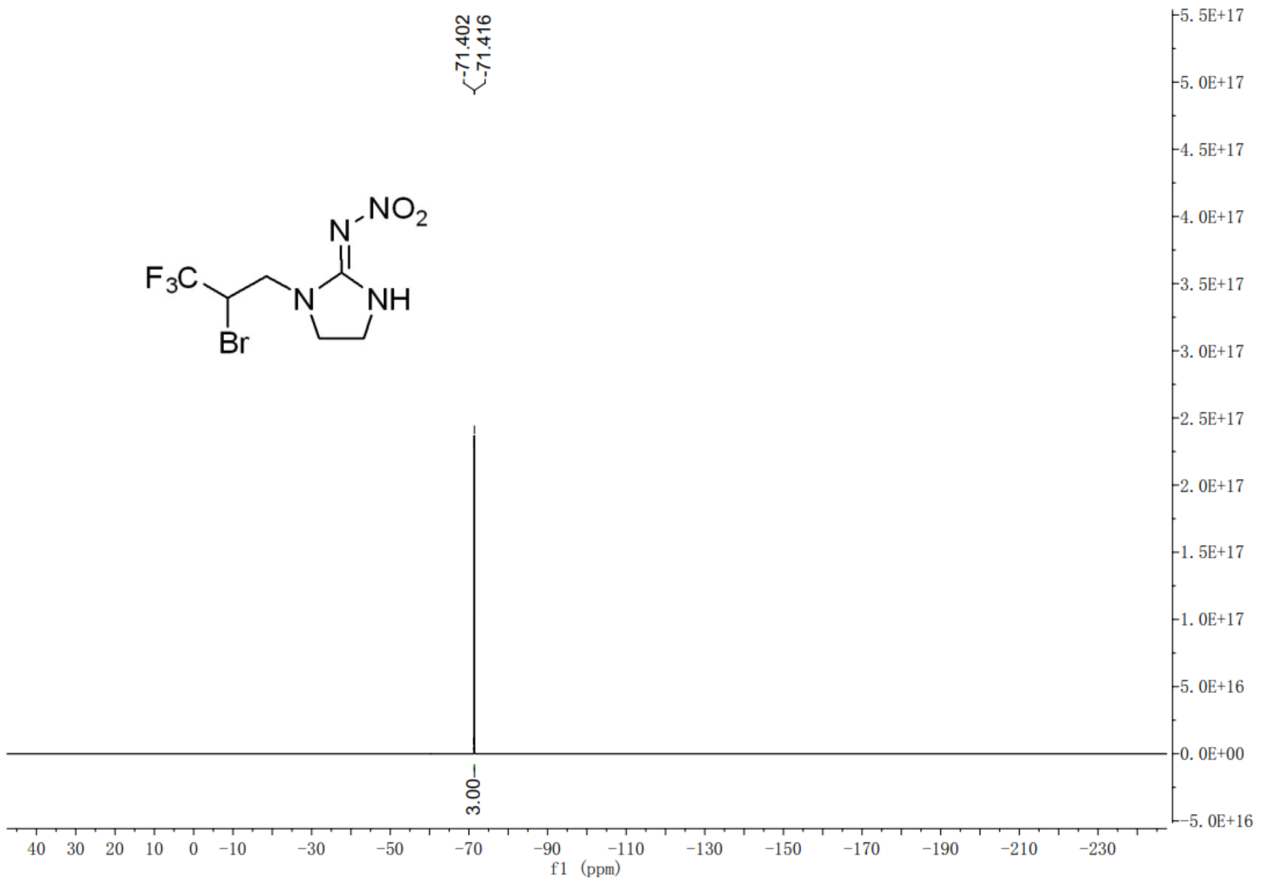

HRMS (EI) spectrum of 6

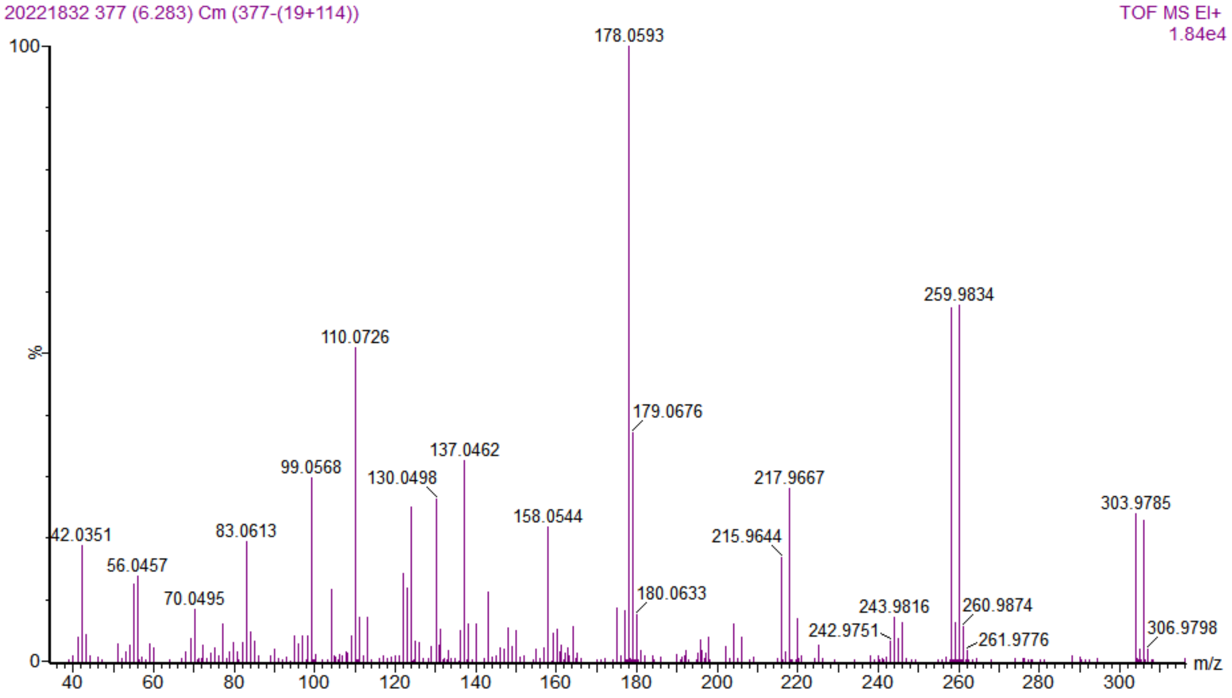

Supplement: Supplementary file 1 [file molecules-28-03530-s001.zip › molecules-2323732-supplementary.pdf]
